# Morphogenesis and development of human telencephalic organoids in the absence and presence of exogenous extracellular matrix

**DOI:** 10.15252/embj.2022113213

**Published:** 2023-10-16

**Authors:** Catarina Martins‐Costa, Vincent A Pham, Jaydeep Sidhaye, Maria Novatchkova, Andrea Wiegers, Angela Peer, Paul Möseneder, Nina S Corsini, Jürgen A Knoblich

**Affiliations:** ^1^ Institute of Molecular Biotechnology of the Austrian Academy of Sciences (IMBA), Vienna BioCenter Vienna Austria; ^2^ Vienna BioCenter PhD Program Doctoral School of the University of Vienna and Medical University of Vienna Vienna Austria; ^3^ Department of Neurology Medical University of Vienna Vienna Austria

**Keywords:** corticogenesis, ECM, human neural organoids, Matrigel, neuroepithelium, Development, Neuroscience

## Abstract

The establishment and maintenance of apical‐basal polarity is a fundamental step in brain development, instructing the organization of neural progenitor cells (NPCs) and the developing cerebral cortex. Particularly, basally located extracellular matrix (ECM) is crucial for this process. *In vitro*, epithelial polarization can be achieved via endogenous ECM production, or exogenous ECM supplementation. While neuroepithelial development is recapitulated in neural organoids, the effects of different ECM sources in tissue morphogenesis remain underexplored. Here, we show that exposure to a solubilized basement membrane matrix substrate, Matrigel, at early neuroepithelial stages causes rapid tissue polarization and rearrangement of neuroepithelial architecture. In cultures exposed to pure ECM components or unexposed to any exogenous ECM, polarity acquisition is slower and driven by endogenous ECM production. After the onset of neurogenesis, tissue architecture and neuronal differentiation are largely independent of the initial ECM source, but Matrigel exposure has long‐lasting effects on tissue patterning. These results advance the knowledge on mechanisms of exogenously and endogenously guided morphogenesis, demonstrating the self‐sustainability of neuroepithelial cultures by endogenous processes.

## Graphical abstract

Human telencephalic organoids cultured in the absence of Matrigel endogenously produce and self‐organize the extracellular matrix, without affecting global programs of neuronal differentiation.

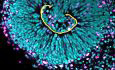

## Introduction

Epithelial morphogenesis is an essential step in the development of several organs, including the brain (Arai & Taverna, 2017; Hakanen *et al*, 2019). In the central nervous system, crucial morphogenic steps take place during neurulation, when the neural plate gives rise to the neural tube (Colas & Schoenwolf, 2001; Eom *et al*, 2013; Hakanen *et al*, 2019). At that stage, polarized neuroepithelial cells present an apical domain adjacent to the fluid‐filled ventricular lumen and a basal domain at the outer neural tube surface (Colas & Schoenwolf, 2001). Analogous apical‐basal organization is later found during corticogenesis, when the polarization of apical radial glia enables the stratified organization of other progenitor and neuronal populations across the cortical plate (Arai & Taverna, 2017). Impairment in these processes leads to severe neurodevelopmental defects (Hakanen & Salminen, 2015).

Polarization steps rely on coordinated signaling from neighboring cells and extracellular matrix (ECM) proteins. In particular, basement membrane proteins are cell surface‐associated ECMs that line the basal surface of epithelial tissues, providing cues that initiate and maintain polarity (Henry & Campbell, 1998; Colognato *et al*, 1999; Miner & Yurchenco, 2004; Datta *et al*, 2011). In the brain, ECM production is carried out by different cell types, such as the meninges (Decimo *et al*, 2021) and neural progenitor cells (NPCs) themselves, and ECM signaling lies at the core of neurodevelopmental processes that differ between mice and humans (Fietz *et al*, 2012; Florio & Huttner, 2014; Long *et al*, 2018; Namba *et al*, 2019). Despite their central role in neurodevelopment (Long & Huttner, 2019; Amin & Borrell, 2020), processes of ECM production and cell–ECM interactions remain understudied in cell culture models of the developing human brain.

Key features of neuroepithelium generation, morphogenesis, and differentiation can be recapitulated with neural organoids, three‐dimensional models of developing human brain regions (Quadrato & Arlotta, 2017; Pasca, 2018; Sidhaye & Knoblich, 2021; Eichmüller & Knoblich, 2022). *In vitro* systems of various epithelial tissues are able to model aspects of endogenous morphogenesis with remarkable accuracy, but often lack endogenous ECM production, therefore requiring exogenous ECM supplementation (Inman & Bissell, 2010; Simian & Bissell, 2017; Kratochvil *et al*, 2019; Corsini & Knoblich, 2022). Accordingly, the vast majority of protocols used for brain organoid generation resorts to the early exposure of embryoid bodies (EBs) to exogenous ECM in the form of Matrigel, which has been proposed as an adjuvant to the process of neuroepithelium development (Qian *et al*, 2018; Velasco *et al*, 2019; Bhaduri *et al*, 2020; Esk *et al*, 2020; Eichmüller *et al*, 2022; He *et al*, 2022; Kelava *et al*, 2022; Paulsen *et al*, 2022; Villa *et al*, 2022). However, certain methodologies entirely forgo the addition of exogenous ECM (Eiraku *et al*, 2008; Sakaguchi *et al*, 2015; Yoon *et al*, 2019; Gordon *et al*, 2021). Currently, it is not known how these distinct experimental paradigms impact neuroepithelial development *in vitro*.

Here, we examined the effects of ECM exposure during human telencephalic organoid development. We resorted to Matrigel, an ECM preparation extracted from murine Engelbreth‐Holm‐Swarm (EHS) sarcomas (Orkin *et al*, 1977) and mainly composed of ECM proteins—including laminin (60%), collagen IV (30%), entactin (8%), fibronectin, and heparan sulfate proteoglycan—and growth factors (Corning Incorporated Life Sciences, 2016). Matrigel was used in the formation of early 3D organoid‐like cultures, such as mammary gland alveolar structures (Barcellos‐Hoff *et al*, 1989; Simian & Bissell, 2017), and later to support *in vitro* culture of organoids from intestinal (Sato *et al*, 2009), brain (Lancaster *et al*, 2013), and other epithelial tissues (Eiraku *et al*, 2011; Nakano *et al*, 2012; Huch *et al*, 2013; Stange *et al*, 2013; Dorrell *et al*, 2014; Boretto *et al*, 2017; Nie *et al*, 2017; Turco *et al*, 2017; Jeong *et al*, 2021; Kim *et al*, 2021; Gurumurthy *et al*, 2022). To gain insight into the mechanisms of ECM action, we further tested organoid exposure to the most abundant components of Matrigel, namely purified Laminin and Collagen IV. Finally, we used an experimental setup without any exogenous ECM supplementation. We conclude that Matrigel, but not single purified ECM components, highly affects early neuroepithelial morphogenesis by rapidly establishing an apical‐basal polarity axis; it further increases tissue mis‐patterning by upregulating transcriptional pathways of eye development. In the absence of Matrigel, neuroepithelial cells endogenously produce and self‐organize ECM components while acquiring a homogenous telencephalic fate. Global features of cell‐fate acquisition during long‐term organoid development are comparable in the presence or absence of Matrigel. With this systematic characterization, we have generated new insights into how the ECM influences neuroepithelial development *in vitro*, by exogenously or endogenously guided processes.

## Results

**Figure 1:**
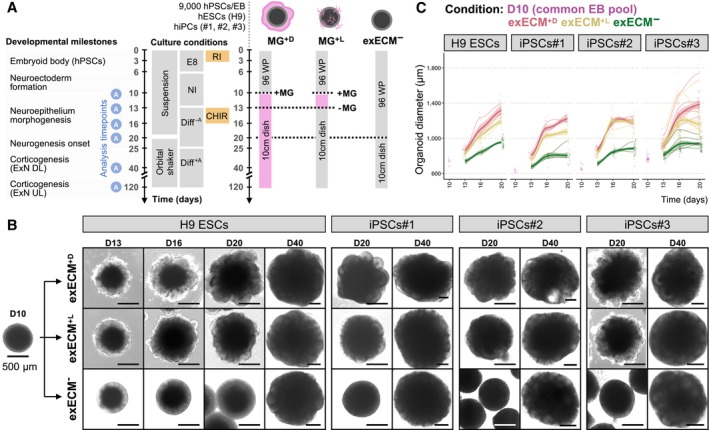
Matrigel exposure influences organoid morphology and growth in the first 20 days of development Summary of the three organoid protocols used, timepoints of analysis, and relevant developmental milestones. A common pool of EBs was split into three experimental conditions at D10: embedding in a droplet of Matrigel (MG^+D^), transient addition of Matrigel to the culture medium from D10 to D13 at a concentration of 2%V/V (MG^+L^), and no exposure to any exogenous ECM (exECM^−^). Experiments were performed with 4 human pluripotent stem cell lines (hPSCs) and analyzed at D10, D13, D16, D20, D40, and D120, when key developmental processes could be examined.Organoid morphology in the first 40 days of culture. Note the appearance of tissue budding in MG^+D^ and MG^+L^ at D16, and the comparable organoid morphology across conditions at D40. Scale bars: 500 μm. For representative images of iPSCs#1‐3‐derived organoids at D10‐16, see Appendix Fig S1A.Organoid diameter in the first 20 days of culture. For each condition and cell line, datapoints were fit to a smoothed trend line to visualize growth dynamics; the initial D10 timepoint was not fit to any condition, as it represents the initial pool of EBs. The colors indicate experimental condition; bold lines indicate the overall growth trend of all batches; the shaded area indicates the 95% confidence interval; thin lines indicate the growth trend of individual batches (biological replicates); single datapoints indicate individual organoids (technical replicates). 1,062 single organoids are plotted (Appendix Fig S1B). Source data are available online for this figure.

To evaluate how ECM supplementation influences early organoid development, we supplied exogenous ECM (exECM) in the form of Matrigel (MG) at the beginning of neuroepithelial development (day 10, D10). Matrigel application was done either as a solid droplet that provides long‐term exposure to a polymerized network of ECM proteins (droplet embedding, MG^+D^; Lancaster *et al*, 2017) or as transient dissolution in the culture medium (concentration of 2%V/V) from D10 to D13 (liquid embedding, MG^+L^). The latter is a technically simpler mode of Matrigel exposure that has been used in other organoid systems (Eiraku *et al*, 2011; Veenvliet *et al*, 2020; Hocevar *et al*, 2021; Sanaki‐Matsumiya *et al*, 2022) but so far not tested in early‐stage neural organoids. These protocols were compared to one without exposure to any exogenous ECM (exECM^−^; Fig 1A). To investigate the effects on the dorsal telencephalon, which has a well‐studied apical‐basal polarity, we provided a three‐day pulse of the GSK3β‐inhibitor CHIR99021 from D13 to D16, activating the Wnt pathway and guiding neuroepithelial differentiation as previously described (Lancaster *et al*, 2017). Four human pluripotent stem cell lines (hPSCs) from healthy donors were used—one embryonic stem cell line (ESCs; H9), and three induced pluripotent stem cell lines (iPSCs; #1, #2, and #3). Analysis timepoints corresponded to key milestones in organoid development (Fig 1A), starting with the different stages of neuroepithelial morphogenesis, a process strongly influenced by ECM signaling.

### Matrigel exposure influences early organoid morphology and growth dynamics

To assess general features of organoid morphology and size, we used longitudinal brightfield microscopy imaging (Fig 1B, and Appendix Fig S1A and B). At D10, the EBs had a smooth circular shape and brightening of the outer rim of the tissue, indicating that neuroepithelium formation had started (Fig 1B). Morphological changes were rapidly observed in the presence of Matrigel (Fig 1B and Appendix Fig S1A; MG^+D^ and MG^+L^). Organoids appeared irregularly shaped at D13, and tissue budding was visible from D16, most prominently in MG^+D^ conditions. In the absence of Matrigel, organoids remained spherical and maintained the outer brightening during the first 20 days (Fig 1B and Appendix Fig S1A; exECM^−^). The organoid diameter was measured from over 1,000 images (Fig 1C and Appendix Fig S1B). At D10, the organoid diameter varied between 600 and 800 μm. From D13 to D20, the organoid diameter increased more prominently in MG^+D^, followed by MG^+L^, and finally exECM^−^ conditions (Fig 1C). These growth dynamics were reproducible across different batches of the same cell line within each experimental condition, showing slight variation across all four cell lines. Remarkably, evident morphological differences gradually vanished, and organoids appeared identical across conditions and cell lines at D40, with clear tissue budding also in exECM^−^ organoids (Fig 1B and Appendix Fig S1A, D40). Thus, the presence and concentration of Matrigel impacted the shape and size of organoids during neuroepithelium generation and expansion (first 20 days of development) but organoids later converged to a similar tissue architecture.

### Matrigel exposure promotes a fast rearrangement of neural progenitors

**Figure 2:**
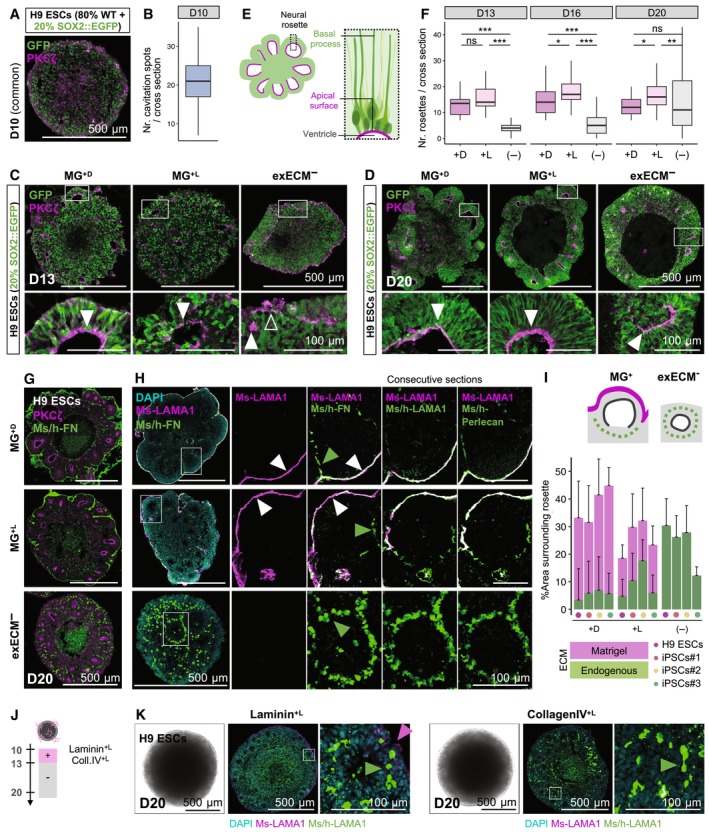
Neuroepithelial polarization can be driven by Matrigel or by endogenous self‐organization of ECM proteins A–DOrganoids generated with 80% WT H9 ESCs and 20% SOX2::EGFP were used to visualize the organization of neural progenitors. (A) Immunostaining for PKCζ and GFP at D10. (B) Quantification of the number of cavitation spots per cross section, using 45 organoids (see results for individual organoids of all cell lines in Appendix Fig S2A). Immunostaining for PKCζ and GFP at D13 (C), and D20 (D). Bottom panels: magnification of inset. Arrowheads mark the location of PKCζ lining the organoid outer surface (empty arrowheads) or the ventricular zone of neural rosettes (filled arrowheads).ESchematic representation of the radial organization of neural progenitors in neural rosettes, seen in all conditions at D20.FQuantification of the number of neural rosettes per cross section at D13 (110 organoids), D16 (160 organoids), and D20 (148 organoids; see results for individual organoids of all cell lines and timepoints in Appendix Fig S2B–D). Boxplots mark the median value; the two hinges correspond to the first and third quartiles (the 25^th^ and 75^th^ percentiles); and the whiskers extend from the hinge to the highest/lowest value no further than 1.5*IQR from the hinge (where IQR is the inter‐quartile range, or distance between the first and third quartiles). Statistical tests are analysis of variance (ANOVA); 0 ≤ *P* < 0.001, ***; 0.001 ≤ *P* < 0.01, **; 0.01 ≤ *P* < 0.05, *; *P* ≥ 0.05, ns (see results of statistical tests in Appendix Table S1).GImmunostaining for PKCζ and Ms/h‐FN, marking the apical and basal domains of neural rosettes, respectively. For a zoomed‐in view and representative images of all cell lines from D13 to D20, see Appendix Fig S3B.HImmunostaining for ECM proteins. The Ms‐LAMA1 antibody can be used to identify mouse‐derived ECM (Matrigel). At D20, MG^+D^, and MG^+L^ organoids show a coating of Ms‐LAMA1 originating from Matrigel, which co‐localizes with Ms/h‐FN, LAMA1, and Perlecan (white arrowheads); also, FN^+^ but Ms‐LAMA1^−^ speckles are seen within the tissue, indicating endogenously produced FN (green arrowheads). ExECM^−^ organoids show abundant endogenously produced ECM surrounding neural rosettes (green arrowheads).IQuantification of the area around rosettes covered by Matrigel‐derived (Ms‐LAMA1^+^) and endogenously derived ECM (Ms‐LAMA1^−^Ms/h‐FN^+^), using 88 organoids and 276 rosettes (see methodology and results for individual organoids of all cell lines in Appendix Fig S4). The whiskers represent the positive standard deviation from the mean value (mean + SD).JExperimental paradigm to test the effect of liquid embedding with purified Laminin or Collagen IV.KBrightfield imaging and co‐staining of Ms‐LAMA1 (magenta arrowheads) and Ms/h‐LAMA1 (green arrowheads) of Laminin^+L^ and Coll.IV^+L^ organoids at D20. For representative images at D13‐20, see Appendix Fig S6. Source data are available online for this figure.

To understand how Matrigel exposure alters neuroepithelial morphology, we assessed the early organization of neural progenitors. To visualize the position of individual NPCs within the tissue, we used an H9‐derived reporter cell line in which the expression of the green fluorescent protein was driven by the SOX2 promoter (SOX2::SOX2‐p2A‐EGFP, hereafter SOX2::EGFP), marking bona fide neural progenitors (Sidhaye *et al*, 2022). We analyzed organoids containing 80% H9 wild‐type (WT) ESCs and 20% H9 SOX2::EGFP ESCs, as this mixing ratio was sparse enough to allow the recognition of individual SOX2::EGFP NPCs while also revealing their overall tissue distribution (Fig 2A, C and D).

To mark the neuroepithelial apical domain, we used immunostaining of a member of the atypical protein kinase C subfamily (PKCζ; Soriano *et al*, 2016). At D10, small hollow regions—cavitation spots—were present throughout the EB tissue, indicating initial points of structural asymmetry (Fig 2A and B, and Appendix Fig S2A). However, SOX2::EGFP^+^ neural progenitors appeared scattered, lacking a clear organization, and the direction of apical‐basal polarity was yet undefined, with PKCζ staining lining both external and internal surfaces (Fig 2A). Exposure to Matrigel led to rapid tissue rearrangements. Within 3 days (Fig 2C, D13), neural progenitors in MG^+D^ and MG^+L^ organoids were organized in neural rosettes, acquiring an elongated morphology around large internal lumina (ventricular zones) delimited by a PKCζ^+^ apical surface (Fig 2C, filled arrowhead; schematized in Fig 2E). In contrast, exECM^−^ organoids maintained outer apical domains (Fig 2C; empty arrowhead) and few small internal lumina (Fig 2C; filled arrowhead). At D20, however, neural rosettes with larger lumina and radial progenitor arrangement were also widespread in exECM^−^ organoids (Fig 2D; filled arrowhead). To quantify these observations, we counted the number of PKCζ^+^ neural rosette lumina per cross section, from D13 to D20, in over 400 organoids of all cell lines (Fig 2F and Appendix Fig S2B–D). The induction of rosette formation was very efficient in liquid embedding mode, as seen by comparable or higher number of neural rosettes in MG^+L^ than in MG^+D^ organoids from D13 to D20 (Fig 2F and Appendix Fig S2B–D). In exECM^−^ organoids, the number of neural rosettes was initially lower (Appendix Fig S2B and C) but, by D20, was comparable among all conditions in most cell lines (Appendix Fig S2D). Thus, Matrigel exposure caused fast changes in tissue polarity and NPC organization, concomitant with the formation of neural rosettes. Interestingly, analogous reorganization happened in the absence of exogenous ECM with a delay of 5–7 days, suggesting that intrinsic self‐organization processes must be in place in exECM^−^ organoids.

### Exogenous or endogenous ECM proteins show organized distribution in neural rosettes

To better understand the timeline of NPC polarization, we assessed the location of PKCζ and Fibronectin (FN), markers of apical and basal domains, respectively (Fig 2G, and Appendix Fig S3A and B). The FN antibody recognized FN of mouse (Matrigel‐derived) and human (endogenously produced) origin (Ms/h‐FN). Embedding in a droplet of Matrigel led to the formation of a permanent basal domain on the outer organoid surface, as seen by the surrounding mesh of FN from D13 to D20 (Fig 2G and Appendix Fig S3B; MG^+D^). Matrigel dissolution in the culture medium led to the formation of a thin ECM coating at the organoid surface that remained visible even one week after the end of exposure (Fig 2G and Appendix Fig S3B, MG^+L^). A complete polarization of PKCζ^+^/FN^+^ surfaces was achieved between D13 and D16 in MG^+^ conditions (Appendix Fig S3B). Remarkably, exECM^−^ organoids showed abundant endogenous FN production from early developmental stages. Until D16, FN was mostly scattered across the tissue of exECM^−^ organoids (Appendix Fig S3A and B), whereas, at D20, an apical‐basal axis was established, with circular arrangement of FN^+^ regions around PKCζ^+^ lumina of neural rosettes (Fig 2G and Appendix Fig S3B). In summary, Matrigel established a clear basal‐out/apical‐in polarity axis from D13 and exECM^−^ organoids endogenously produced fibronectin that self‐organized around neural rosettes (schematized in Appendix Fig S3C).

The patterns of FN^+^ regions at D20 were very different in exECM^−^ and MG^+^ conditions (Fig 2G). To discriminate between ECM produced endogenously and ECM contributed by Matrigel, we used an antibody that recognizes mouse, but not human, laminin‐α1 (Ms‐LAMA1); together, we used antibodies that recognize both mouse and human (Ms/h) FN, LAMA1, and Perlecan (Fig 2H), known components of the brain ECM *in vivo* (Amin & Borrell, 2020). In exECM^−^ organoids, Ms‐LAMA1 was absent, as expected; Ms/h‐FN, LAMA1, and Perlecan showed overlapping expression, with a speckled pattern around neural rosettes that did not reach the outer‐most surface of the organoids—indicative of endogenously produced ECM (Fig 2H). In MG^+^ organoids, the smooth FN^+^LAMA1^+^Perlecan^+^ surface was co‐positive for Ms‐LAMA1, identifying Matrigel‐derived ECM; in addition, Ms/h‐ECM‐positive but Ms‐LAMA1‐negative speckles were seen within the tissue (Fig 2H). To quantify these observations, we analyzed 276 rosettes of 88 organoids from all cell lines, at D20. We segmented and measured the percentage of the area surrounding rosettes that was covered by endogenous or exogenous ECM (Fig 2I and Appendix Fig S4A–D). We show that there was a large proportion of MG‐derived ECM in both MG^+^ conditions, especially in MG^+D^ organoids, which were covered by a thicker Matrigel layer. In exECM^−^ conditions, the percentage of area covered by ECM was comparable to MG^+L^ organoids, but its origin entirely endogenous. To further address the production of ECM proteins absent from Matrigel, we assessed the presence and tissue distribution of Lumican (LUM), which is produced by human NPCs and plays an important role in cortical development *in vivo* (Long *et al*, 2018). LUM was abundant in organoids from early stages of development, and its tissue distribution followed a pattern like that of PKCζ: scattered and disordered at D10 (Appendix Fig S5A) and accumulated in rosette lumina from D13 in MG^+^ organoids (Appendix Fig S5B) and at D20 in all conditions (Appendix Fig S5C). Thus, Matrigel addition led to the formation of a sheet of ECM at the outermost organoid surface, distinguishable from, but not replacing, endogenously produced ECM within the tissue; in its absence, several ECM components were produced endogenously and underwent self‐organization in all organoids analyzed, corroborating the robustness of this process.

### Purified ECM components do not impact neuroepithelial morphogenesis

Given the effects of Matrigel on organoid morphology, we asked whether purified Matrigel protein components could mimic this action. We exposed organoids to mouse Laminin or Collagen IV, the two most abundant ECMs found in Matrigel (Corning Incorporated Life Sciences, 2016; Amin & Borrell, 2020). Since we observed changed organoid morphology both with Matrigel droplet embedding and with Matrigel liquid embedding, we used the same protocol as for MG^+L^ conditions, dissolving these proteins in the culture medium (2%V/V) from D13 to D16 (Fig 2J and Appendix Fig S6A, Laminin^+L^ and Coll.IV^+L^). To assess organoid morphology, we resorted to brightfield imaging. Interestingly, Laminin^+L^ and Coll.IV^+L^ organoids were comparable to exECM^−^ organoids, presenting a smooth surface with outer brightening, and lacking the budding seen in MG^+^ organoids (Fig 2K and Appendix Fig S6B). To assess the interaction of mouse Laminin with organoid cells, as well as the endogenous production of ECM, we stained organoids with Ms‐LAMA1 and Ms/h‐LAMA1 and Ms/h‐Perlecan antibodies. A thin Ms‐LAMA1 coating was visible in Laminin^+L^ organoids (Fig 2K and Appendix Fig S6C), indicating that exogenously supplied mouse Laminin accumulates at the organoid surface and interacts with organoid cells. However, ECM proteins of human origin presented a speckled pattern around neural rosettes in Laminin^+L^ and Coll.IV^+L^ organoids, analogous to exECM^−^ conditions (Fig 2K and Appendix Fig S6C). Thus, single ECM components were not able to replicate the effects of Matrigel on neuroepithelial budding, following instead a morphological development that closely resembled exECM^−^ cultures.

### Matrigel exposure upregulates transcriptional pathways of eye development

**Figure 3:**
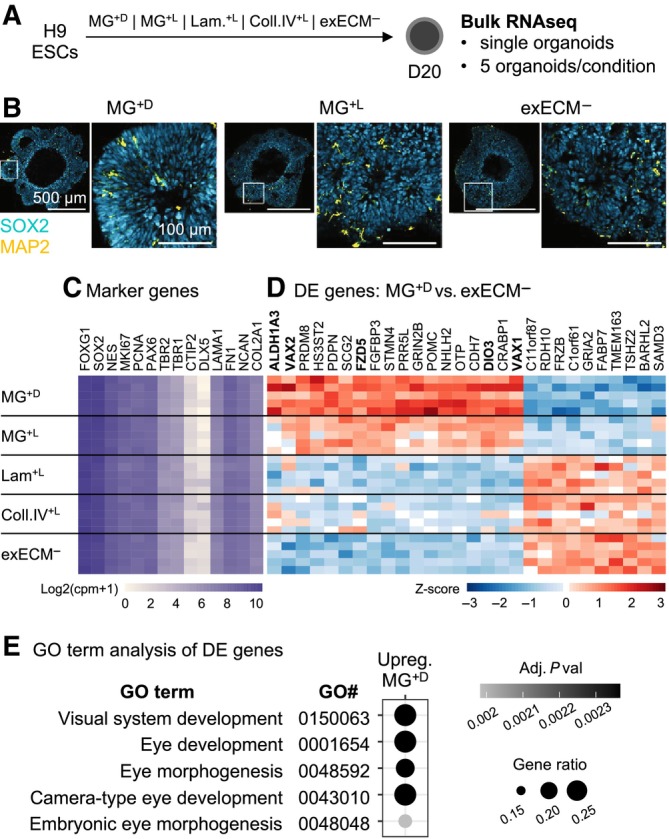
Matrigel causes the upregulation of eye development pathways in D20 organoids Experimental paradigm used for bulkRNAseq analysis. A common pool of H9‐derived organoids was divided and exposed to the five different experimental conditions. At D20, five organoids per condition were used for RNA preparation and sequencing.Immunostaining for SOX2 and MAP2, showing that organoids are predominantly composed of SOX2^+^ neural progenitors at D20. For representative images of iPSCs#1‐3‐derived organoids, see Appendix Fig S8.Expression level (in log2 copies per million, cpm + 1) of known marker genes.Normalized (by variance stabilizing transformation, vst) and row‐scaled expression levels of genes found differentially expressed between MG^+D^ and exECM^−^ organoids (adjusted *P* value below 0.05 and fold change above 1.5).GO term enrichment analysis of genes found upregulated in MG^+D^ conditions, compared to exECM^−^. Enrichment in these terms is driven by the genes highlighted in bold in panel (D). Source data are available online for this figure.

To assess how exogenous ECM signaling affects early organoid patterning and gene expression profiles, we performed bulk RNA sequencing at D20, from single H9‐derived organoids of all experimental conditions (Fig 3A and Appendix Fig S7A). At this stage, organoids were almost exclusively composed of neural progenitor cells (SOX2^+^) and the first neurons (MAP2^+^) started to be differentiated (Fig 3B and Appendix Fig S8). To assess tissue identity, we verified the expression of common marker genes (Fig 3C). Genes marking the telencephalon (*FOXG1*), neural progenitors (*SOX2*, *NES*), cycling NPCs (*MKI67*, *PCNA*), dorsal telencephalic progenitors (*PAX6*), intermediate progenitors (*TBR2*), and early‐born excitatory neurons (*TBR1*, *CTIP2*) were equally expressed across conditions. Markers of the ventral telencephalon (*DLX5*) were low or absent. Several ECM components known to be expressed during human brain development (*LAMA1*, *FN1*, *NCAN*, *COL2A1*; Amin & Borrell, 2020) showed comparable levels across conditions. To identify putative differences, we performed differential gene expression analysis (Fig 3D and Appendix Fig S7B). Only 28 genes were found differentially expressed (DE) between MG^+D^ and exECM^−^ conditions (Fig 3D) and 38 genes considering all pairwise comparisons (Appendix Fig S7B). The two clusters of DE genes confirmed the transcriptional similarity between exECM^−^, Laminin^+L^, and Coll.IV^+L^ organoids and between MG^+D^ and MG^+L^ organoids. To assess the cell processes associated with DE genes, we performed gene ontology enrichment analysis. The genes downregulated in MG^+^ conditions did not yield any GO term enrichment. The genes upregulated in MG^+^ conditions were associated with eye development and morphogenesis (Fig 3E), and this signature was stronger in MG^+D^ than in MG^+L^ organoids (Fig 3D and Appendix Fig S7B). Notably, Frizzled‐5 (*FZD5*) and fibroblast growth factor‐binding protein 3 (*FGFBP3*) genes were upregulated in MG^+^ organoids (Fig 3D and Appendix Fig S7B), suggesting that Wnt and FGF ligands present in Matrigel may influence tissue patterning. Laminin or Collagen IV did not have the same effect, indicating that signaling cues introduced by Matrigel are absent from these purified preparations, which likely act as inert matrices. Overall, D20 organoids showed comparable transcription of markers indicative of telencephalic identity, cell‐type composition, NPC proliferation, and ECM production, independent of exogenous ECM exposure. Based on the few DE genes found, Matrigel activated eye development pathways, particularly when provided as a pure droplet.

### Matrigel affects rosette size and the level of tissue mis‐patterning at early neurogenic stages

**Figure 4:**
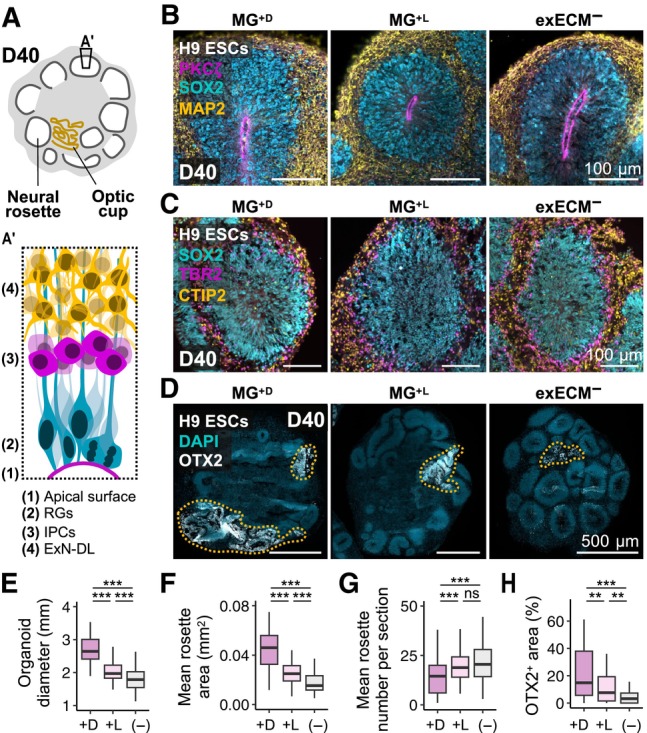
At D40, the organoid tissue is mostly comprised of dorsal‐cortical neural rosettes and minor regions of mis‐patterned cells A, A′Schematic representation of tissue architecture at D40. Abundant neural rosettes and smaller regions of optic cup tissue are present. (A′) Schematic representation of a dorsal‐cortical rosette organization.BOrganoids from all conditions show neural rosettes with PKCζ^+^ ventricular zone, and inside‐out organization of SOX2^+^ neural progenitors and MAP2^+^ neurons. For whole‐organoid view and representative images of iPSCs#1‐3‐derived organoids, see Appendix Fig S10.CNeural rosettes have dorsal‐cortical identity and radial glia (RGs, SOX2^+^), dorsal intermediate progenitors (IPCs, TBR2^+^), and early‐born deep‐layer excitatory neurons (ExN‐DL, CTIP2^+^) show layered arrangement. For whole‐organoid view and representative images of iPSCs#1‐2‐derived organoids, see Appendix Fig S11.DNontelencephalic tissue shows convoluted and disorganized morphology and is marked by OTX2 (and TTR—see Appendix Fig S12B; demarcated by yellow dashes).E–HOrganoid diameter (254 organoids) (E), mean rosette area (F), mean rosette number per cross‐section (G) (190 organoids and 7,927 rosettes), and percentage of OTX2^+^ area (190 organoids) (H) across experimental conditions at D40 (see results for individual organoids of all cell lines in Appendix Fig S13). Boxplots mark the median value; the two hinges correspond to the first and third quartiles (the 25^th^ and 75^th^ percentiles); and the whiskers extend from the hinge to the highest/lowest value no further than 1.5*IQR from the hinge (where IQR is the interquartile range, or distance between the first and third quartiles). Statistical tests are analysis of variance (ANOVA); 0 ≤ *P* < 0.001, ***; 0.001 ≤ *P* < 0.01, **; *P* ≥ 0.05, ns (see results of statistical tests in Appendix Table S1). Source data are available online for this figure.

Given the morphological and transcriptional similarity between exECM^−^, Laminin^+L^, and Coll.IV^+L^ organoids, further experiments compared only exECM^−^, MG^+L^, and MG^+D^ conditions. To assess if the initial differences in neuroepithelial morphogenesis affected early neurogenic stages, we evaluated the tissue architecture at D40. At this stage, prominent neural rosettes were visible in all conditions with brightfield imaging (Fig 1B and Appendix Fig S1A, tissue architecture schematized in Fig 4A). To assess the presence and distribution of endogenous and exogenous ECM, we resorted to immunostaining of Ms‐LAMA1 and Ms/h‐LAMA1. Ms‐LAMA1 staining showed that most MG^+D^ organoids remained encapsulated in a Matrigel droplet while remnants of Matrigel were still visible within MG^+L^ organoids (Appendix Fig S9A). The production of endogenous ECM was also sustained, as seen by abundant Ms/h‐LAMA1 expression (Appendix Fig S9B). Although all rosettes in exECM^−^ organoids were surrounded by endogenously produced LAMA1, some rosettes in MG^+^ organoids were still encapsulated by Matrigel‐derived ECM (Appendix Fig S9B). Thus, there was continued presence of Matrigel in the tissue, even 30 days after exposure.

To assess cellular organization, we used immunostaining of neural progenitor and neuronal markers. In all conditions, SOX2^+^ neural progenitors were radially arranged around large lumina and surrounded by abundant MAP2^+^ neurons (Fig 4B and Appendix Fig S10). Also, rosettes presented a dorsal‐cortical identity and a stereotypical inside‐out organization of radial glia (SOX2^+^), intermediate progenitor cells (TBR2^+^), and early‐born excitatory neurons (CTIP2^+^; Fig 4C and Appendix Fig S11, schematized in Fig 4A′). Small clusters of DLX2^+^ interneuron progenitors were only occasionally seen (Appendix Fig S12A). Thus, as verified at D20, organoids presented a predominantly dorsal telencephalic fate. To further quantify these morphological features, we measured the diameter of over 250 organoids (Appendix Fig S13A) and quantified the area and number of around 8,000 rosettes from 190 organoids (Appendix Fig S13B and C). This analysis revealed that MG^+D^ organoids were significantly larger (Fig 4E and Appendix Fig S13A) and presented a higher rosette area than exECM^−^ (Fig 4F and Appendix Fig S13B), whereas the differences between MG^+L^ and exECM^−^ were less or not significant (Appendix Fig S13A and B). On the other hand, exECM^−^ organoids presented a higher number of rosettes than MG^+D^ organoids (Fig 4G and Appendix Fig S13C). Thus, during the production of deep‐layer excitatory neurons, general features of rosette identity and spatial organization were largely independent of early Matrigel exposure. Matrigel droplet embedding caused an expansion of tissue and rosette size, balanced by a lower number of rosettes in comparison to exECM^−^ organoids.

Tissue mis‐patterning due to protocol variability or unknown exogenous cues can cause the presence of unwanted regional fates within organoids. While examining organoid morphology at D40, we identified regions that did not organize in neural rosettes, appearing more convoluted and disordered (Fig 4D, schematized in Fig 4A). The co‐expression of OTX2 and TTR in such areas indicated optic‐cup identity (Fig 4D and Appendix Fig S12B). By quantifying the percentage of OTX2^+^ tissue area per organoid in 190 organoids, we found that the percentage of OTX2^+^ regions was significantly higher in MG^+^ than in exECM^−^ organoids, with the most prominent expansion in MG^+D^ conditions (Fig 4H and Appendix Fig S13D). These results are in agreement with the patterning assessment performed with bulk RNA sequencing at D20, which suggested that Matrigel promotes the upregulation of signaling pathways of eye development and morphogenesis. Therefore, while organoids cultured in the absence of exogenous ECM were more homogenous, Matrigel potentiated an increased differentiation of non‐telencephalic tissue.

### Long‐term cell‐fate acquisition is independent of early Matrigel exposure

**Figure 5:**
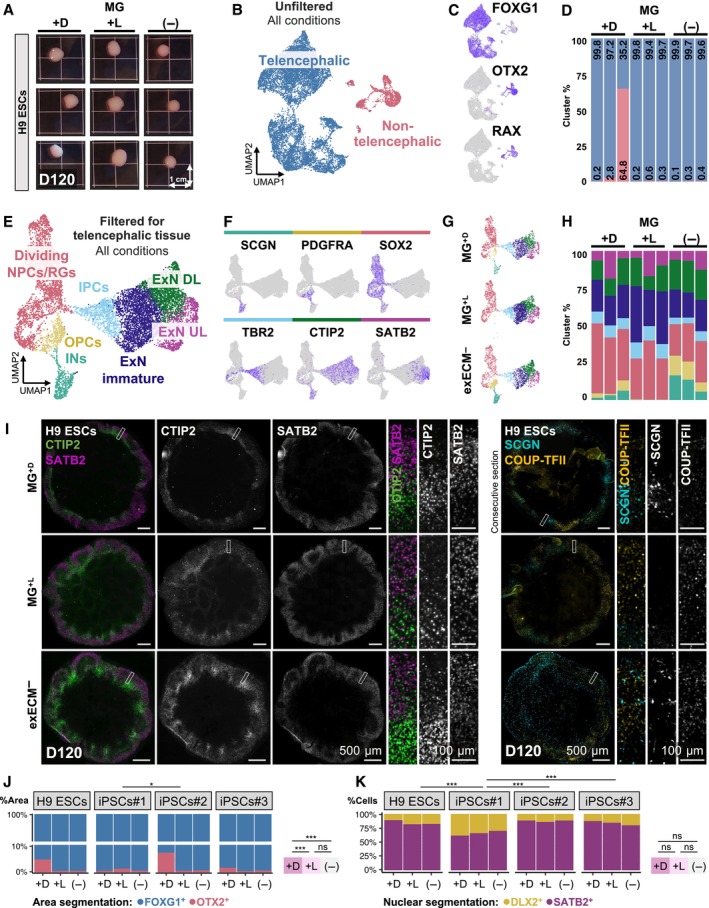
Long‐term organoid culture produces comparable cell types across conditions ANine H9‐derived organoids used for scRNAseq analysis at D120.B, CUMAP projection of cells isolated from each organoid identifies two main clusters: FOXG1^+^ telencephalic cells; and OTX2^+^RAX^+^ non‐telencephalic cells (all conditions shown).DNon‐telencephalic cells derive mainly from one MG^+D^ organoid, making up over 60% of the tissue.E, F(E) UMAP projection after filtering and exclusion of non‐telencephalic clusters allows the identification of 8 clusters, including dividing NPCs/radial glia progenitors (RGs), oligodendrocyte precursor cells (OPCs), interneurons (INs), intermediate progenitor cells (IPCs), immature excitatory neurons (ExN), deep‐layer excitatory neurons (ExNs DL), and upper‐layer ExNs (ExNs UL), marked by cell‐type specific marker genes (F) (all conditions shown).GUMAP projection of telencephalic cells, split by condition.HPercentage of telencephalic cells per cluster and per organoid.ITissue immunostaining of organoids at D120 shows abundant deep‐ and upper‐layer neurons (CTIP2^+^ and SATB2^+^, respectively) with rudimentary layer organization, as well as less abundant populations of interneurons (SCGN^+^ and COUP‐TFII^+^).J, KQuantification of the ratio of telencephalic/non‐telencephalic (FOGX1^+^/OTX2^+^) (J) and dorsal/ventral (SATB2^+^/DLX2^+^) (K) tissue in 98 organoids at D120 (see results for individual organoids of all cell lines in Appendix Fig S16). Statistical tests are analysis of variance (ANOVA); 0 ≤ *P* < 0.001, ***; 0.01 ≤ *P* < 0.05, *; *P* ≥ 0.05, ns (see results of statistical tests in Appendix Table S1). Source data are available online for this figure.

One of the main goals of *in vitro* modeling is to gain access to mature features of developing neuronal tissue. Thus, to evaluate potential long‐term effects of differential early exposure to exogenous ECM on organoid maturation, we investigated the cellular composition of organoids at a developmental stage when different classes of mature neurons were present (D120). Remarkably, although the organoid tissue had by this time completely outgrown the Matrigel added early on, large solid formations remained visible in MG^+D^ organoids, or as small remnants in MG^+L^ organoids (Appendix Fig S9C). To perform an unbiased analysis of cell‐type composition, we resorted to single‐cell RNA sequencing (scRNAseq), focusing on H9‐derived organoids and three organoids per condition (Fig 5A–H and Appendix Fig S14A). To preserve organoid origin information for each cell, we used tagging with a unique molecular identifier oligonucleotide and computational demultiplexing after sequencing; most recovered cells had unique barcodes, yielding 14.5 k high quality cells used for further analysis (Materials and Methods, Appendix Fig S14A–C). Although exECM^−^ organoids were smaller than MG^+L^ and MG^+D^ organoids (Fig 5A), no other relevant morphological distinctions were found.

Unsupervised clustering in UMAP projection identified two main clusters (Fig 5B). Differential gene expression analysis revealed that the most abundant cluster comprised FOXG1^+^ telencephalic cells, whereas the second cluster was positive for non‐telencephalic markers, including OTX2 and RAX (Fig 5C), and originated mostly from a single MG^+D^ organoid (Fig 5D). Thus, the second cluster represented a mis‐differentiation to optic cup that was already predominant in MG^+D^ organoids at D20 and D40 (Figs 3E, and 4D and H). After filtering out low‐quality cells and removing non‐telencephalic clusters, we separated clusters of dividing NPCs and radial glia progenitors (RGs), oligodendrocyte precursor cells (OPCs), interneurons (INs), intermediate progenitor cells (IPCs), immature excitatory neurons (ExNs), deep‐layer ExNs (ExNs DL), and upper‐layer ExNs (ExNs UL; Materials and Methods and Fig 5E). Each cluster was marked by the expression of cell‐type specific marker genes (Fig 5F and Appendix Fig S14D). Calculation of the percentage of cells per cluster indicated slight variations in the abundance of ventral progenitors and interneurons (Fig 5G and H). However, the proportions of cell types along the excitatory lineage (excluding OPCs and INs) were comparable between conditions (Appendix Fig S14E–G). These data suggested that slight patterning differences may be introduced by Matrigel exposure and sustained after long‐term culture.

To validate the cell‐type composition in all cell lines and conditions, we resorted to immunostaining at D120 (Fig 5I, and Appendix Fig S15A and B). The organoids were composed mostly of deep‐ and upper‐layer ExNs (CTIP2^+^ and SATB2^+^, respectively) with rudimentary layer organization. Interneurons (SCGN^+^ and COUPTFII^+^, indicating caudal ganglionic eminence origin) were found intermingled with ExNs in all conditions and cell lines (Fig 5I and Appendix Fig S15B). To quantify potential patterning differences, we measured the ratio between telencephalic/non‐telencephalic and dorsal/ventral tissue in around 100 organoids of all cell lines (Fig 5J and K, and Appendix Fig S16). The proportion of area positive for FOXG1 (telencephalon) and OTX2 (non‐telencephalon; Appendix Fig S16A and B) showed that most of the tissue was FOXG1^+^ for all cell lines and batches (Fig 5J and Appendix Fig S16E and F). However, MG^+D^ conditions contributed to an expansion of OTX2^+^ non‐telencephalic regions, most prominently in H9‐ and iPSCs#2‐derived organoids (Fig 5J and Appendix Fig S16E and F). The increased mis‐patterning was mostly driven by exposure to Matrigel and not cell line dependent (Fig 5J). Because interneurons are often seen intermingled with excitatory neurons, it is not always possible to delineate dorsal and ventral areas within an organoid. Therefore, the nuclear markers SATB2 and DLX2 were used as proxies for dorsal and ventral cells (Appendix Fig S16C), which could be segmented and counted (Appendix Fig S16D). Interestingly, this analysis revealed that the dorsal/ventral patterning was independent of Matrigel exposure (Fig 5K, and Appendix Fig S16E). Instead, there was a cell line dependency, whereby some cell lines showed an intrinsic tendency to produce more interneurons, as seen for iPSCs#1 (Fig 5K and Appendix Fig S16E). These findings corroborated the patterning analyses done at D20 (Fig 3C) and D40 (Fig 4B and C, and Appendix Figs S10 and S11) and the indications from the scRNAseq data at D120 (Fig 5B–H). Thus, aside the expansion of optic cup tissue promoted by Matrigel, global telencephalic patterning and cell‐type composition were highly similar across experimental conditions after long‐term organoid culture.

## Discussion

Epithelial morphogenesis involves the consecutive coordination of several processes, including tissue polarization by cell‐ECM and cell–cell interactions, and lumen formation, maintenance, and expansion (Martín‐Belmonte & Mostov, 2008; Datta *et al*, 2011). Given its central role in defining tissue morphology, a critical component of epithelial systems is the source of ECM.

### Tissue morphogenesis can be endogenously or exogenously driven

Here, we characterized how the presence or absence of exogenous ECM affects the development of human telencephalic organoids. We found that, in the absence of exECM, early spots of cavitation initiate tissue asymmetry and are likely the starting points of rosette lumina. These observations suggest a mechanism resembling secondary neurulation *in vivo*, which has also been reported in 2D neural progenitors (Hříbková *et al*, 2018; Fedorova *et al*, 2019) and in other *in vitro* epithelial systems (Yu *et al*, 2005; Martín‐Belmonte *et al*, 2008). Furthermore, exECM^−^ organoids show abundant production of ECM proteins—such as Fibronectin, Laminin, Perlecan, Lumican, Neurocan, and Collagens—that self‐assemble along an apical‐basal polarity axis at pre‐neurogenic stages. This shows that organoid NPCs generate ECM proteins with relevance during *in vivo* neurodevelopment (Camp *et al*, 2015; Amin & Borrell, 2020). In the human brain, NPCs have been postulated to contribute to basal deposition of ECM constituents via vesicular transport in their basal processes (Fietz *et al*, 2012), thereby contributing to tissue polarization. We propose that an analogous self‐sustained process may take place during *in vitro* development, contributing to the establishment and maintenance of apical‐basal polarity in the absence of exogenous instructive signals.

Although polarization and lumen formation emerge spontaneously *in vitro*, both processes can be influenced by culture conditions. Here, we demonstrate that Matrigel has a strong effect on tissue polarization, both when applied as a solid droplet or transiently dissolved in the culture medium. These observations contrast with other organoid systems in which both Matrigel presence and jellification (Plachot *et al*, 2009; Inman & Bissell, 2010; Kakni *et al*, 2022), as well as continuity of exposure (Co *et al*, 2019, 2021; Krüger *et al*, 2020; Li *et al*, 2020; Salahudeen *et al*, 2020; Nash *et al*, 2021; Stroulios *et al*, 2021) are needed to establish and maintain an apical‐in/basal‐out polarity. In telencephalic organoids, the action of Matrigel is likely twofold: (i) introduction of a strong basement membrane signal at the organoid surface, seen to persist for many days after initial exposure, in both MG^+D^ and MG^+L^ conditions; and (ii) signal amplification by recruitment and polymerization of endogenously produced ECM. In fact, endogenous ECM within the organoid tissue is widespread in exECM^−^ organoids and sparser in MG^+^ organoids; these differences may be due to recruitment and assembly of endogenously produced ECM at the organoid surface in the presence of Matrigel. Laminin may play a role in these processes, as it constitutes around 60% of Matrigel (Corning Incorporated Life Sciences, 2016), and has been shown to form the initial cell‐anchored polymer needed for subsequent ECM assembly, and to nucleate the polymerization of other ECM proteins (Cheng *et al*, 1997; Li *et al*, 2002, 2003). Thus, slow assembly of endogenous ECM is overtaken by a mass action of exogenous ECM upon Matrigel exposure, leading to a quick polarization process that likely occurs through a different molecular mechanism than that seen in exECM^−^ organoids. Overall, Matrigel exposure leads to the formation of neural rosettes with larger lumina, impacting the organization of neural progenitor cells early on. Due to this effect, Matrigel supplementation can be experimentally advantageous to assess NPC arrangement and morphology with short experimental timelines, as has been applied in evolutionary (Benito‐Kwiecinski *et al*, 2021) and disease modeling studies (Krenn *et al*, 2021).

Remarkably, when exposed to purified ECM components, Laminin or Collagen IV, organoids presented a morphology and transcriptional programs analogous to exECM^−^ conditions. Several aspects may contribute to this result. The mix of ECMs present in Matrigel more closely resembles an *in vivo* environment, where ECMs are present in combination. Also, due to its composition, Matrigel undergoes gelation at temperatures of 22–37°C, such that entactin cross‐links Laminin and Collagen IV, creating a hydrogel (Aisenbrey & Murphy, 2020). These physical properties, which promote the stickiness and jellification of Matrigel on the organoid tissue, produce an efficient stimulation of basal signaling, whereby several ECM proteins polymerize simultaneously and at a high concentration at the organoid surface. Additionally, in contrast to Laminin or Collagen IV alone, Matrigel led to the upregulation of Wnt and FGF receptors. This indicates that there may be growth factors in Matrigel that further contribute to its action. Overall, our results show that the complex composition and biophysical and/or biochemical properties of Matrigel could not be replaced by dissolution of pure ECM proteins in the culture medium, in the model system here characterized.

The impact of endogenous and exogenous ECM sources on epithelial morphogenesis has been addressed in other *in vitro* systems. An example are experimental paradigms that show the formation of somites, for which either a low concentration of Matrigel in the culture medium (van den Brink *et al*, 2020; Veenvliet *et al*, 2020; Sanaki‐Matsumiya *et al*, 2022) or differentiation of ECM‐producing cells within the tissue are required (Amadei *et al*, 2022; Bao *et al*, 2022; Lau *et al*, 2022). Similarly, breast luminal epithelial cells can be polarized *in vitro* by exogenous addition of laminin‐1, or co‐culture with ECM‐producing myoepithelial cells (Gudjonsson *et al*, 2002). Our findings indicate that although polarization and lumen formation of neuroepithelial cells can be rapidly potentiated by Matrigel exposure, the presence of ECM‐producing tissue‐resident cells is enough to intrinsically drive self‐organization. Ultimately, Matrigel addition and ECM‐producing cells offer alternative paths for reaching comparable morphogenic outcomes.

### Matrigel increases tissue mis‐patterning but does not affect cell‐fate acquisition

To assess tissue patterning and cell type composition, we extensively characterized organoids from D20 to D120 of development. We observed that biases in tissue patterning that increase the likelihood of optic cup tissue expansion in MG^+D^ organoids are already significantly higher at D20 and persist throughout time. These findings are in agreement with pioneering studies on *in vitro* differentiation of the optic cup, where the retinal epithelium was shown to expand in the presence of Matrigel, but not in its absence (Eiraku *et al*, 2011). Importantly, although MG^+L^ conditions efficiently promoted rosette formation, unwanted expansion of non‐telencephalic tissue was lower than in MG^+D^ conditions. Thus, we propose that when fast tissue polarization is experimentally required, liquid embedding of EBs may be advantageous in comparison to droplet embedding.

Apart from biases in telencephalic patterning, general features of long‐term maintenance and cellular composition of organoids are independent of early exposure to Matrigel. In all conditions and hPSC genetic backgrounds, the tissue is organized in neural rosettes with comparable dorsal‐telencephalic identity, inside‐out organization of progenitors and neurons, and apical‐basal polarity axis. NPCs give rise to, first, CTIP2^+^ deep‐layer neurons and, later, SATB2^+^ upper‐layer neurons. Transcriptional features and rudimentary layering of mature cortical neurons are acquired equally across conditions, as seen by scRNAseq and tissue staining, respectively. Finally, the proportion of dorsal/ventral telencephalic patterning is dependent on the genetic background, but independent of Matrigel. Of note, besides the present work, we have used the exECM^−^ culture paradigm to model a condition related to the maturation and axonal projection of upper‐layer neurons, using another four different cell lines, including patient‐derived iPSCs (preprint: Martins‐Costa *et al*, 2023). Thus, the Matrigel‐free protocol here described is a robust method to bypass the downsides of Matrigel exposure without affecting cell‐fate acquisition in long‐term cultures.

### Limitations of the study

Here, we explored the effect of exposure of telencephalic brain organoids to exogenous ECM preparations, Matrigel, Laminin, and Collagen IV, at the stage of neuroepithelium formation. We provide a detailed morphological analysis of the organoid tissue at early stages, mainly focusing on apical‐basal polarity and ECM composition, but the exact molecular mechanisms of rosette assembly were not explored. In the future, it will be interesting to perform a more detailed biochemical characterization of cavitation and polarization processes, coupled with live imaging of mosaic reporter PSC‐derived organoids at early stages, to visualize tissue morphogenesis.

Additionally, we chose to use Matrigel, it being the golden‐standard exogenous ECM in the neural organoid field. However, Matrigel contains an incompletely defined mix of ECM components and growth factors, which poses challenges in the interpretation of experimental results. For example, tissue patterning effects observed in MG^+^ organoids may be attributable to unknown growth factors introduced in the culture. Furthermore, we only tested Matrigel from one single provider and these components may be different in other preparations due to the complex production process, as previously described (Chang *et al*, 2022). Although we assessed the effect of pure Laminin and Collagen IV on organoid development, we were unable to precisely pinpoint the component or components responsible for the observed effects in Matrigel‐exposed cultures. In these experiments, Laminin^+L^ conditions led to the formation of a thin layer of mouse Laminin at the organoid surface; and the direct interaction of dissolved Collagen IV with organoid cells could not be confirmed due to lack of a mouse Collagen‐specific antibody. Furthermore, we limited these analyses to pure ECM proteins. Recent efforts to replace Matrigel make use of synthetic hydrogels, such as polymer matrices functionalized with ECM‐derived cell adhesion peptide motifs and mixed with cross‐linkers; properties like hydrogel stiffness, concentration and type of ECM proteins, and cross‐linker density can be fine‐tuned to achieve biological responses of interest (Kozlowski *et al*, 2021). It is possible that such synthetic hydrogels would produce very different effects on neuroepithelial morphogenesis, but these experiments were out of the scope of this study.

Finally, we focused on the action of exogenous ECM at specific stages of neuroepithelium formation and morphogenesis. Our data reveal differences in organoid size and in the number and size of neural rosettes. While we could not detect any consequences of these early differences in cell‐fate acquisition throughout organoid development, we did not explore network formation or electrophysiological properties of neuronal populations. Work from other groups has demonstrated that Matrigel addition at later timepoints or throughout long‐term organoid culture can improve cortical plate formation (Kadoshima *et al*, 2013; Lancaster *et al*, 2017; Velasco *et al*, 2019; Bhaduri *et al*, 2020). By focusing on Matrigel addition at early stages of organoid development, we could not evaluate this effect or others described in the many different organoid protocols previously developed.

### Impact and future directions

Supplementation of organoid cultures with exogenous ECM is widespread, but a full characterization of its necessity and impact is missing for certain *in vitro* systems, including neural organoids. Our findings follow recent attempts to tackle the variable and undefined composition of Matrigel by developing synthetic alternatives and Matrigel‐free culturing methods (Kratochvil *et al*, 2019; Aisenbrey & Murphy, 2020; Kozlowski *et al*, 2021; Nayler *et al*, 2021; Roth *et al*, 2021; Kim *et al*, 2022). Our data strongly support an experimental model in which early Matrigel exposure is useful to trigger quick morphogenic changes, when experimentally needed, but not necessary for fate‐acquisition during long‐term development of human telencephalic organoids. Key findings are supported by a comparable study carried out in cerebellar organoids (Nayler *et al*, 2021), showing how they may be transversal to neuroectoderm‐derived tissues and applicable to *in vitro* models of other brain regions. These findings generate important knowledge regarding neuroepithelial biology *in vitro* and the self‐sustainability of tissue morphogenesis without the need for extrinsic guiding factors. Ultimately, adding to previous efforts from other groups, we believe that this study answers long‐standing questions in the field and is a promising step toward fully characterizing and unifying neural organoid research.

## Materials and Methods

### hPSC maintenance and passaging

Feeder‐free hESC line WA09 (H9 ESCs) were commercially obtained from WiCell. iPSCs#1 and iPSCs#2 were reprogrammed in‐house from fibroblasts of healthy donors (internal nomenclature: iPSCs 178/4 and iPSCs 176/1, respectively). iPSCs#1 and iPSCs#2 cell lines can be obtained from the IMBA iPSC Biobank (https://www.oeaw.ac.at/imba/scientific-facilities/stem-cell-core-facility) after ethical approval. iPSCs#3 were Rozh‐5 iPSCs commercially obtained from HipSci. Therefore, we used a very common ESC line, as well as commercially available and in‐house reprogrammed iPSC lines. All cells were cultured on six well‐plates (Corning, 3516) coated with hESCs‐qualified Matrigel (Corning, 354277) and maintained in complete mTeSR1 medium (StemCell Technologies, 85875). Cells were fed daily with 2 ml of mTeSR1 (feeding with 4 ml allowed skipping of one feeding day per week) and passaged after reaching 60–80% confluency (every 3–5 days). For passaging, cells were exposed to 0.5 mM EDTA diluted in PBS (pH 7.4, without MgCl_2_ or CaCl_2_; Gibco, 14190‐250) during 3 min at 37°C, lifted in mTeSR1 by gentle spraying of the bottom of the well, and triturated to small clusters of 20–50 cells. Cells were routinely tested for mycoplasma, and verified to display a normal karyotype resorting to short tandem repeat (STR) analysis and intact genomic integrity resorting to single‐nucleotide polymorphism (SNP) analysis. Cells, EBs, and organoids were kept in a 5% CO_2_ incubator at 37°C.

### Dorsal tissue‐enriched telencephalic organoid generation

Media formulations are found in Appendix Table S2. All media were vacuum‐filtered through a membrane with pore size of 0.22 μm. Feeding volumes in 96WP format were of 150 μl and in 10 cm dish format of 15 ml.

#### Embryoid body formation

Dorsal forebrain‐enriched telencephalic organoids were generated as previously described with slight modifications (Esk *et al*, 2020). hPSCs were grown to 60–80% confluency and dissociated into a single cell suspension by Accutase (Sigma‐Aldrich, A6964) treatment for 5 min at 37°C, followed by manual trituration. Cells were seeded in an ultralow binding 96‐well plate (Szabo‐Scandic, COR7007), at a density of 9,000 live cells/well, in 150 μl of complete Essential 8 (E8) medium (Thermo Fisher Scientific, A1517001) with 50 μM Rho‐associated protein kinase (ROCK) inhibitor (Selleck Chemicals, S1049). On day 3, the medium was replaced with E8 without ROCK inhibitor supplementation. From day 6, EBs were fed daily with neural induction (NI, Appendix Table S2) medium.

#### Batch quality control assessment

On day 10, the batches in which over 80% of EBs formed successfully were kept for further experiments. Quality criteria included EB size above 500 μm, round morphology, and the appearance of peripheral tissue clearing, indicative of the start of neuroepithelium formation. Batches compliant with these criteria were randomly divided at D10 into three groups of different conditions of exogenous ECM (exECM) supplementation. Details of handling for each condition are described below. Of note, within successful batches, organoid quality was comparable across all conditions and, thus, likely determined by factors preceding or independent of Matrigel exposure, such as pluripotency state, confluency, and passage number of the starting population of hPSCs, as well as user technique, at the stage of EB setup.

#### Tissue patterning

On day 13, the NI medium was replaced with Differentiation Medium without vitamin A (Diff^−A^, Appendix Table S2). Two pulse applications of 3 μM of GSK‐3 Inhibitor CHIR99021 (Merck Millipore, 361571) were done on days 13 and 14; feeding was skipped on day 15; and the medium was replaced with Diff^−A^ without CHIR99021 on day 16.

#### Long‐term culture

On day 20, all organoids, regardless of Matrigel condition, were transferred to 10 cm dishes and cultured on an orbital shaker (Celltron) at a rotating speed of 57 rpm. On day 25, the medium was replaced with Differentiation Medium with vitamin A (Diff^+A^, Appendix Table S2). From day 25 onward, the medium formulation remained unaltered throughout organoid development, and the medium was changed twice a week (every 3–4 days).

### Exogenous ECM application

The following exogenous ECM preparations were used: Matrigel LDEV‐Free (Corning, 354234); Cultrex Mouse Laminin I Pathclear (R&D Systems, 3400‐010‐02); and Cultrex Mouse Collagen IV (R&D Systems, 3410‐010‐02).

#### No exogenous ECM (exECM ^−^)

At D10, EBs remained in 96‐well plates and received daily exchange of medium according to the schedule described above. Exceptions occurred during weekends, when often one day of feeding was skipped; and during the pulse application of CHIR99021, when medium exchange occurred precisely on D13 and D14 and skipped on D15.

#### Droplet embedding (MG ^+D^)

At D10, EBs were embedded into droplets of Matrigel on dimpled sheets of parafilm, as previously described (Lancaster *et al*, 2017). Only organoids that remained within Matrigel droplets were used for downstream analyses; organoids that detached from the droplet were discarded.

#### Liquid embedding (MG ^+L^, Laminin^+L^, Coll.IV ^+L^)

The culture medium (NI) was supplemented with exogenous ECM (Matrigel, Laminin or Collagen IV) at a concentration of 2%V/V at D10. Exogenous ECM was left out from the following feeding, at D13.

Until D20, MG^+D^, MG^+L^, Laminin^+L^, and Coll.IV^+L^ organoids were cultured in suspension, in a stationary 10 cm dish, with media exchange every 3 days, according to the schedule of media described above. Exceptions occurred during the pulse application of CHIR99021, when medium exchange occurred precisely on D13 and D14 and was skipped on D15. To avoid organoid attachment, 10 cm dishes were coated with anti‐adherence rinsing solution (Stemcell Technologies, 7010) during 2 min, followed by one rinse with PBS, immediately before organoid transfer.

### Cryosectioning

Organoids were collected at days 10, 13, 16, 20, 40, and 120, and fixed in 4% paraformaldehyde (PFA) for 30 min (D10–D20) or 1–2 h (D40–D120) at room temperature (RT). After three 15 min washes with PBS, organoids were immersed in a 15% sucrose (Merck Millipore, 84097)/10% gelatin (Sigma, G1890‐500G) solution at 37°C, until they sunk to the bottom of the tube (from 1 h up to overnight). Organoids were embedded in the same sucrose/gelatin solution, solidified for 30 min at 4°C, and flash frozen in a bath of 2‐methylbutane supercooled to a temperature of −50°C by dry ice. Samples were stored at −70°C until further processing. Cryoblocks were sectioned at 20 μm thickness using a cryostat (Thermo Fisher Scientific, CryoStar NX70).

### Immunohistological staining

The slides were defrosted and hydrated during 5 min in PBS. Cryo‐sections were permeabilized and blocked with blocking solution (5% bovine serum albumin (BSA; Europa Bioproducts, EQBAH‐0500) and 0.3% Triton X‐100 (Merck Millipore, 93420) in PBS) for 1–2 h at RT. Antibody incubations were done in antibody solution (1% BSA, 0.1% Triton X‐100 in PBS); after dilution of antibodies, the solution was spun at maximum speed for 2 min. First, sections were incubated with antibody solution containing 1:200 diluted primary antibodies, during 3.5 h (up to overnight) at RT. Then, after one wash of 5 min with PBS, sections were incubated with antibody solution containing 1:500 diluted secondary antibodies and 1:10,000 diluted Hoechst 33342 nuclear dye (Thermo Fisher Scientific, H3569), for 1–2 h at RT. Primary and secondary antibodies used in this study are summarized in Appendix Table S3. After one wash of 5 min with PBS, the slides were mounted with DAKO mounting medium (Agilent, S302380‐2). The slides were left at RT to dry overnight and kept at 4°C for long‐term storage.

### Image acquisition

Immunostaining images of organoids from D10 to D20 were acquired with an upright LSM 800 confocal microscope (Zeiss). Immunostaining images of D40 and D120 organoids were acquired with a Pannoramic FLASH 250 II digital Slide Scanner (3DHISTECH). Brightfield images of intact organoids in Fig 1B and Appendix Fig S1 were acquired with a widefield microscope (AxioVert.A1, Zeiss GmbH) with a SONY Chameleon®3 CM3‐U3‐31S4M CMOS camera (Zeiss GmbH). Images of whole organoids in Fig 5A and Appendix Fig S15A were acquired with a Google Pixel 6. Immunostaining panels were prepared in Inkscape.

### Imaging data analysis

Data matrices of quantifications were processed in R software v4.2.2 using dplyr (v1.0.9) and visualized using ggplot2 (v3.4.0). Boxplots mark the median value; the two hinges correspond to the first and third quartiles (the 25^th^ and 75^th^ percentiles); and the whiskers extend from the hinge to the highest/lowest value no further than 1.5*IQR from the hinge (where IQR is the inter‐quartile range, or distance between the first and third quartiles). Statistical analyses were performed in R software by analysis of variance (ANOVA). The threshold for statistical significance was *P* < 0.05. Where indicated: 0 ≤ *P* < 0.001, ***; 0.001 ≤ *P* < 0.01, **; 0.01 ≤ *P* < 0.05, *; *P* ≥ 0.05, ns.

#### Quantification of organoid diameter at D10–D20

Measurement of organoid diameter from brightfield images was performed in Fiji software using the line tool. When organoids presented noncircular (e.g. oval) morphology, the largest dimension was measured. To aid visualization of growth dynamics, a trendline was added to the plot, using a smoothed conditional means function (ggplot2::geom_smooth, formula = ‘y ~ x’).

#### Quantification of endogenous and exogenous ECM at D20

The area surrounding rosettes covered by endogenous or exogenous ECM was quantified as exemplified in Appendix Fig S4. Organoid sections were co‐stained for PKCζ, Ms‐LAMA1 (Matrigel‐derived), and Ms/h‐FN (Matrigel or endogenously derived). Neural rosettes were identified by a PKCζ^+^ lumen with radial arrangement of cell nuclei. The outside region of individual neural rosettes was segmented with a 15.6 μm‐thick band (50 pixels, “Segmented line” tool in Fiji) and straightened (“Straighten” tool in Fiji). In the Ms/h‐FN channel, the following commands were performed in Fiji: (i) definition of an intensity threshold (“Threshold” command, method: triangle) and (ii) detection of positive areas and generation of regions of interest (ROIs; “Analyze Particles” command). Using the “Multi‐measure” option, both channels were measured. The results of “Area” measurement for each ROI on each channel were exported. In R, ROI positivity was defined as percentage of positive area above 20%; the total area was calculated based on the dimensions of the straightened rosette file (50 px × rosette perimeter). Finally, the percentage of area covered by Matrigel‐derived (Ms‐LAMA1^+^Ms/h‐FN^+^) or endogenously derived (Ms‐LAMA1^−^Ms/h‐FN^+^) ECM, or by negative staining, was plotted. In Fig 2I, the mean and positive standard deviation (mean + SD) per cell line and condition is shown.

#### Quantification of organoid, rosette, and OTX2 ^+^ areas at D40

Demarcation of organoid, rosette, and OTX2^+^ areas was performed manually by drawing regions of interest (ROIs) in the CaseViewer software (3DHISTECH), using 2 or 3 slices per organoid.

#### Quantification of FOXG1 ^+^ and OTX2 ^+^ areas at D120

Demarcation of FOXG1^+^ and OTX2^+^ areas was performed manually by drawing regions of interest (ROIs) in the CaseViewer software (3DHISTECH), using 2 or 3 slices per organoid.

#### Quantification of SATB2 ^+^ and DLX2 ^+^ cells at D120

Organoid sections were stained for SATB2 and DLX2. For detecting positive nuclei, a custom workflow was designed, using “CaseViewer”, “Fiji”, and “Cellpose”. The organoid slices were scanned, marked, and exported as individual channels via the CaseViewer software. In Fiji, we performed segmentation of the outermost organoid surface, corresponding to healthy tissue, with a 325 μm thick band (1,000 pixels), using the “Segmented line” tool. To map this region to an xy‐coordinate system, reduce image size and exclude unwanted areas the “Straighten” command was used. Via the Cellpose implementation in Fiji, the positive cells in each channel were detected using the pre‐trained “Cyto” model. Positive cells were segmented, and regions of interest generated. Exemplary outputs of different steps of the protocol are depicted in Appendix Fig S16D.

### Bulk RNA sequencing

Single organoids from the same batch were used for bulk RNA sequencing analysis at D20. We used five organoids per each condition of exECM exposure (MG^+D^, MG^+L^, Laminin^+L^, Coll.IV^+L^, and exECM^−^).

#### RNA preparation

RNA was isolated from single organoids using the Qiagen RNeasy Mini kit (Qiagen, 74104), following the manufacturer's instructions.

#### Sequencing

For RNA sequencing, the Lexogen's Quantseq kit was used. Sequencing was performed in an Illumina NovaSeq flowcell (single read, 100 bp). All kits were used according to manufacturers' instructions.

#### Data analysis

RNAseq reads were trimmed using BBDuk v38.06 (ref = polyA.fa.gz, truseq.fa.gz, k = 13, ktrim = r, useshortkmers = t, mink = 5, qtrim = r, trimq = 10, minlength = 20) and reads mapping to abundant sequences included in the iGenomes UCSC hg38 reference (human rDNA, human mitochondrial chromosome, phiX174 genome, adapter) were removed using bowtie2 v2.3.4.1 alignment. The remaining reads were analyzed using genome and gene annotation for the GRCh38/hg38 assembly obtained from Homo sapiens Ensembl release 94. Reads were aligned to the genome using star v2.6.0c and reads in genes were counted with featureCounts (subread v1.6.2) using strand‐specific read counting for QuantSeq experiments (−s 1). Differential gene expression analysis on raw counts and variance‐stabilized transformation (vst) of count data for heatmap visualization were performed using DESeq2 v1.38.3; genes were considered differentially expressed when the adjusted *P* value was below 0.05 and the fold change above 1.5. Functional annotation enrichment analysis of differentially expressed genes was conducted using clusterprofiler v4.6.2; GO terms were considered when the adjusted the *P* value was below 0.01 and the gene number above 2.

### Single‐cell RNA sequencing

#### Generation of single‐cell suspension of organoid cells

Organoids used for scRNAseq were harvested, cut into 2 or 3 pieces using two P10 pipette tips, and washed with DPBS^−/−^. Each individual organoid was incubated in 1.5 ml of Trypsin (Thermo Fisher Scientific, 15400054)/Accutase (Sigma‐Aldrich, A6964) (1:1) containing 1 μl/ml of TURBO™ DNase (Thermo Fisher Scientific, AM2238, 2 U/μl) in a gentleMACS Dissociator (Miltenyi Biotec, 130‐093‐235) in the program NTDK1. Tubes with dissociated cells were briefly spun down and 1.5 ml of buffer (ice‐cold DPBS^−/−^ (Thermo Fisher Scientific) with 0.1% BSA (Sigma‐Aldrich)) was added to the dissociated cells. The cell suspension was spun at 400 *g* for 5 min at 4°C. The supernatant was aspirated, leaving a margin of around 200 μl, and cells were resuspended in 700 μl of buffer. The suspension was filtered through a 70 μm strainer once, and through a FACS tube cap twice.

#### FACS sorting of viable cells

The viability dye DraQ7 (Biostatus, DR70250, 0.3 mM) was added at a concentration of 20 μl/ml and the suspension gently mixed with a P1000 pipette. 250 k live cells of each individual organoid were FACS sorted using a 100 μm nozzle; singlets were gated based on forward and side scatter, and live cells based on negative excitation with an Alexa 700 filter.

#### Organoid multiplexing

The 10× Genomics 3′ CellPlex Kit was used to multiplex each individual organoid with a unique Cell Multiplexing Oligo (CMO), as described in the manufacturer's protocol, except for the use of the aforementioned buffer formulation and 400 *g* in the spinning steps.

#### Library preparation

After multiplexing, live cells of each organoid were counted and pooled in equal numbers, normalized to the organoid with the lowest live cell count. The final pool was spun and resuspended in a small volume, and live cells were counted again. Two libraries were prepared, having been loaded with 40 and 50 k live cells per channel (to give estimated recovery of 10 k cells per channel) onto a Chromium Single Cell 3′ B Chip (10× Genomics, PN‐1000073) and processed through the Chromium controller to generate single‐cell GEMs (Gel Beads in Emulsion). ScRNAseq libraries were prepared with the Chromium Single Cell 3′ Library & Gel Bead Kit v.3 (10× Genomics, PN‐1000075).

#### Library sequencing

The two libraries were pooled in a NovaSeq S4 flowcell (Illumina, together with other samples) and pair‐end sequenced.

### Single‐cell RNA data analysis

#### Data preprocessing

ScRNAseq reads were processed with Cell Ranger multi v6.0.1 (10× Genomics), using the prebuilt 10× GRCh38 reference refdata‐gex‐GRCh38‐2020‐A, and including introns. Further processing such as dimensionality reduction, clustering, and visualization of the scRNAseq data was performed in R software v4.2.2 with Seurat v4.2.0. No batch effect was detected between the two libraries, therefore they were processed jointly.

#### Identification of non‐telencephalic clusters

Non‐telencephalic cells were separated at clustering resolution 0.1 (FindClusters). Non‐telencephalic clusters (clusters 3, 5 and 6) were removed for downstream analysis, based on absent/low FOXG1 expression.

#### Removal of low‐quality cells

After exclusion of non‐telencephalic cells, we used the Gruffi algorithm to identify and remove cells presenting a transcriptional signature of cellular stress (Vértesy *et al*, 2022). Cells with 800 to 6,000 detected genes, and less than 6% mitochondrial and 30% ribosomal content were retained. Count data was log‐normalized and scaled regressing out the number of genes and percentage of mitochondrial and ribossomal RNAs. Dimensionality reduction was performed using PCA on the top 2,000 most variable genes, and the first 20 PCs were selected for the subsequent analysis.

#### Identification of telencephalic clusters

Clustering at resolution 1 and 0.5 yielded the most reliable separation by cell types, based on the expression of known marker genes: dividing NPCs and RGs (clusters 3, 5, 7, 8,12 at resolution 1), OPCs (cluster 10 at resolution 1), INs (cluster 9 at resolution 1), IPCs (cluster 4 at resolution 1), immature excitatory neurons (clusters 0, 1, and 2 at resolution 1 and clusters 0 and 6 at resolution 0.5), ExNs DL (cluster 1 at resolution 0.5), and ExNs UL (clusters 6 and 11 at resolution 1).

#### Data plotting

Two‐dimensional representations were generated using uniform manifold approximation and projection (UMAP; uwot v0.1.14). Data matrices of quantifications were processed in R software v4.2.2 using dplyr (v1.0.9) and visualized using ggplot2 (v3.4.0).

## Author contributions

**Catarina Martins‐Costa:** Conceptualization; data curation; formal analysis; validation; investigation; visualization; methodology; writing – original draft; writing – review and editing. **Vincent A Pham:** Methodology. **Jaydeep Sidhaye:** Conceptualization; supervision; writing – review and editing. **Maria Novatchkova:** Methodology. **Andrea Wiegers:** Methodology. **Angela Peer:** Methodology. **Paul Möseneder:** Methodology. **Nina S Corsini:** Conceptualization; supervision; project administration; writing – review and editing. **Jürgen A Knoblich:** Conceptualization; supervision; funding acquisition; project administration; writing – review and editing.

## Disclosure and competing interests statement

JAK is inventor on a patent describing cerebral organoid technology and co‐founder and scientific advisory board member of a:head bio AG.

## Supporting information



Appendix

Source Data for Figure 1

Source Data for Figure 2

Source Data for Figure 3

Source Data for Figure 4

Source Data for Figure 5

## Data Availability

The scRNAseq and bulk RNAseq data discussed in this publication have been deposited in NCBI's Gene Expression Omnibus (Edgar *et al*, 2002) and are accessible through GEO Series accession number GSE220085 (http://www.ncbi.nlm.nih.gov/geo/query/acc.cgi?acc=GSE220085). Analyses were performed as outlined in the Materials and Methods. No custom code was generated for this study; the used code is available upon request.
