# Supplementary material for: Morphogenesis and development of human telencephalic organoids in the absence and presence of exogenous extracellular matrix
Source: EMBO J. 2023 Oct 16;42(22):e113213. doi: 10.15252/embj.2022113213 (PMC10646563; doi:10.15252/embj.2022113213)
Supplement: Supplementary file 1 — Appendix [file EMBJ-42-e113213-s004.pdf]

Appendix

Appendix Figure S1 2
Appendix Figure S2 3
Appendix Figure S3 4
Appendix Figure S4 5
Appendix Figure S5 6
Appendix Figure S6 7
Appendix Figure S7 8
Appendix Figure S8 9
Appendix Figure S9 10
Appendix Figure S10 11
Appendix Figure S11 12
Appendix Figure S12 13
Appendix Figure S13 14
Appendix Figure S14 15
Appendix Figure S15 16
Appendix Figure S16 17
Appendix Table S1 18
Appendix Table S2 21
Appendix Table S3 22

A

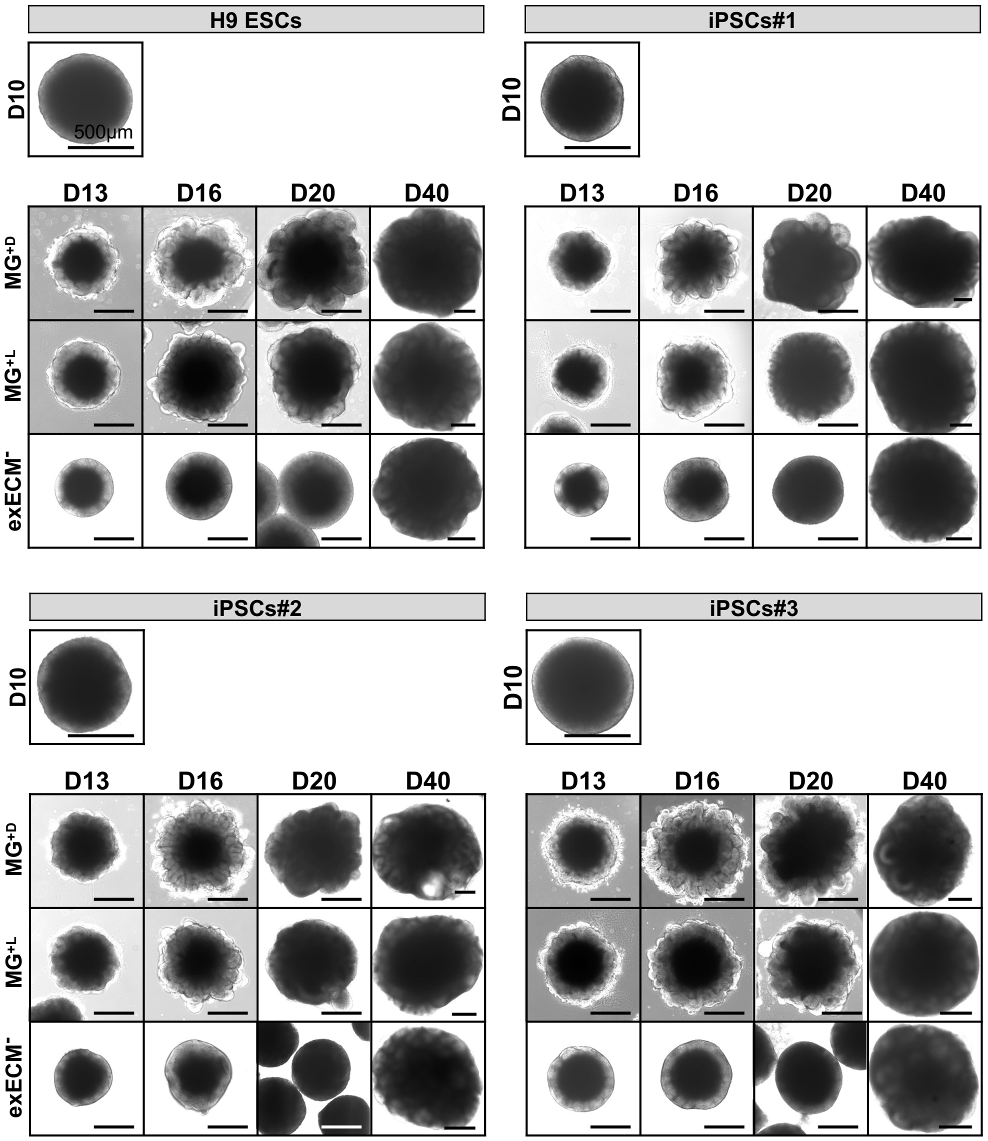

B

Imaging + size measurement		H9 ESCs			iPSCs#1			iPSCs#2			iPSCs#3			Total
Day 10	Batches	2			2			3			5			
	Organoids	12			12			17			34			**75**
		+D	+L	(-)	+D	+L	(-)	+D	+L	(-)	+D	+L	(-)	
Day 13	Batches	2	2	2	2	2	2	2	3	3	5	5	5	
	Organoids	14	40	10	13	28	13	21	43	15	37	63	26	**323**
Day 16	Batches	2	2	2	2	2	2	3	3	3	5	5	5	
	Organoids	14	38	10	13	20	14	20	30	23	39	61	34	**316**
Day 20	Batches	2	2	2	2	2	2	3	3	3	5	5	5	
	Organoids	12	28	35	8	12	15	18	23	38	38	66	55	**348**

Appendix Figure S1. (Related to Fig. 1B-C) **Organoid morphology at D10, D13, D16, D20, and D40 evolves in comparable ways in all cell lines. (A)** Morphological changes that are comparable across cell lines include outer brightening at D10, tissue budding in MG^{+D} and MG^{+L} conditions from D16, and formation of neural rosettes across conditions at D40. Scale bars: 500 µm. Images of H9 ESCs and D20 and D40 timepoints are as in **Fig.1B**. **(B)** Number of organoids (technical replicates) and batches (biological replicates) used for the measurement of organoid diameter across timepoints (**Fig. 1C**).

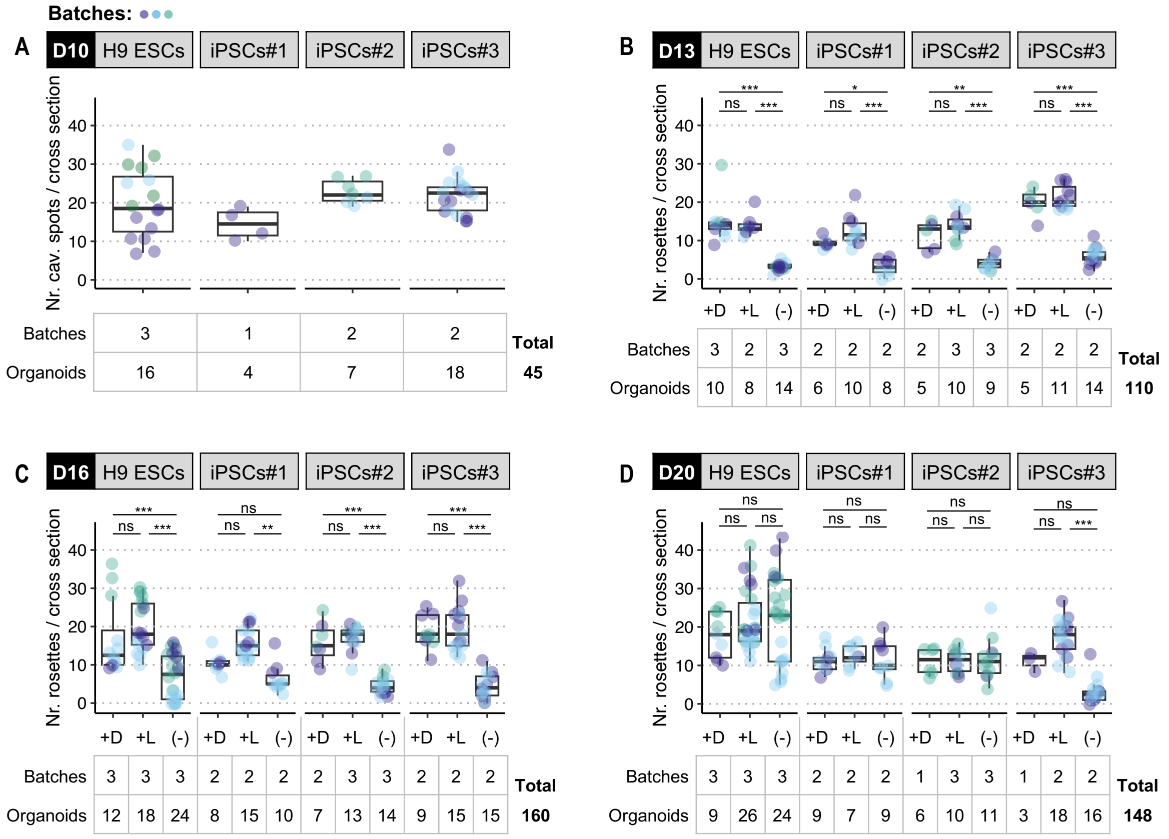

Appendix Figure S2. (Related to Fig. 2B and 2F) **Number of organoids and batches used to quantify the number of cavitation spots and neural rosettes from D10 to D20. (A)** Cavitation spots at D10. Neural rosettes at D13 **(B)**, D16 **(C)**, and D20 **(D)**. Boxplots mark the median value; the two hinges correspond to the first and third quartiles (the 25th and 75th percentiles); and the whiskers extend from the hinge to the highest/lowest value no further than 1.5 * IQR from the hinge (where IQR is the inter-quartile range, or distance between the first and third quartiles). Each datapoint is an individual organoid (technical replicate); datapoint colors indicate organoid batches (biological replicates). Statistical tests are analysis of variance (ANOVA); $0 \leq p < 0.001$, ***; $0.001 \leq p < 0.01$, **; $0.01 \leq p < 0.05$, *; $p \geq 0.05$, ns (see results of statistical tests in **Appendix Table S1**). The tables indicate the number of organoids (technical replicates) and batches (biological replicates) used in these analyses.

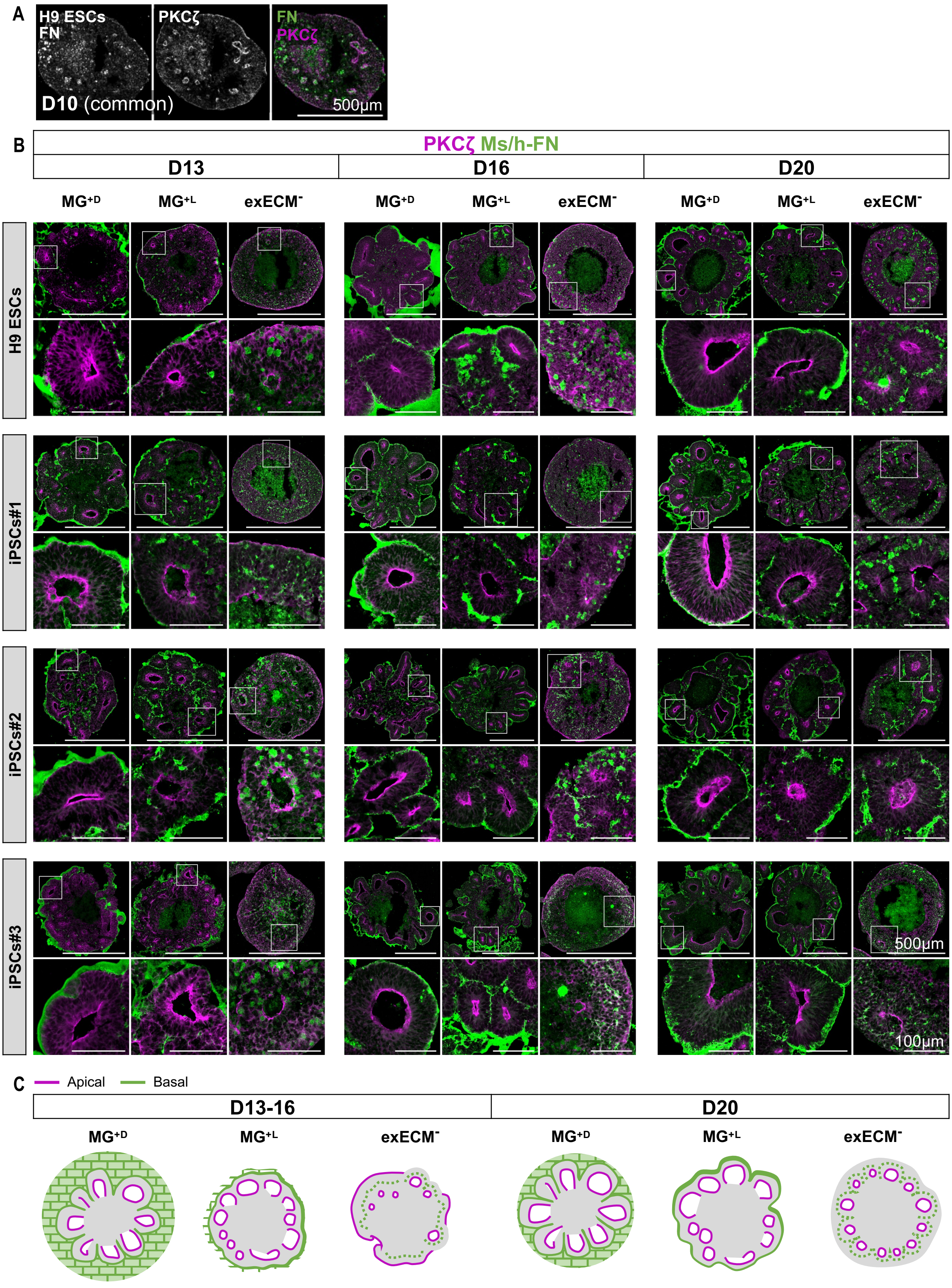

Appendix Figure S3. (Related to Fig. 2G) **Comparison of the distribution of polarity proteins at D10 (A) and D13-D20 (B) shows the quick action of Matrigel on tissue morphogenesis.** Apical and basal domains are marked by PKCζ and FN, respectively. Bottom panels: magnification of inset. Whole-organoid images of H9 ESCs at D20 are as in **Fig.2G**. **(C)** Schematic representation of tissue morphogenesis from D13 to D20. An apical-in/basal-out polarity axis is defined at D13-D16 in MG^{+D} and MG^{+L} organoids, but still undefined in most exECM^{-} organoids. At D20, an apical-in/basal-out polarity axis is defined in all conditions.

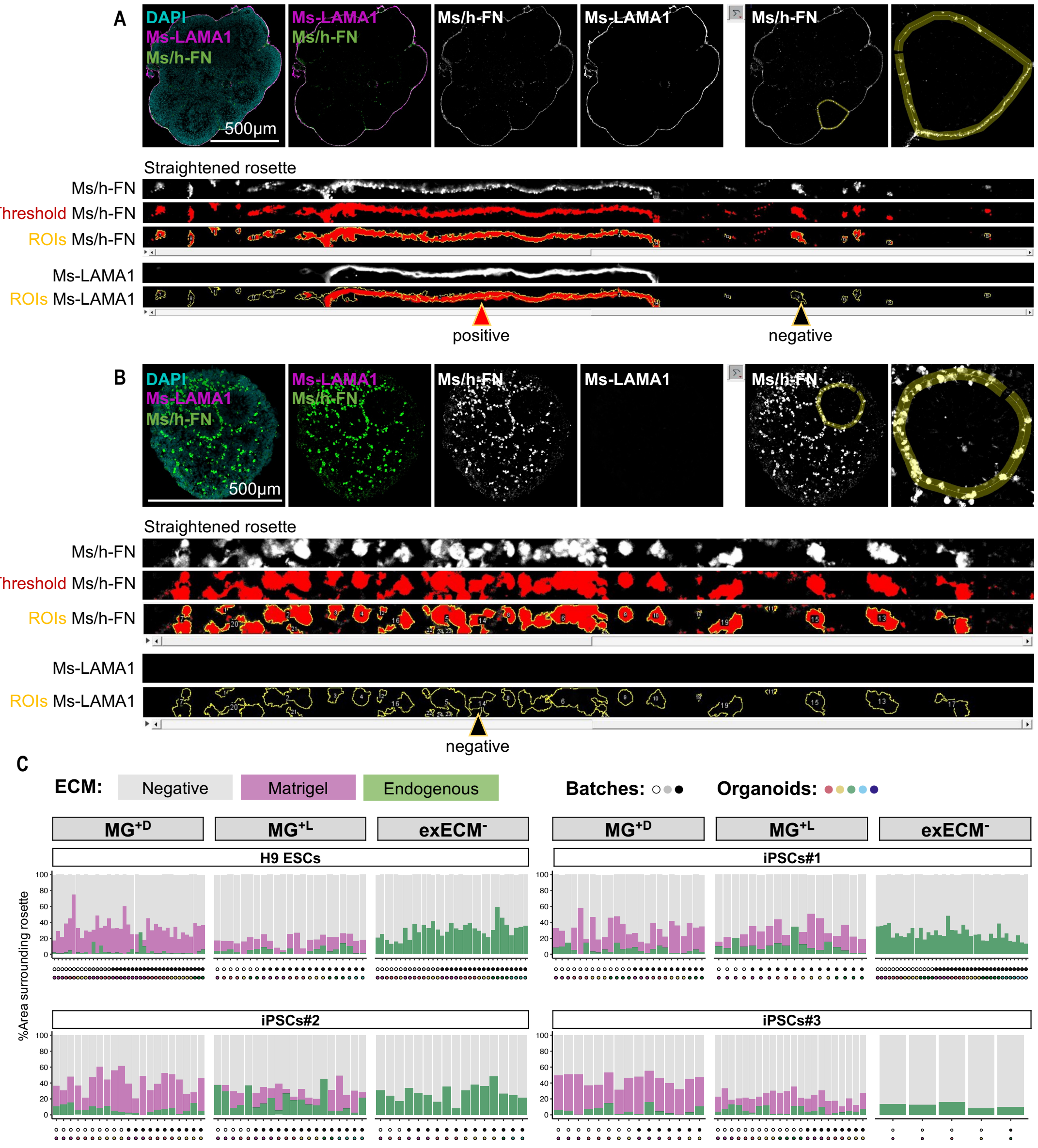

D

	H9 ESCs			iPSCs#1			iPSCs#2			iPSCs#3			
	+D	+L	(-)	+D	+L	(-)	+D	+L	(-)	+D	+L	(-)	**Total**
Batches	3	2	3	2	2	2	2	2	2	2	2	2	
Organoids	9	9	11	8	5	9	6	7	7	6	7	4	**88**
Rosettes	39	23	33	25	18	39	21	20	16	15	22	5	**276**

Appendix Figure S4. (Related to Fig. 2H-I) **Quantification of the area surrounding neural rosettes covered by endogenous or exogenous ECM. (A-B)** Co-staining of Ms-LAMA1 (Matrigel-derived) and Ms/h-FN (Matrigel or endogenously derived). Exemplary output of the method in one MG^{+D} **(A)** and one exECM^{-} organoid **(B)** (details in **Materials and Methods**). Briefly, the outside region of individual neural rosettes was segmented with a 15.6µm-thick band (50 pixels, "Segmented line" tool in Fiji) and straightened ("Straighten" tool in Fiji). In the Ms/h-FN channel, which represents both endogenous and exogenous ECM, the following commands were performed: 1) definition of an intensity threshold ("Threshold" command, method: triangle); 2) detection of positive areas and generation of regions of interest (ROIs) ("Analyse Particles" command in Fiji). In both channels, ROIs were defined as positive (red arrowheads) or negative (black arrowheads) based on the percentage of positive area. The two organoids shown here are as in **Fig.2H**. **(C)** Percentage of area covered by Matrigel-derived (Ms-LAMA1^{+}Ms/h-FN^{+}) or endogenously derived (Ms-LAMA1^{-}Ms/h-FN^{+}) ECM, or by negative staining, for each single rosette analyzed. **(D)** Number of rosettes, organoids (technical replicates), and batches (biological replicates) used for this quantification.

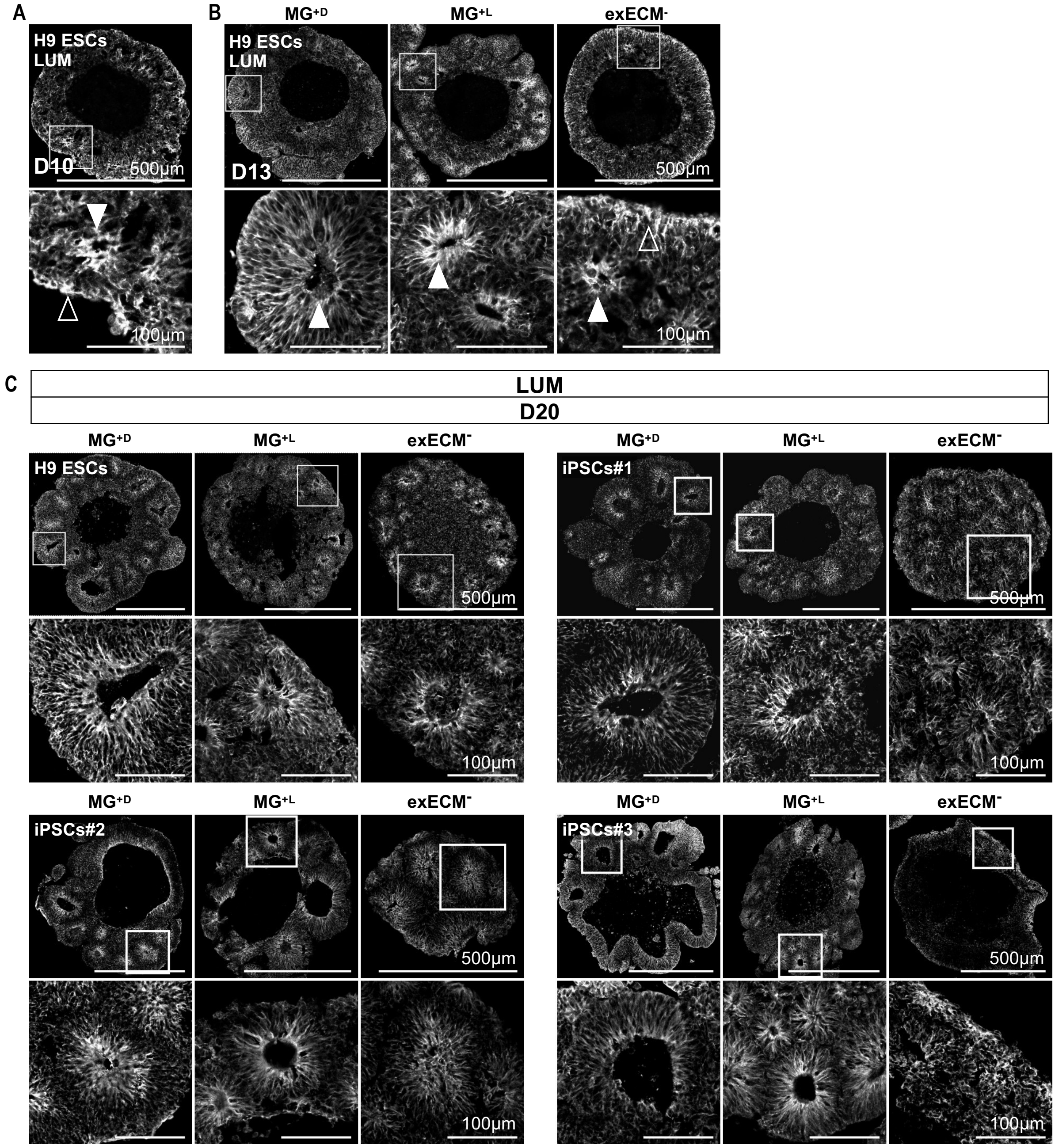

Appendix Figure S5. (Related to Fig. 2) **Lumican is endogenously produced by NPCs in all conditions. (A)** At D10, LUM is abundantly expressed but presents disorganized tissue distribution. Apical localization to rosette ventricular zones is defined at D13 in MG^{+D} and MG^{+L} organoids **(B)** and at D20 in all conditions **(C)**. Arrowheads mark the location of LUM lining the organoid outer surface (empty arrowheads) or the ventricular zone of neural rosettes within the tissue (white arrowheads). Bottom panels: magnification of inset.

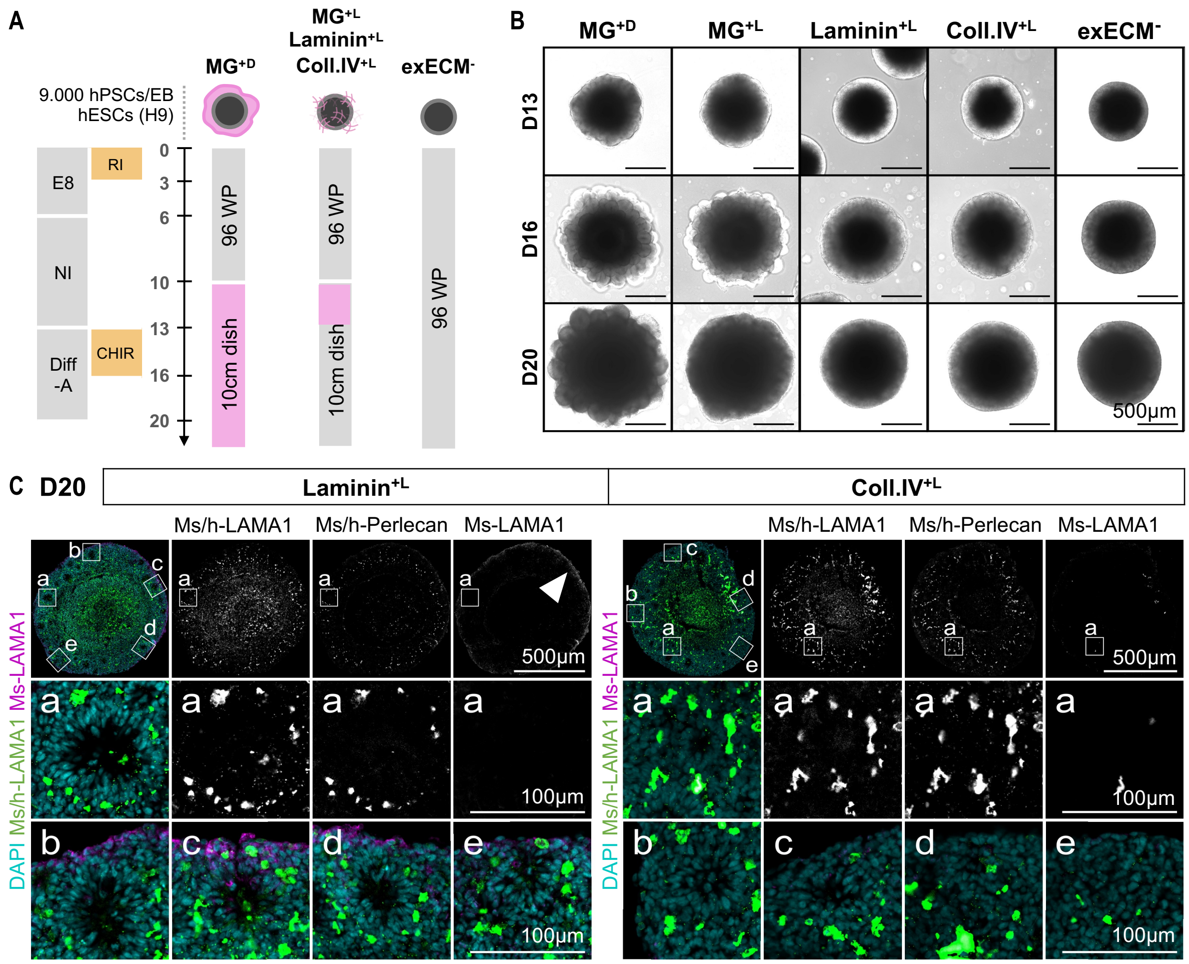

Appendix Figure S6. (Related to Fig. 2J-K) **Exposure to purified Laminin of Collagen IV does not have the same effect on organoid morphogenesis as exposure to Matrigel. (A)** Experimental paradigm to test the effect of liquid embedding with purified Laminin or Collagen IV on organoid development. **(B)** Brightfield imaging of organoids from the same batch exposed to the five different experimental conditions, at D13, D16, and D20. Images of Laminin^{+L} and Coll.IV^{+L} organoids at D20 are as in **Fig.2K**. **(C)** Co-staining of Ms-LAMA1, Ms/h-LAMA1, and Ms/h-Perlecan in Laminin^{+L} and Coll.IV^{+L} organoids at D20. Note the accumulation of Ms-LAMA1 at the organoid surface in Laminin^{+L} conditions (white arrowhead; zoom-in in **Cb-Ce**). Whole-organoid composite images are as in **Fig.2K**.

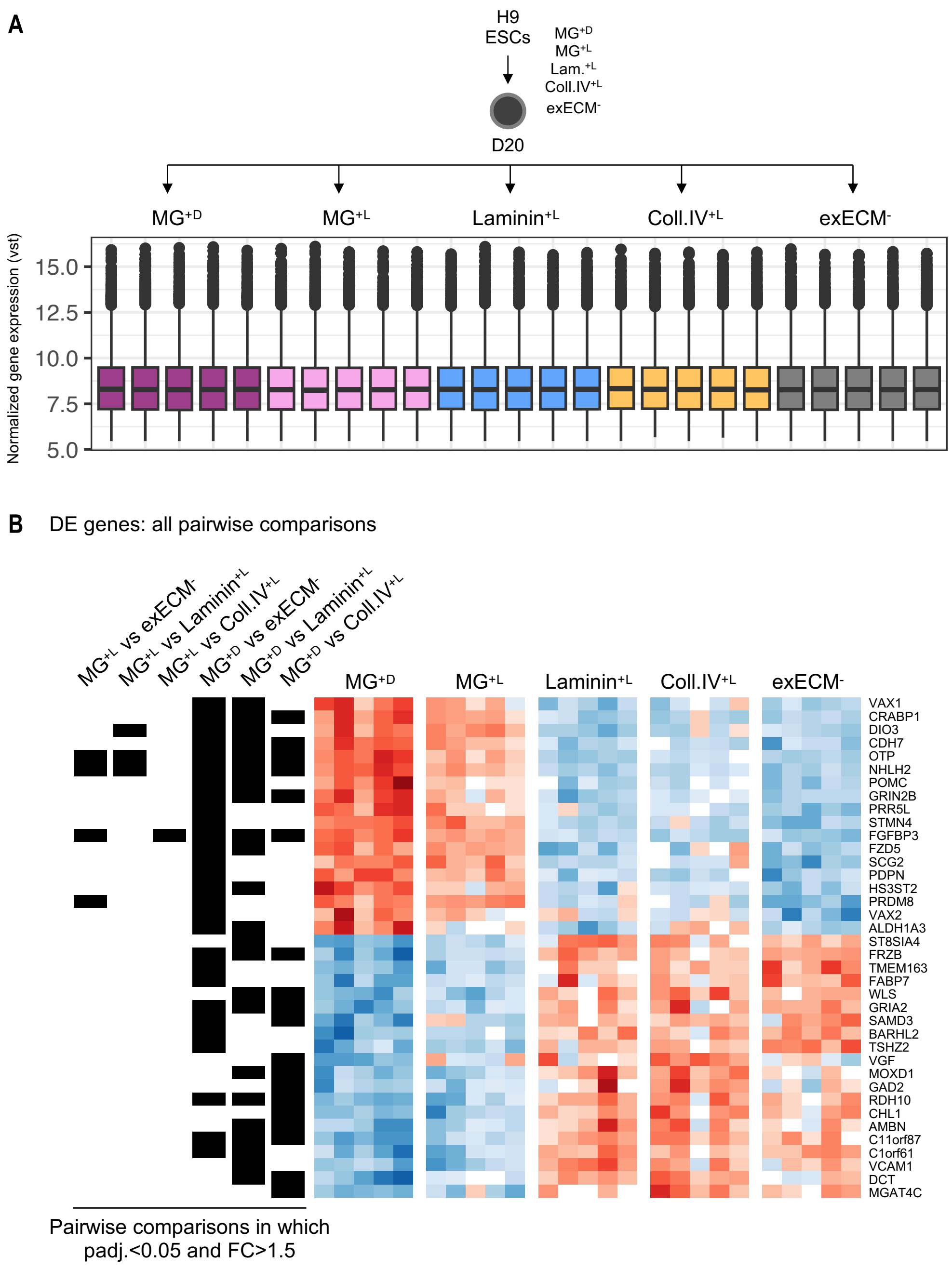

Appendix Figure S7. (Related to Fig. 3) **Bulk RNA sequencing analysis of organoids at D20. (A)** Experimental paradigm and normalized gene expression (after variance stabilizing transformation, vst) of each of the 25 analyzed organoids. **(D)** Normalized (vst) and row-scaled expression levels of genes found differentially expressed (adjusted p value, padj., below 0.05 and fold change, FC, above 1.5) considering all pairwise comparisons between single experimental conditions.

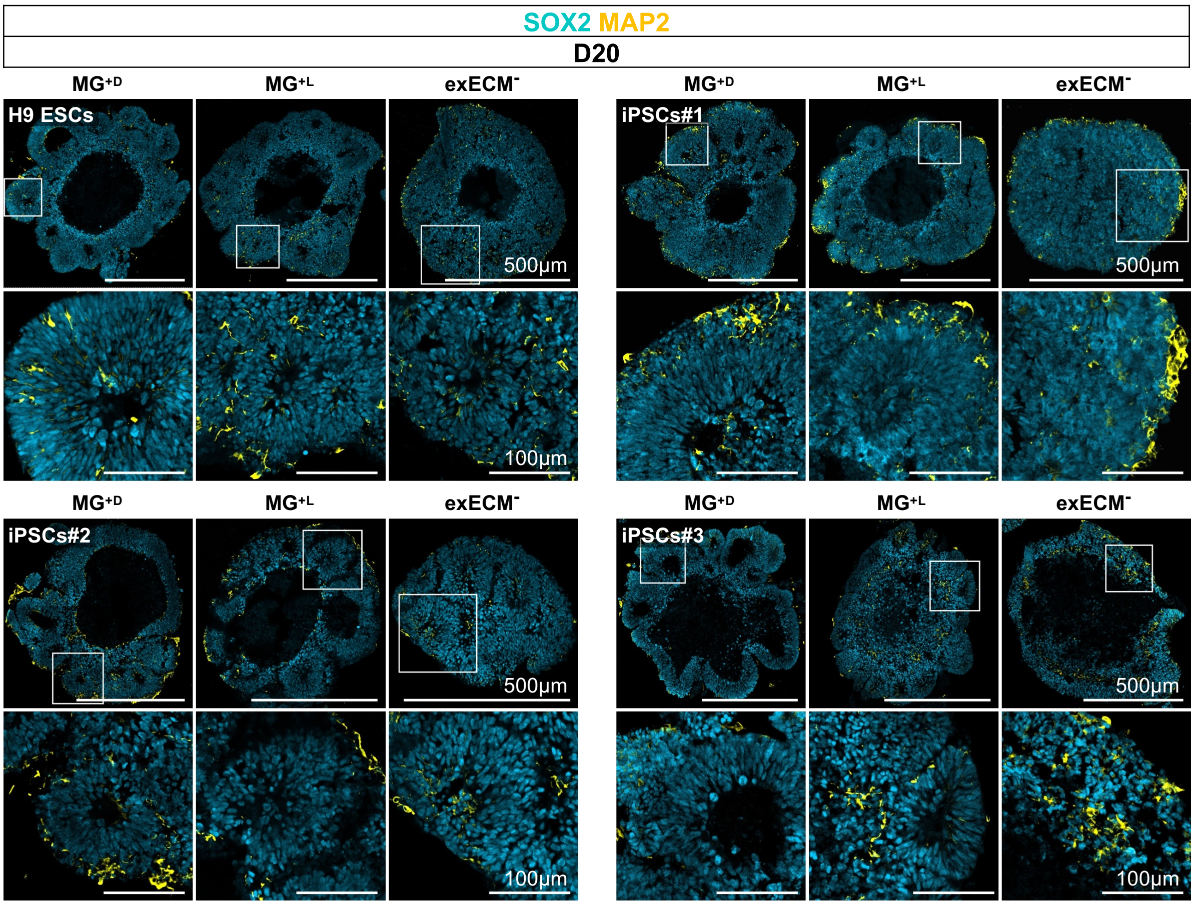

Appendix Figure S8. (Related to Fig. 3B) **The onset of neurogenesis occurs at around D20.** At D20, few scattered neurons (MAP2^{+}) are seen mainly at the outer organoid surface or surrounding neural rosettes (SOX2^{+}), in all conditions and cell lines. Bottom panels: magnification of inset. Images of H9 ESCs are as in **Fig.3B**.

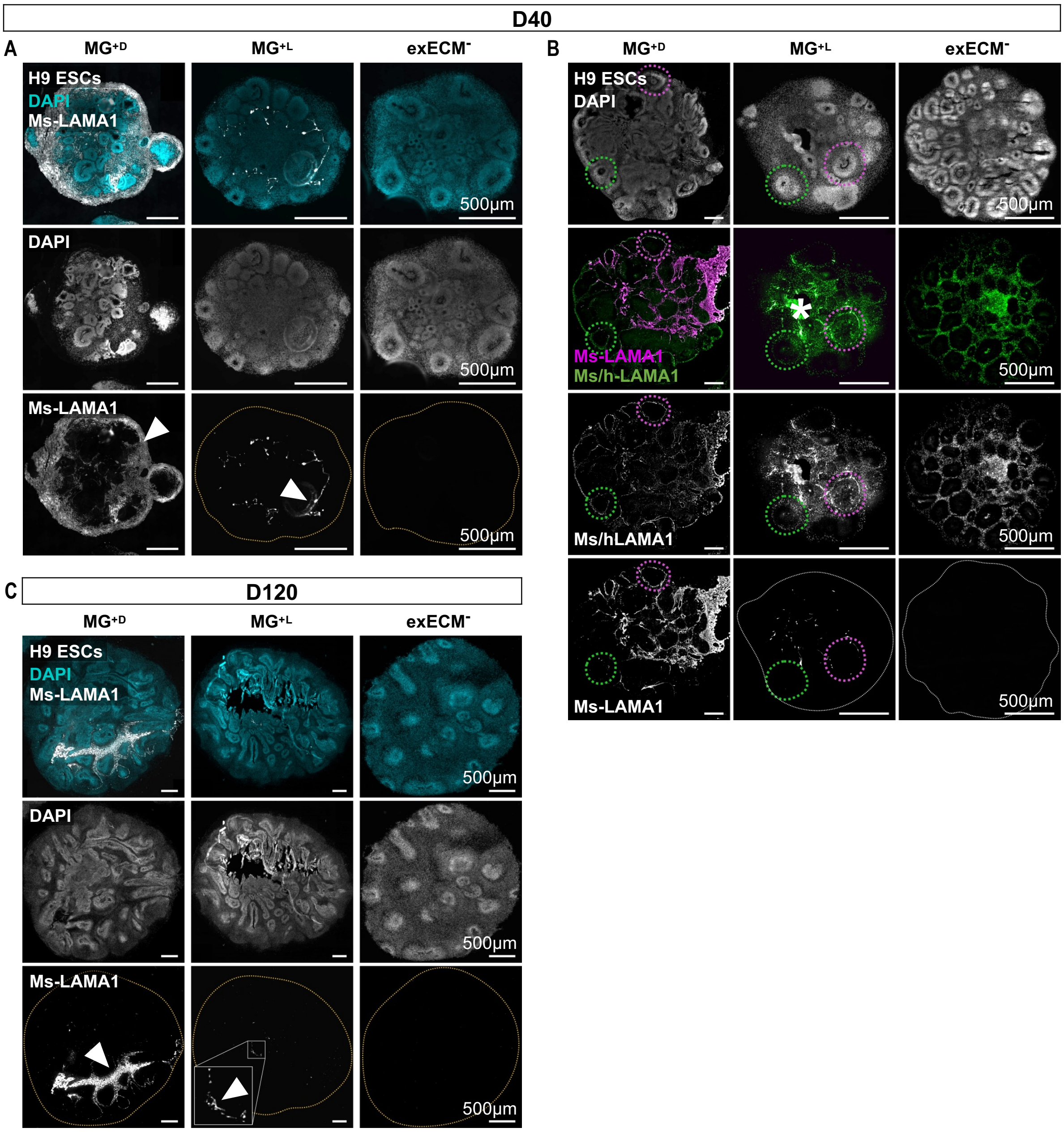

Appendix Figure S9. (Related to Fig. 4 and 5) **Matrigel shows long-term permanence within the organoid tissue. (A)** At D40, MG^{+D} organoids remain encapsulated by Matrigel (white arrowhead); and Matrigel remnants are visible within the tissue of MG^{+L} organoids (marked by Ms-LAMA1; white arrowhead). **(B)** Co-staining of Ms-LAMA1 and Ms/h-LAMA1 at D40. In MG^{+} organoids some neural rosettes are surrounded by Matrigel-derived ECM (Ms-LAMA1^{+}; magenta-delimited areas) while others are surrounded by endogenously produced ECM (Ms-LAMA1^{-} Ms/h-LAMA1^{+}; green-delimited areas). In exECM- organoids, there is abundant endogenous production of LAMA1 (Ms/h-LAMA1^{+}), which surrounds all neural rosettes. **(C)** At D120, large Ms-LAMA1^{+} regions are visible in MG^{+D} organoids (white arrowhead), when Matrigel has been engulfed by the growing tissue; and Matrigel remnants are still present in MG^{+L} organoids (white arrowhead). ExECM^{-} organoids show complete absence of Ms-LAMA1 staining, as expected.

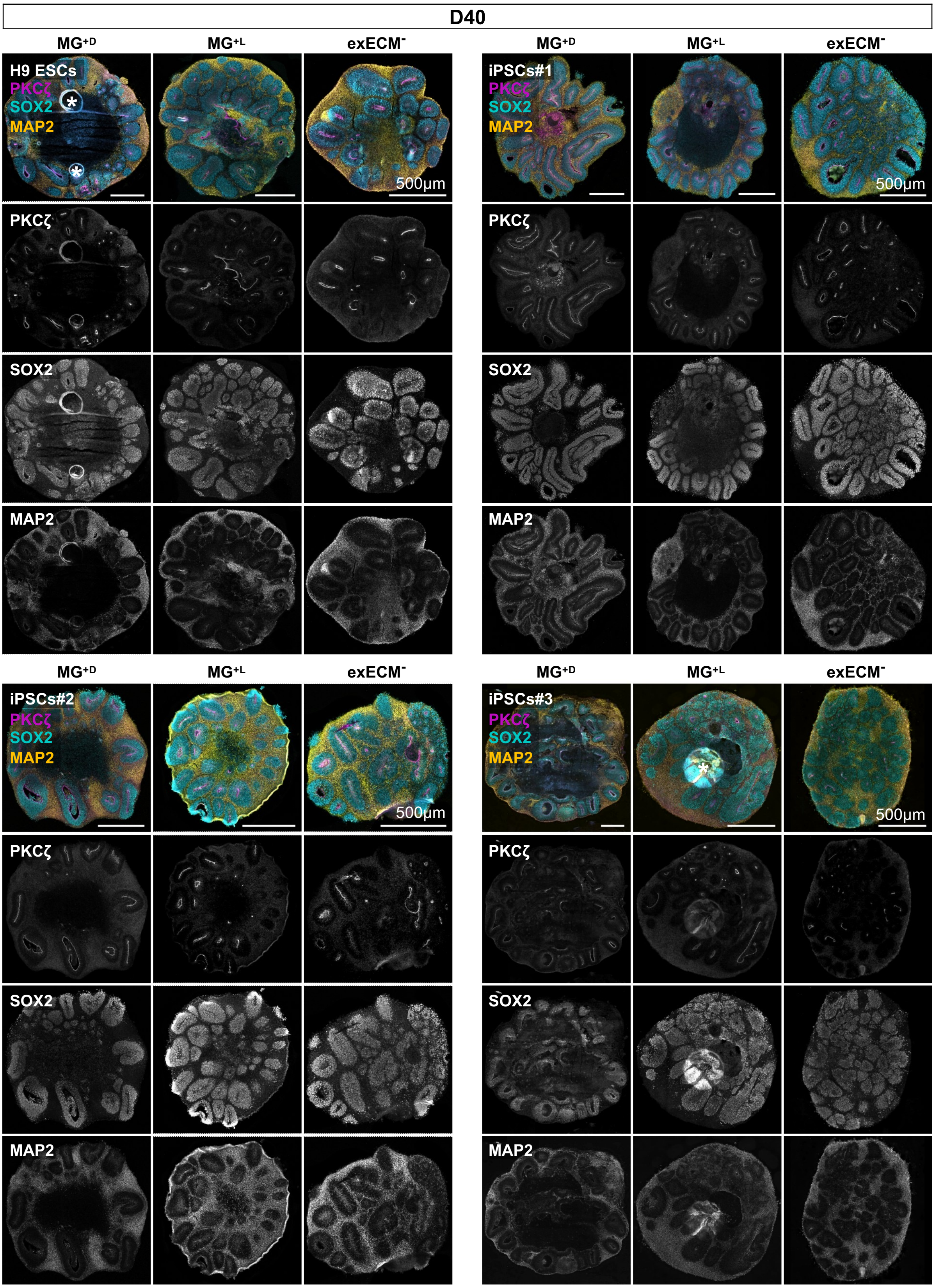

Appendix Figure S10. (Related to Fig. 4B) **Organization of neural progenitors and neurons is comparable between conditions at D40.** Organoids from all conditions and cell lines show abundant neural rosettes with PKCζ$^+$ ventricular zone, and inside-out organization of SOX2$^+$ neural progenitors and MAP2$^+$ neurons. *: staining artifact. A zoomed-in view of one rosette of H9 ESC-derived organoids is shown in **Fig. 4B**.

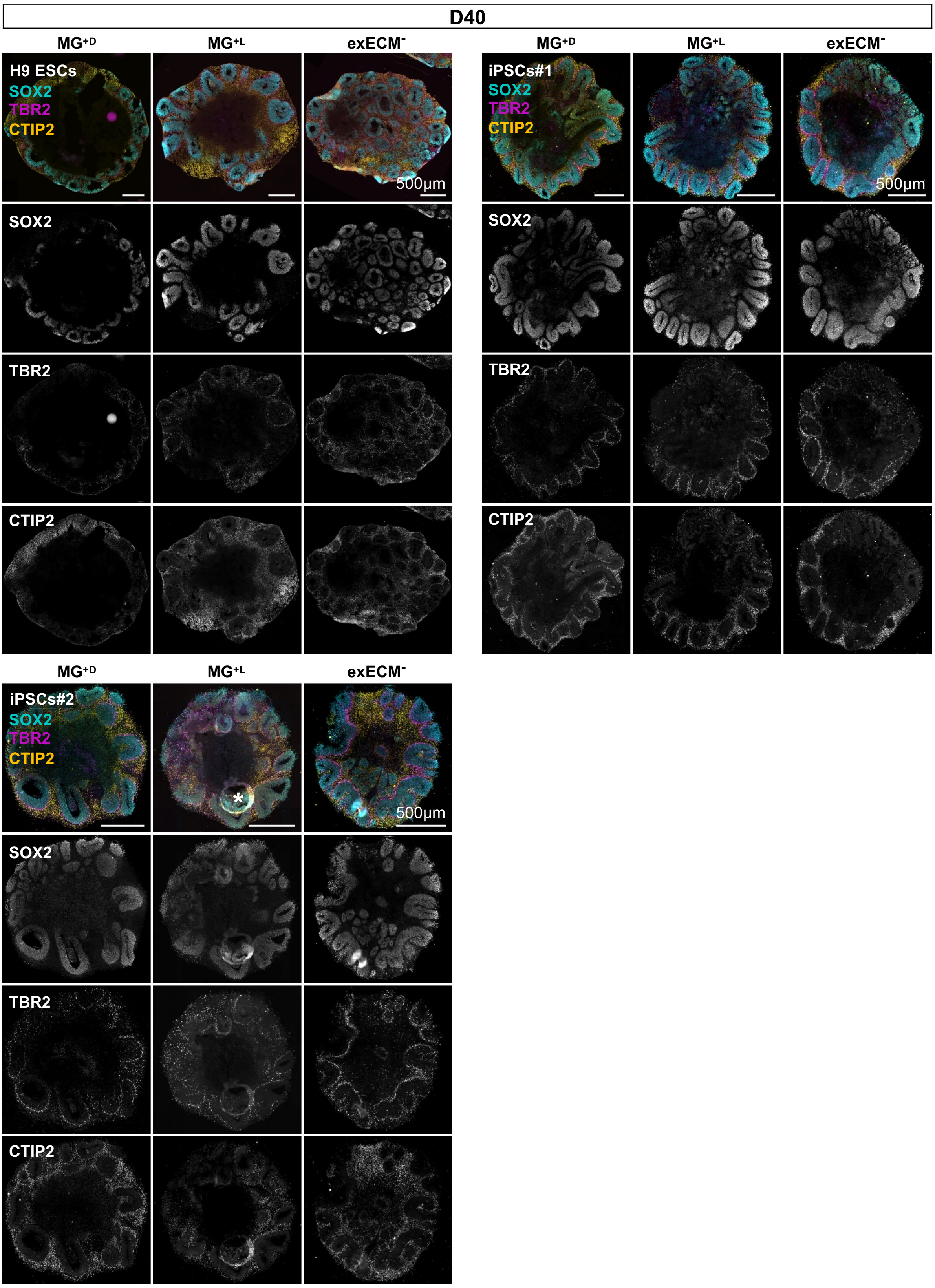

Appendix Figure S11. (Related to Fig. 4C) **Tissue identity and organization of dorsal-cortical cell types is comparable between conditions at D40.** Radial glia (SOX2⁺), dorsal intermediate progenitors (TBR2⁺) and early born excitatory neurons (CTIP2⁺) show layered arrangement in neural rosettes of organoids from all conditions and cell lines. *: staining artifact. A zoomed-in view of one rosette of H9 ESC-derived organoids is shown in **Fig. 4C**.

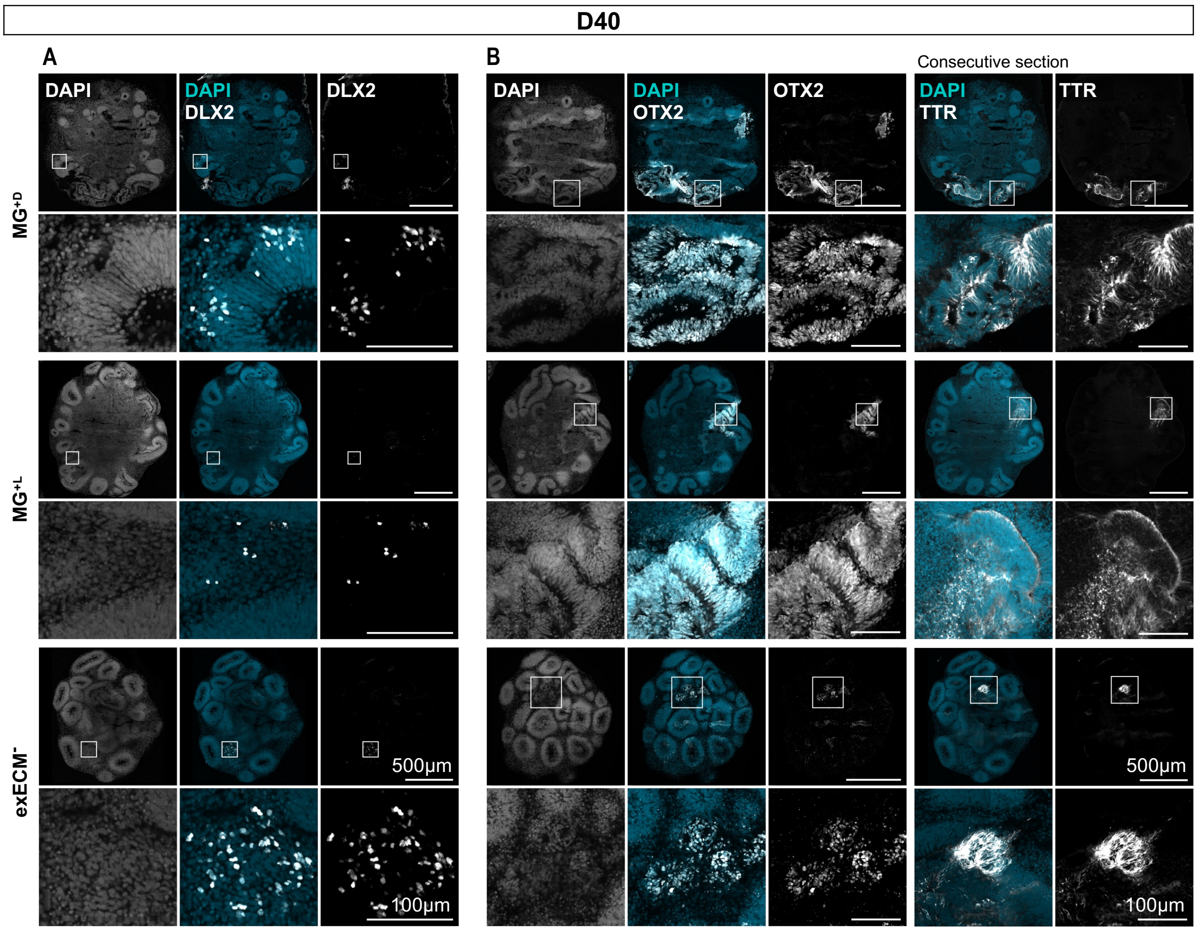

Appendix Figure S12. (Related to Fig. 4D and H) **(A)** At D40, few DLX2^{+} ventral neural progenitor cells are seen in organoids from all conditions. **(B)** Optic cup-like regions show convoluted morphology and are marked by the expression of OTX2 and TTR (shown in consecutive sections of the same organoid). Bottom panels: magnification of inset. Whole-organoid composite images of DAPI/OTX2 immunostaining are as in **Fig. 4D**.

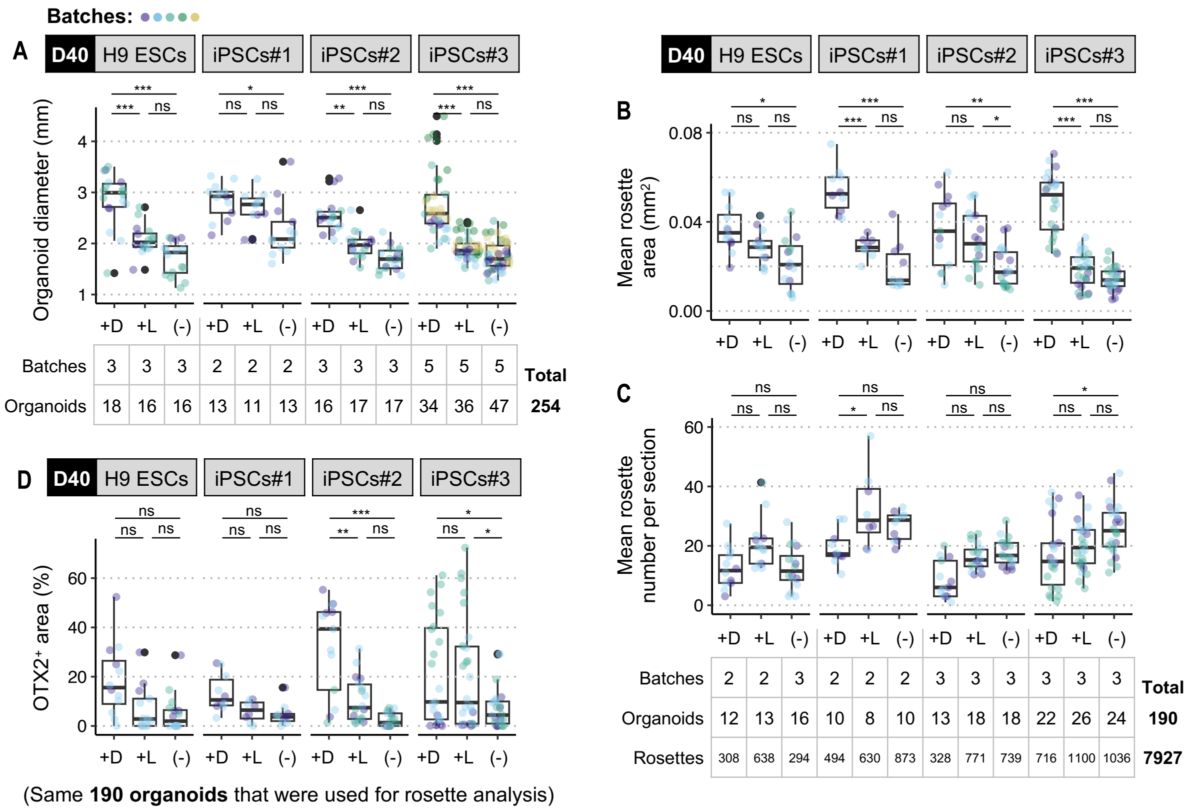

Appendix Figure S13. (Related to Fig. 4E-H) **Number of organoids and batches used to quantify rosette metrics and percentage of OTX2$^+$ tissue at D40. (A)** Organoid diameter. **(B)** Mean rosette area. **(C)** Mean rosette number per section. **(D)** Percentage of OTX2$^+$ area. Boxplots mark the median value; the two hinges correspond to the first and third quartiles (the 25th and 75th percentiles); and the whiskers extend from the hinge to the highest/lowest value no further than 1.5 * IQR from the hinge (where IQR is the inter-quartile range, or distance between the first and third quartiles). Each datapoint is an individual organoid (technical replicate); datapoint colors indicate organoid batches (biological replicates). Statistical tests are analysis of variance (ANOVA); $0 \le p < 0.001$, ***; $0.001 \le p < 0.01$, **; $0.01 \le p < 0.05$, *; $p \ge 0.05$, ns (see results of statistical tests in **Appendix Table S1**). The tables indicate the number of rosettes, organoids (technical replicates), and batches (biological replicates) used in these analyses.

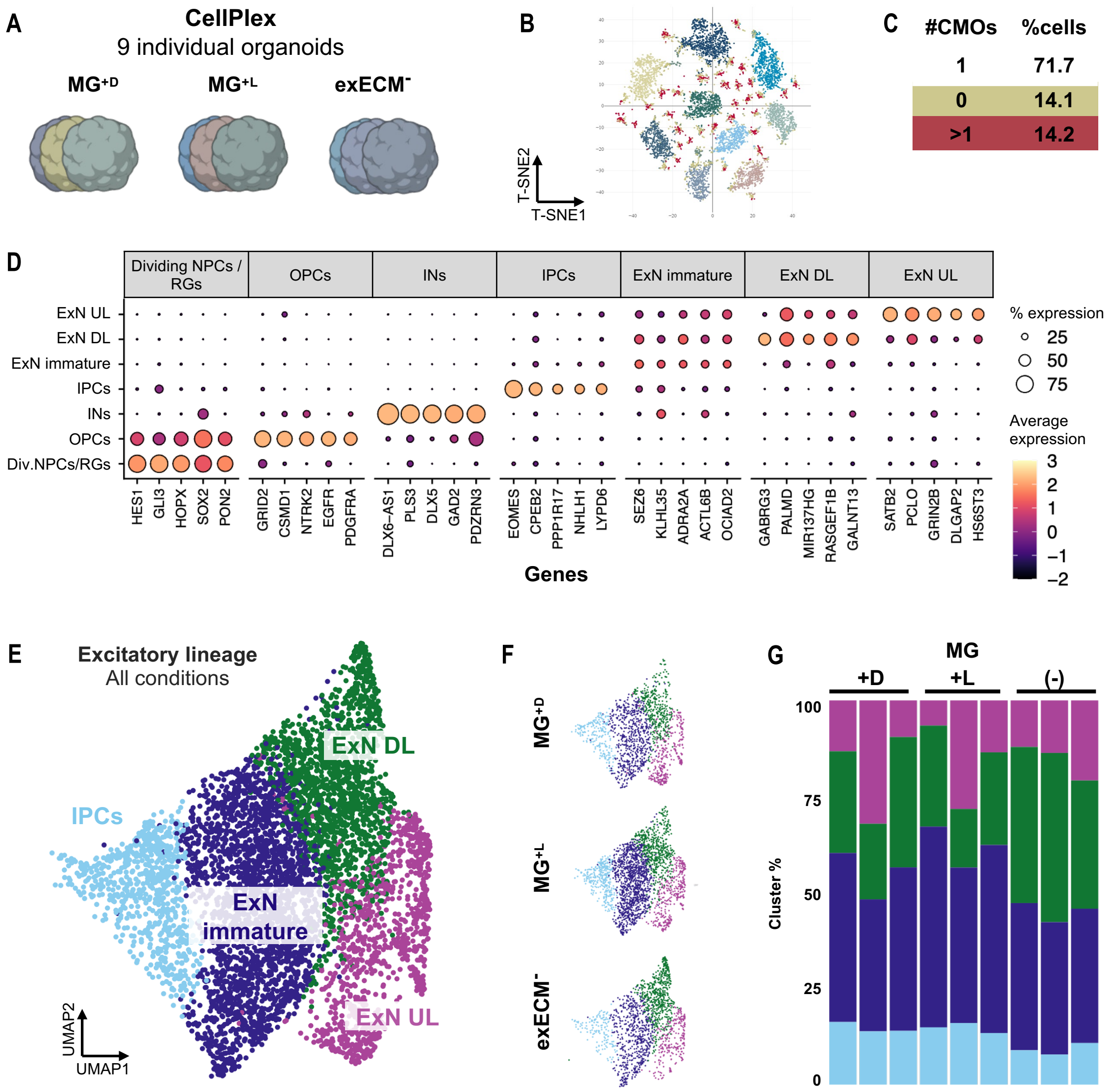

Appendix Figure S14. (Related to Fig. 5A-H) **Details of scRNAseq analysis. (A)** Nine individual organoids (3 per condition) were multiplexed with unique molecular identifiers (Cell Multiplexing Oligos, CMOs). **(B)** T-SNE projection of cells retrieved after sequencing, based on recovered CMOs. **(C)** Demultiplexing of used CMOs allows the assignment of individual cells to the respective organoid of origin. More than 70% of cells (corresponding to 6714 cells) were assigned to an individual organoid; cells tagged with zero (12.9%) or more than one CMO (15%) were excluded from the downstream analysis. Here, one of two sequenced libraries is shown; in the second library, about 56% of cells (7756 cells) were assigned to a unique CMO. **(D)** Top 5 cluster markers of telencephalic clusters: dividing NPCs/radial glia progenitors (RGs), oligodendrocyte precursor cells (OPCs), interneurons (INs), intermediate progenitor cells (IPCs), immature excitatory neurons (ExN), deep-layer excitatory neurons (ExNs DL), and upper-layer ExNs (ExNs UL). **(E)** Subset of the excitatory neuron lineage in UMAP projection after filtering and exclusion of non-telencephalic clusters; selected clusters: IPCs, immature exNs, deep-layer ExNs and upper-layer ExNs. **(F)** UMAP projection of the same dataset, split by each individual condition. **(G)** Percentage of cells in each cluster of the excitatory neuron lineage, per condition and per organoid.

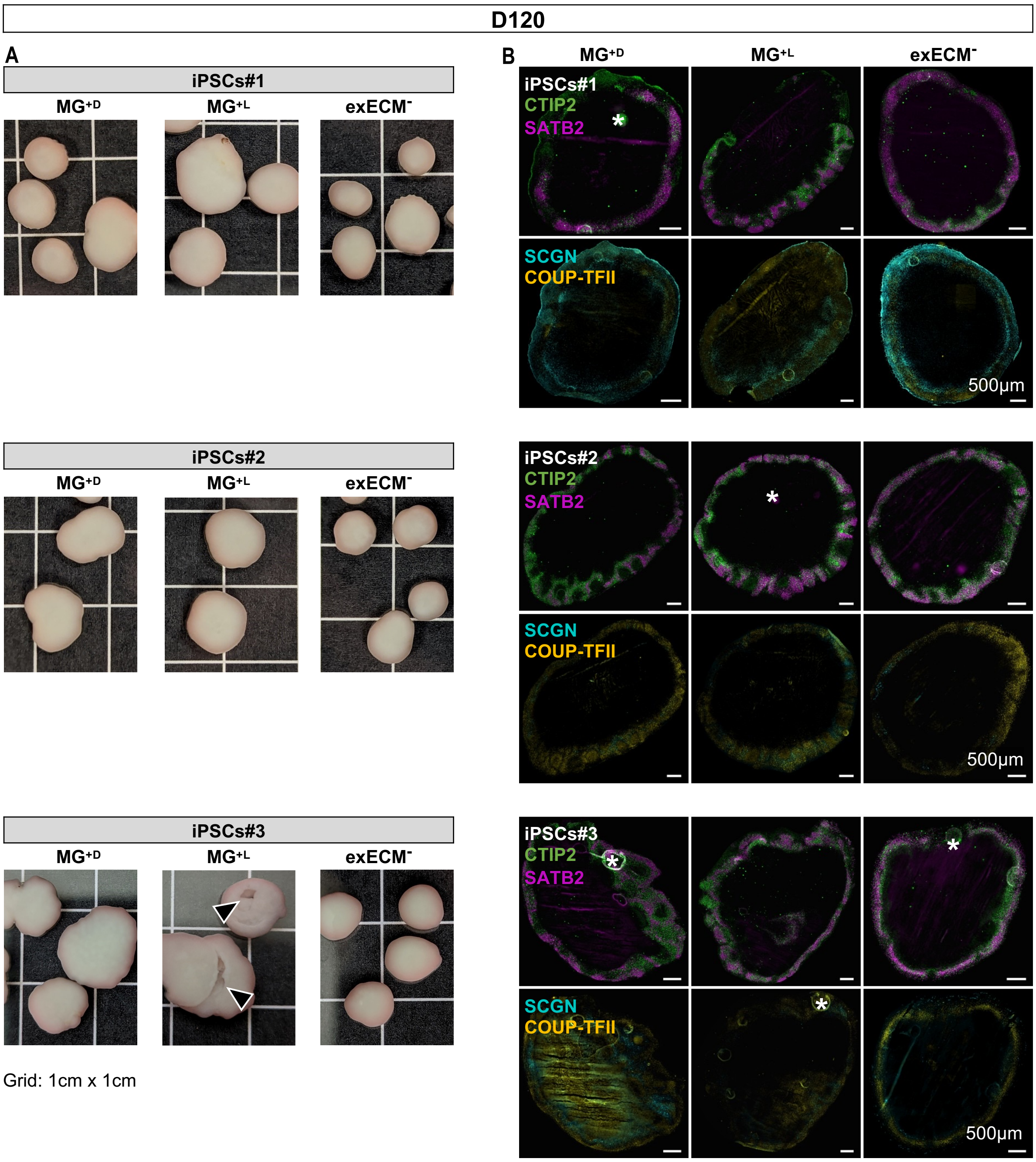

Appendix Figure S15. (Related to Fig. 5I) **Organoids cultured for 120 days show size dependent on Matrigel exposure, but comparable cell types and tissue morphology. (A)** Although organoid size is variable at D120, exECM$^-$ organoids are generally smaller than MG^{+D} or MG^{+L} organoids. Overgrowth may cause tissue damage, as seen in iPSCs#3-derived MG^{+L} organoids (black arrowheads). Grid size: 1cm x 1cm. **(B)** Tissue immunostaining of organoids at D120 shows abundant deep- and upper-layer neurons (CTIP2$^+$ and SATB2$^+$, respectively), as well as less abundant populations of interneurons (SCGN$^+$ and COUP-TFII$^+$) across all conditions and cell lines. *: staining artifacts.

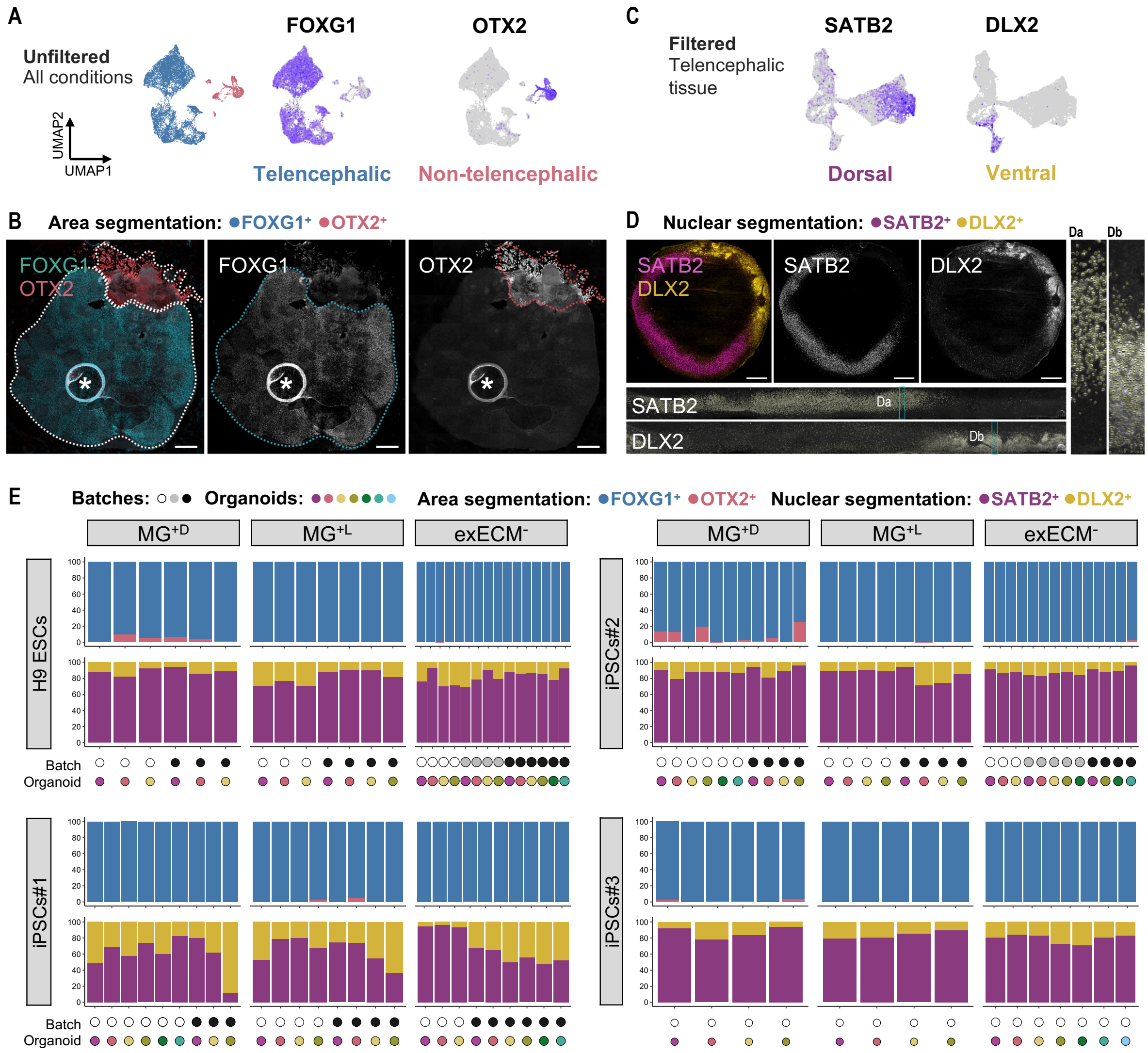

F

		H9 ESCs			iPSCs#1			iPSCs#2			iPSCs#3			
		+D	+L	(-)	+D	+L	(-)	+D	+L	(-)	+D	+L	(-)	Total organoids
Batches		2	2	3	2	2	2	2	2	3	1	1	1	
Organoids	●FOXG1 ●OTX2	6	7	16	9	8	10	11	8	14	6	5	8	**108**
	●SATB2 ●DLX2	6	7	14	9	8	9	10	8	12	4	4	7	**98**

Appendix Figure S16. (Related to Fig. 5J-K) **Patterning assessment at D120 of organoid development. (A-B)** Telencephalic/non-telencephalic tissue regions were identified by the expression of FOXG1 and OTX2, respectively. **(C-D)** Dorsal/ventral cells were segmented by nuclear staining of SATB2 and DLX2, respectively. **(E)** Results of the patterning analyses for each single organoid analyzed. **(F)** Number of organoids (technical replicates) and batches (biological replicates) used in these analyses.

Appendix Table S1. Statistical analyses. Analysis of variance (ANOVA); 0 ≤ p < 0.001, ***; 0.001 ≤ p < 0.01, **; 0.01 ≤ p < 0.05, *; p ≥ 0.05, ns. The influence on the quantitative variable was calculated from the interaction between cell line (H9, iPSCs#1 iPSCs#2, and iPSCs#3) and Matrigel condition (Drop, MG^{+D}; Liq, MG^{+L}; Null, $exECM^{-}$).

Figure and data	Comparison		Mean difference	Lower range	Upper range	Adj. p-val.	
Fig. 2F: number of rosettes / section D13	All lines: Liq-Drop		1.807692	-0.04706	3.662443	0.057773	ns
	All lines: Null-Drop		-9.68462	-11.4892	-7.88	3.08E-10	***
	All lines: Null-Liq		-11.4923	-13.095	-9.88962	3.08E-10	***
Appendix Fig. S2: number of rosettes / section D13	H9 ESCs	Liq-Drop	-0.925	-5.81414	3.964136	0.999966	ns
	H9 ESCs	Null-Drop	-11.6571	-15.9247	-7.38955	3.09E-10	***
	H9 ESCs	Null-Liq	-10.7321	-15.3003	-6.16396	6.29E-10	***
	iPSCs#1	Liq-Drop	3.1	-2.22262	8.422617	0.724825	ns
	iPSCs#1	Null-Drop	-6.375	-11.9415	-0.80847	0.011356	*
	iPSCs#1	Null-Liq	-9.475	-14.3641	-4.58586	2.31E-07	***
	iPSCs#2	Liq-Drop	2.5	-3.14549	8.145488	0.941835	ns
	iPSCs#2	Null-Drop	-7.4	-13.1491	-1.65092	0.002195	**
	iPSCs#2	Null-Liq	-9.9	-14.6358	-5.16417	2.13E-08	***
	iPSCs#3	Liq-Drop	1.654545	-3.90475	7.213838	0.99746	ns
	iPSCs#3	Null-Drop	-13.8	-19.1699	-8.43007	3.17E-10	***
	iPSCs#3	Null-Liq	-15.4545	-19.6074	-11.3017	3.08E-10	***

Figure and data	Comparison		Mean difference	Lower range	Upper range	Adj. p-val.	
Fig. 2F: number of rosettes / section D16	All lines: Liq-Drop		2.672587	0.058384	5.286789	0.043854	*
	All lines: Null-Drop		-9.94048	-12.5392	-7.34172	5.33E-14	***
	All lines: Null-Liq		-12.6131	-14.8474	-10.3787	2.31E-14	***
Appendix Fig. S2: number of rosettes / section D16	H9 ESCs	Liq-Drop	3.638889	-2.86376	10.14153	0.782386	ns
	H9 ESCs	Null-Drop	-9.83333	-16.0023	-3.66438	2.69E-05	***
	H9 ESCs	Null-Liq	-13.4722	-18.9127	-8.03172	6.23E-12	***
	iPSCs#1	Liq-Drop	5.233333	-2.40555	12.87221	0.498767	ns
	iPSCs#1	Null-Drop	-4.1	-12.3765	4.176517	0.889129	ns
	iPSCs#1	Null-Liq	-9.33333	-16.4566	-2.21004	0.001462	**
	iPSCs#2	Liq-Drop	1.296703	-6.88325	9.476653	0.999995	ns
	iPSCs#2	Null-Drop	-11.3571	-19.4342	-3.28009	0.000407	***
	iPSCs#2	Null-Liq	-12.6538	-19.3744	-5.93333	2.66E-07	***
	iPSCs#3	Liq-Drop	0.844444	-6.51246	8.201348	1	ns
	iPSCs#3	Null-Drop	-14.1556	-21.5125	-6.79865	1.33E-07	***
	iPSCs#3	Null-Liq	-15	-21.3713	-8.62873	6.08E-11	***

Figure and data	Comparison		Mean difference	Lower range	Upper range	Adj. p-val.	
Fig. 2F: number of rosettes / section D20	All lines: Liq-Drop		4.350941	0.683021	8.018862	0.015553	*
	All lines: Null-Drop		0.17963	-3.49766	3.856916	0.992641	ns
	All lines: Null-Liq		-4.17131	-7.05652	-1.2861	0.002324	**
Appendix Fig. S2: number of rosettes / section D20	H9 ESCs	Liq-Drop	4.179487	-4.43373	12.7927	0.901146	ns
	H9 ESCs	Null-Drop	4.458333	-4.24668	13.16335	0.863703	ns
	H9 ESCs	Null-Liq	0.278846	-6.02538	6.583075	1	ns
	iPSCs#1	Liq-Drop	1.269841	-9.95368	12.49336	1	ns
	iPSCs#1	Null-Drop	1.78E-15	-10.4986	10.49864	1	ns
	iPSCs#1	Null-Liq	-1.26984	-12.4934	9.953676	1	ns
	iPSCs#2	Liq-Drop	0.2	-11.3007	11.70068	1	ns
	iPSCs#2	Null-Drop	0.636364	-10.6666	11.93931	1	ns
	iPSCs#2	Null-Liq	0.436364	-9.29453	10.16725	1	ns
	iPSCs#3	Liq-Drop	6.333333	-7.55506	20.22173	0.933532	ns
	iPSCs#3	Null-Drop	-7.8125	-21.8243	6.199349	0.784591	ns
	iPSCs#3	Null-Liq	-14.1458	-21.798	-6.4937	5.31E-07	***

Figure and data	Comparison		Mean difference	Lower range	Upper range	Adj. p-val.	
Fig. 4E: organoid diameter D40	All lines: Liq-Drop		-701.48	-845.905	-557.055	9.77E-14	***
	All lines: Null-Drop		-945.449	-1084.7	-806.195	9E-14	***
	All lines: Null-Liq		-243.969	-383.687	-104.25	0.000155	***
Appendix Fig. S13A: organoid diameter D40	H9 ESCs	Liq-Drop	-784.444	-1225.04	-343.846	9.02E-07	***
	H9 ESCs	Null-Drop	-1156.16	-1596.76	-715.565	2.53E-13	***
	H9 ESCs	Null-Liq	-371.719	-825.09	81.65228	0.229743	ns
	iPSCs#1	Liq-Drop	-164.933	-690.268	360.4022	0.996702	ns
	iPSCs#1	Null-Drop	-573.515	-1076.49	-70.5454	0.011135	*
	iPSCs#1	Null-Liq	-408.583	-933.918	116.7525	0.304588	ns
	iPSCs#2	Liq-Drop	-569.957	-1016.61	-123.303	0.002083	**
	iPSCs#2	Null-Drop	-831.263	-1277.92	-384.609	2.17E-07	***
	iPSCs#2	Null-Liq	-261.306	-701.14	178.5286	0.71929	ns
	iPSCs#3	Liq-Drop	-875.703	-1182.36	-569.043	2.06E-13	***
	iPSCs#3	Null-Drop	-1015.06	-1303.77	-726.36	1.11E-13	***
	iPSCs#3	Null-Liq	-139.36	-423.373	144.6522	0.900461	ns

Figure and data	Comparison		Mean difference	Lower range	Upper range	Adj. p-val.	
Fig. 4F: mean rosette area D40	All lines: Liq-Drop		-0.01873	-0.02327	-0.01418	3.29E-14	***
	All lines: Null-Drop		-0.02589	-0.03039	-0.0214	3.11E-15	***
	All lines: Null-Liq		-0.00717	-0.01151	-0.00282	0.000398	***
Appendix Fig. S13B: mean rosette area D40	H9 ESCs	Liq-Drop	-0.00822	-0.02227	0.005818	0.732574	ns
	H9 ESCs	Null-Drop	-0.01499	-0.02839	-0.00159	0.014429	*
	H9 ESCs	Null-Liq	-0.00677	-0.01986	0.006333	0.861132	ns
	iPSCs#1	Liq-Drop	-0.02555	-0.04219	-0.00891	5.8E-05	***
	iPSCs#1	Null-Drop	-0.03458	-0.05027	-0.01889	6.05E-10	***
	iPSCs#1	Null-Liq	-0.00903	-0.02567	0.007615	0.817901	ns
	iPSCs#2	Liq-Drop	-0.00435	-0.01712	0.008419	0.993016	ns
	iPSCs#2	Null-Drop	-0.01681	-0.02957	-0.00404	0.001295	**
	iPSCs#2	Null-Liq	-0.01246	-0.02415	-0.00076	0.025815	*
	iPSCs#3	Liq-Drop	-0.02991	-0.04007	-0.01975	1.21E-13	***
	iPSCs#3	Null-Drop	-0.03384	-0.04419	-0.02349	8.37E-14	***
	iPSCs#3	Null-Liq	-0.00393	-0.01386	0.005997	0.976648	ns

Figure and data	Comparison		Mean difference	Lower range	Upper range	Adj. p-val.	
Fig. 4G: mean rosette number per section D40	All lines: Liq-Drop		6.110504	2.719573	9.501434	9.81E-05	***
	All lines: Null-Drop		6.166103	2.810302	9.521904	6.98E-05	***
	All lines: Null-Liq		0.0556	-3.18591	3.297113	0.999094	ns
Appendix Fig. S13C: mean rosette number per section D40	H9 ESCs	Liq-Drop	8.470085	-2.01309	18.95326	0.247052	ns
	H9 ESCs	Null-Drop	0.069444	-9.93086	10.06975	1	ns
	H9 ESCs	Null-Liq	-8.40064	-18.1787	1.37741	0.170478	ns
	iPSCs#1	Liq-Drop	12.8625	0.440938	25.28406	0.035149	*
	iPSCs#1	Null-Drop	7.423333	-4.28783	19.13449	0.623851	ns
	iPSCs#1	Null-Liq	-5.43917	-17.8607	6.982396	0.951473	ns
	iPSCs#2	Liq-Drop	7.639316	-1.89211	17.17074	0.258153	ns
	iPSCs#2	Null-Drop	9.296724	-0.2347	18.82815	0.063227	ns
	iPSCs#2	Null-Liq	1.657407	-7.07158	10.38639	0.999971	ns
	iPSCs#3	Liq-Drop	3.020396	-4.56551	10.6063	0.975691	ns
	iPSCs#3	Null-Drop	8.715909	0.986489	16.44533	0.013092	*
	iPSCs#3	Null-Liq	5.695513	-1.71721	13.10823	0.319646	ns

Figure and data	Comparison		Mean difference	Lower range	Upper range	Adj. p-val.	
Fig. 4F: $OTX2^{+}$ area D40	All lines: Liq-Drop		-8.35492	-14.3973	-2.31252	0.003701	**
	All lines: Null-Drop		-16.725	-22.7043	-10.7458	1.27E-09	***
	All lines: Null-Liq		-8.37011	-14.173	-2.56725	0.002313	**

Appendix Fig. S13D: $OTX2^+$ area D40	H9 ESCs	Liq-Drop	-11.7291	-30.4953	7.037044	0.644639	ns
	H9 ESCs	Null-Drop	-13.5799	-31.4816	4.321929	0.339277	ns
	H9 ESCs	Null-Liq	-1.85073	-19.3546	15.65318	1	ns
	iPSCs#1	Liq-Drop	-7.40224	-29.6384	14.83389	0.994253	ns
	iPSCs#1	Null-Drop	-8.83626	-29.8007	12.12816	0.963001	ns
	iPSCs#1	Null-Liq	-1.43402	-23.6701	20.80211	1	ns
	iPSCs#2	Liq-Drop	-21.2544	-38.3169	-4.192	0.003208	**
	iPSCs#2	Null-Drop	-29.4243	-46.4868	-12.3619	2.97E-06	***
	iPSCs#2	Null-Liq	-8.16991	-23.7959	7.456046	0.851193	ns
	iPSCs#3	Liq-Drop	-0.45795	-13.8768	12.9609	1	ns
	iPSCs#3	Null-Drop	-14.1598	-27.8386	-0.481	0.035266	*
	iPSCs#3	Null-Liq	-13.7018	-26.9715	-0.43214	0.036207	*

**Figure and data**	**Comparison**		**Mean difference**	**Lower range**	**Upper range**	**Adj. p-val.**	
Fig. 5J: $\%FOXG1^+/OTX2^+$ areas D120	All lines: Liq-Drop		-3.2781	-5.22179	-1.33442	0.000345	***
	All lines: Null-Drop		-3.42248	-5.13665	-1.70832	2.08E-05	***
	All lines: Null-Liq		-0.14438	-1.9305	1.641741	0.979797	ns
	All conditions: H9 ESCs-iPSCs#1		-1.07702	-3.28318	1.129127	0.580094	ns
	All conditions: H9 ESCs-iPSCs#2		1.131396	-0.96833	3.231119	0.496984	ns
	All conditions: H9 ESCs-iPSCs#3		-0.86912	-3.30394	1.565702	0.787057	ns
	All conditions: iPSCs#1-iPSCs#2		2.20842	0.067705	4.349136	0.040498	*
	All conditions: iPSCs#1-iPSCs#3		0.207906	-2.26235	2.678165	0.996214	ns
	All conditions: iPSCs#2-iPSCs#3		-2.00051	-4.37621	0.375177	0.130116	ns
	H9 ESCs	Liq-Drop	-4.4824	-10.363	1.39823	0.320851	ns
	H9 ESCs	Null-Drop	-4.49332	-9.55334	0.566702	0.13243	ns
	H9 ESCs	Null-Liq	-0.01092	-4.80088	4.779034	1	ns
	iPSCs#1	Liq-Drop	0.86442	-4.2717	6.000536	0.99999	ns
	iPSCs#1	Null-Drop	0.067681	-4.78892	4.924279	1	ns
	iPSCs#1	Null-Liq	-0.79674	-5.81055	4.217071	0.999994	ns
	iPSCs#2	Liq-Drop	-7.1034	-12.0149	-2.19192	0.000292	***
	iPSCs#2	Null-Drop	-6.92347	-11.1823	-2.66468	2.49E-05	***
	iPSCs#2	Null-Liq	0.179931	-4.50474	4.864601	1	ns
	iPSCs#3	Liq-Drop	-1.286	-7.68648	5.114475	0.999938	ns
	iPSCs#3	Null-Drop	-1.0532	-6.76167	4.655274	0.999974	ns
	iPSCs#3	Null-Liq	0.232803	-5.79305	6.258653	1	ns

**Figure and data**	**Comparison**		**Mean difference**	**Lower range**	**Upper range**	**Adj. p-val.**	
Fig. 5K: $\%SATB2^+/DLX2^+$ nuclei D120	All lines: Liq-Drop		-1.48882	-8.77696	5.79933	0.87757	ns
	All lines: Null-Drop		0.92519	-5.65456	7.504939	0.939936	ns
	All lines: Null-Liq		2.414005	-4.30836	9.136366	0.66906	ns
	All conditions: H9 ESCs-iPSCs#1		-17.9547	-26.1807	-9.72863	9.06E-07	***
	All conditions: H9 ESCs-iPSCs#2		4.254357	-3.68739	12.1961	0.500644	ns
	All conditions: H9 ESCs-iPSCs#3		-0.48987	-10.1308	9.151043	0.999151	ns
	All conditions: iPSCs#1-iPSCs#2		22.20902	14.18729	30.23074	1.26E-09	***
	All conditions: iPSCs#1-iPSCs#3		17.46479	7.757888	27.17169	5.43E-05	***
	All conditions: iPSCs#2-iPSCs#3		-4.74423	-14.2114	4.722962	0.557299	ns
	H9 ESCs	Liq-Drop	-7.45806	-28.817	13.90085	0.989688	ns
	H9 ESCs	Null-Drop	-6.90264	-25.6356	11.83035	0.984187	ns
	H9 ESCs	Null-Liq	0.555414	-17.2163	18.32709	1	ns
	iPSCs#1	Liq-Drop	4.303088	-14.3517	22.95787	0.999747	ns
	iPSCs#1	Null-Drop	8.527653	-9.57014	26.62545	0.91049	ns
	iPSCs#1	Null-Liq	4.224565	-14.4302	22.87934	0.999789	ns
	iPSCs#2	Liq-Drop	-2.57959	-20.7901	15.63097	0.999998	ns
	iPSCs#2	Null-Drop	0.012746	-16.4254	16.45088	1	ns
	iPSCs#2	Null-Liq	2.592333	-14.9308	20.11545	0.999997	ns
	iPSCs#3	Liq-Drop	-3.21546	-30.3622	23.93123	1	ns
	iPSCs#3	Null-Drop	-7.72416	-31.7871	16.33881	0.994868	ns
	iPSCs#3	Null-Liq	-4.5087	-28.5717	19.55427	0.999968	ns

Appendix Table S2. Medium composition for telencephalic organoid culture. Medium components and recipe for 500 mL of Neural Induction medium (NI), Differentiation medium without vitamin A (Diff-A), and Differentiation medium with Vitamin A (Diff+A). All media were vacuum-filtered through a membrane with a pore size of 0.22 µm.

Medium	Components	Manufacturer	Catalog number	Recipe for 500 mL
**Neural induction**	DMEM/F12(1:1) 1x, with HEPES	ThermoFisher Scientific	11330057	500 mL
	N-2 Supplement (100X)	ThermoFisher Scientific	17502048	5 mL
	GlutaMAX supplement-I (100X)	ThermoFisher Scientific	35050-038	5 mL
	MEM Non-essential amino acid solution (100X)	ThermoFisher Scientific	11140050	5 mL
	Antibiotic-Antimycotic (100X)	ThermoFisher Scientific	15240062	5 mL
	Heparin solution (1 mg/mL in DPBS-/-)	Merck	H3149	500 µL
**Diff. medium - vit. A**	DMEM/F12(1:1) 1x, with HEPES	ThermoFisher Scientific	11330057	250 mL
	Neurobasal Medium	ThermoFisher Scientific	21103049	250 mL
	N-2 Supplement (100X)	ThermoFisher Scientific	17502048	2.5 mL
	B-27 Supplement (50X), minus vitamin A	ThermoFisher Scientific	12587010	10 mL
	Recombinant human Insulin solution	Merck	I9278	125 µL
	GlutaMAX supplement-I (100X)	ThermoFisher Scientific	35050-038	5 mL
	MEM Non-essential amino acid solution (100X)	ThermoFisher Scientific	11140050	2.5 mL
	Antibiotic-Antimycotic (100X)	ThermoFisher Scientific	15240062	5 mL
**Diff. medium + vit. A**	DMEM/F12(1:1) 1x, with HEPES	ThermoFisher Scientific	11330057	250 mL
	Neurobasal Medium	ThermoFisher Scientific	21103049	250 mL
	N-2 Supplement (100X)	ThermoFisher Scientific	17502048	2.5 mL
	B-27 Supplement (50X), serum free	ThermoFisher Scientific	17504044	10 mL
	Recombinant human Insulin solution	Merck	I9278	125 µL
	GlutaMAX supplement-I (100X)	ThermoFisher Scientific	35050-038	5 mL
	MEM Non-essential amino acid solution (100X)	ThermoFisher Scientific	11140050	2.5 mL
	Antibiotic-Antimycotic (100X)	ThermoFisher Scientific	15240062	5 mL
	Sodium bicarbonate	Merck	S5761	1 g

Appendix Table S3. Antibodies used in this study.

Primary antibodies			
**Species**	**Antigen**	**Manufacturer**	**Catalog#**
Chicken	GFP	Aves Labs	GFP-1020
Mouse	PKCζ	Santa Cruz Biotechnology	sc-17781
Rabbit	PKCζ	Santa Cruz Biotechnology	sc-216
Mouse	Ms/h-Fibronectin	R&D Systems	MAB1918
Mouse	Ms/h-Perlecan	Invitrogen	13-4400
Rabbit	Ms/h-LAMA1	Abcam	ab11575
Rat	Ms-LAMA1	R&D Systems	MAB4656
Rabbit	Lumican	Abcam	ab168348
Mouse	SOX2	R&D Systems	MAB2018
Goat	SOX2	R&D Systems	AF2018
Chicken	MAP2	Thermo Fisher Scientific	PA1-10005
Rabbit	TBR2	Abcam	ab23345
Sheep	TBR2	R&D Systems	AF6166
Rat	CTIP2	Abcam	ab18465
Mouse	SATB2	Abcam	ab51502
Goat	OTX2	Neuromics	GT15095
Sheep	TTR	AbD Serotec	ahp 1837
Mouse	DLX2	Santa Cruz Biotechnology	sc-393879
Rabbit	SCGN	Sigma-Aldrich	HPA006641
Mouse	COUP-TFII	R&D Systems	pp-h7147-00

Secondary antibodies		
**Antibody**	**Manufacturer**	**Catalog#**
Alexa Fluor 488 Donkey anti-rabbit	Invitrogen	A21206
Alexa Fluor 488 Donkey anti-mouse	Invitrogen	A21202
Alexa Fluor 488 Donkey anti-chicken	Jackson ImmunoResearch	703-545-155
Alexa Fluor 488 Donkey anti-goat	Invitrogen	A11055
Alexa Fluor 568 Donkey anti-rabbit	Invitrogen	A10042
Alexa Fluor 568 Donkey anti-mouse	Invitrogen	A10037
Alexa Fluor 568 Donkey anti-goat	Invitrogen	A11057
Alexa Fluor 647 Donkey anti-rabbit	Invitrogen	A31573
Alexa Fluor 647 Donkey anti-mouse	Invitrogen	A31571
Alexa Fluor 647 Donkey anti-goat	Invitrogen	A21447
Alexa Fluor 647 Donkey anti-rat	Jackson ImmunoResearch	712-605-150
