# Supplementary figures and images for: Morphogenesis and development of human telencephalic organoids in the absence and presence of exogenous extracellular matrix (part 1 of 2)
Source: EMBO J. 2023 Oct 16;42(22):e113213. doi: 10.15252/embj.2022113213 (PMC10646563; doi:10.15252/embj.2022113213)

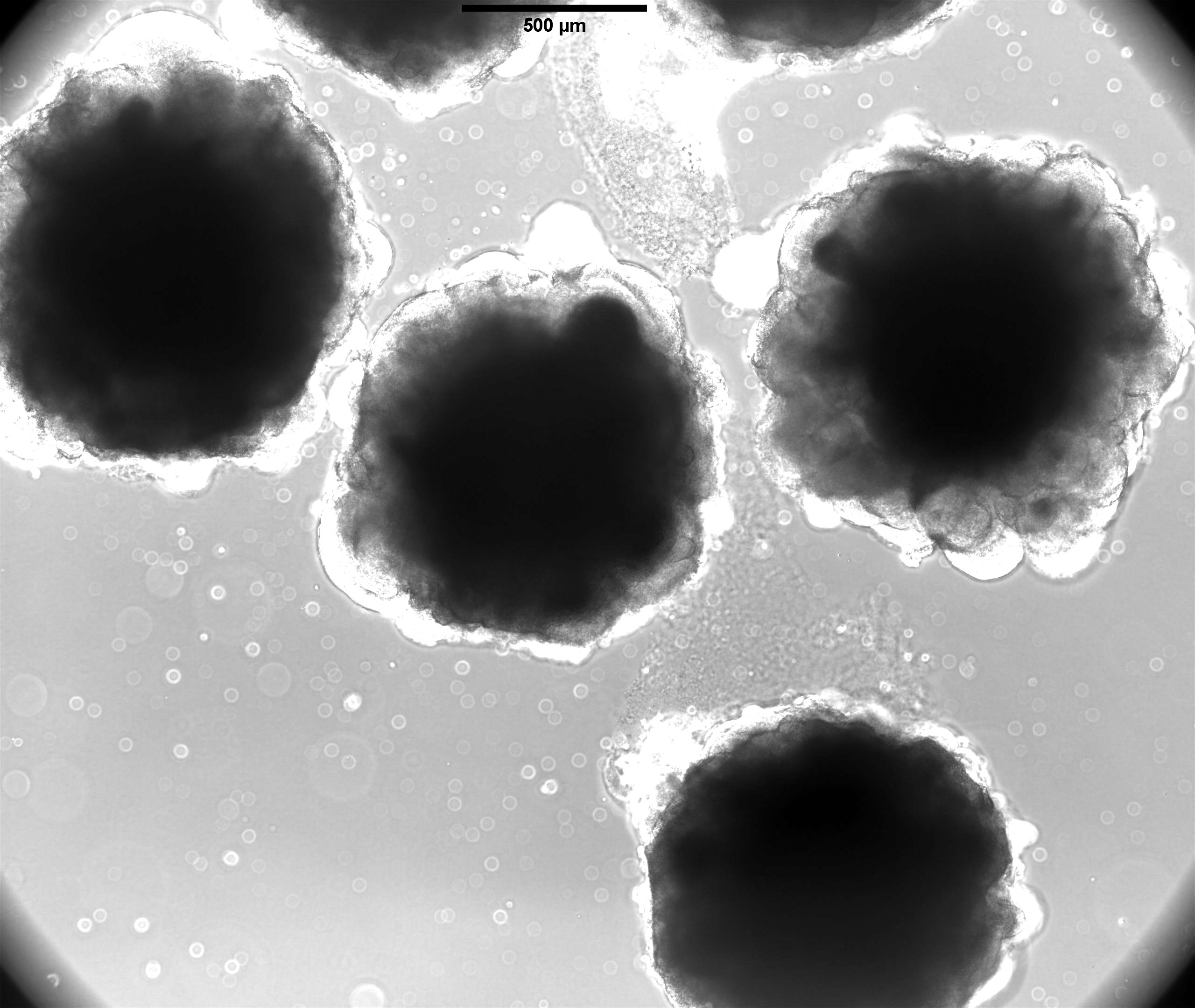

Supplement: Supplementary file 2 — Source Data for Figure 1 [file EMBJ-42-e113213-s003.zip › Figure1/Fig1B/Fig1B_iPSC3_D20_MGliq.jpg]

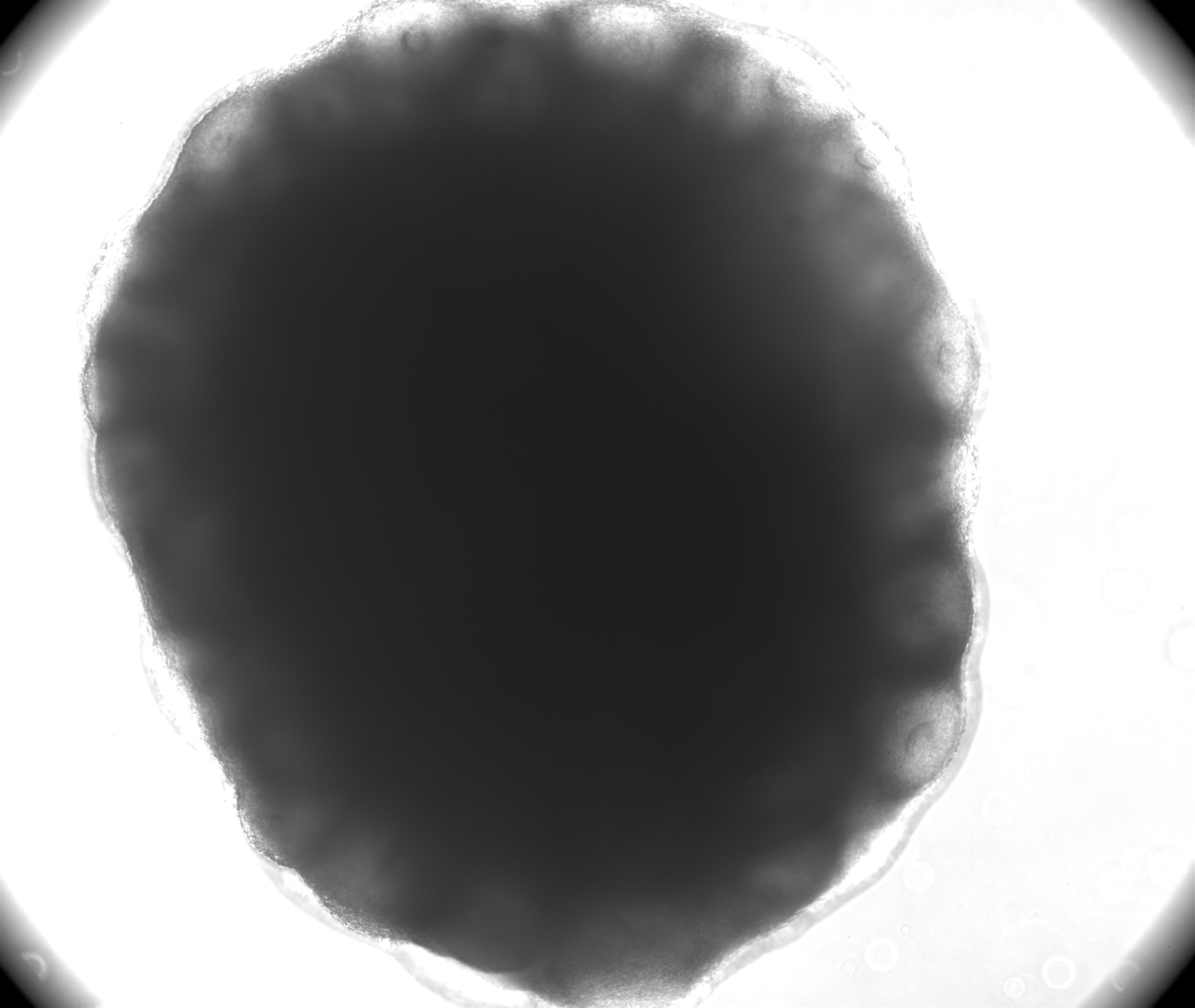

Supplement: Supplementary file 2 — Source Data for Figure 1 [file EMBJ-42-e113213-s003.zip › Figure1/Fig1B/Fig1B_iPSC1_D40_MGliq.jpg]

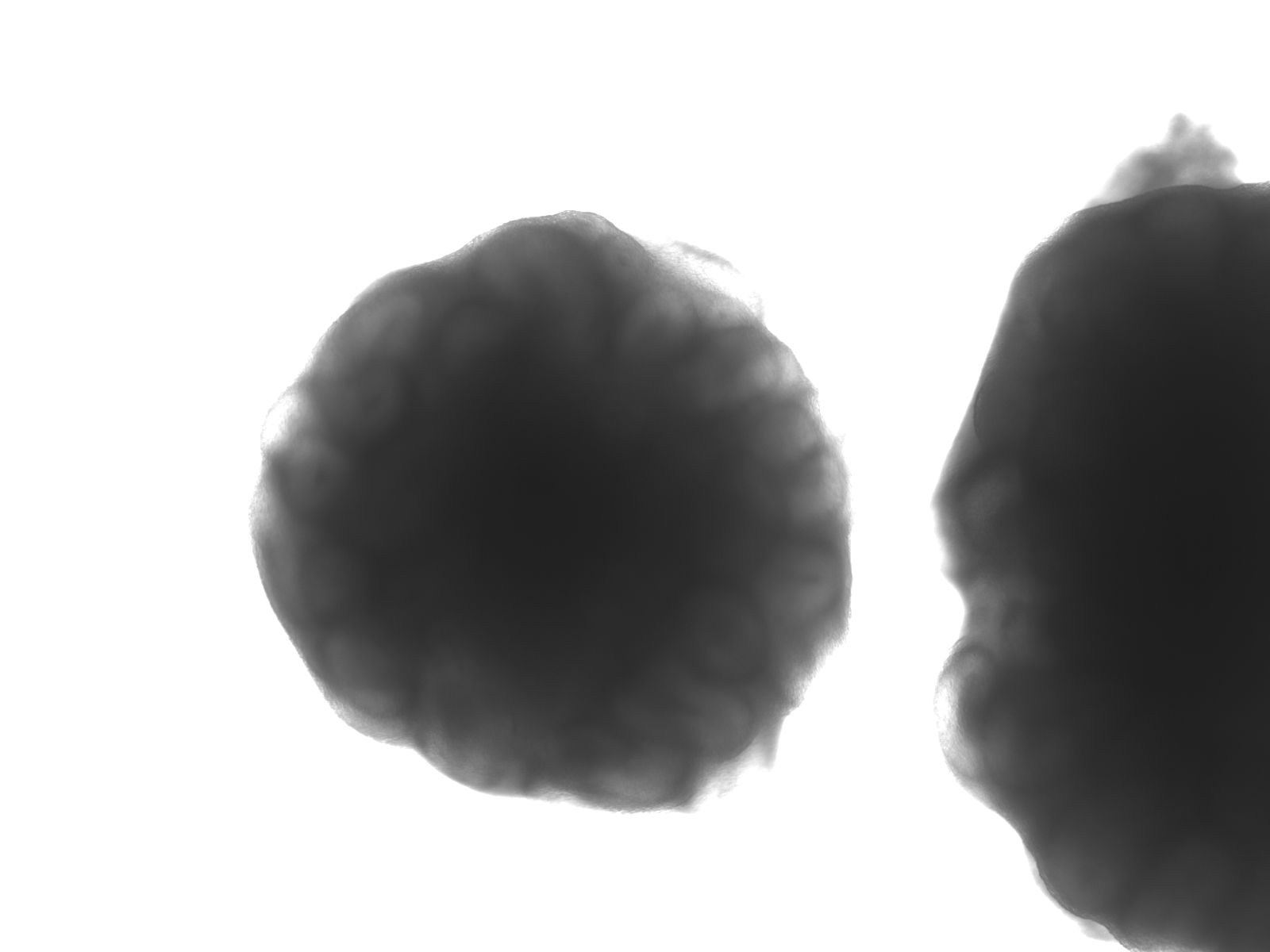

Supplement: Supplementary file 2 — Source Data for Figure 1 [file EMBJ-42-e113213-s003.zip › Figure1/Fig1B/Fig1B_H9_D40_MGliq.jpg]

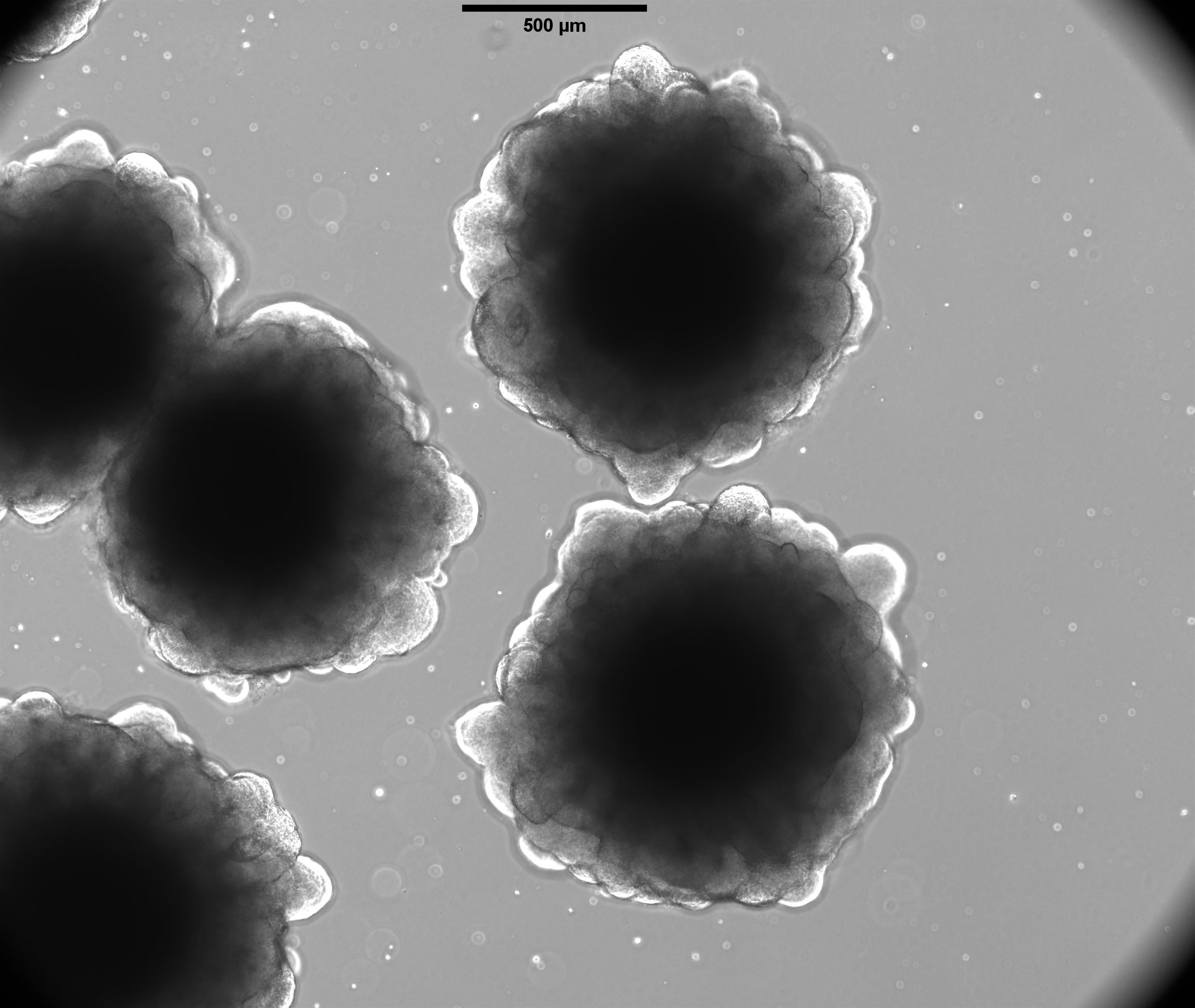

Supplement: Supplementary file 2 — Source Data for Figure 1 [file EMBJ-42-e113213-s003.zip › Figure1/Fig1B/Fig1B_H9_D16_MGliq.jpg]

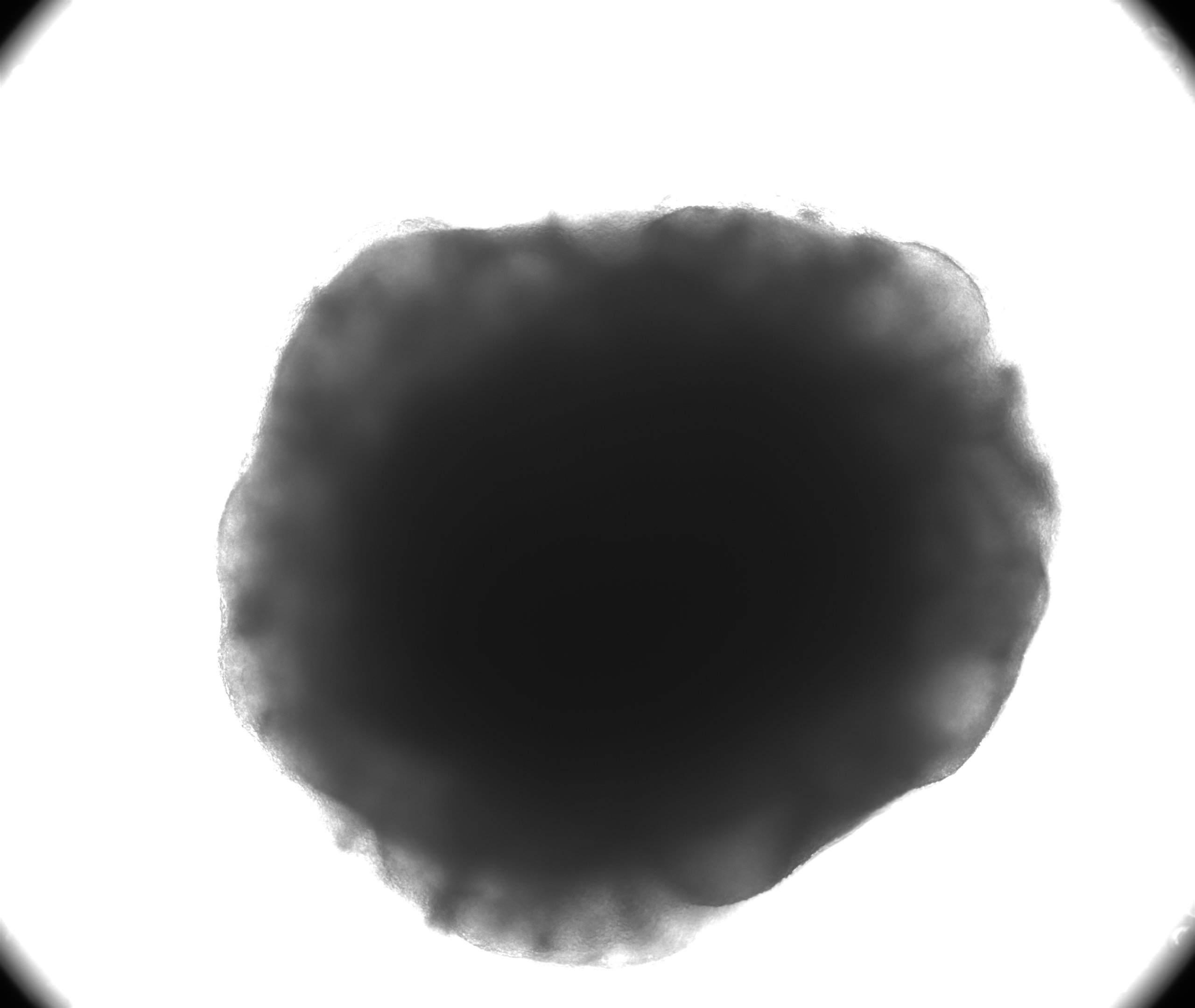

Supplement: Supplementary file 2 — Source Data for Figure 1 [file EMBJ-42-e113213-s003.zip › Figure1/Fig1B/Fig1B_iPSC2_D40_MGliq.jpg]

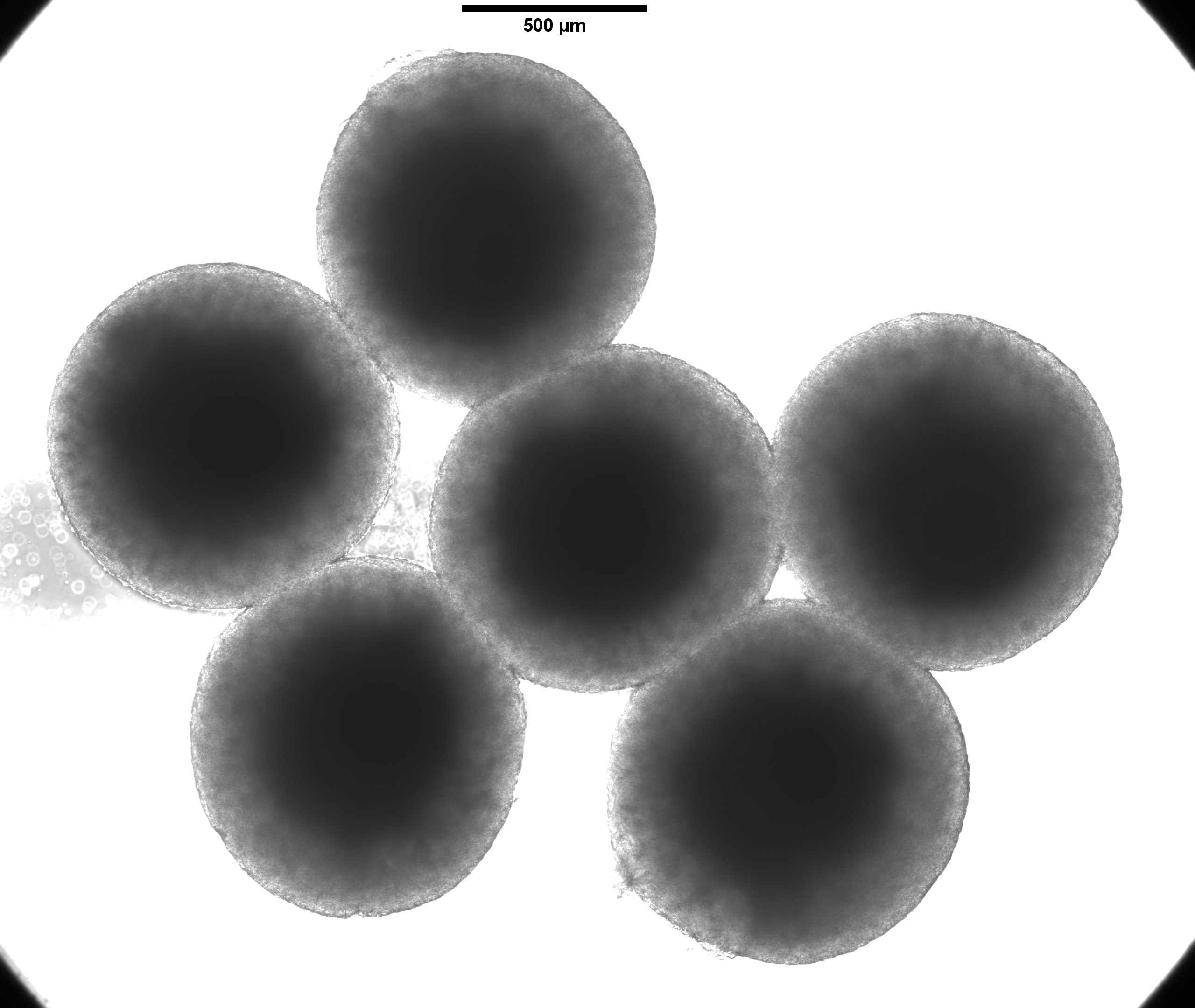

Supplement: Supplementary file 2 — Source Data for Figure 1 [file EMBJ-42-e113213-s003.zip › Figure1/Fig1B/Fig1B_H9_D20_MGnull.jpg]

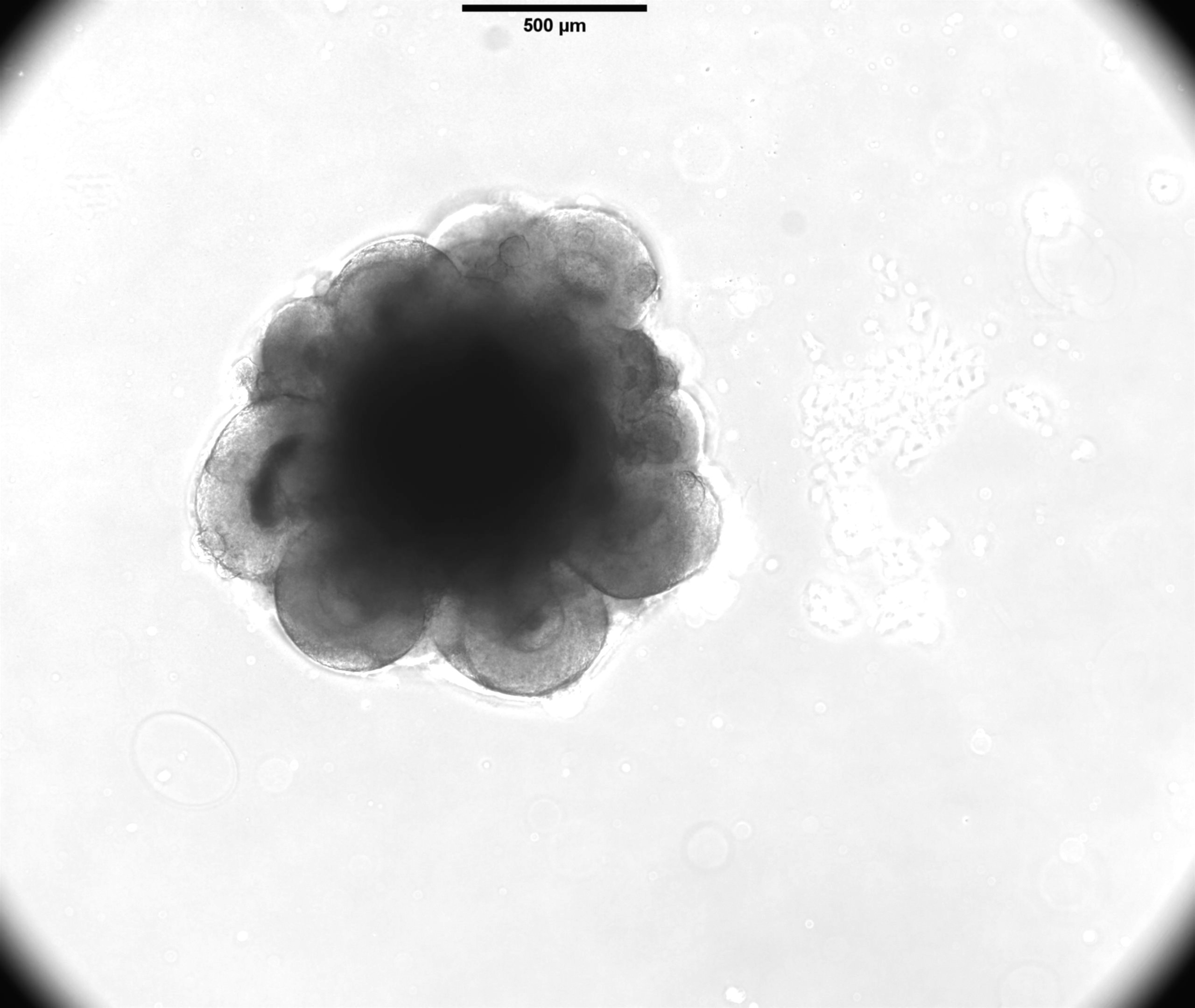

Supplement: Supplementary file 2 — Source Data for Figure 1 [file EMBJ-42-e113213-s003.zip › Figure1/Fig1B/Fig1B_H9_D20_MGdrop.jpg]

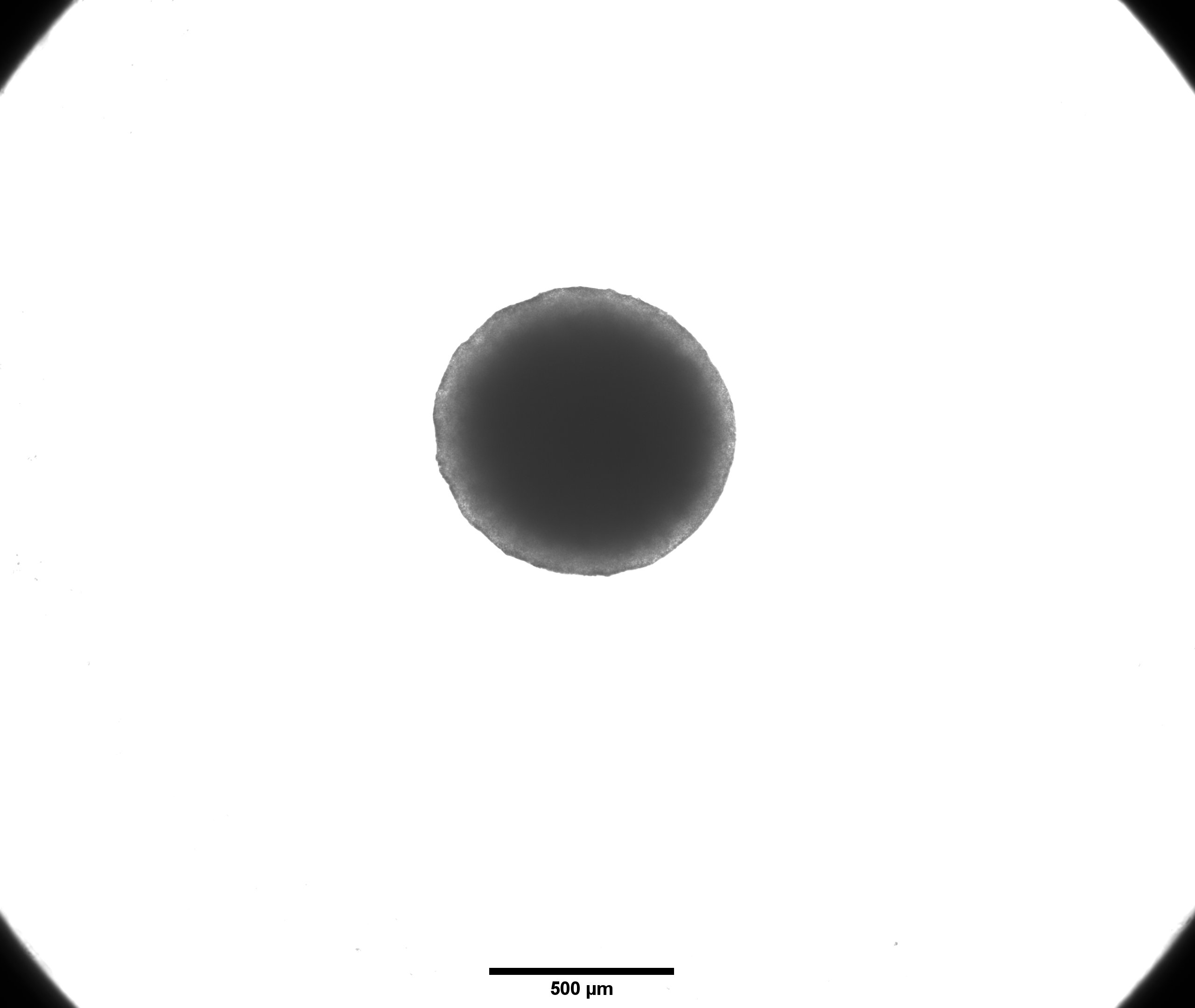

Supplement: Supplementary file 2 — Source Data for Figure 1 [file EMBJ-42-e113213-s003.zip › Figure1/Fig1B/Fig1B_H9_D10_MGnull.jpg]

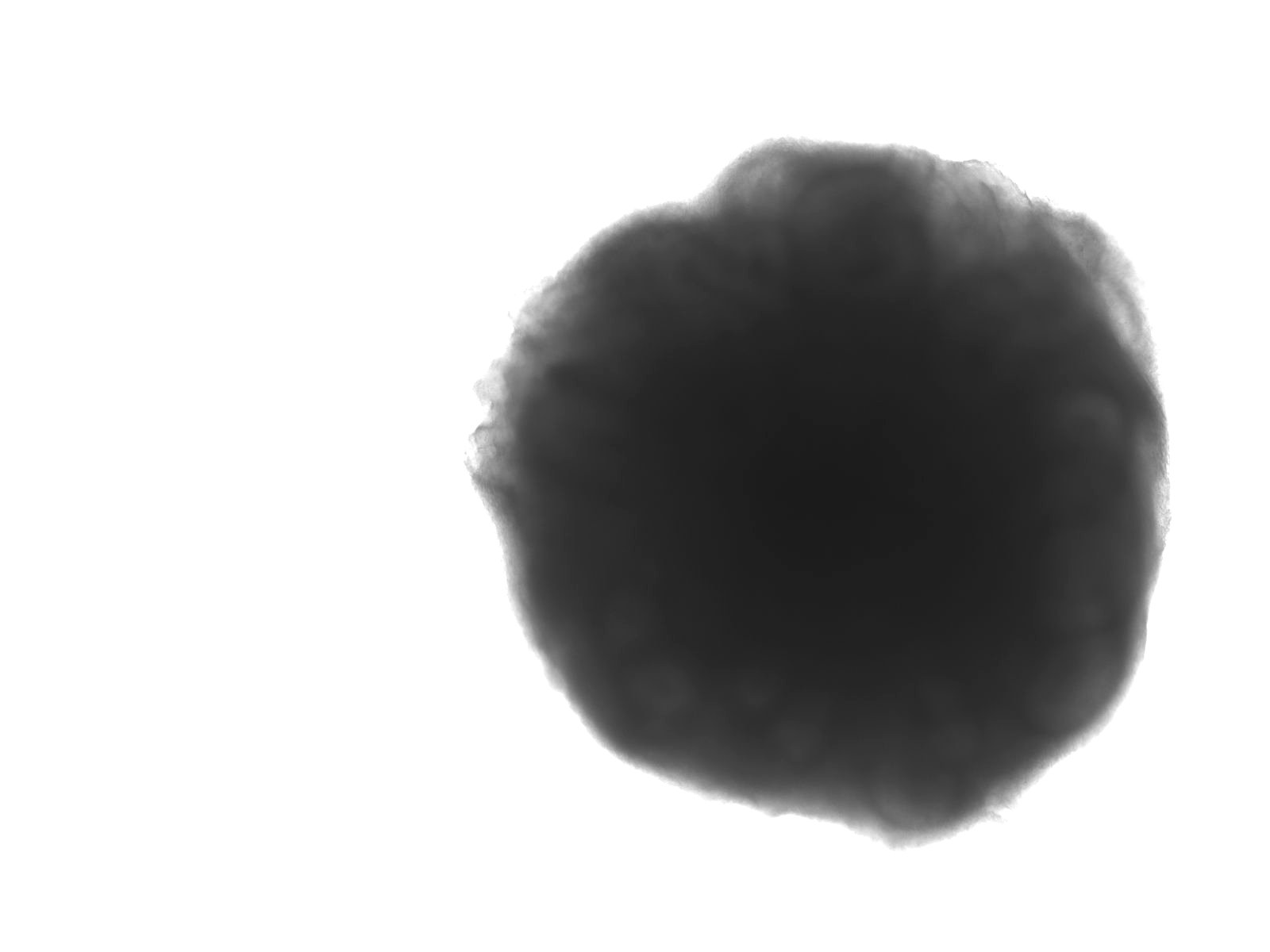

Supplement: Supplementary file 2 — Source Data for Figure 1 [file EMBJ-42-e113213-s003.zip › Figure1/Fig1B/Fig1B_H9_D40_MGdrop.jpg]

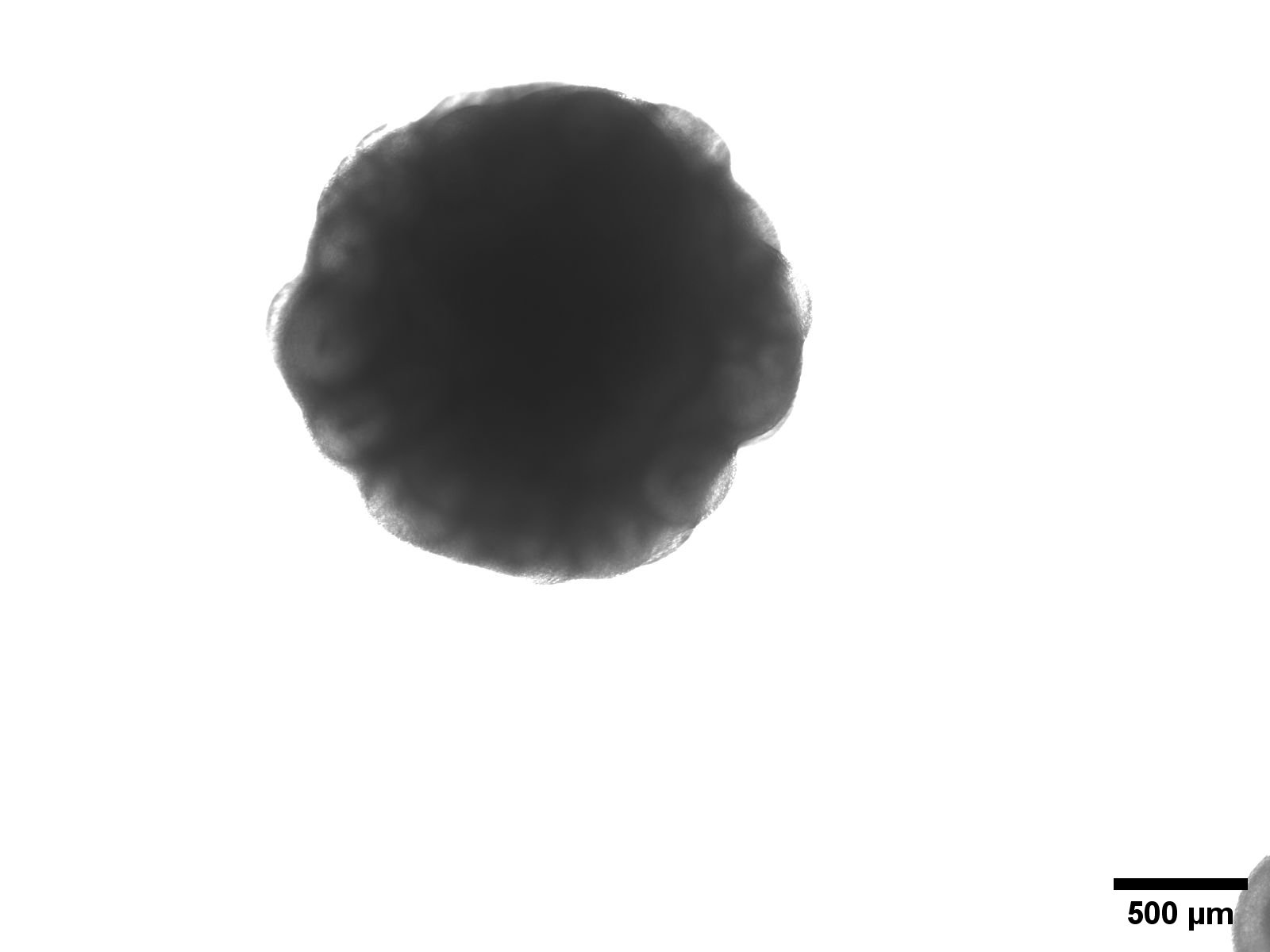

Supplement: Supplementary file 2 — Source Data for Figure 1 [file EMBJ-42-e113213-s003.zip › Figure1/Fig1B/Fig1B_H9_D40_MGnull.jpg]

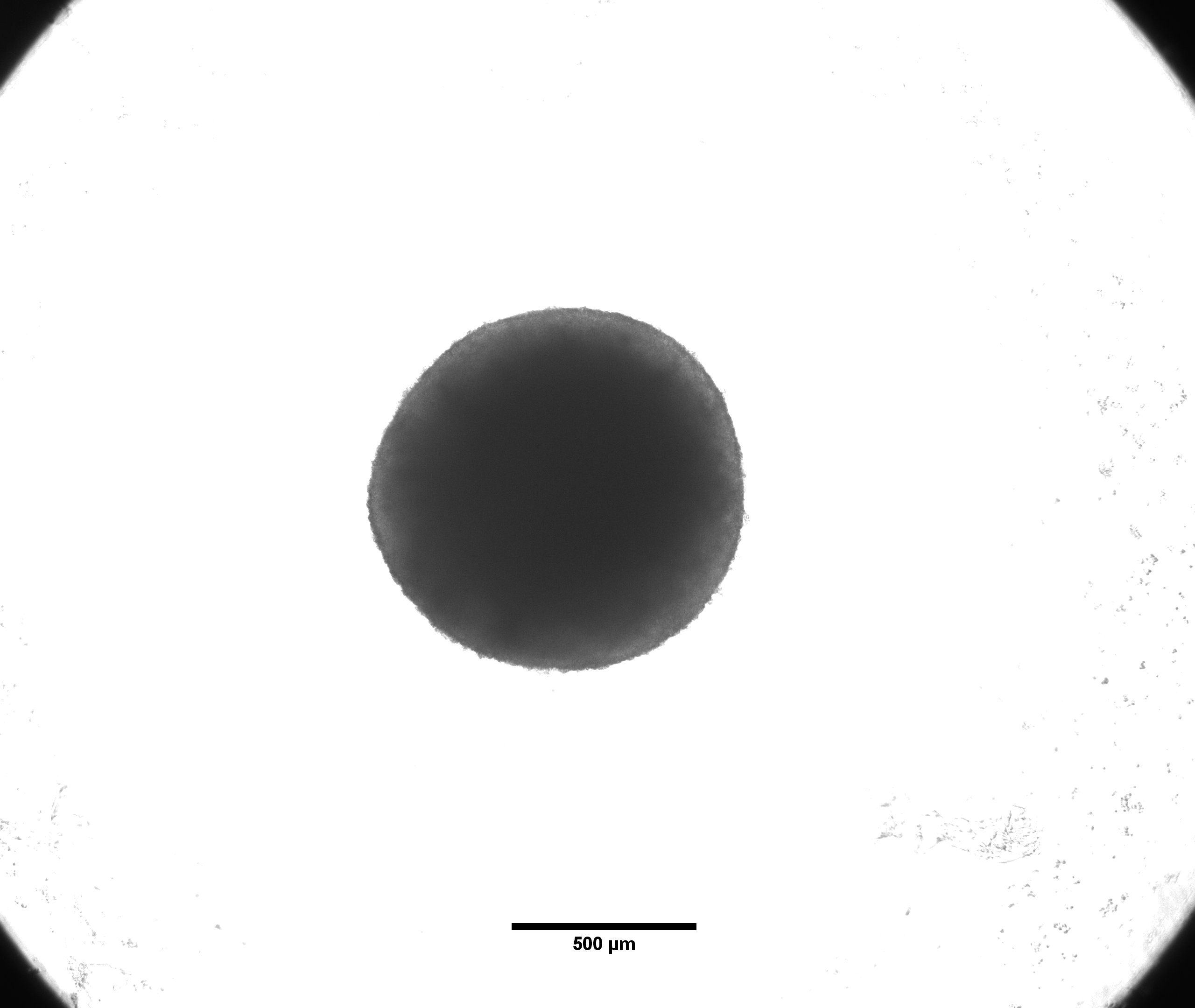

Supplement: Supplementary file 2 — Source Data for Figure 1 [file EMBJ-42-e113213-s003.zip › Figure1/Fig1B/Fig1B_iPSC1_D20_MGnull.jpg]

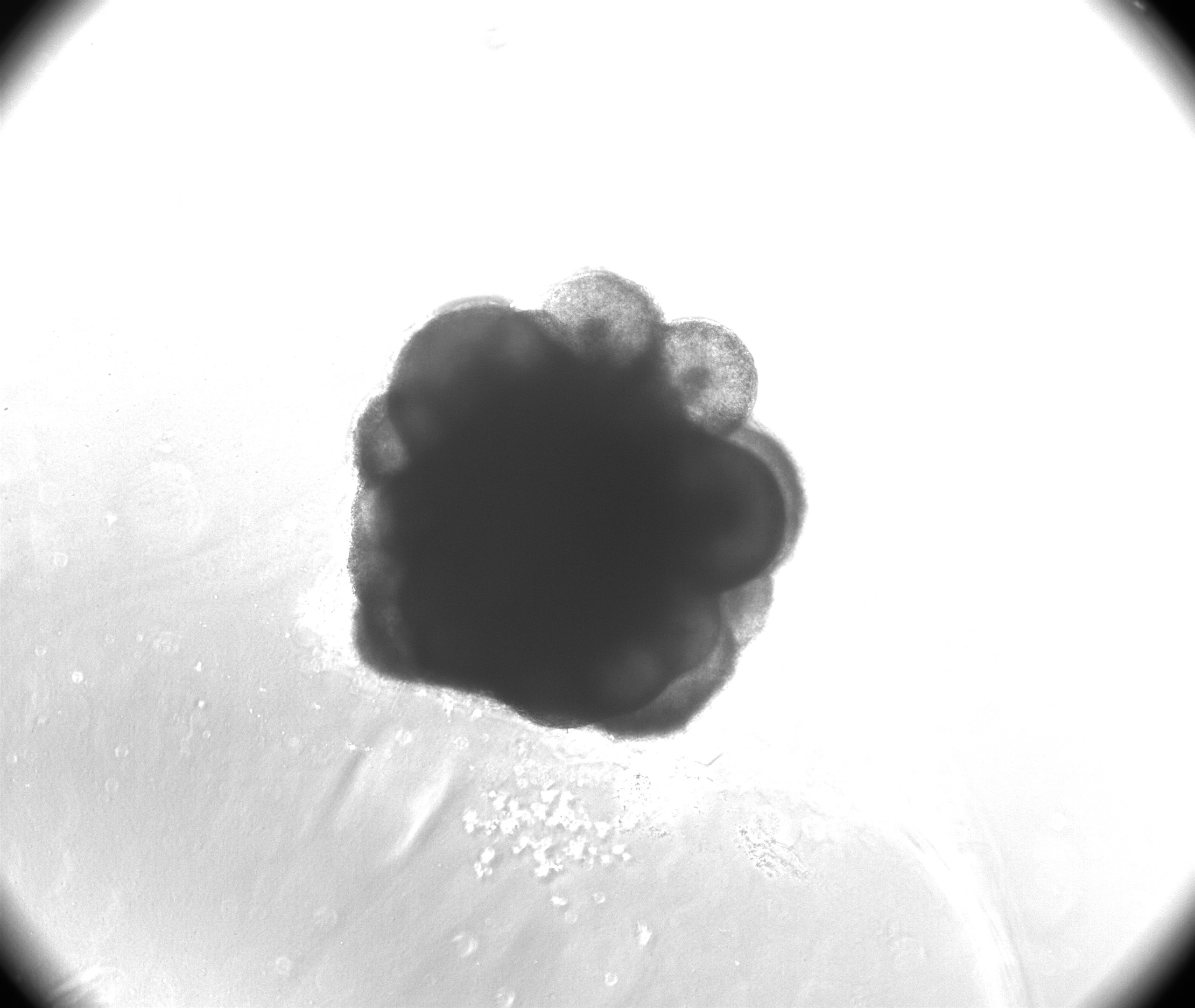

Supplement: Supplementary file 2 — Source Data for Figure 1 [file EMBJ-42-e113213-s003.zip › Figure1/Fig1B/Fig1B_iPSC1_D20_MGdrop.jpg]

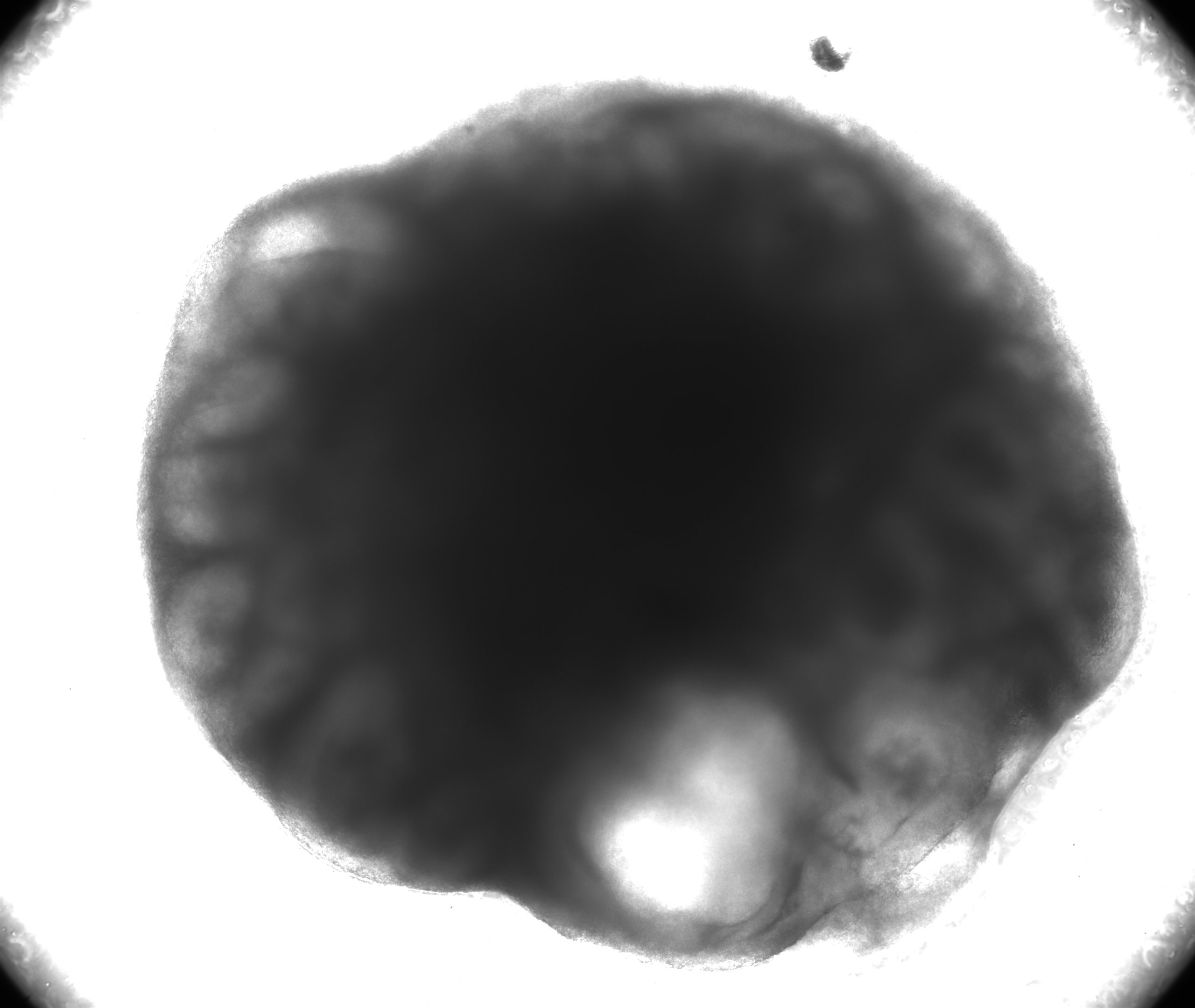

Supplement: Supplementary file 2 — Source Data for Figure 1 [file EMBJ-42-e113213-s003.zip › Figure1/Fig1B/Fig1B_iPSC2_D40_MGdrop.jpg]

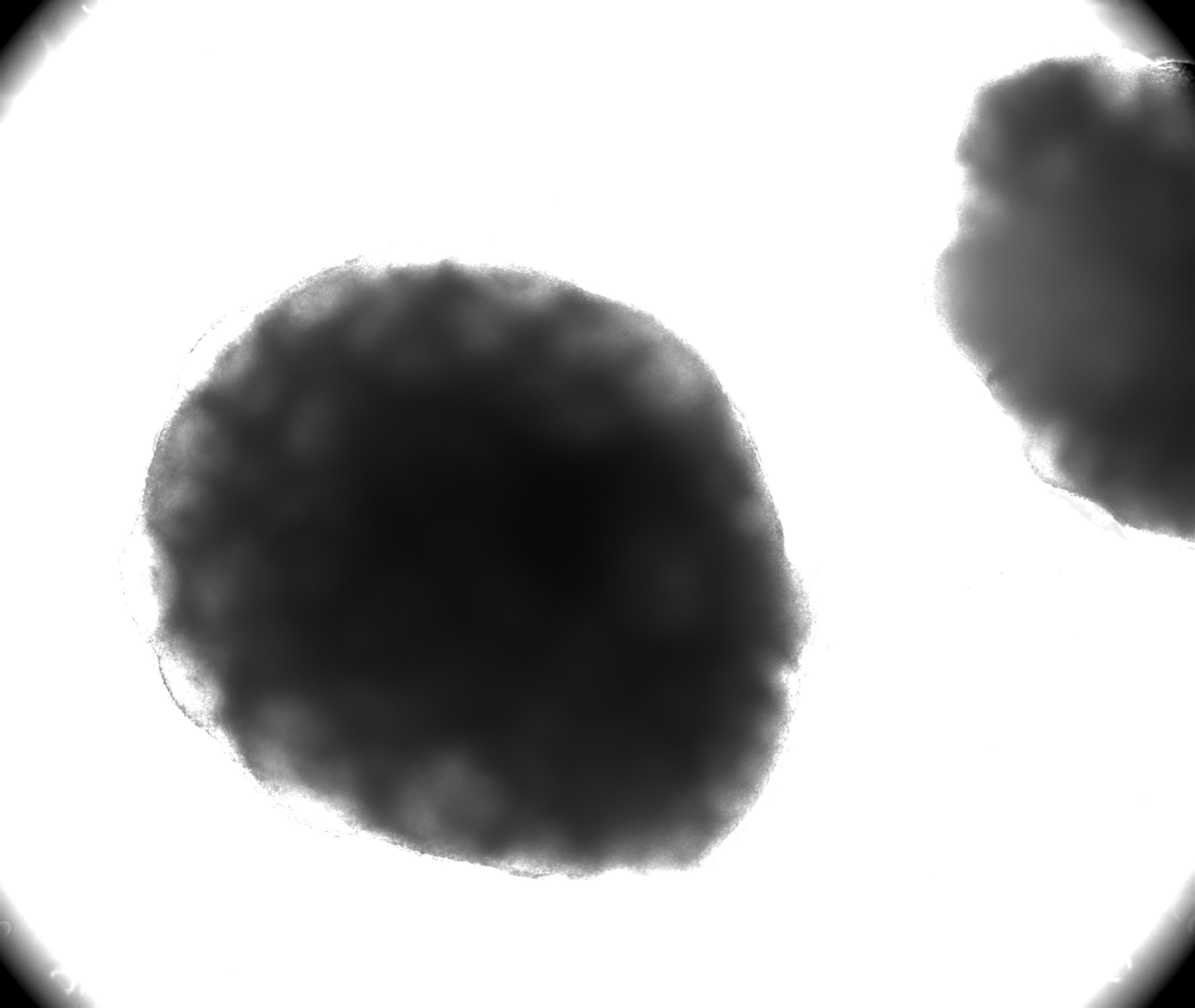

Supplement: Supplementary file 2 — Source Data for Figure 1 [file EMBJ-42-e113213-s003.zip › Figure1/Fig1B/Fig1B_iPSC2_D40_MGnull.jpg]

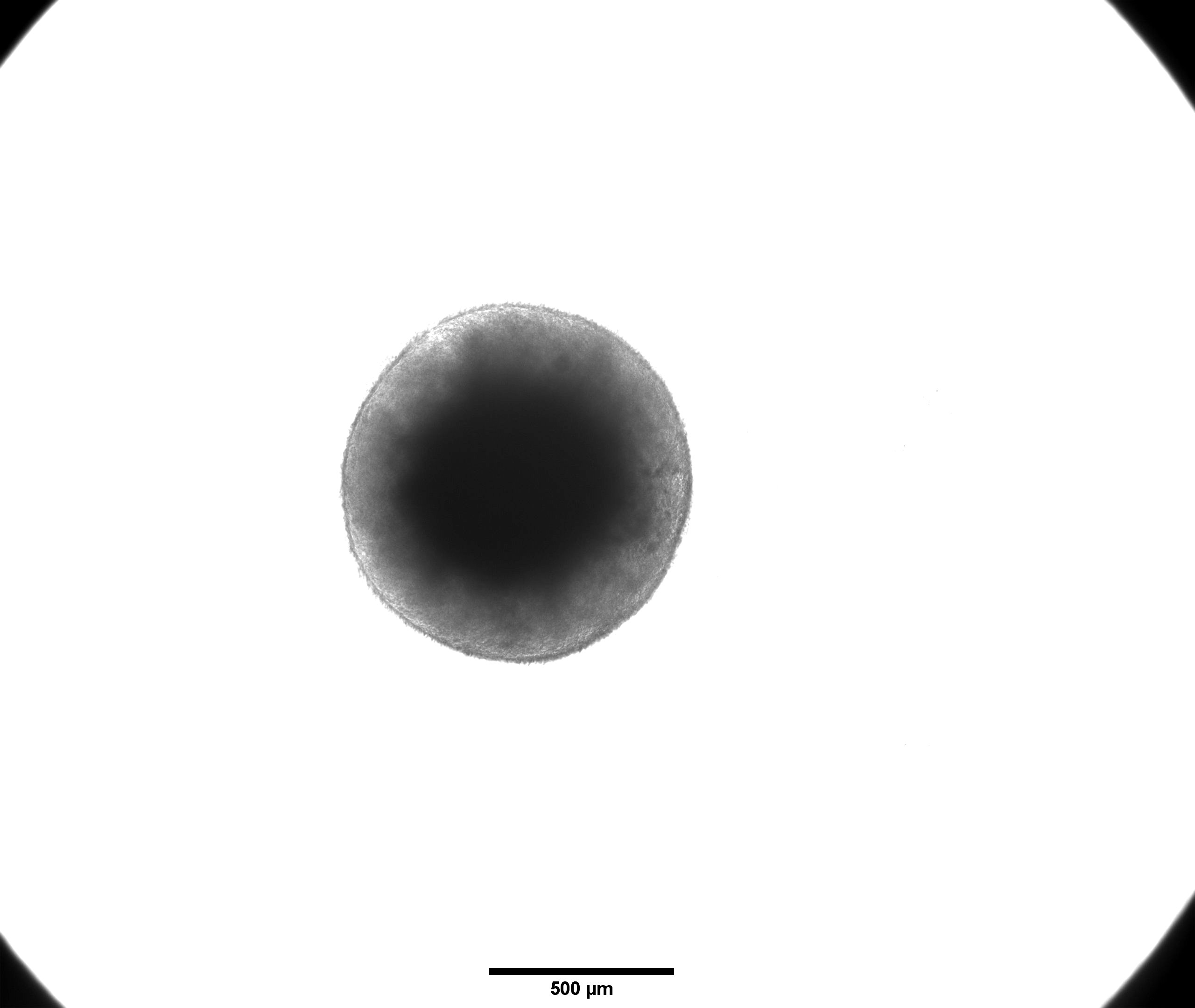

Supplement: Supplementary file 2 — Source Data for Figure 1 [file EMBJ-42-e113213-s003.zip › Figure1/Fig1B/Fig1B_H9_D16_MGnull.jpg]

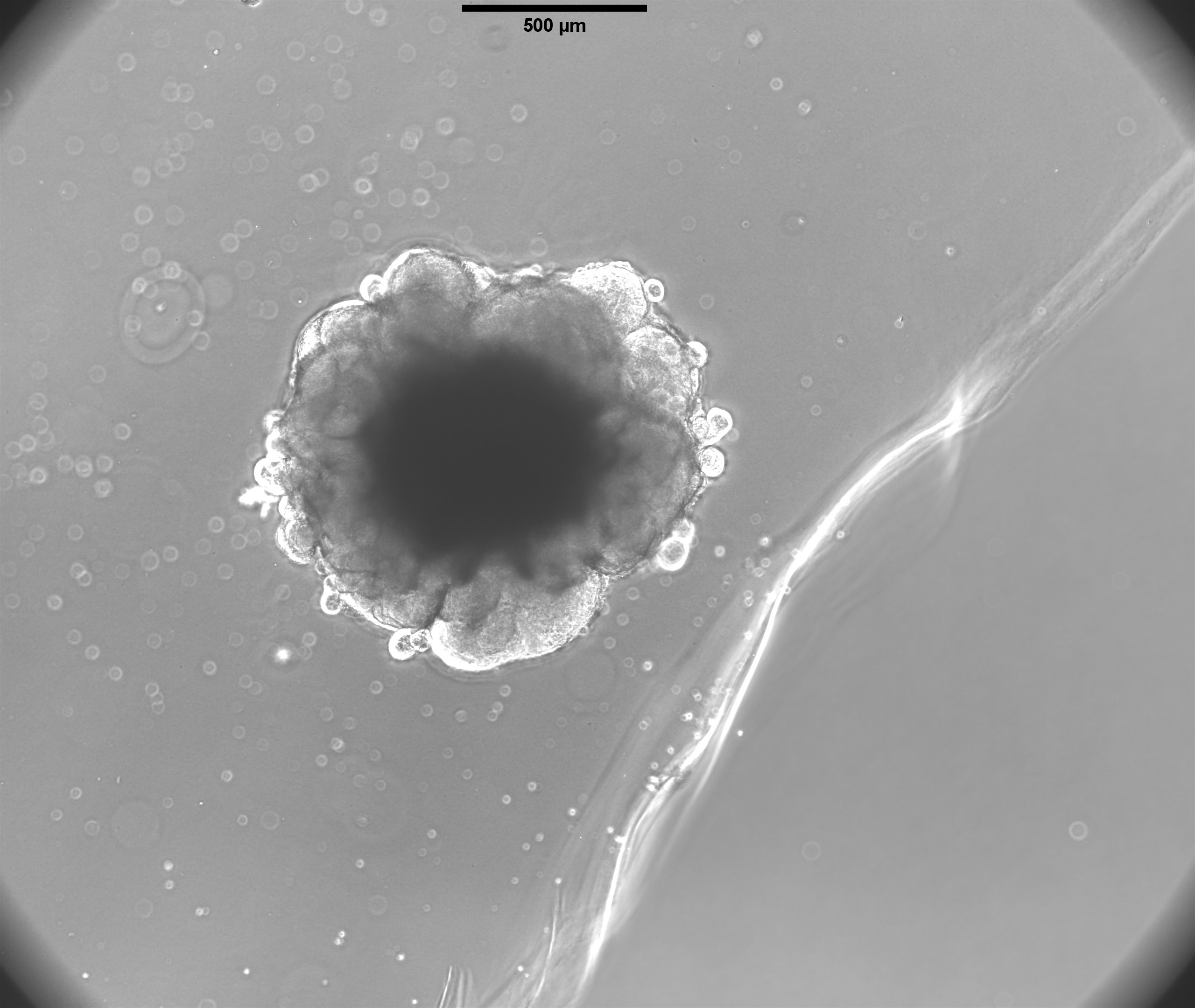

Supplement: Supplementary file 2 — Source Data for Figure 1 [file EMBJ-42-e113213-s003.zip › Figure1/Fig1B/Fig1B_H9_D16_MGdrop.jpg]

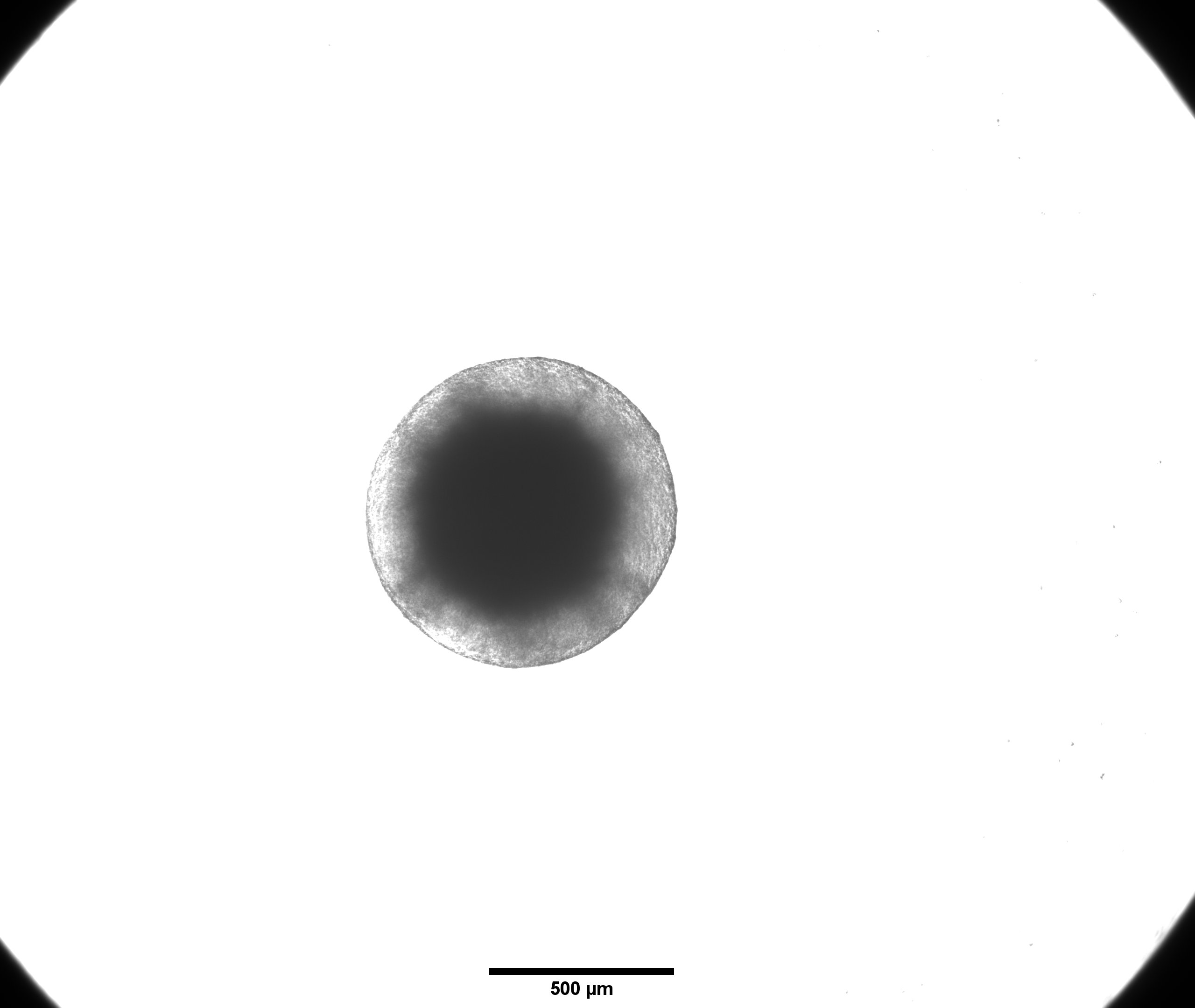

Supplement: Supplementary file 2 — Source Data for Figure 1 [file EMBJ-42-e113213-s003.zip › Figure1/Fig1B/Fig1B_H9_D13_MGnull.jpg]

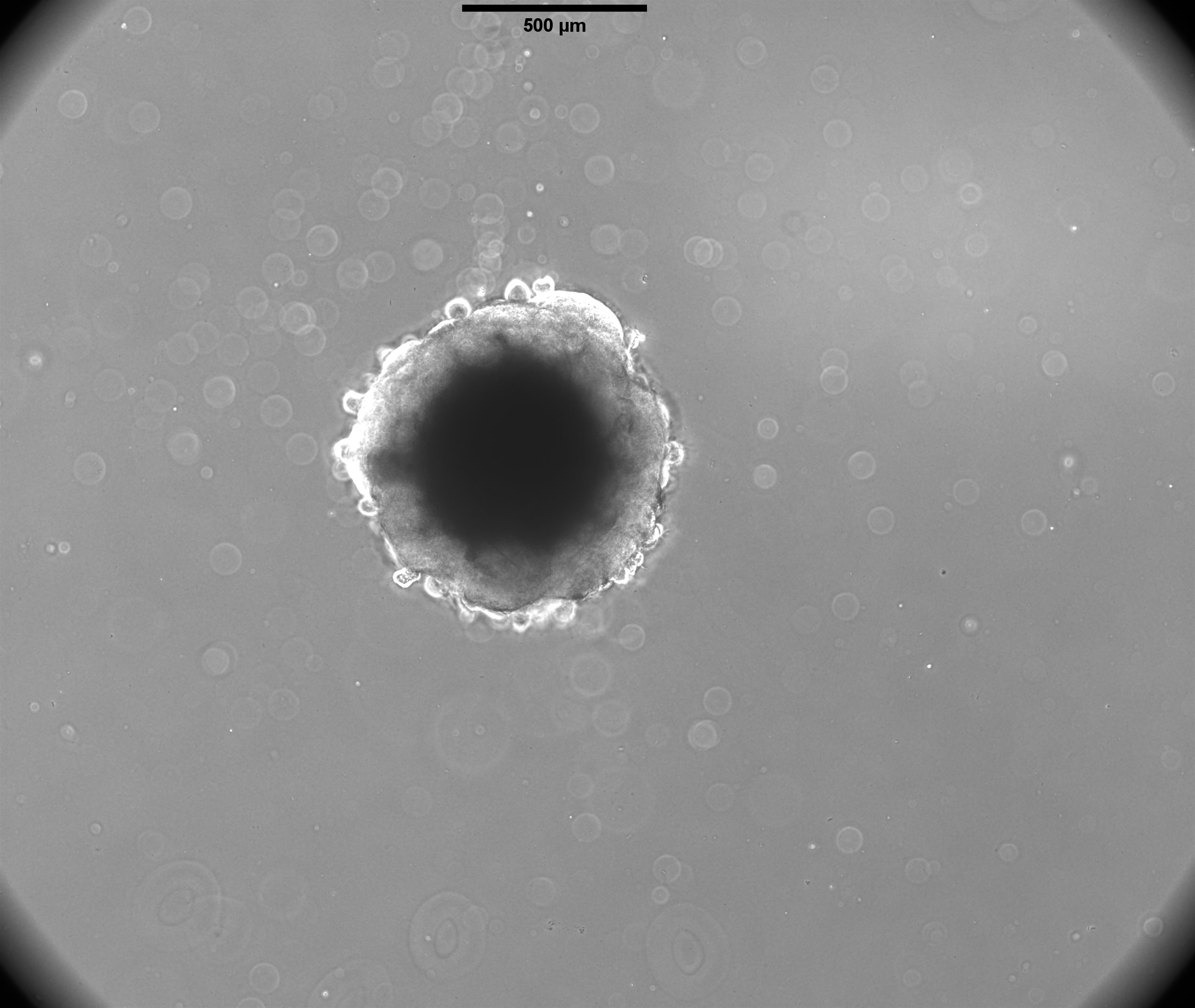

Supplement: Supplementary file 2 — Source Data for Figure 1 [file EMBJ-42-e113213-s003.zip › Figure1/Fig1B/Fig1B_H9_D13_MGdrop.jpg]

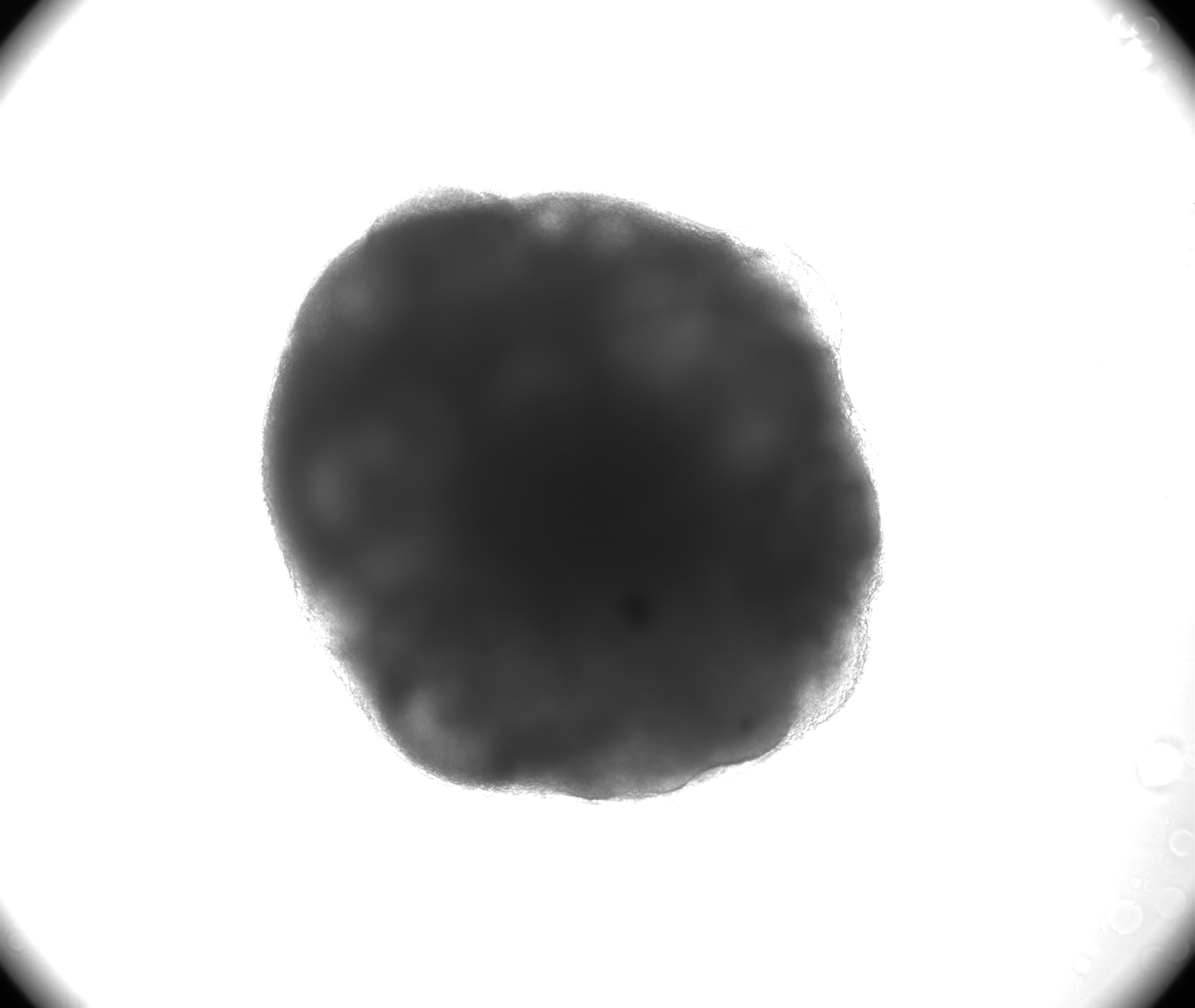

Supplement: Supplementary file 2 — Source Data for Figure 1 [file EMBJ-42-e113213-s003.zip › Figure1/Fig1B/Fig1B_iPSC3_D40_MGnull.jpg]

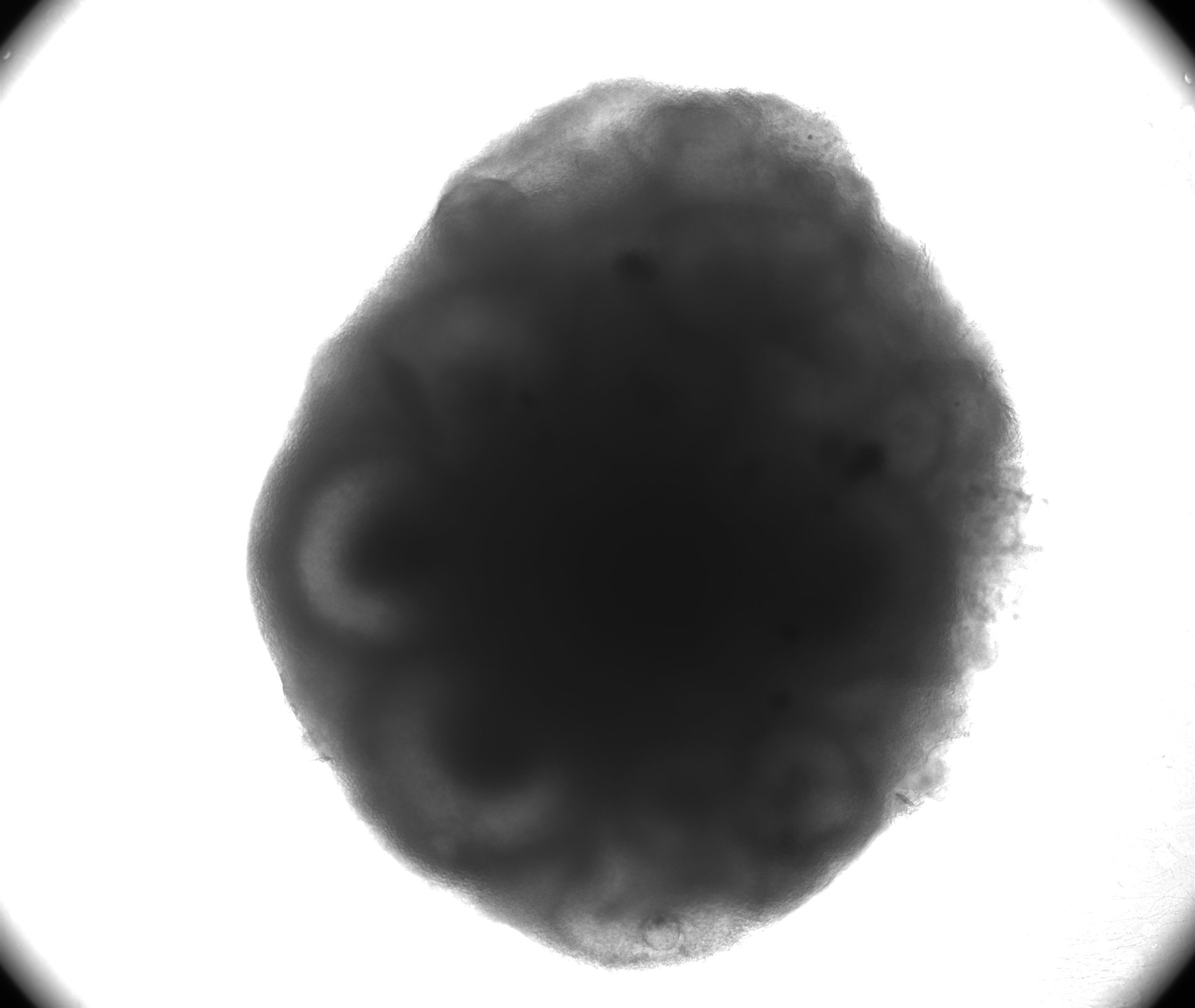

Supplement: Supplementary file 2 — Source Data for Figure 1 [file EMBJ-42-e113213-s003.zip › Figure1/Fig1B/Fig1B_iPSC3_D40_MGdrop.jpg]

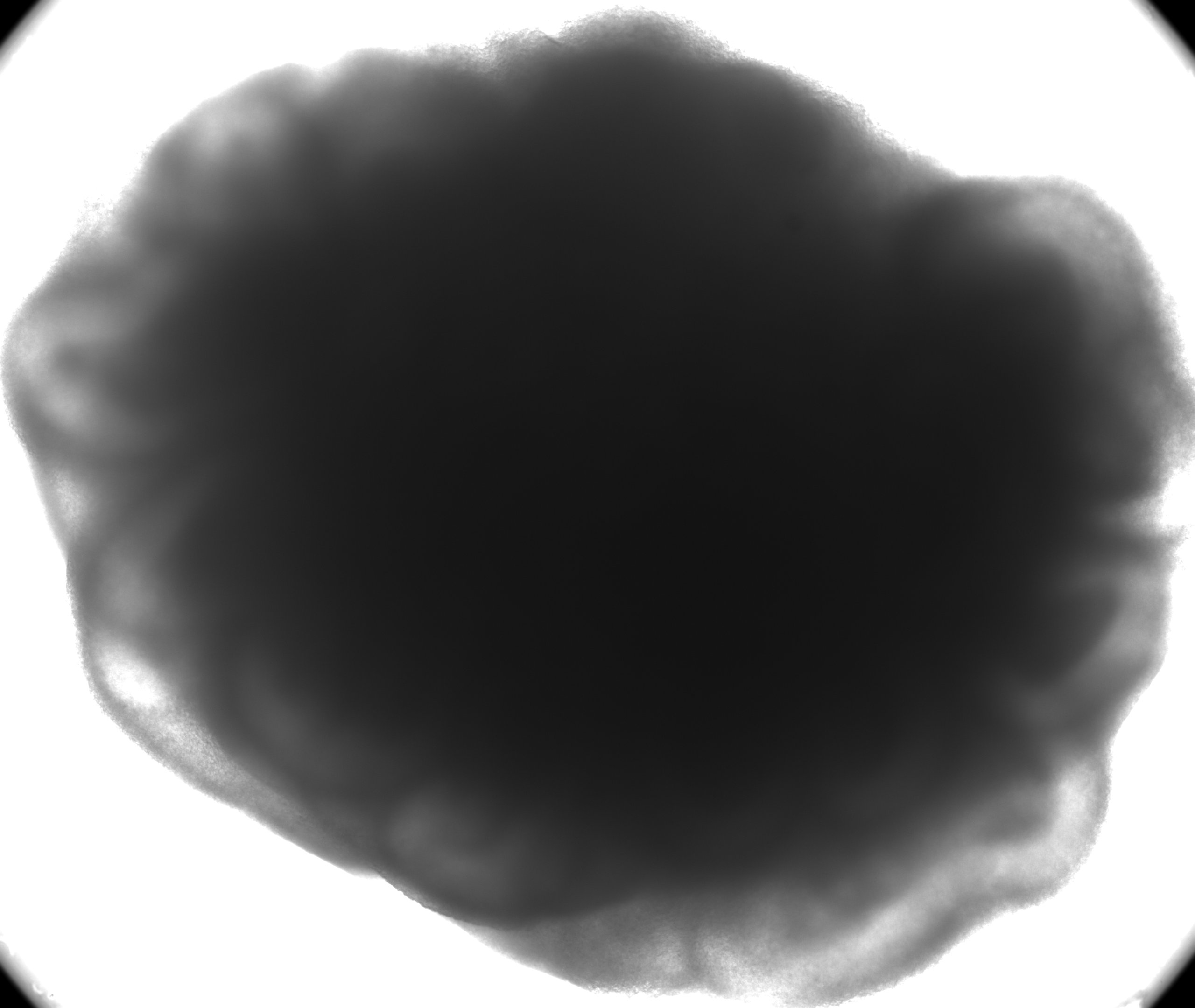

Supplement: Supplementary file 2 — Source Data for Figure 1 [file EMBJ-42-e113213-s003.zip › Figure1/Fig1B/Fig1B_iPSC1_D40_MGdrop.jpg]

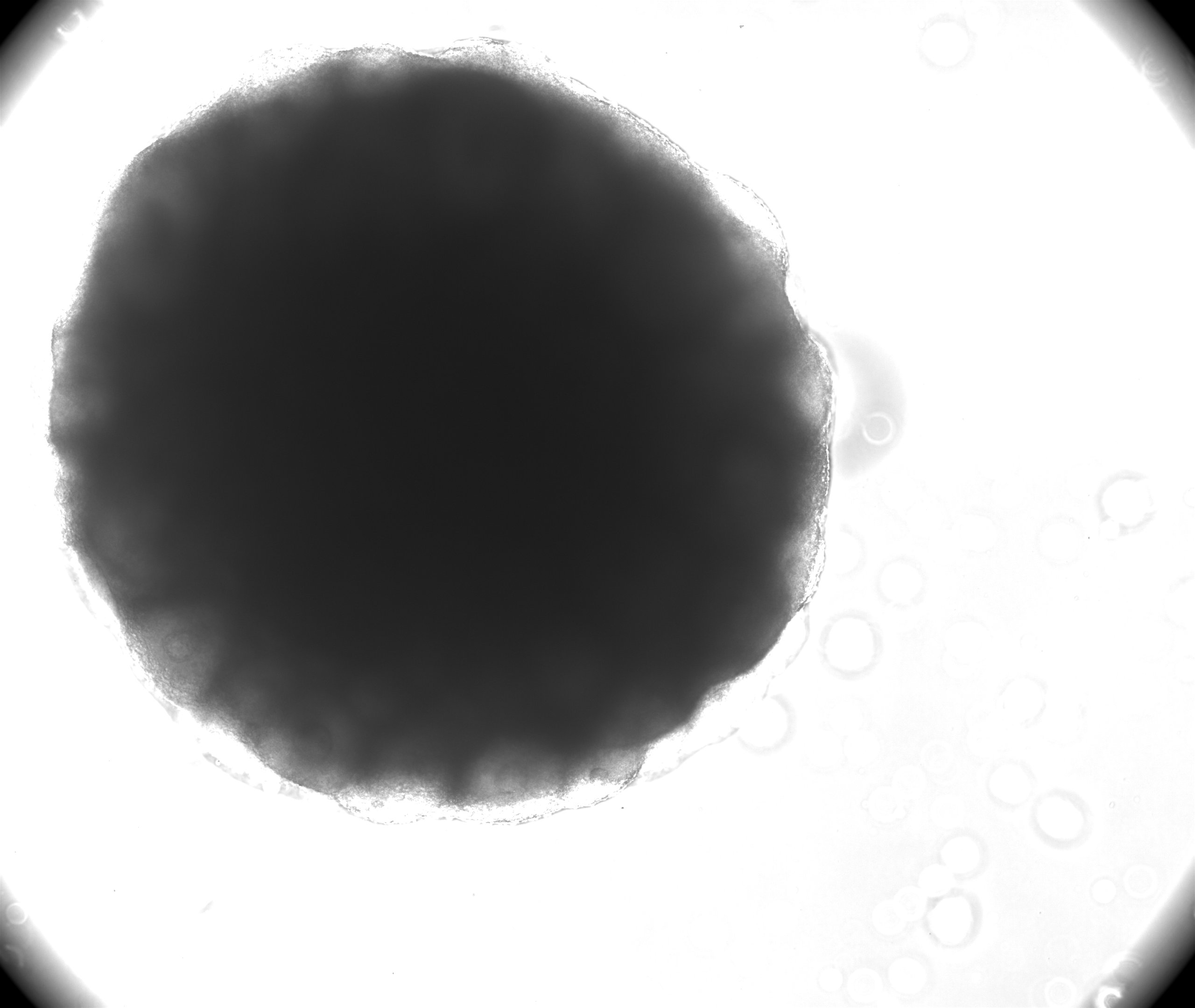

Supplement: Supplementary file 2 — Source Data for Figure 1 [file EMBJ-42-e113213-s003.zip › Figure1/Fig1B/Fig1B_iPSC1_D40_MGnull.jpg]

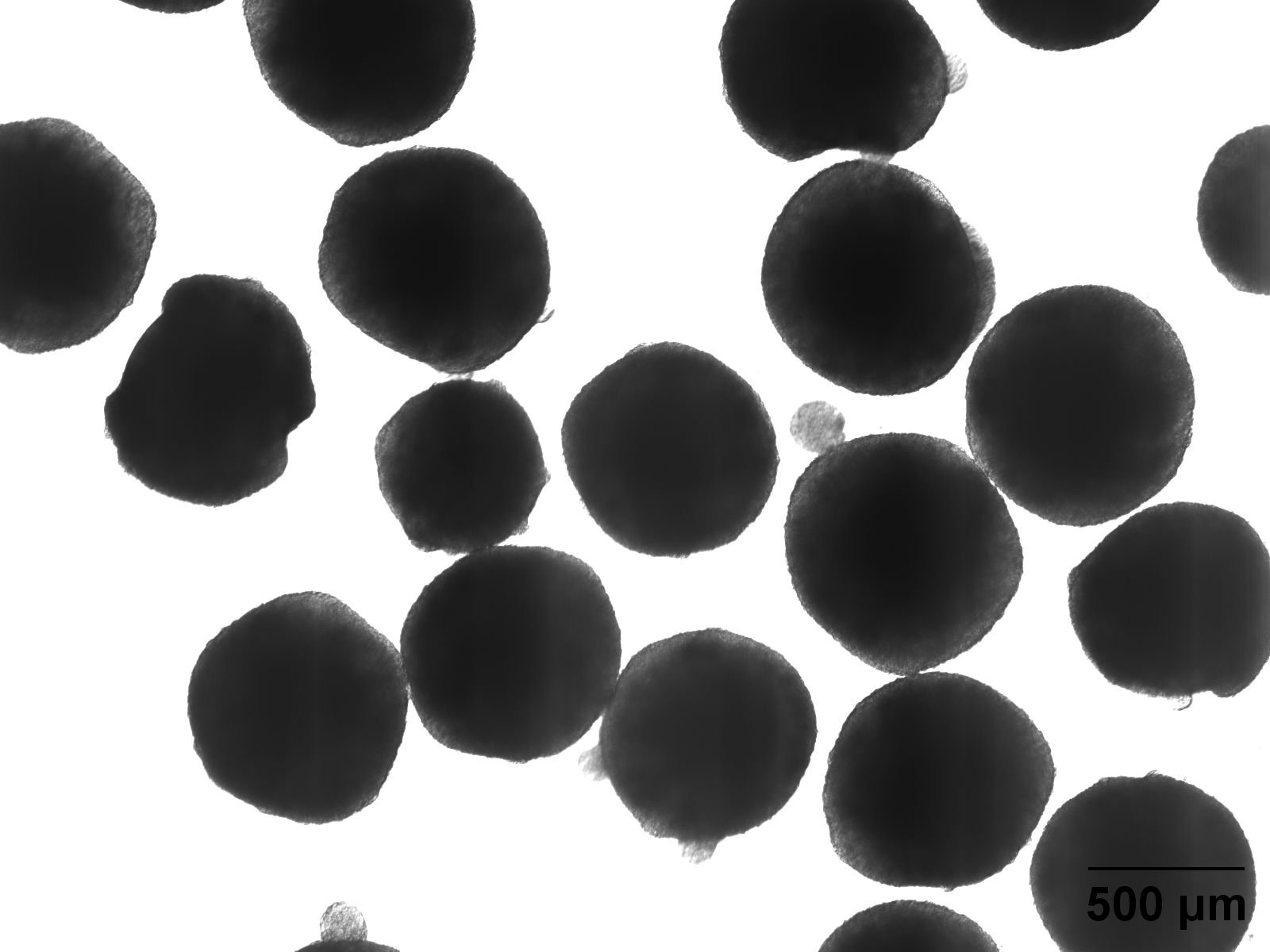

Supplement: Supplementary file 2 — Source Data for Figure 1 [file EMBJ-42-e113213-s003.zip › Figure1/Fig1B/Fig1B_iPSC2_D20_MGnull.jpg]

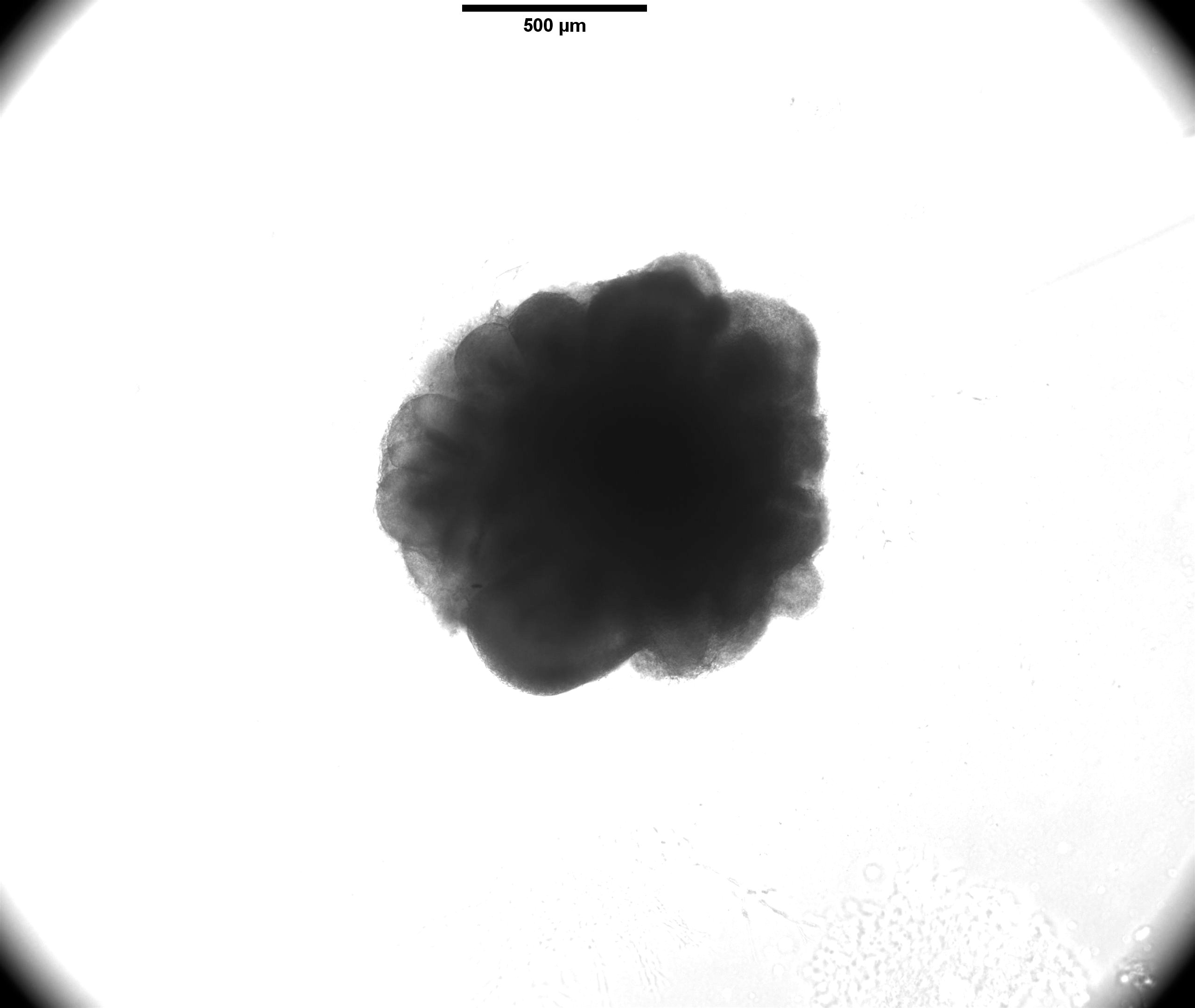

Supplement: Supplementary file 2 — Source Data for Figure 1 [file EMBJ-42-e113213-s003.zip › Figure1/Fig1B/Fig1B_iPSC2_D20_MGdrop.jpg]

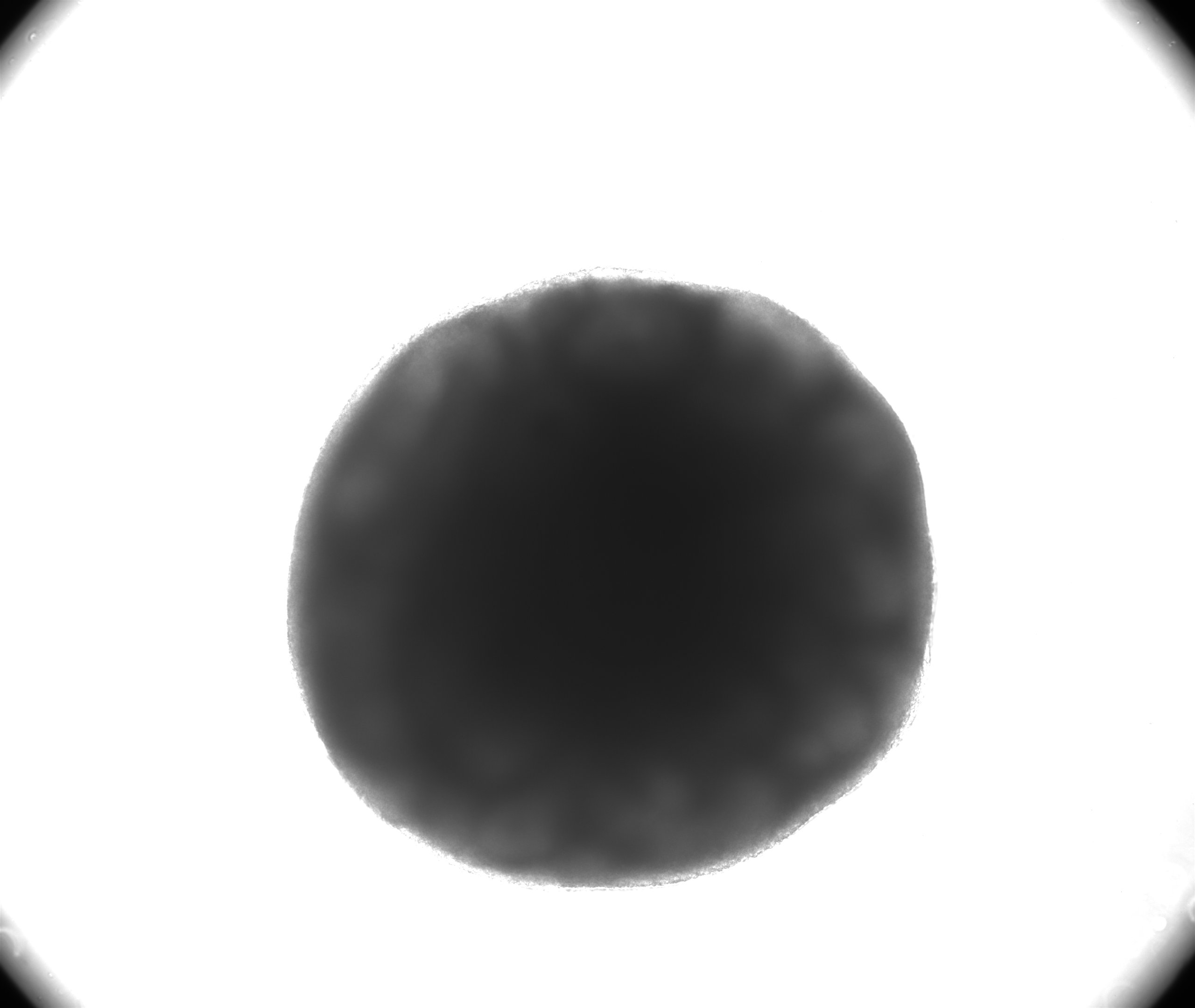

Supplement: Supplementary file 2 — Source Data for Figure 1 [file EMBJ-42-e113213-s003.zip › Figure1/Fig1B/Fig1B_iPSC3_D40_MGliq.jpg]

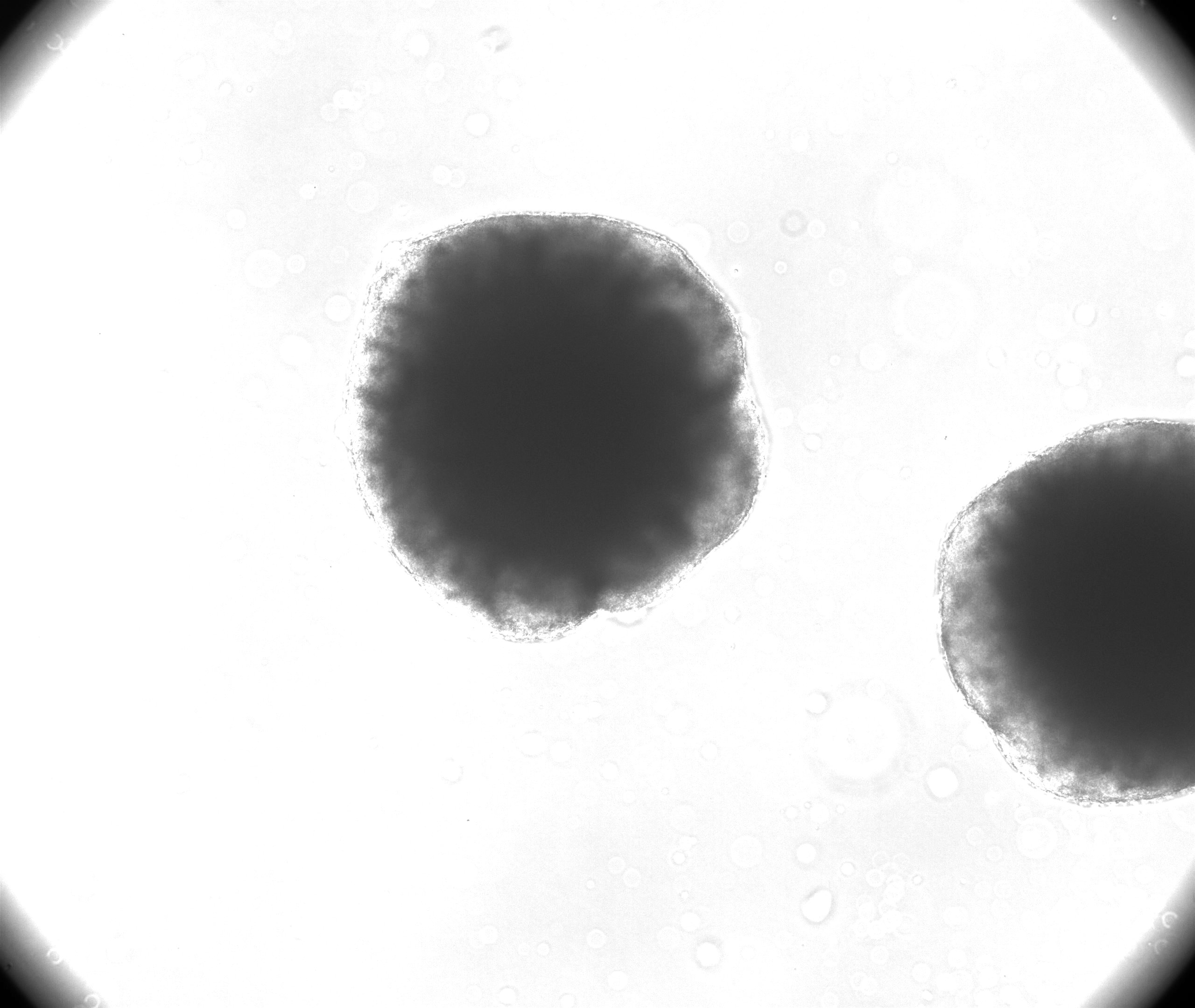

Supplement: Supplementary file 2 — Source Data for Figure 1 [file EMBJ-42-e113213-s003.zip › Figure1/Fig1B/Fig1B_iPSC1_D20_MGliq.jpg]

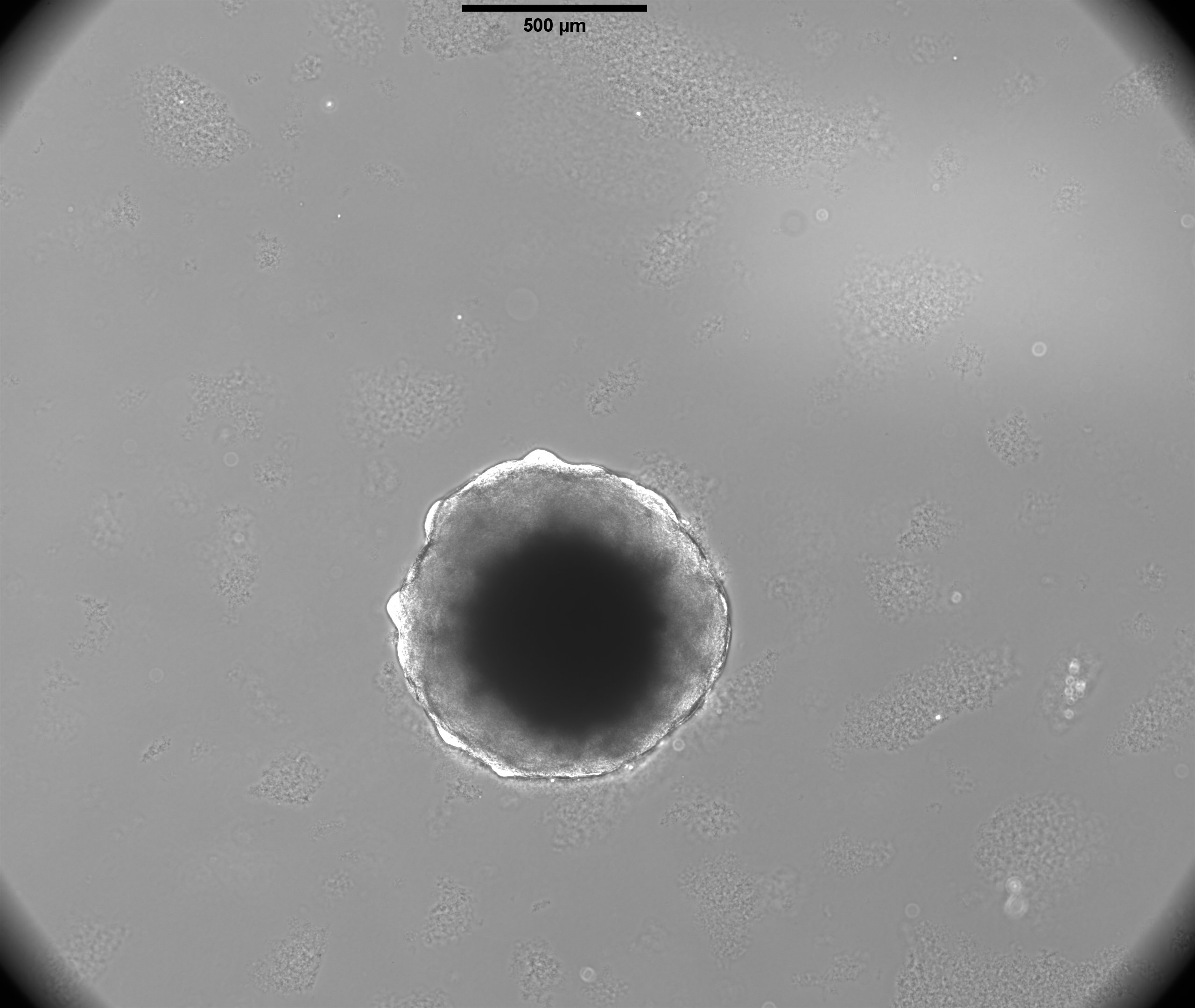

Supplement: Supplementary file 2 — Source Data for Figure 1 [file EMBJ-42-e113213-s003.zip › Figure1/Fig1B/Fig1B_H9_D13_MGliq.jpg]

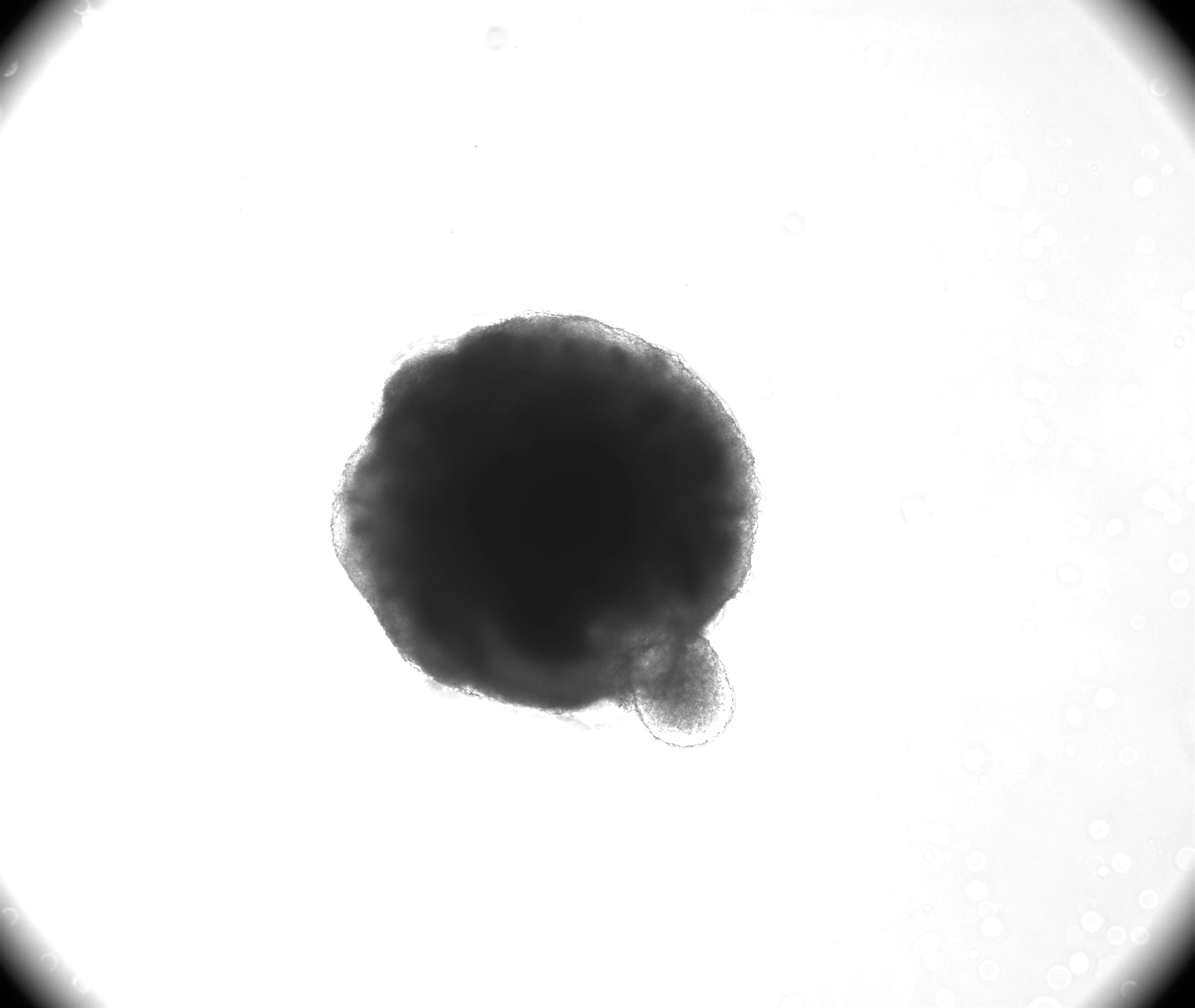

Supplement: Supplementary file 2 — Source Data for Figure 1 [file EMBJ-42-e113213-s003.zip › Figure1/Fig1B/Fig1B_iPSC2_D20_MGliq.jpg]

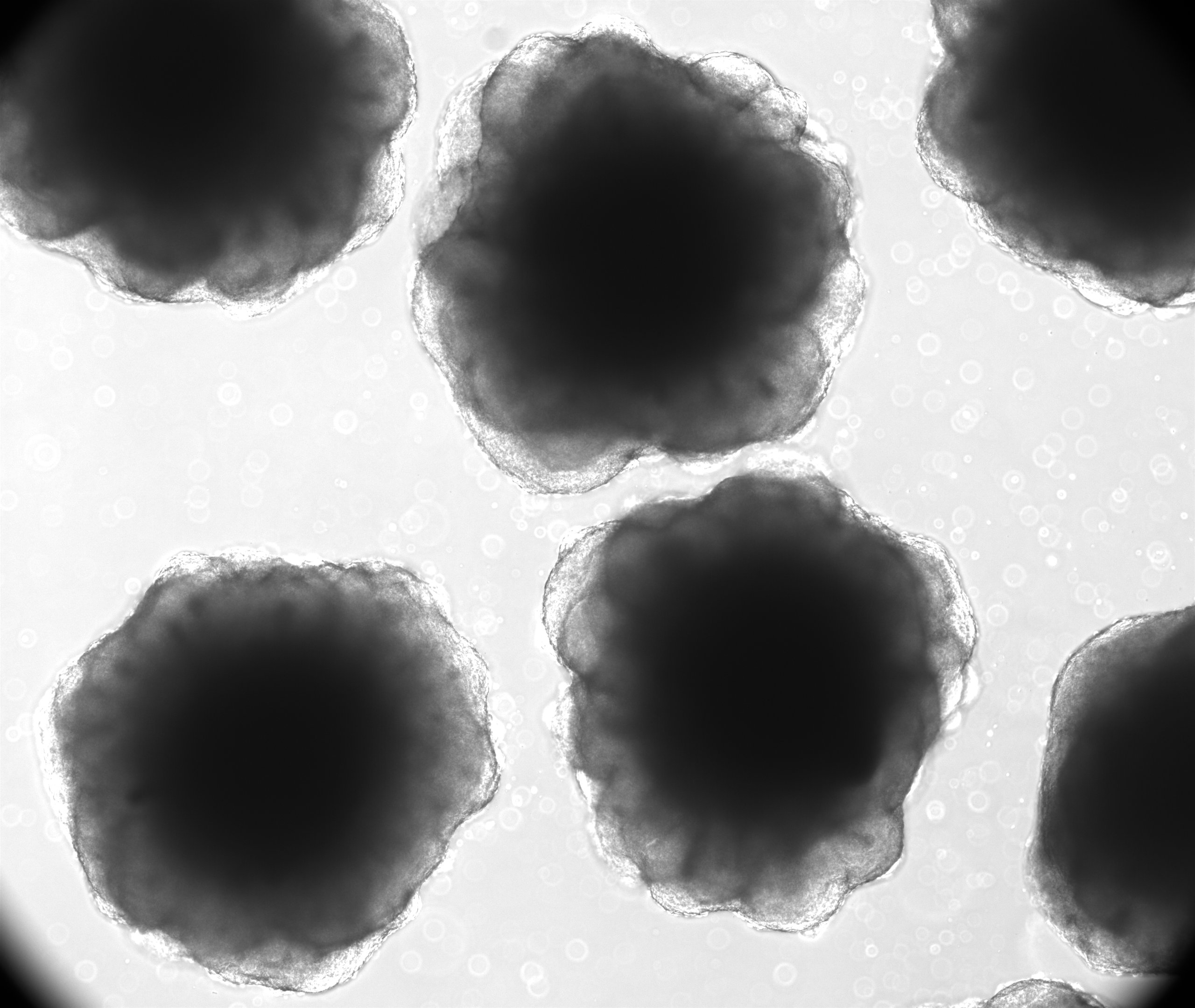

Supplement: Supplementary file 2 — Source Data for Figure 1 [file EMBJ-42-e113213-s003.zip › Figure1/Fig1B/Fig1B_H9_D20_MGliq.jpg]

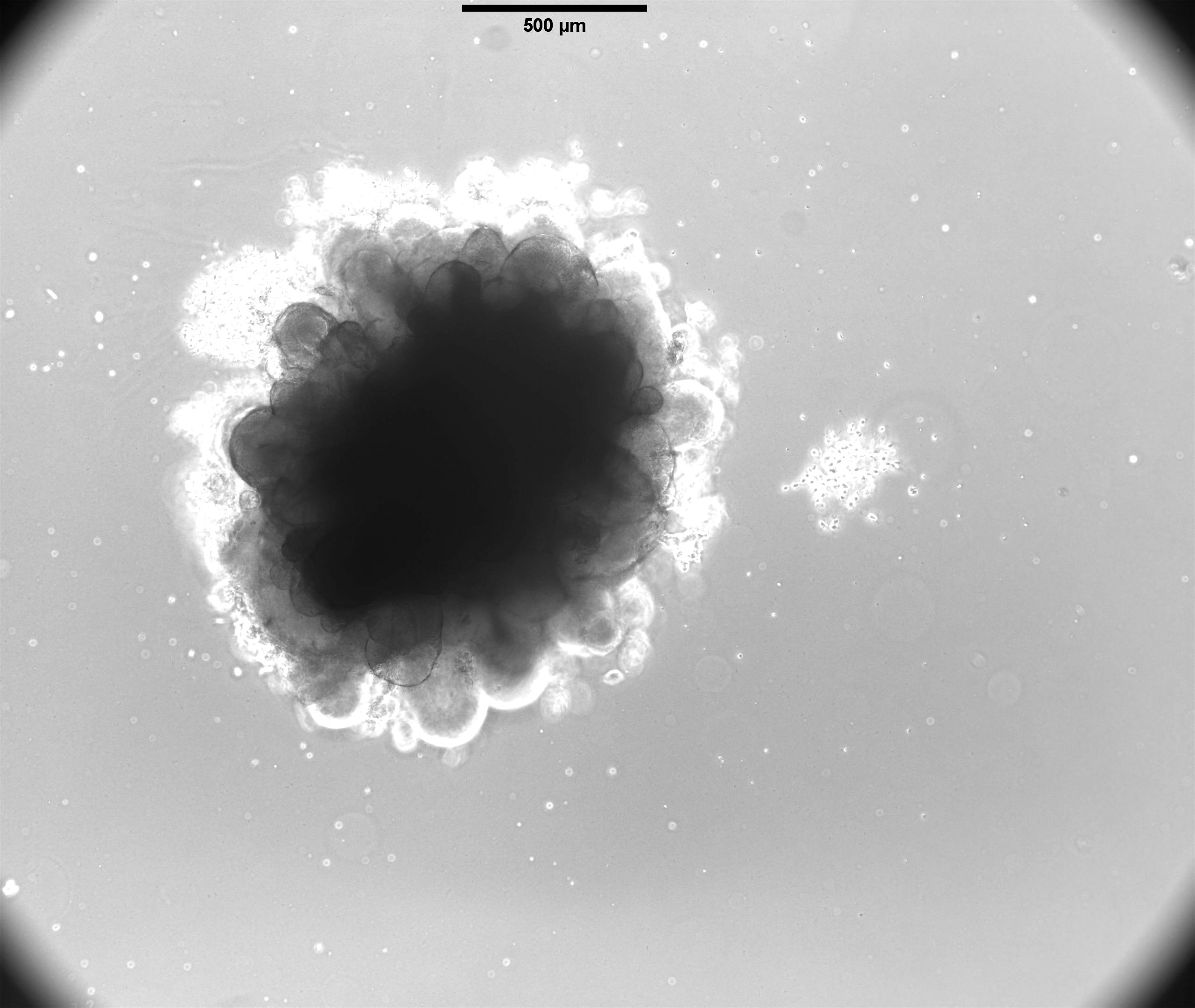

Supplement: Supplementary file 2 — Source Data for Figure 1 [file EMBJ-42-e113213-s003.zip › Figure1/Fig1B/Fig1B_iPSC3_D20_MGdrop.jpg]

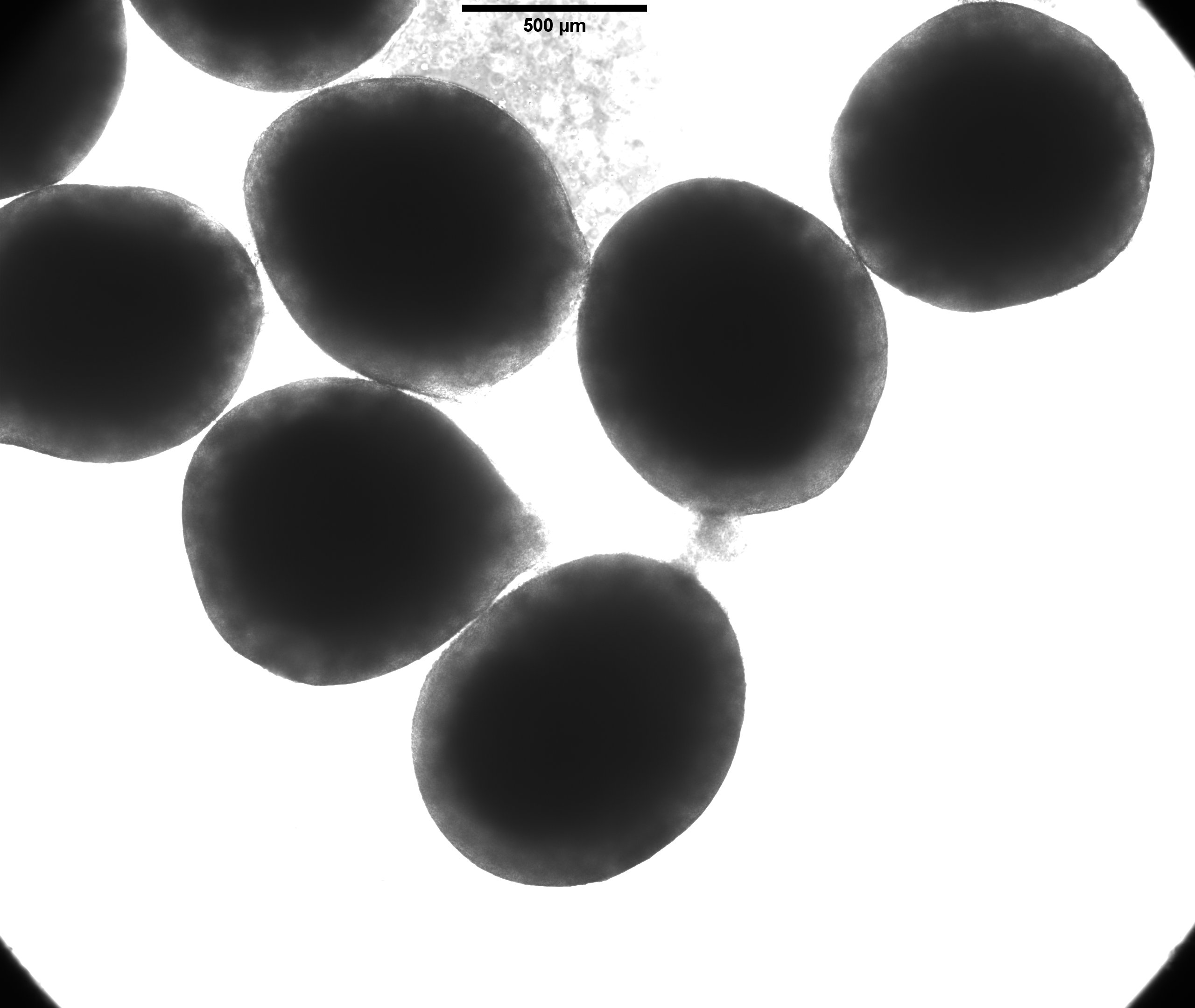

Supplement: Supplementary file 2 — Source Data for Figure 1 [file EMBJ-42-e113213-s003.zip › Figure1/Fig1B/Fig1B_iPSC3_D20_MGnull.jpg]

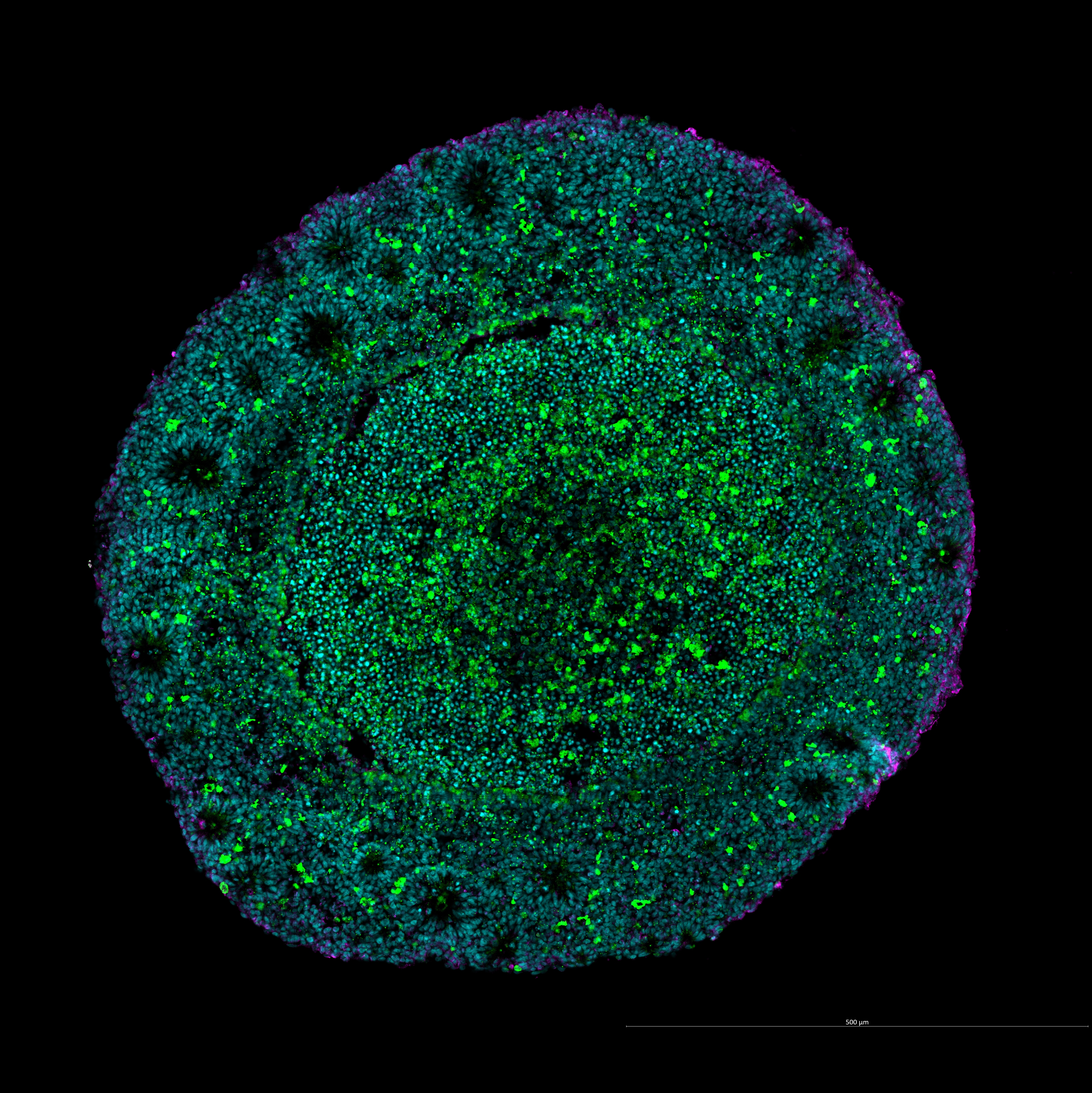

Supplement: Supplementary file 3 — Source Data for Figure 2 [file EMBJ-42-e113213-s006.zip › Figure2/Fig2K/Fig2K_H9_LamininLiq_D20_greenMshLAMA1-magentaMsLAMA1-cyanDAPI.jpg]

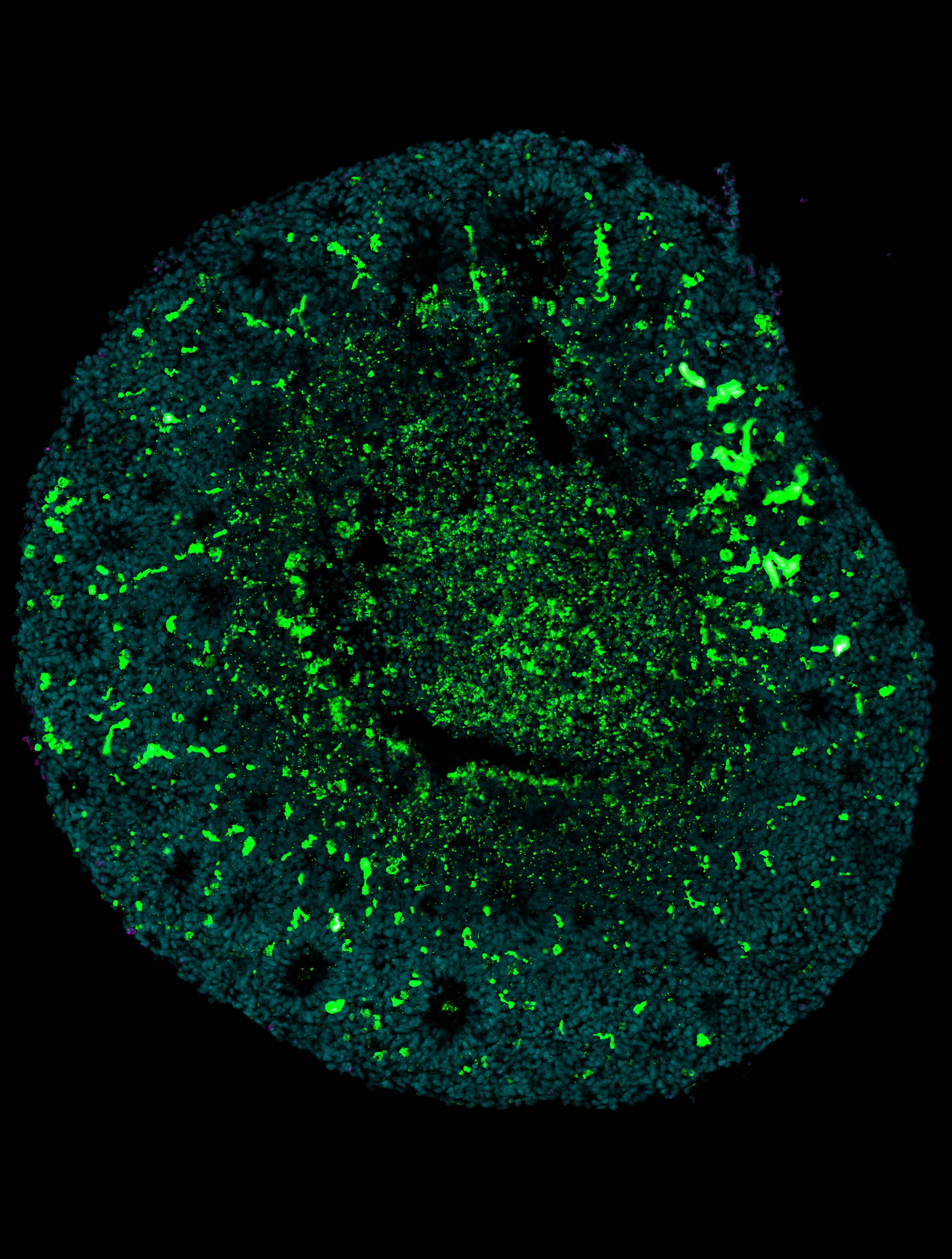

Supplement: Supplementary file 3 — Source Data for Figure 2 [file EMBJ-42-e113213-s006.zip › Figure2/Fig2K/Fig2K_H9_CollagenIVLiq_D20_greenMshLAMA1-magentaMsLAMA1-cyanDAPI.jpg]

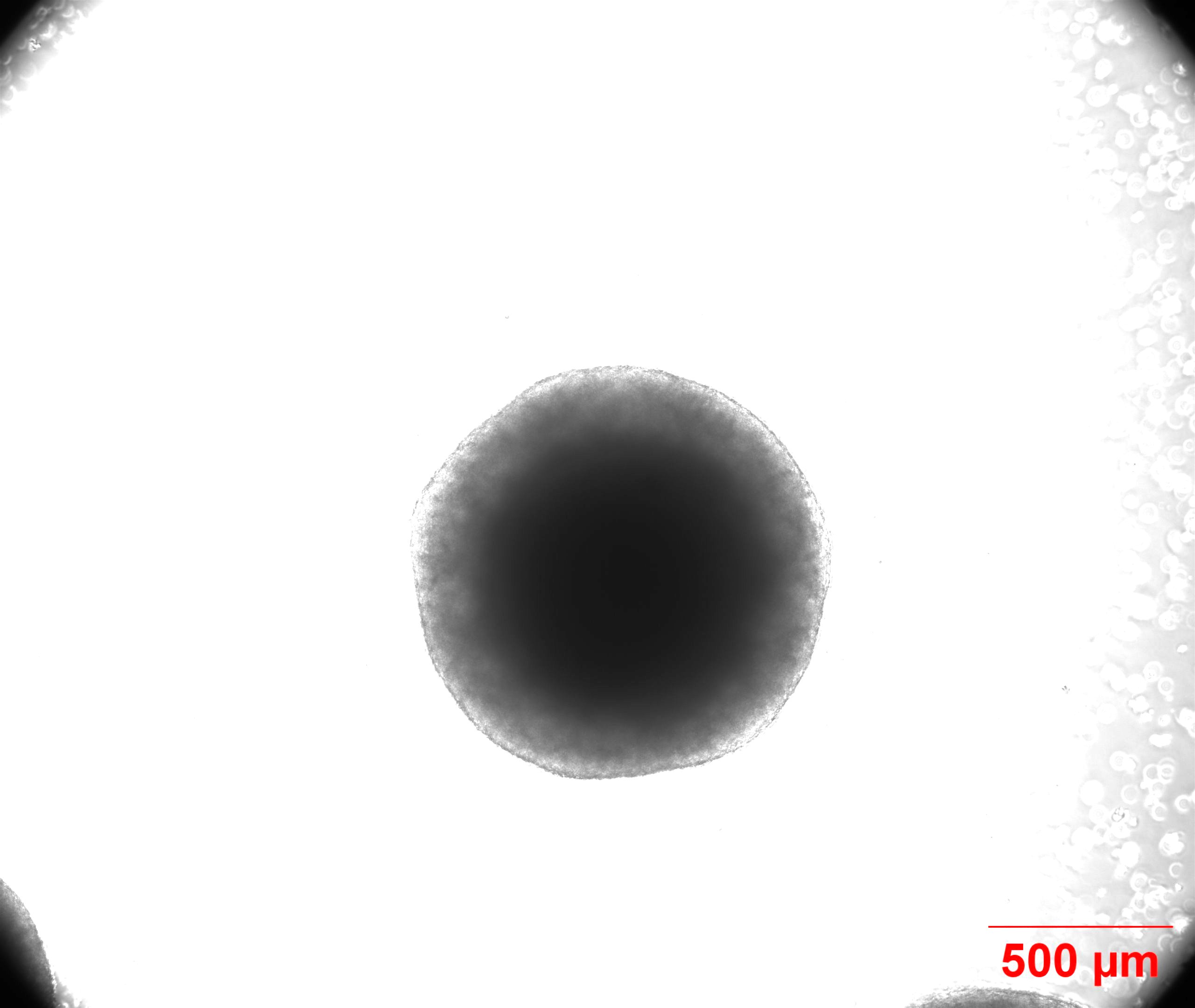

Supplement: Supplementary file 3 — Source Data for Figure 2 [file EMBJ-42-e113213-s006.zip › Figure2/Fig2K/Fig2K_H9_CollagenIVLiq_D20.jpg]

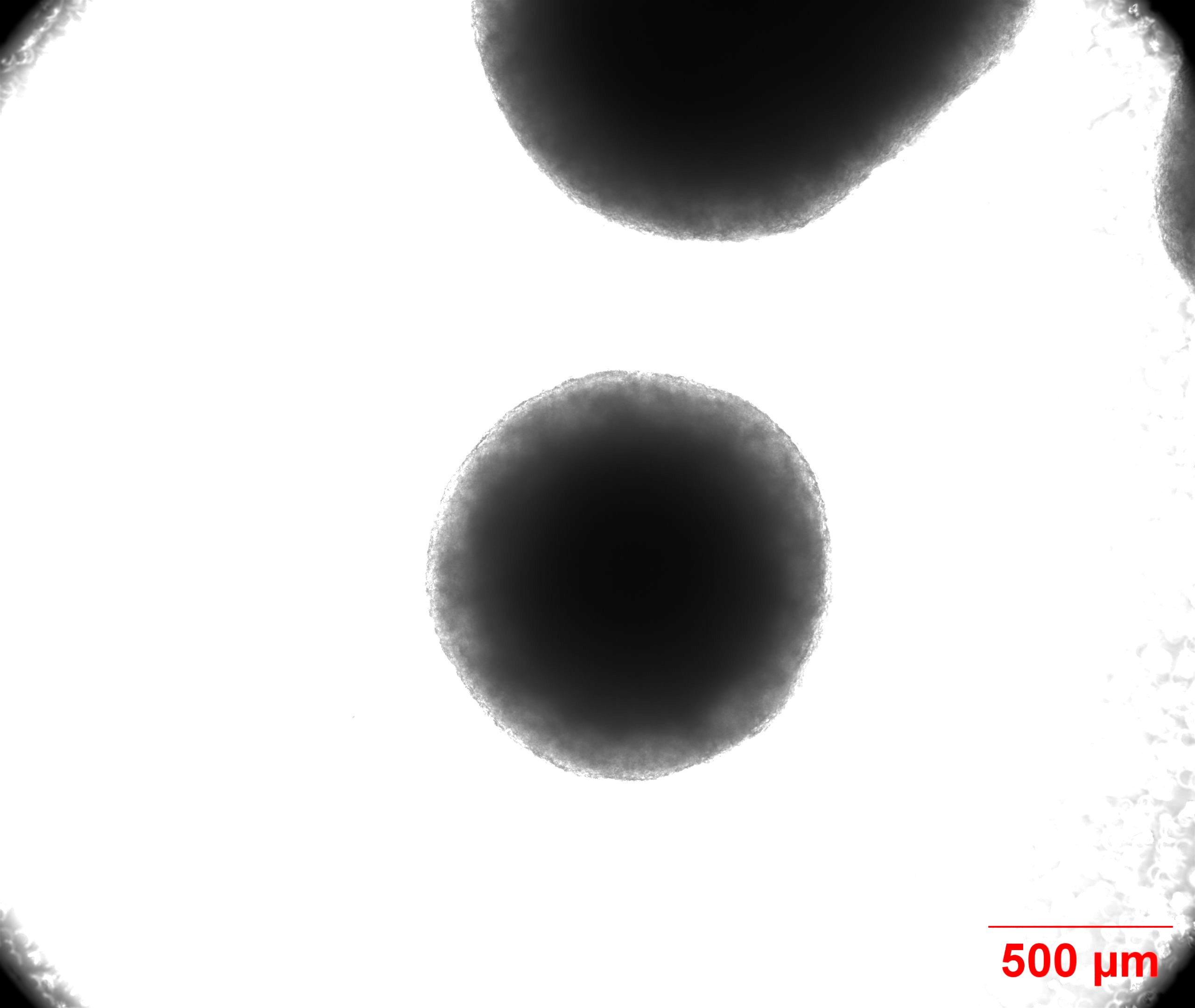

Supplement: Supplementary file 3 — Source Data for Figure 2 [file EMBJ-42-e113213-s006.zip › Figure2/Fig2K/Fig2K_H9_LamininLiq_D20.jpg]

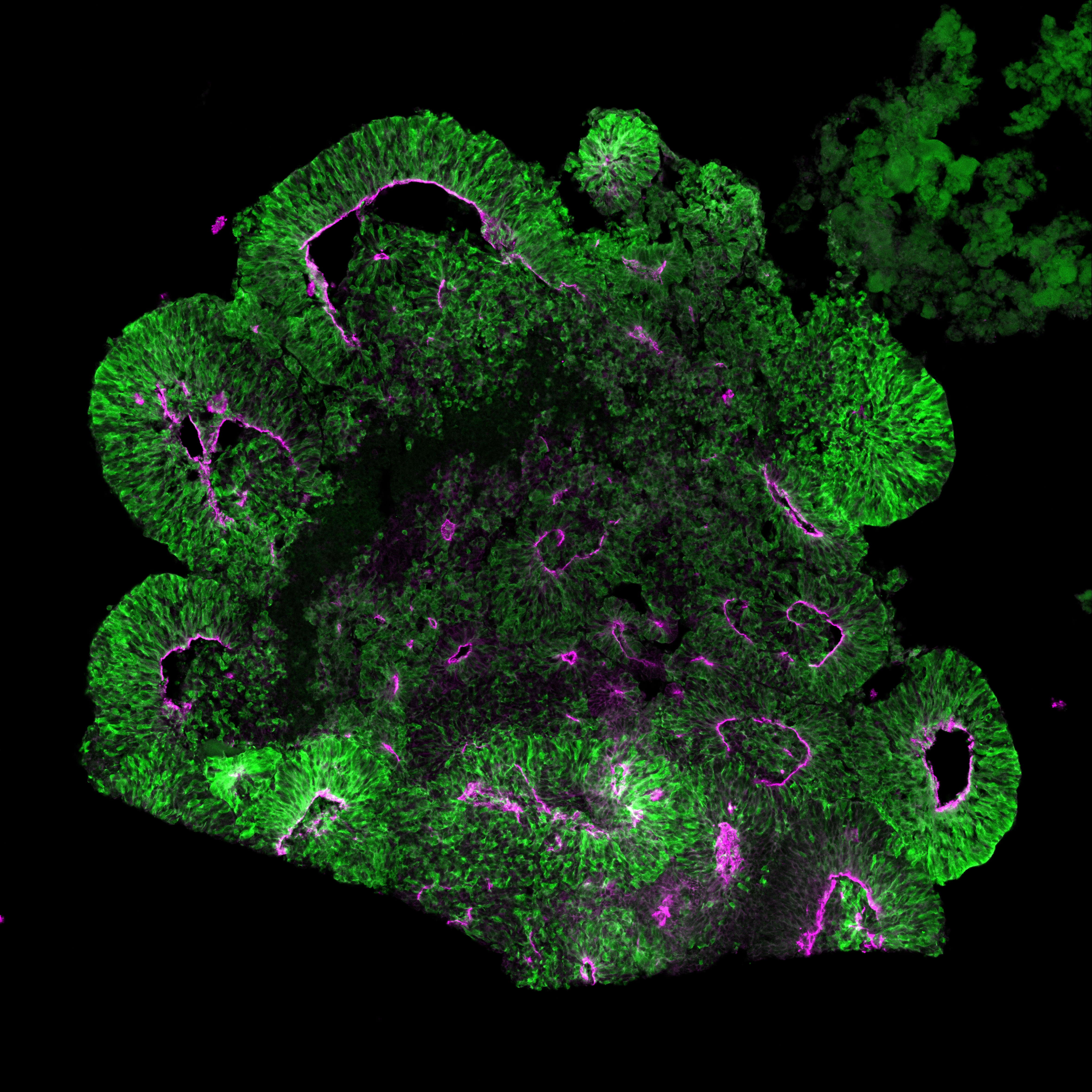

Supplement: Supplementary file 3 — Source Data for Figure 2 [file EMBJ-42-e113213-s006.zip › Figure2/Fig2D/Fig2D_H920percSOX2_MGdrop_D20_greenGFP-magentaPKC.jpg]

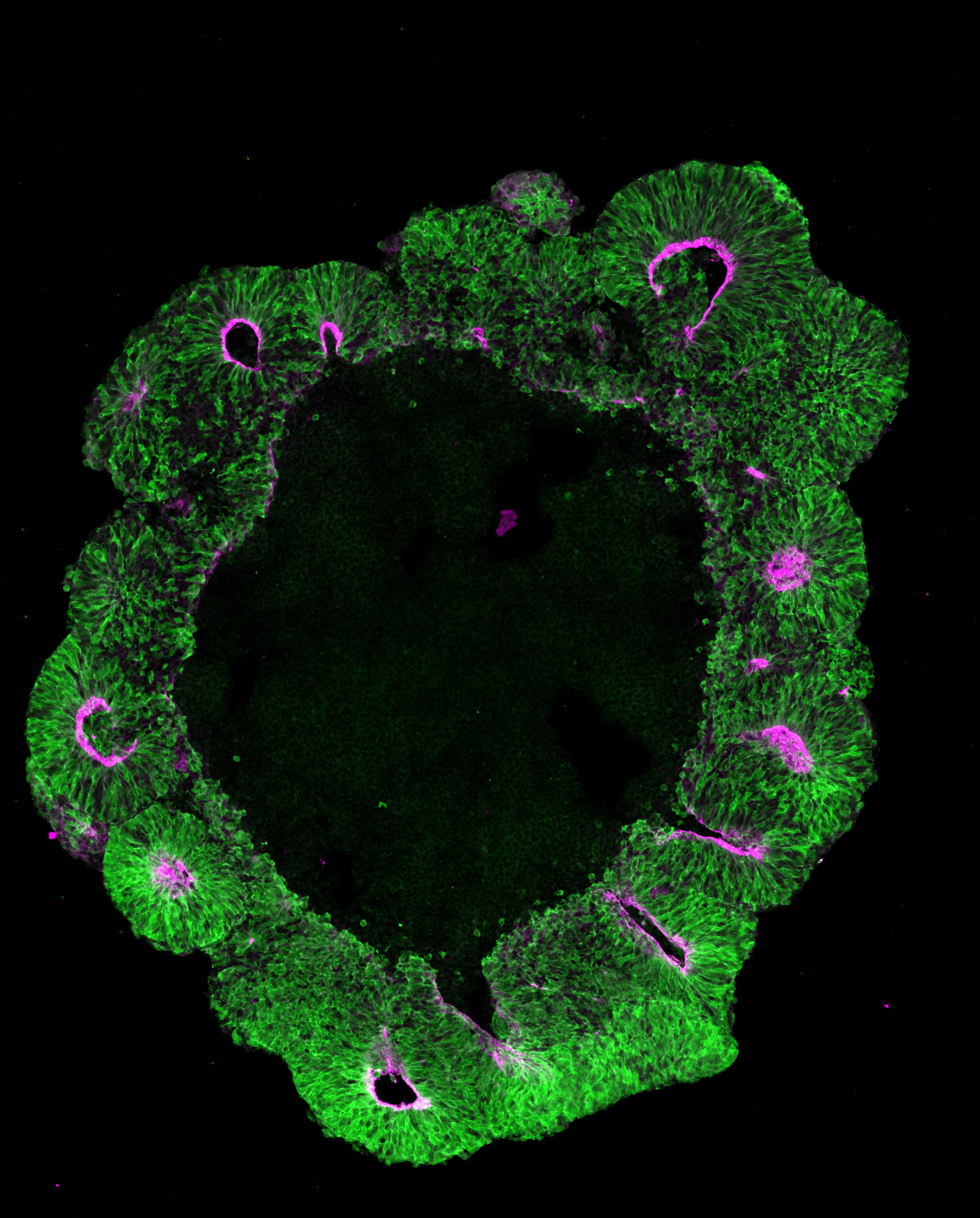

Supplement: Supplementary file 3 — Source Data for Figure 2 [file EMBJ-42-e113213-s006.zip › Figure2/Fig2D/Fig2D_H920percSOX2_MGliq_D20_greenGFP-magentaPKC.jpg.jpg]

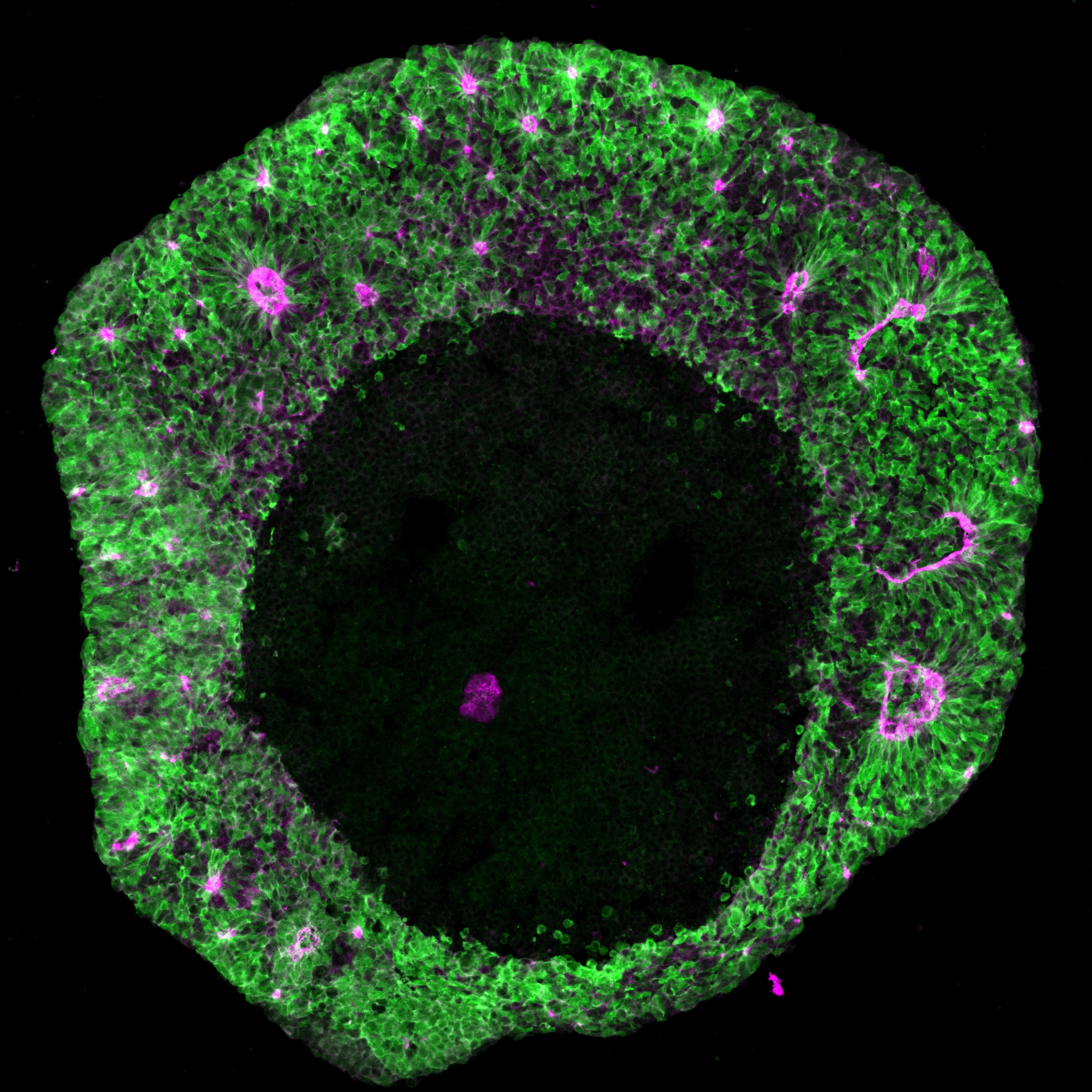

Supplement: Supplementary file 3 — Source Data for Figure 2 [file EMBJ-42-e113213-s006.zip › Figure2/Fig2D/Fig2D_H920percSOX2_MGnull_D20_greenGFP-magentaPKC.jpg.jpg]

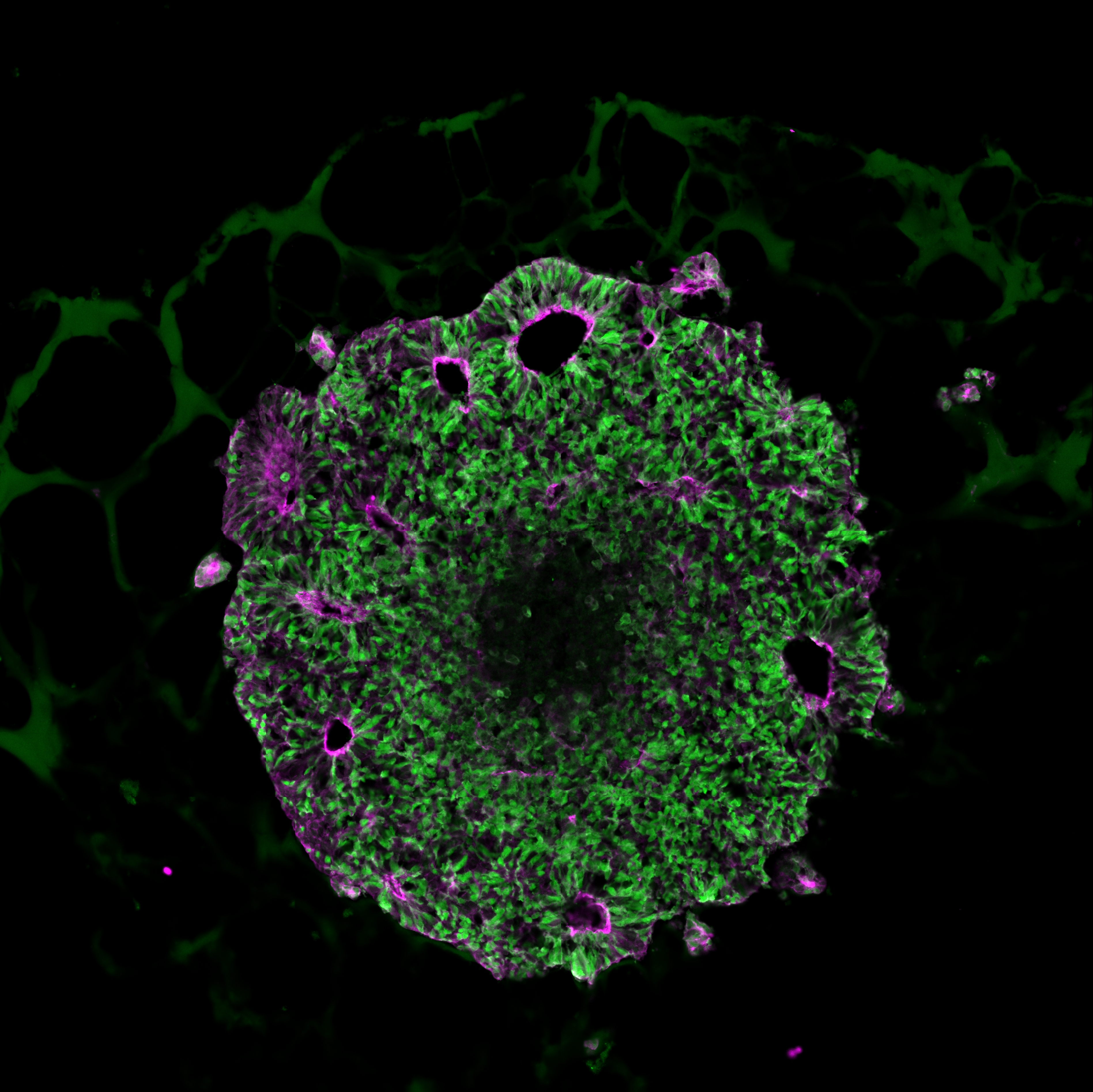

Supplement: Supplementary file 3 — Source Data for Figure 2 [file EMBJ-42-e113213-s006.zip › Figure2/Fig2C/Fig2C_H920percSOX2_MGdrop_D13_greenGFP-magentaPKC.jpg.jpg]

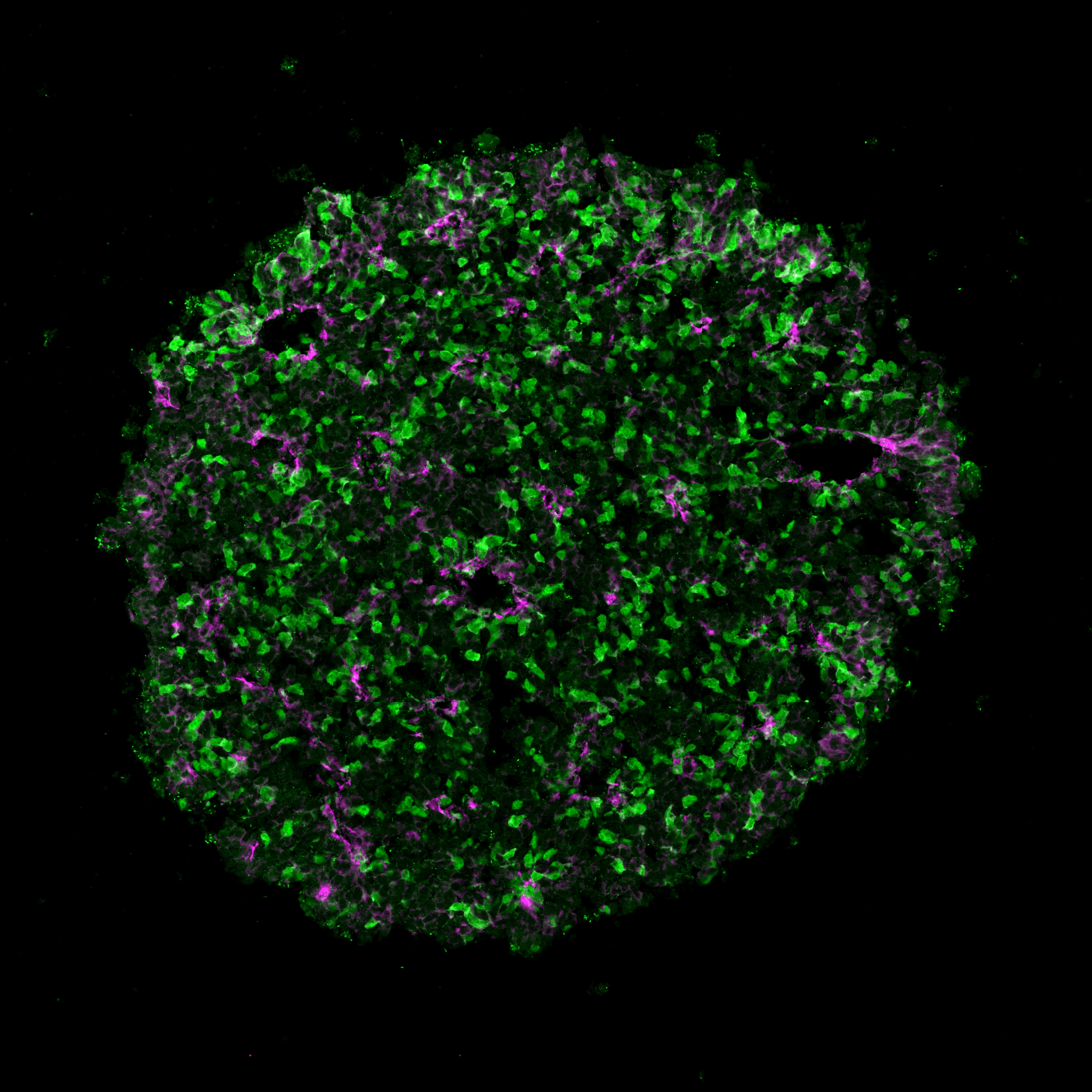

Supplement: Supplementary file 3 — Source Data for Figure 2 [file EMBJ-42-e113213-s006.zip › Figure2/Fig2C/Fig2C_H920percSOX2_MGliq_D13_greenGFP-magentaPKC.jpg.jpg]

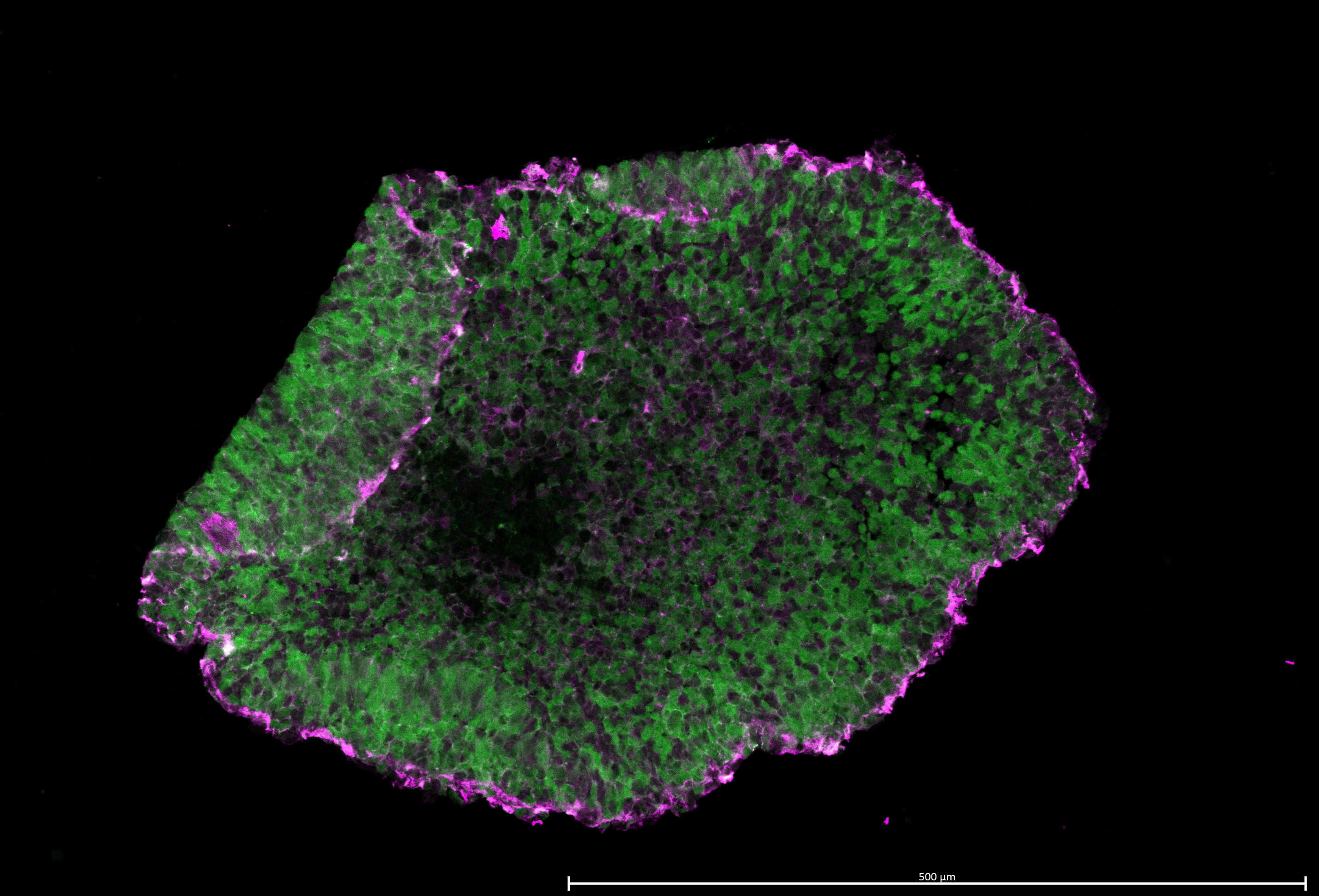

Supplement: Supplementary file 3 — Source Data for Figure 2 [file EMBJ-42-e113213-s006.zip › Figure2/Fig2C/Fig2C_H920percSOX2_MGnull_D13_greenGFP-magentaPKC.jpg]

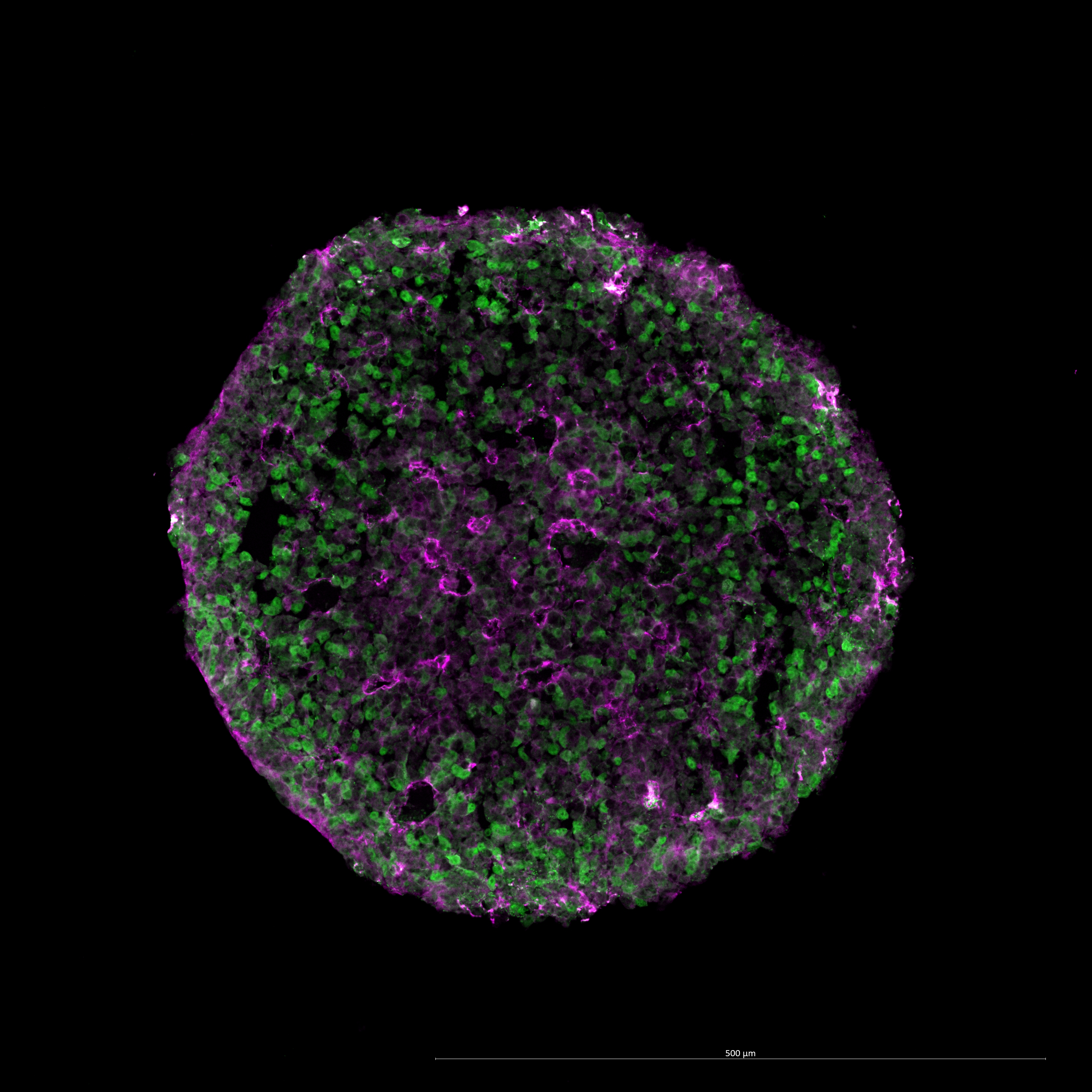

Supplement: Supplementary file 3 — Source Data for Figure 2 [file EMBJ-42-e113213-s006.zip › Figure2/Fig2A/Fig2A_H920percSOX2_D10_greenGFP-magentaPKC.jpg]

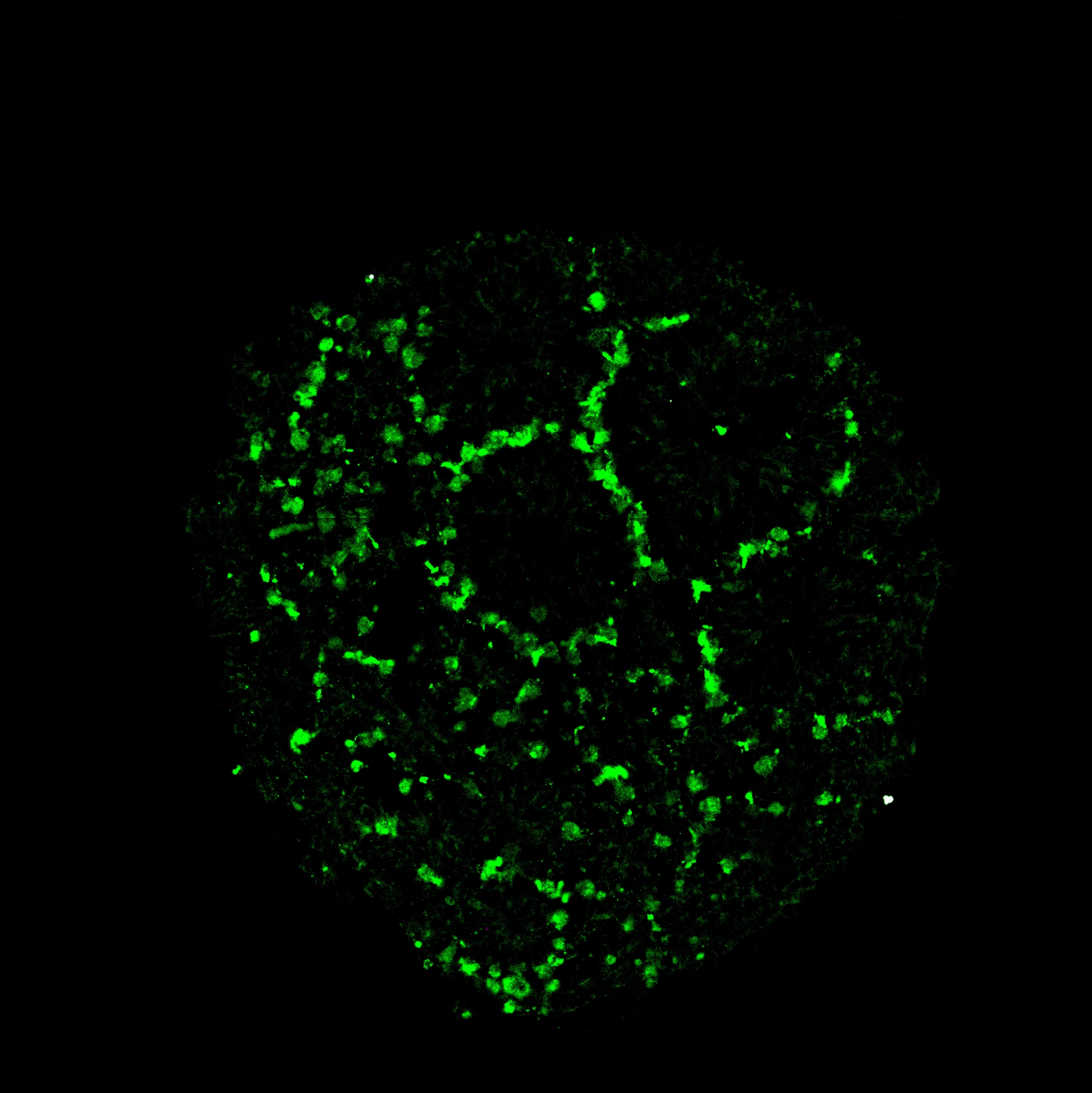

Supplement: Supplementary file 3 — Source Data for Figure 2 [file EMBJ-42-e113213-s006.zip › Figure2/Fig2H/Fig2H_H9_MGnull_D20_greenPerlecan-magentaMsLAMA1.jpg]

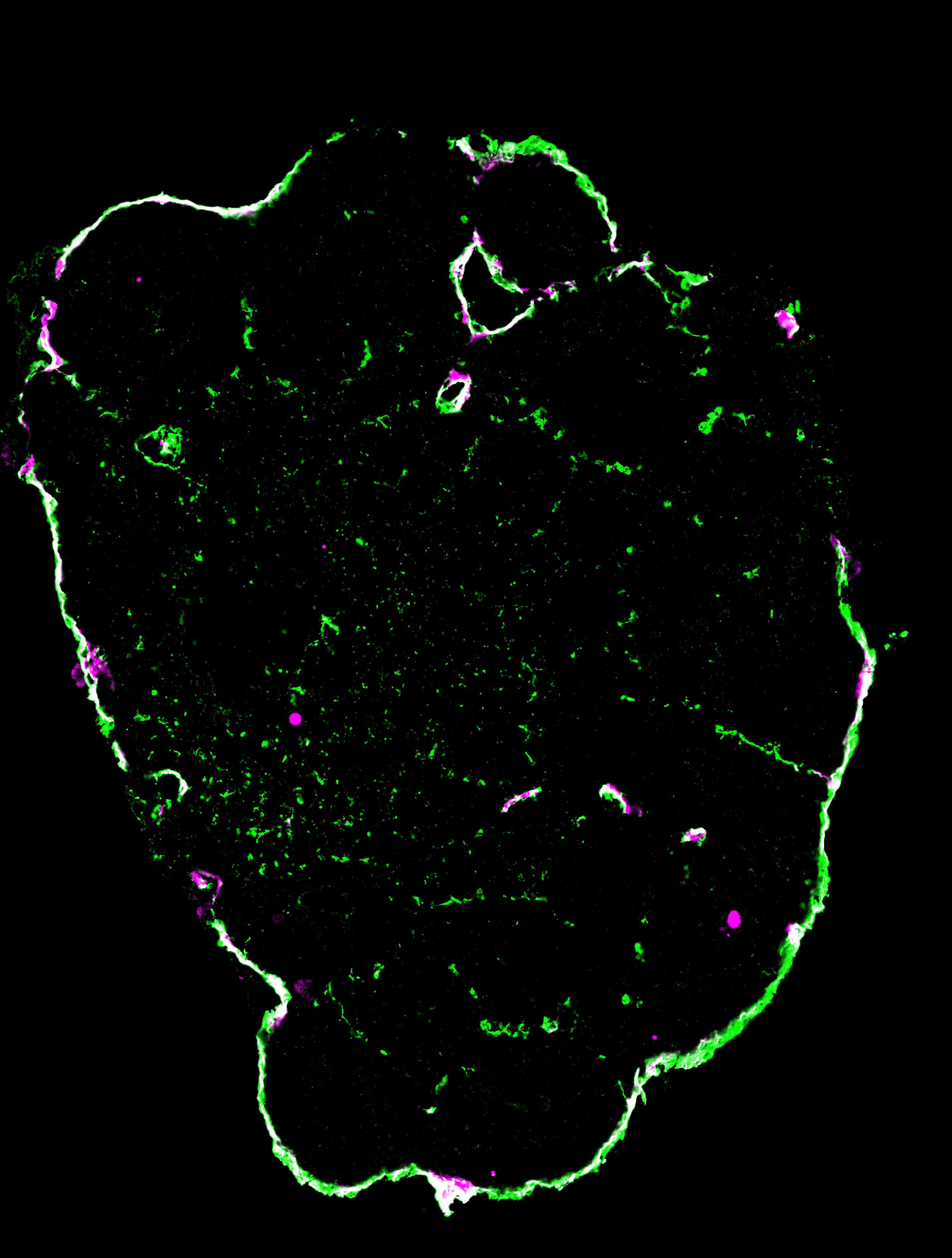

Supplement: Supplementary file 3 — Source Data for Figure 2 [file EMBJ-42-e113213-s006.zip › Figure2/Fig2H/Fig2H_H9_MGliq_D20_greenMshLAMA1-magentaMsLAMA1.jpg]

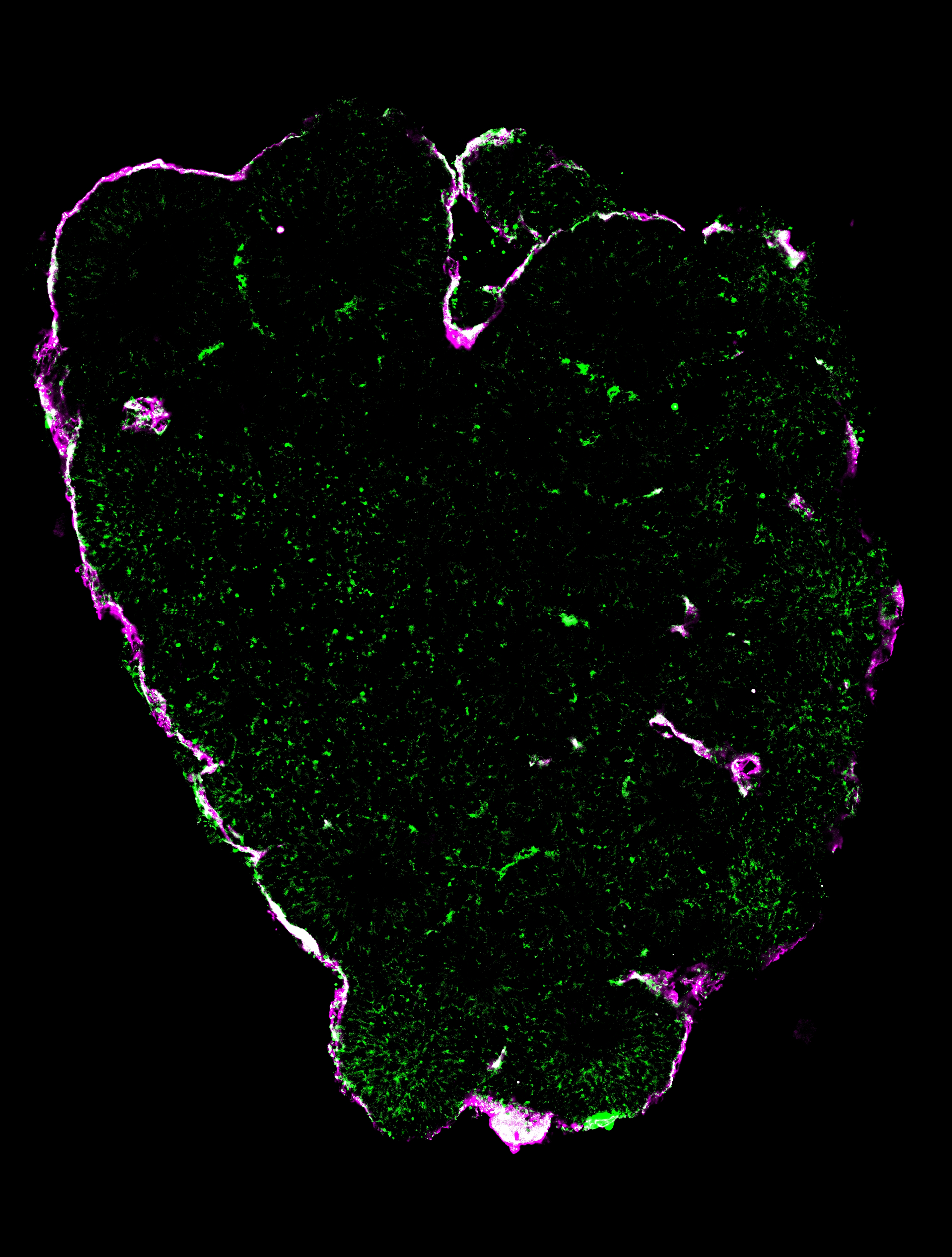

Supplement: Supplementary file 3 — Source Data for Figure 2 [file EMBJ-42-e113213-s006.zip › Figure2/Fig2H/Fig2H_H9_MGliq_D20_greenFN-magentaMsLAMA1.jpg.jpg]

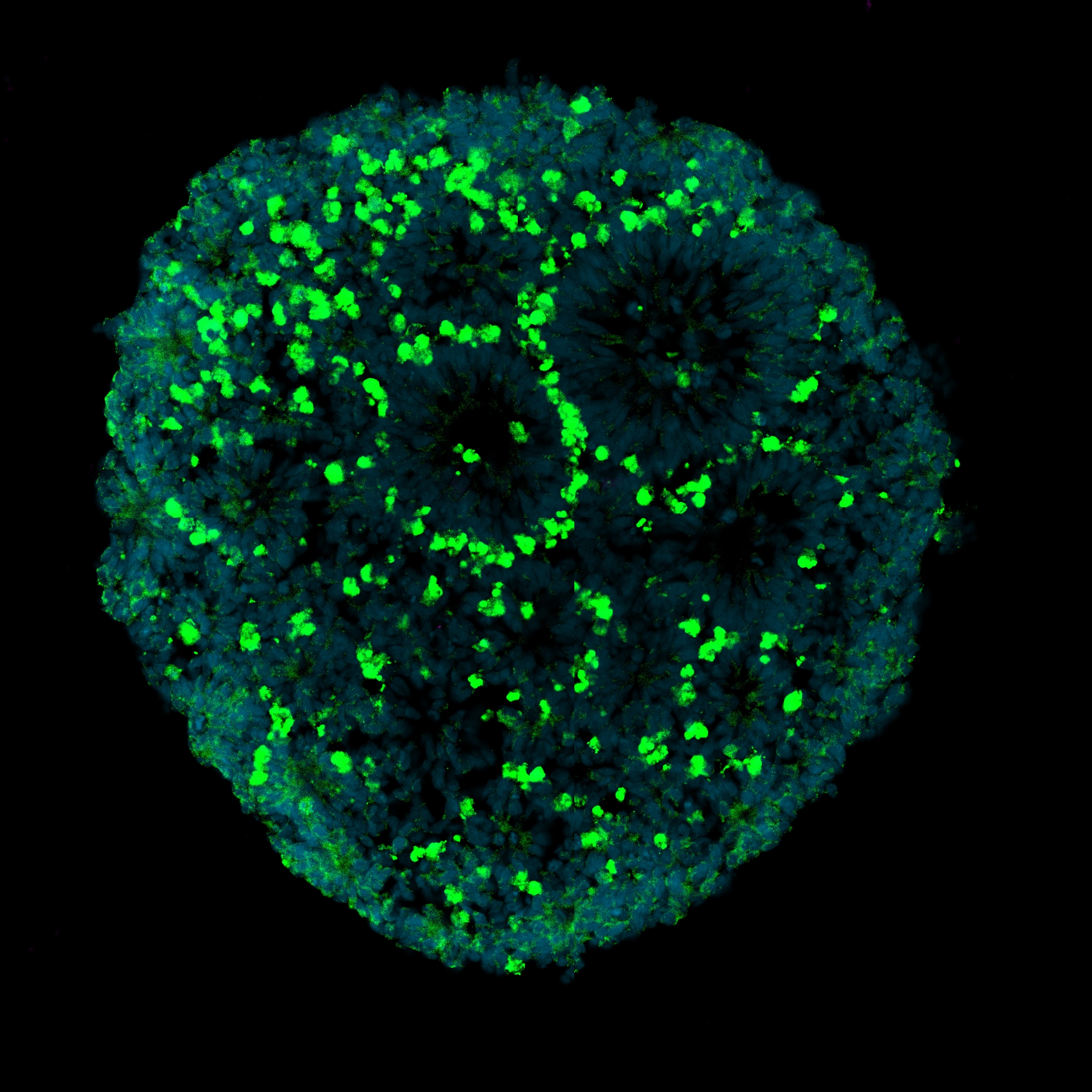

Supplement: Supplementary file 3 — Source Data for Figure 2 [file EMBJ-42-e113213-s006.zip › Figure2/Fig2H/Fig2H_H9_MGnull_D20_greenFN-magentaMsLAMA1-cyanDAPI.jpg]

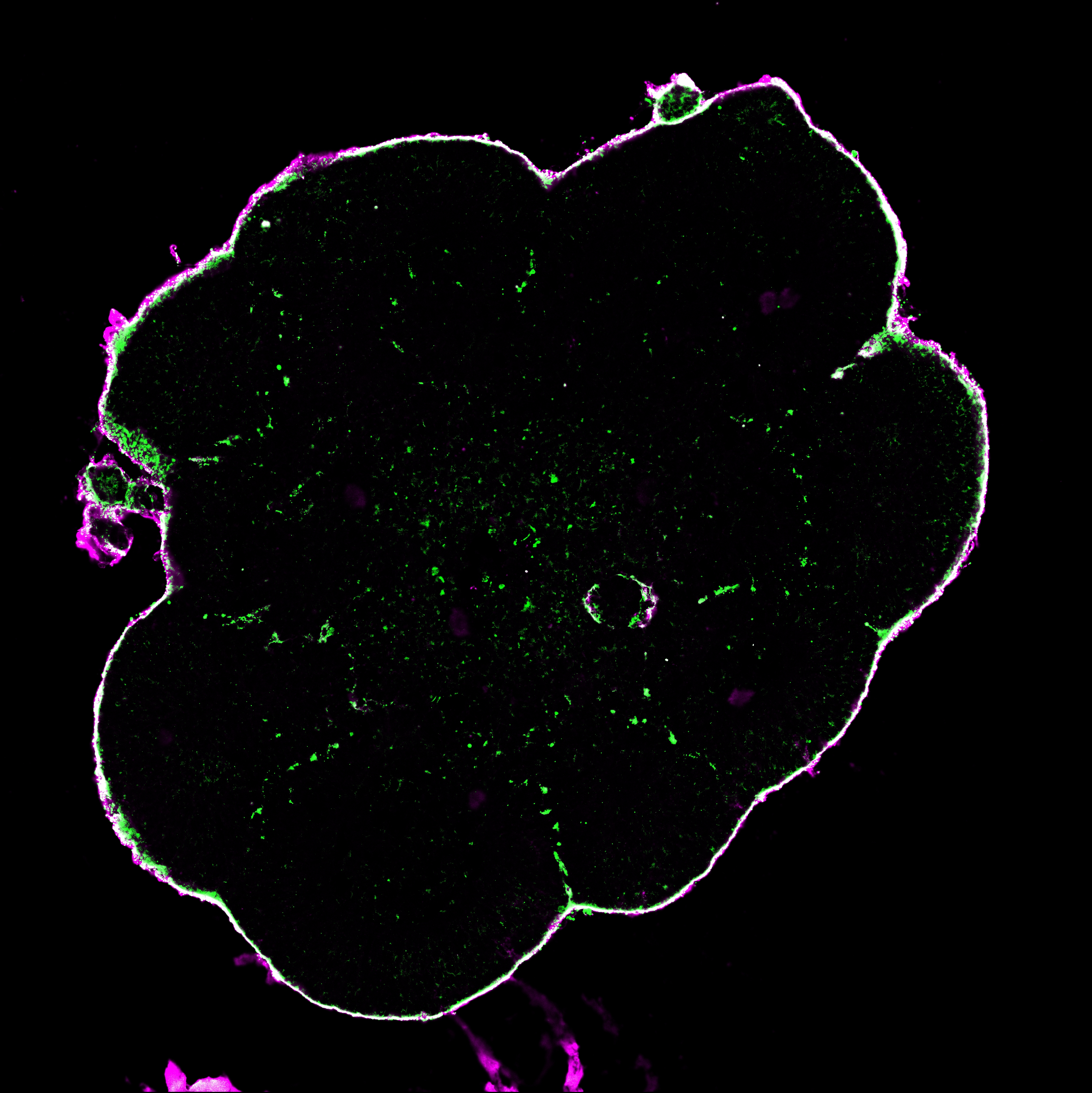

Supplement: Supplementary file 3 — Source Data for Figure 2 [file EMBJ-42-e113213-s006.zip › Figure2/Fig2H/Fig2H_H9_MGdrop_D20_greenFN-magentaMsLAMA1.jpg]

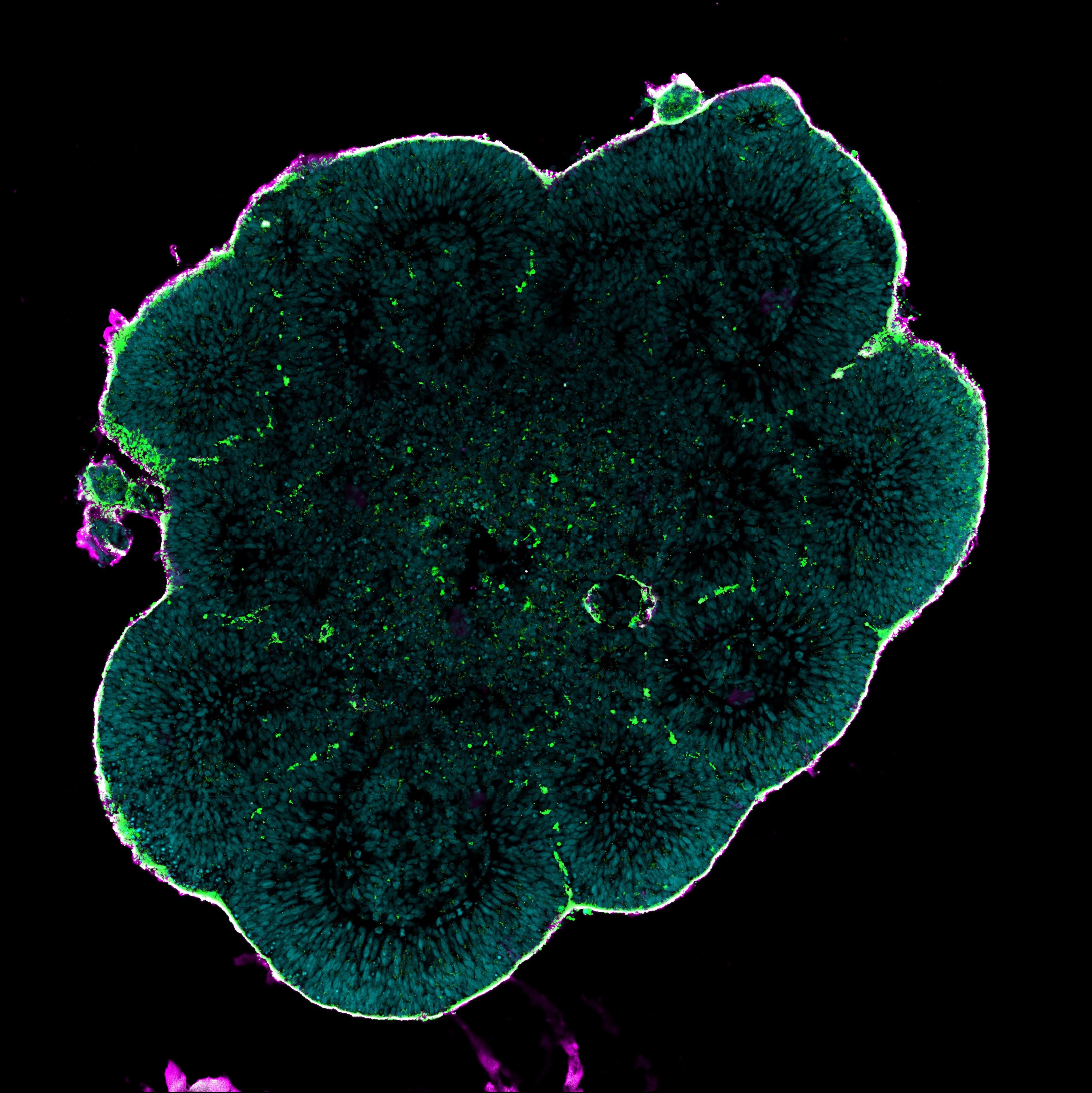

Supplement: Supplementary file 3 — Source Data for Figure 2 [file EMBJ-42-e113213-s006.zip › Figure2/Fig2H/Fig2H_H9_MGdrop_D20_greenFN-magentaMsLAMA1-cyanDAPI.jpg]

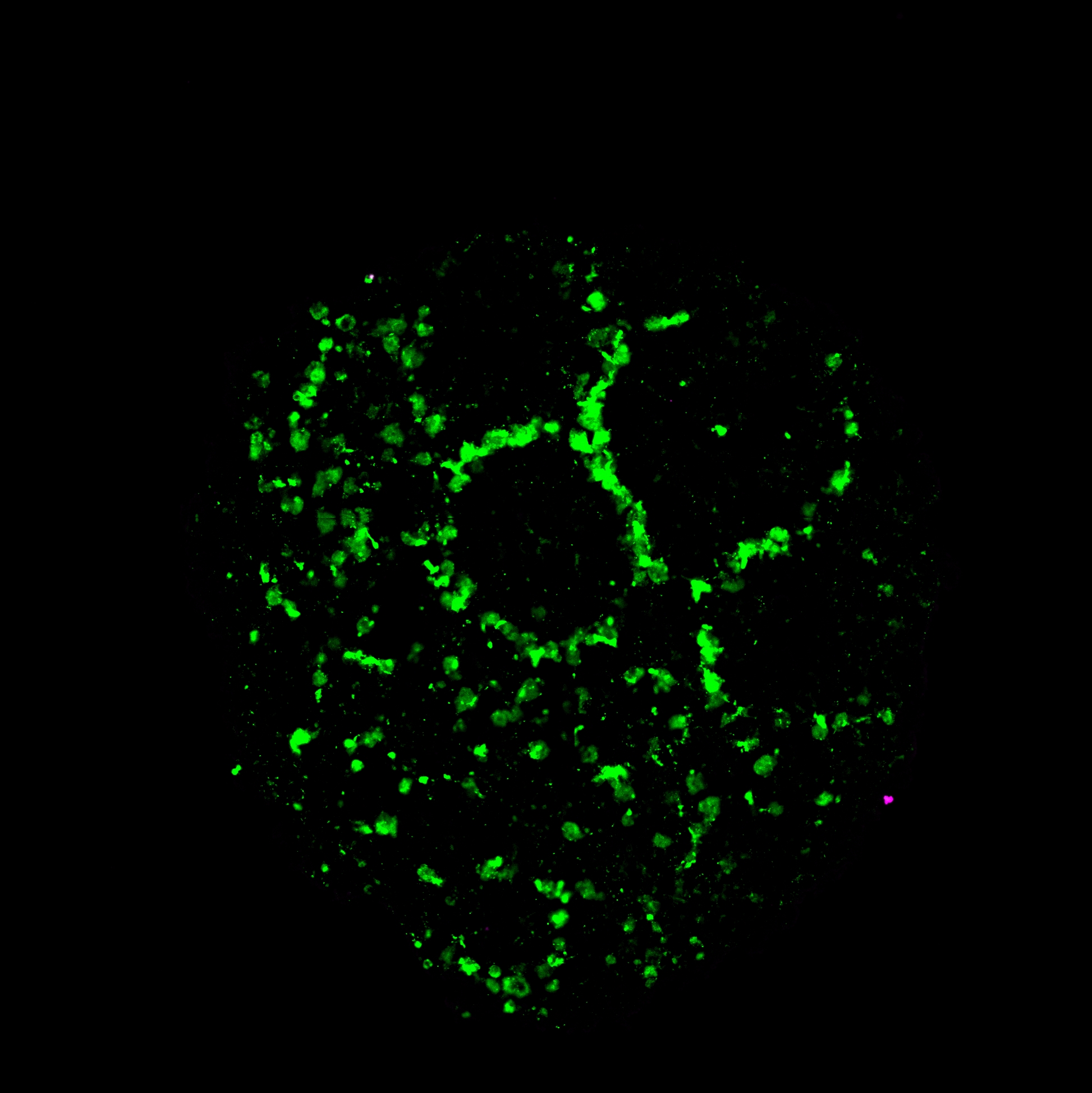

Supplement: Supplementary file 3 — Source Data for Figure 2 [file EMBJ-42-e113213-s006.zip › Figure2/Fig2H/Fig2H_H9_MGnull_D20_greenMshLAMA1-magentaMsLAMA1.jpg]

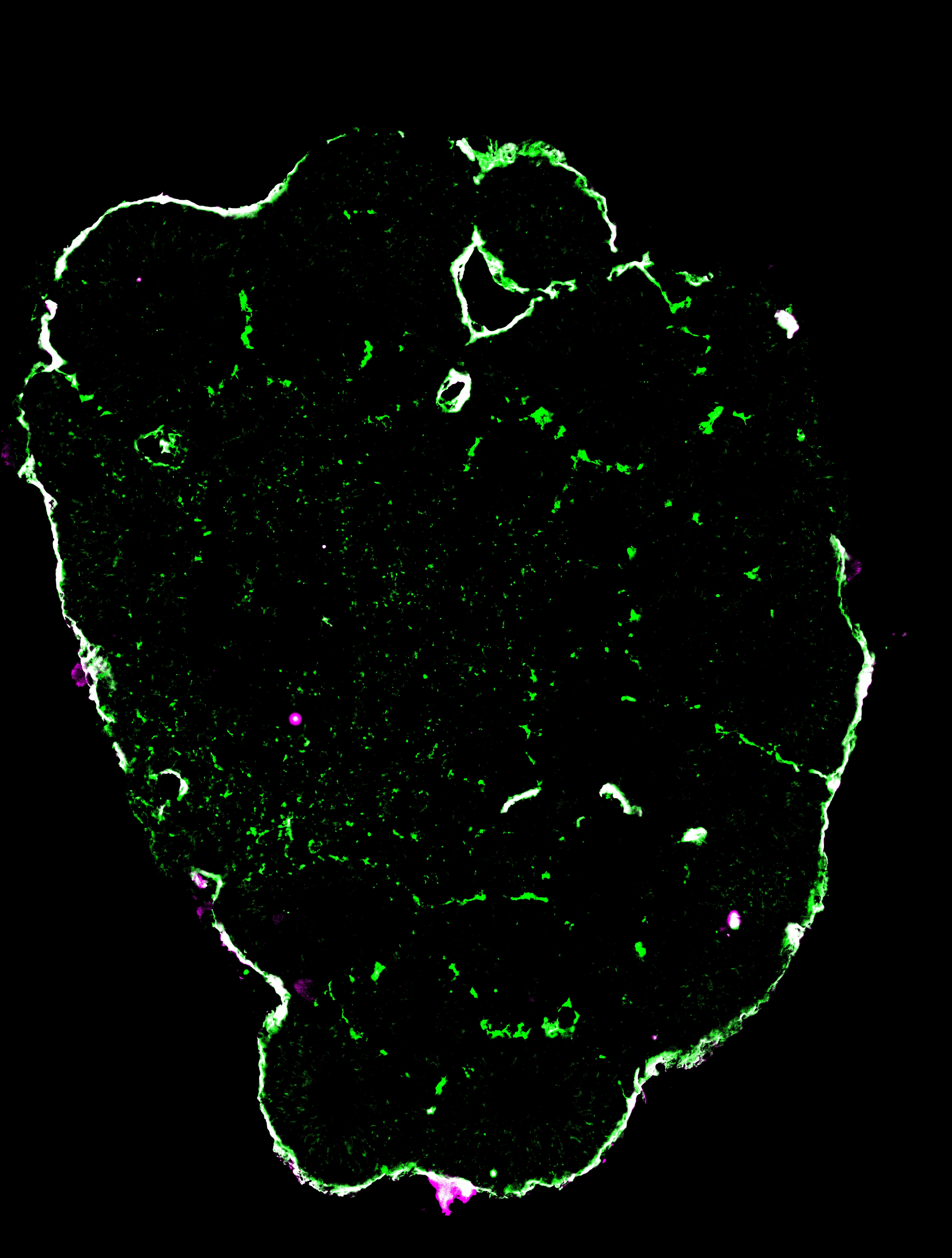

Supplement: Supplementary file 3 — Source Data for Figure 2 [file EMBJ-42-e113213-s006.zip › Figure2/Fig2H/Fig2H_H9_MGliq_D20_greenPerlecan-magentaMsLAMA1.jpg]

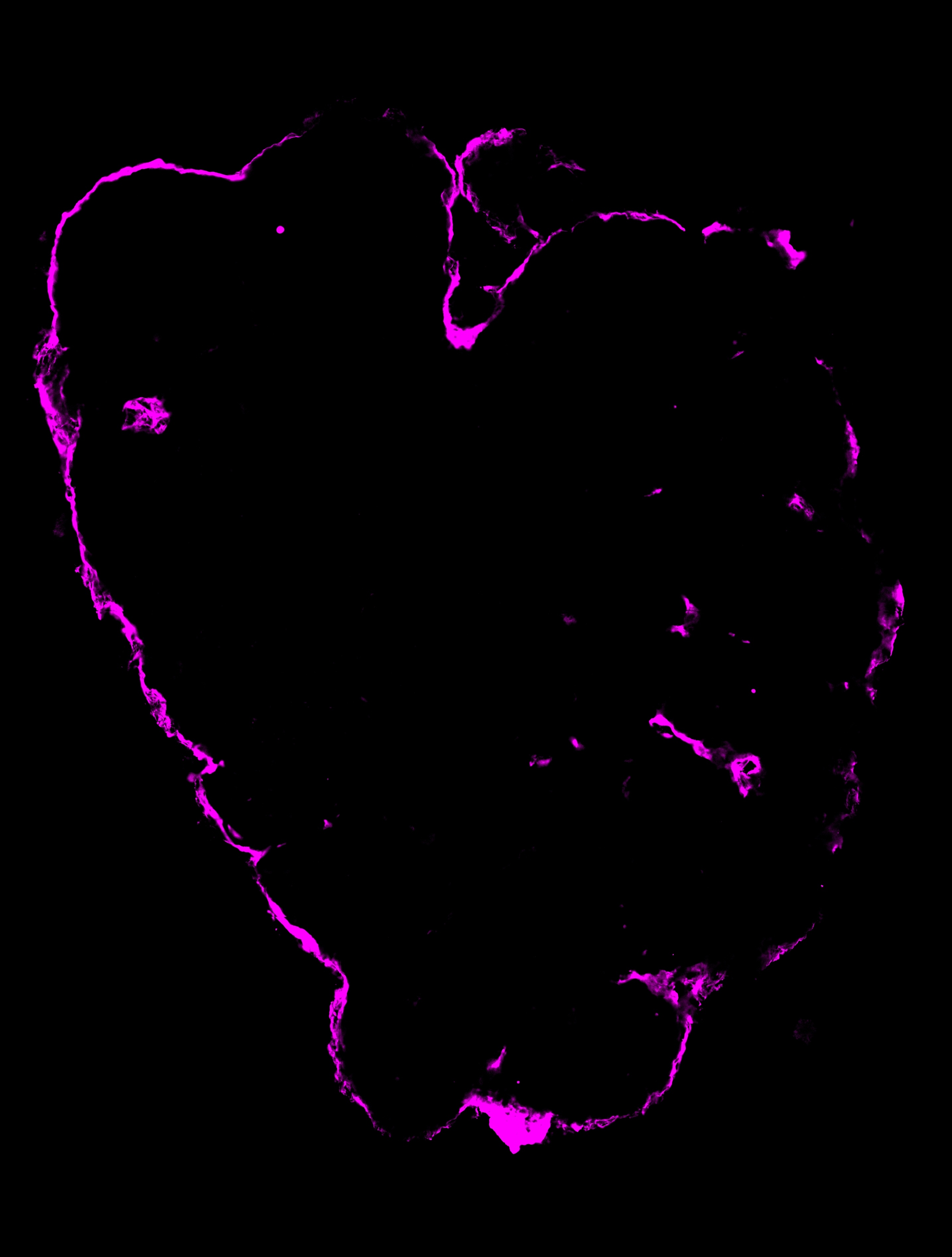

Supplement: Supplementary file 3 — Source Data for Figure 2 [file EMBJ-42-e113213-s006.zip › Figure2/Fig2H/Fig2H_H9_MGliq_D20_magentaMsLAMA1.jpg]

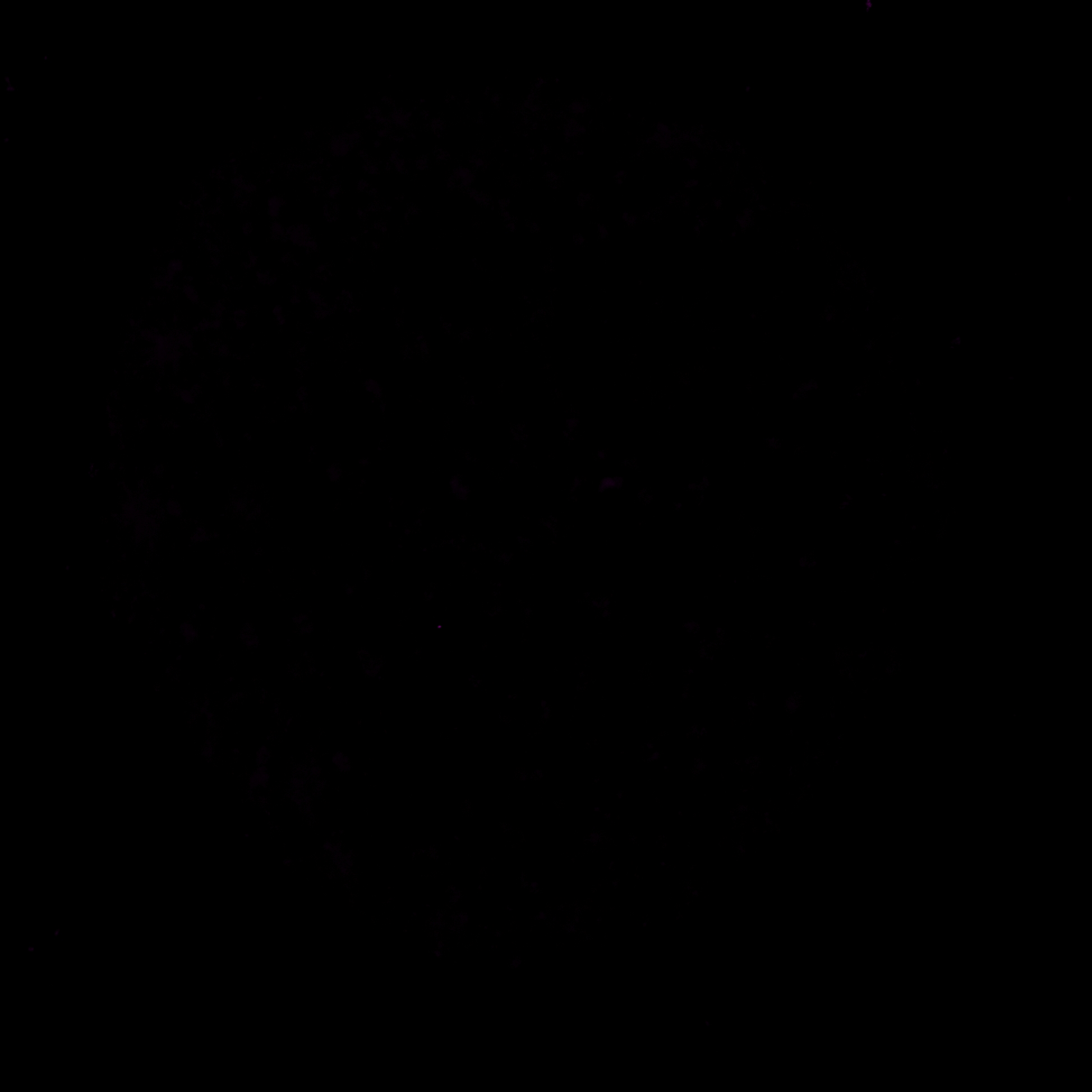

Supplement: Supplementary file 3 — Source Data for Figure 2 [file EMBJ-42-e113213-s006.zip › Figure2/Fig2H/Fig2H_H9_MGnull_D20_magentaMsLAMA1.jpg]

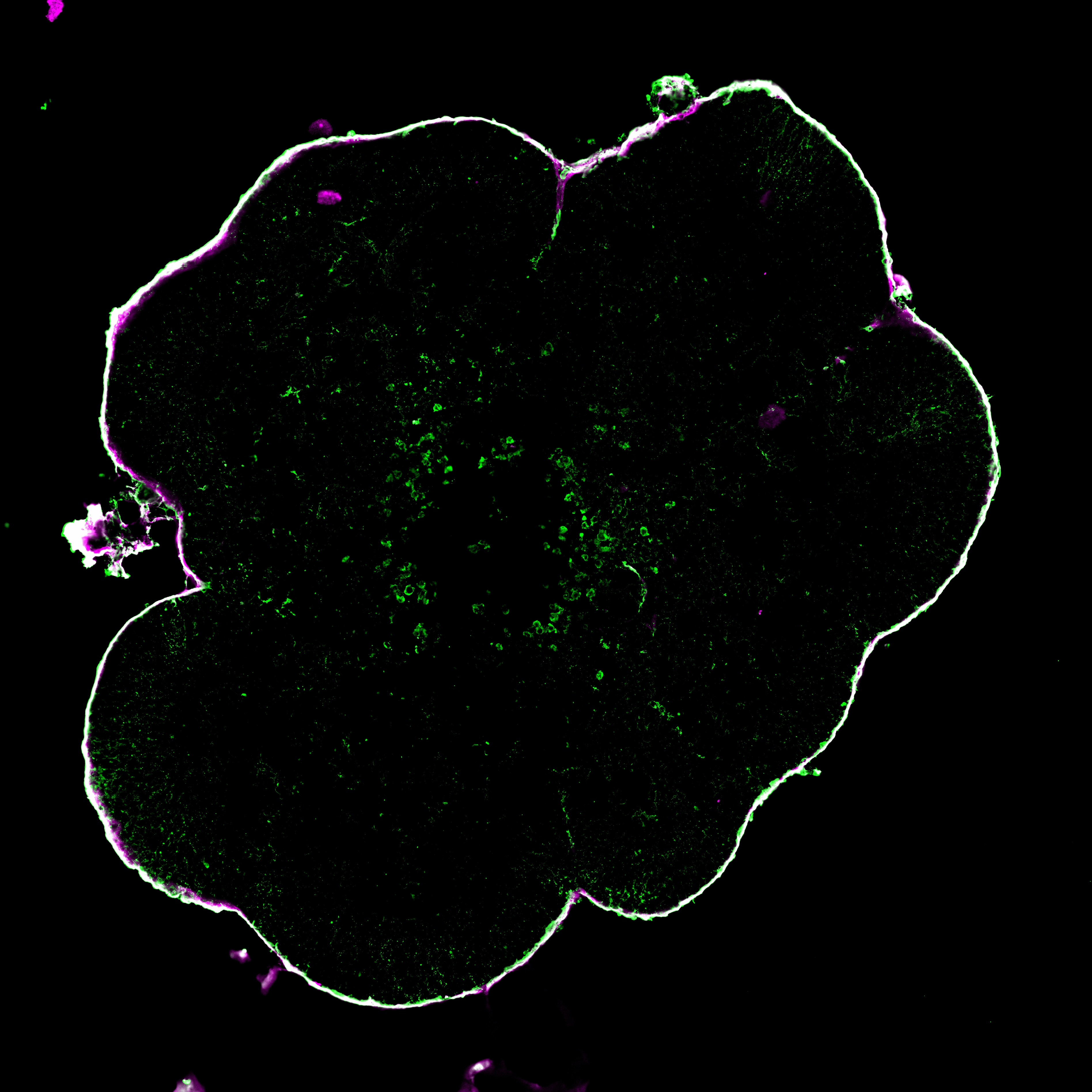

Supplement: Supplementary file 3 — Source Data for Figure 2 [file EMBJ-42-e113213-s006.zip › Figure2/Fig2H/Fig2H_H9_MGdrop_D20_greenMshLAMA1-magentaMsLAMA1.jpg]

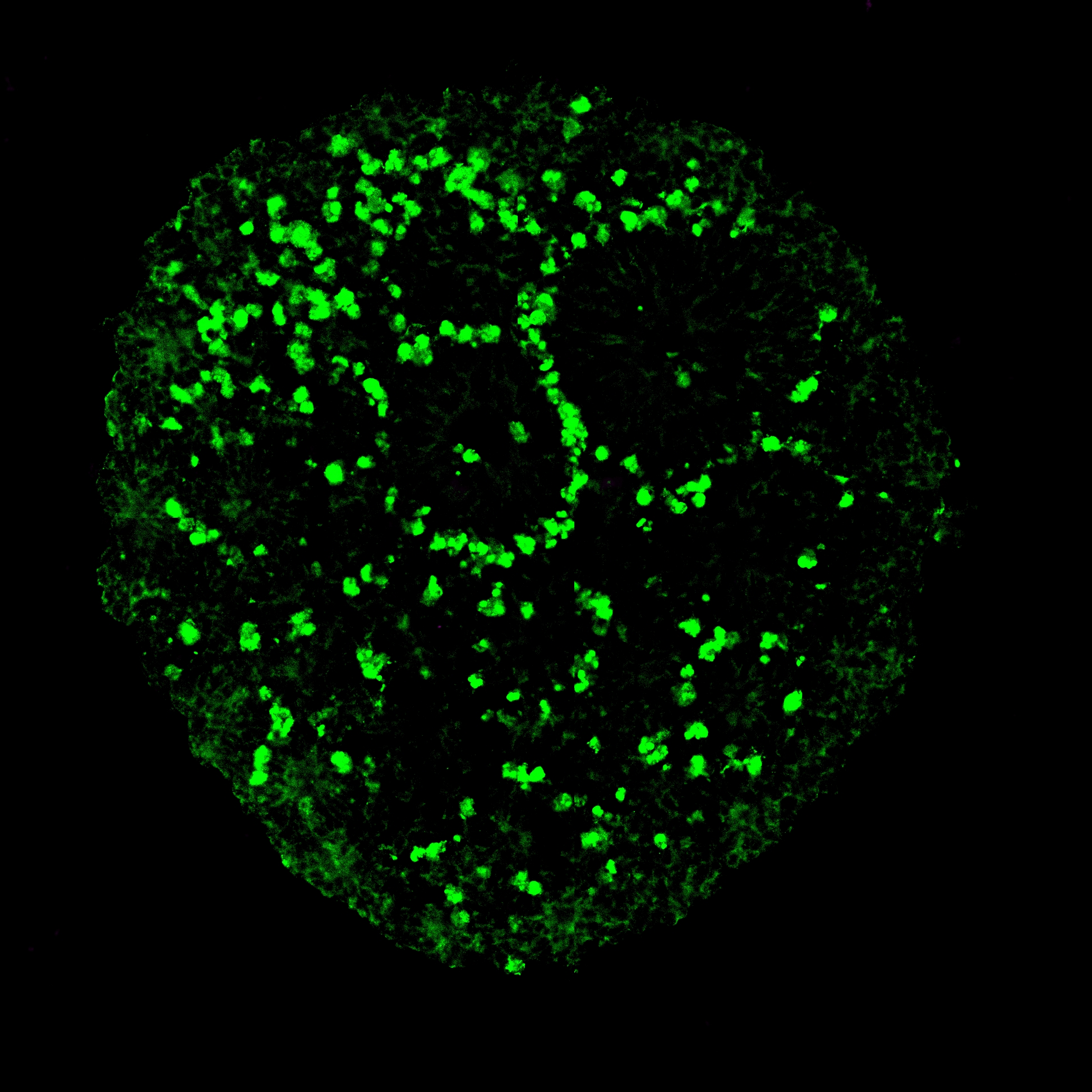

Supplement: Supplementary file 3 — Source Data for Figure 2 [file EMBJ-42-e113213-s006.zip › Figure2/Fig2H/Fig2H_H9_MGnull_D20_greenFN-magentaMsLAMA1.jpg]

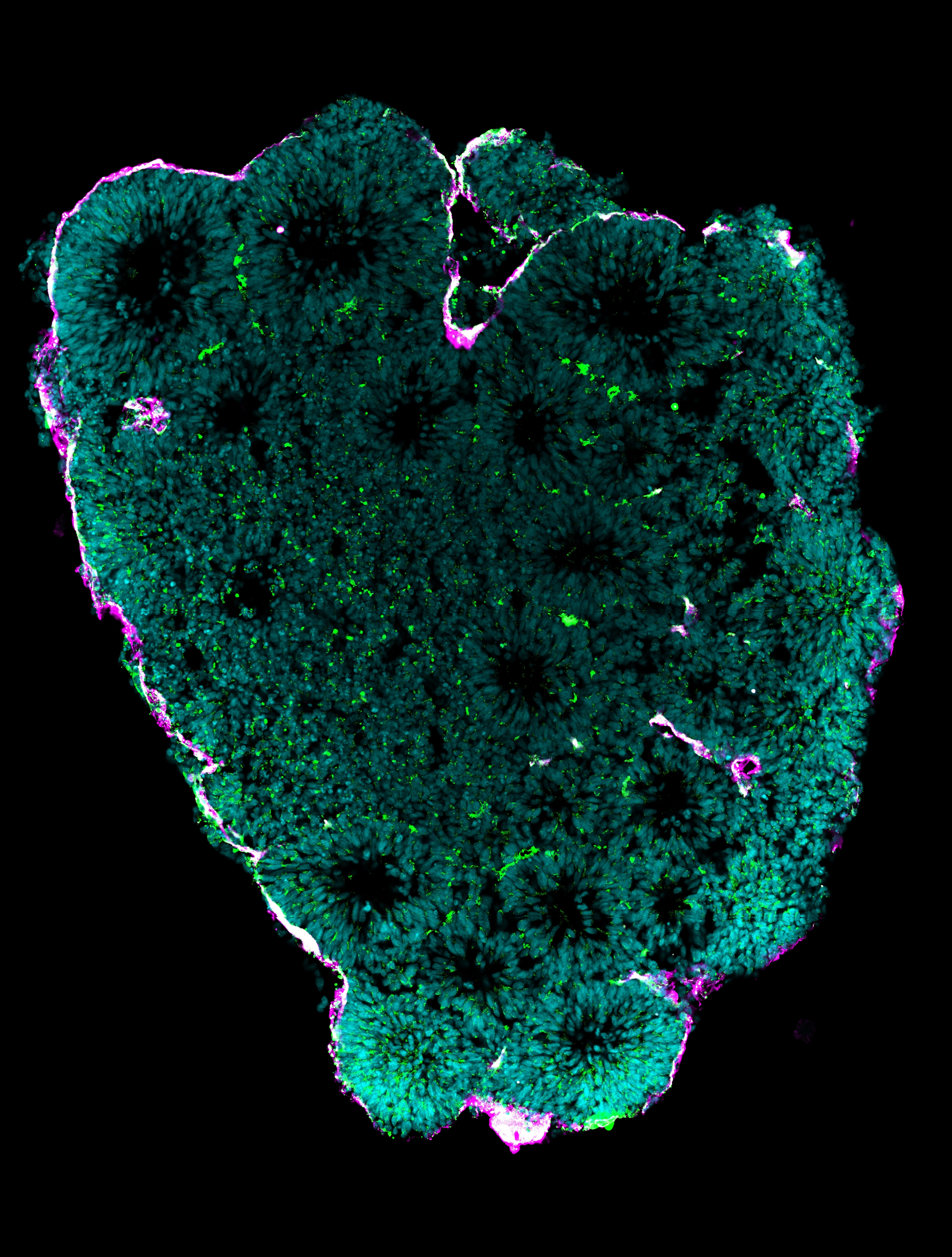

Supplement: Supplementary file 3 — Source Data for Figure 2 [file EMBJ-42-e113213-s006.zip › Figure2/Fig2H/Fig2H_H9_MGliq_D20_greenFN-magentaMsLAMA1-cyanDAPI.jpg]

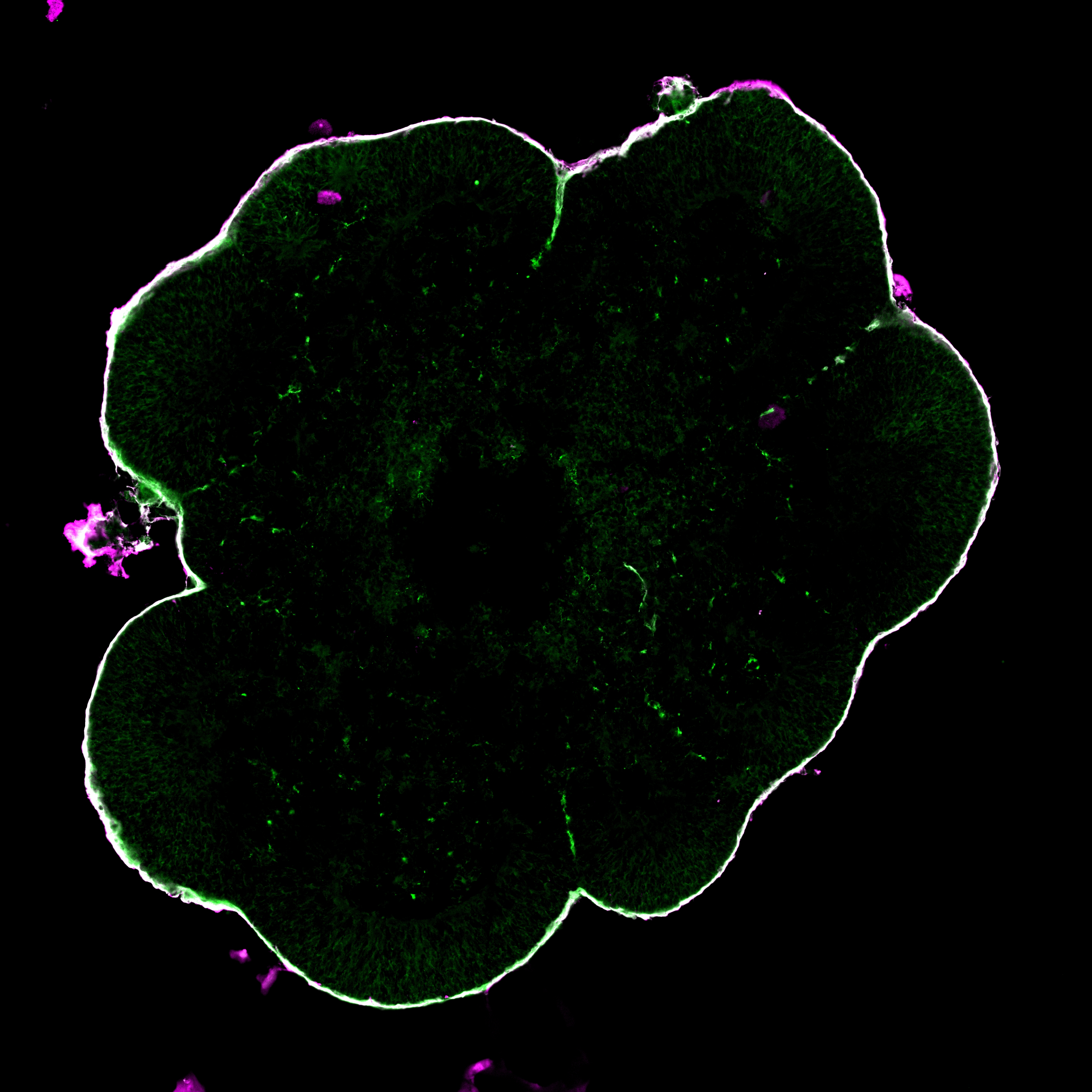

Supplement: Supplementary file 3 — Source Data for Figure 2 [file EMBJ-42-e113213-s006.zip › Figure2/Fig2H/Fig2H_H9_MGdrop_D20_greenPerlecan-magentaMsLAMA1.jpg]

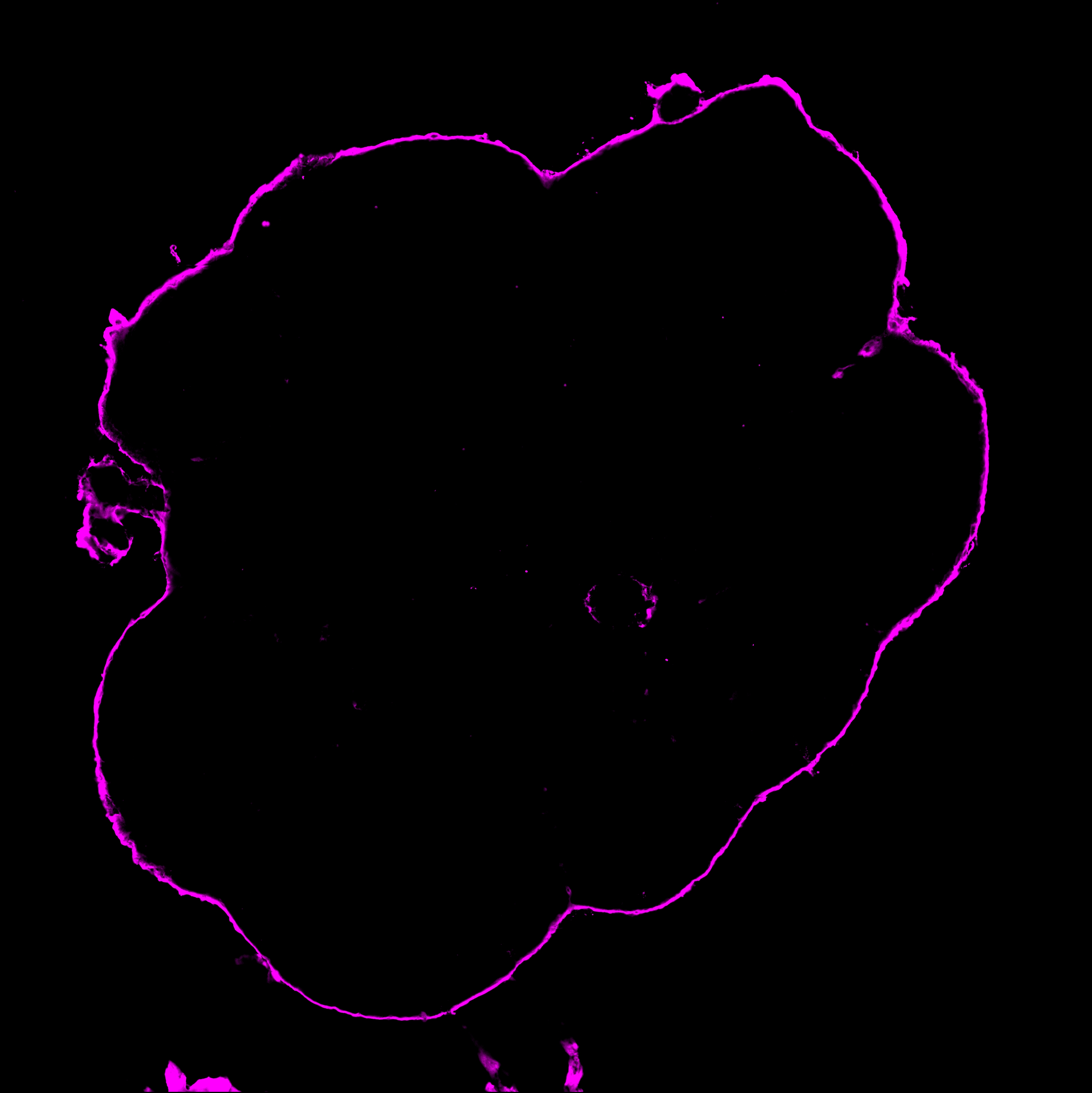

Supplement: Supplementary file 3 — Source Data for Figure 2 [file EMBJ-42-e113213-s006.zip › Figure2/Fig2H/Fig2H_H9_MGdrop_D20_magentaMsLAMA1.jpg]

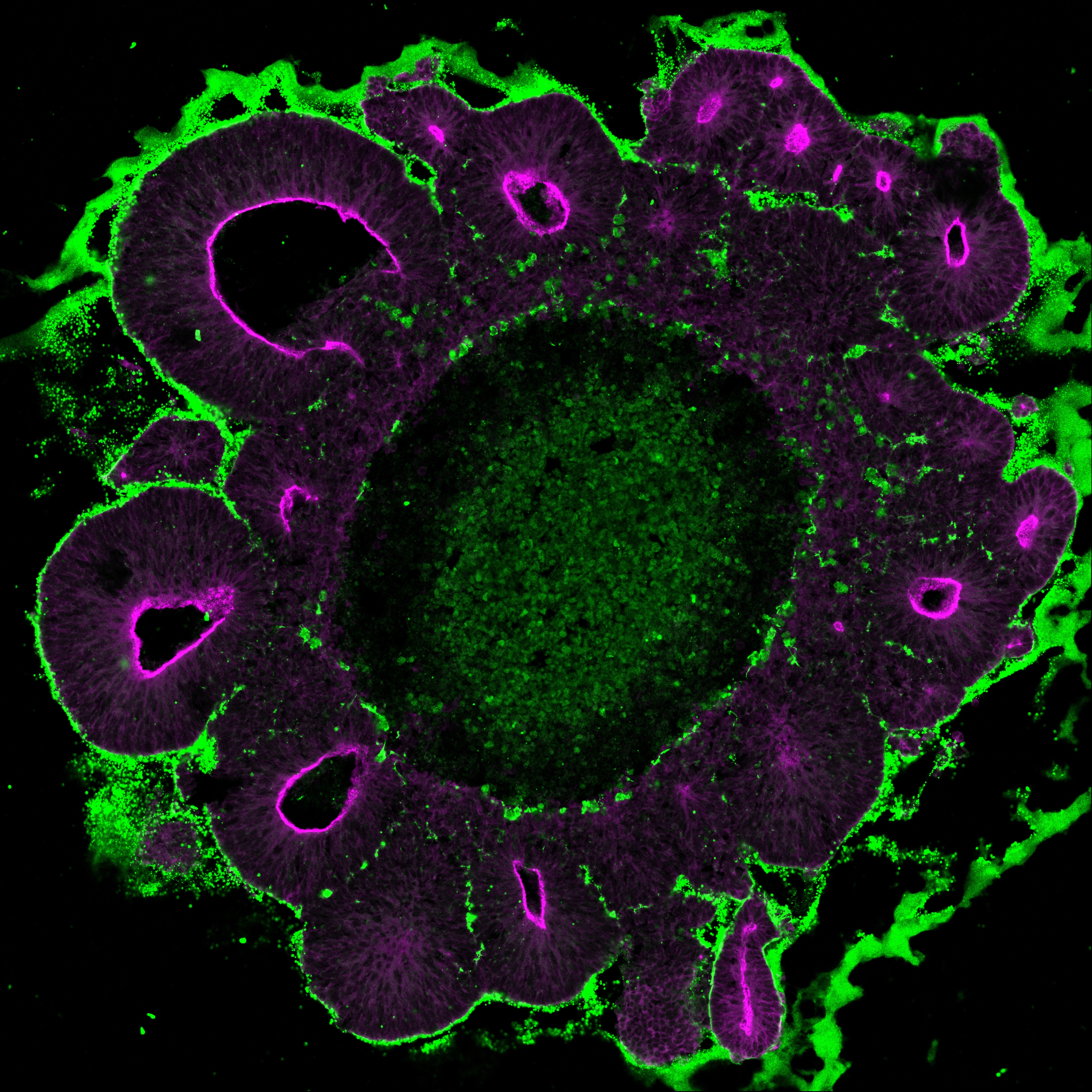

Supplement: Supplementary file 3 — Source Data for Figure 2 [file EMBJ-42-e113213-s006.zip › Figure2/Fig2G/Fig2G_H9_MGdrop_D20_greenFN-magentaPKC.jpg]

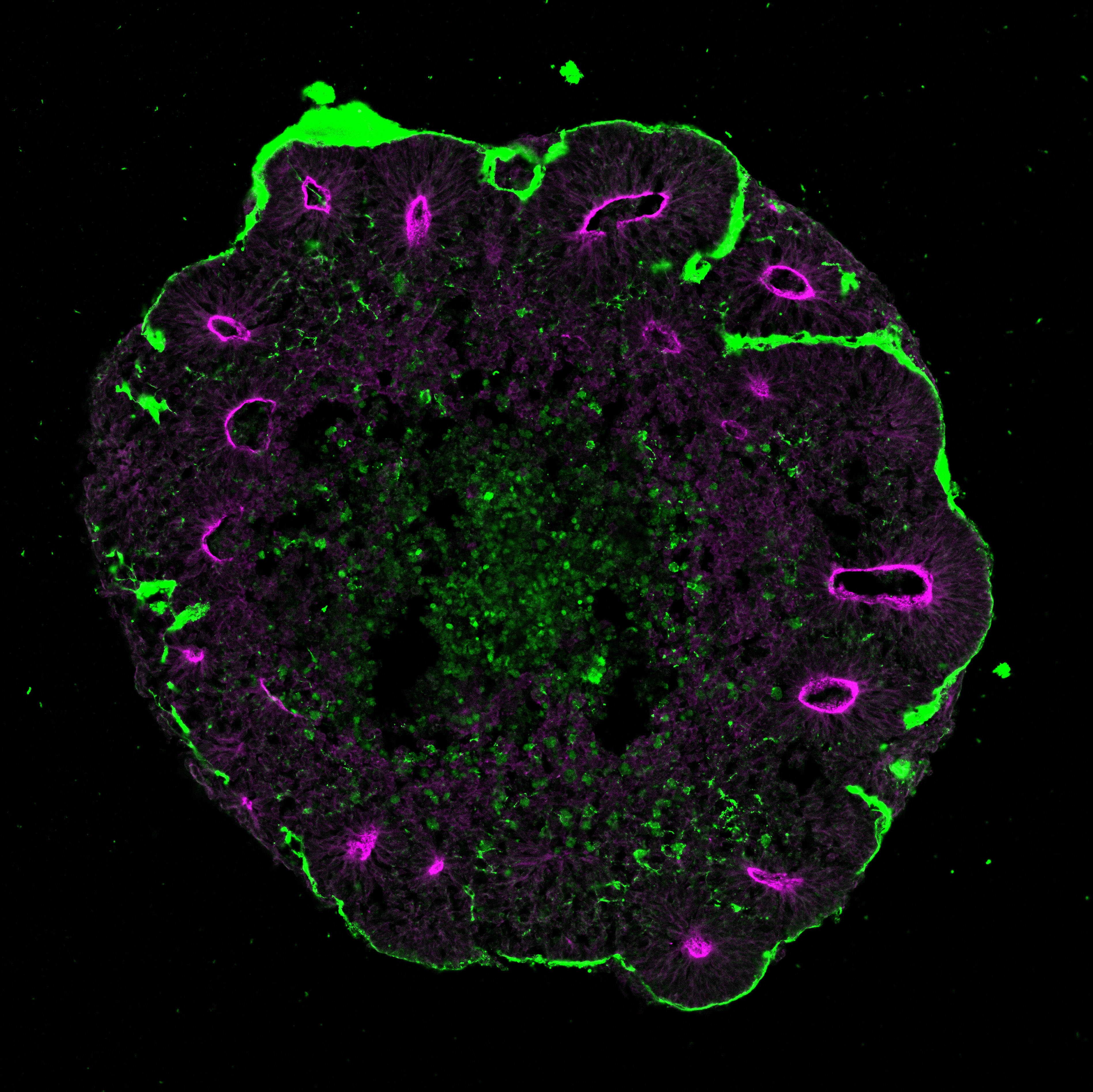

Supplement: Supplementary file 3 — Source Data for Figure 2 [file EMBJ-42-e113213-s006.zip › Figure2/Fig2G/Fig2G_H9_MGliq_D20_greenFN-magentaPKC.jpg]

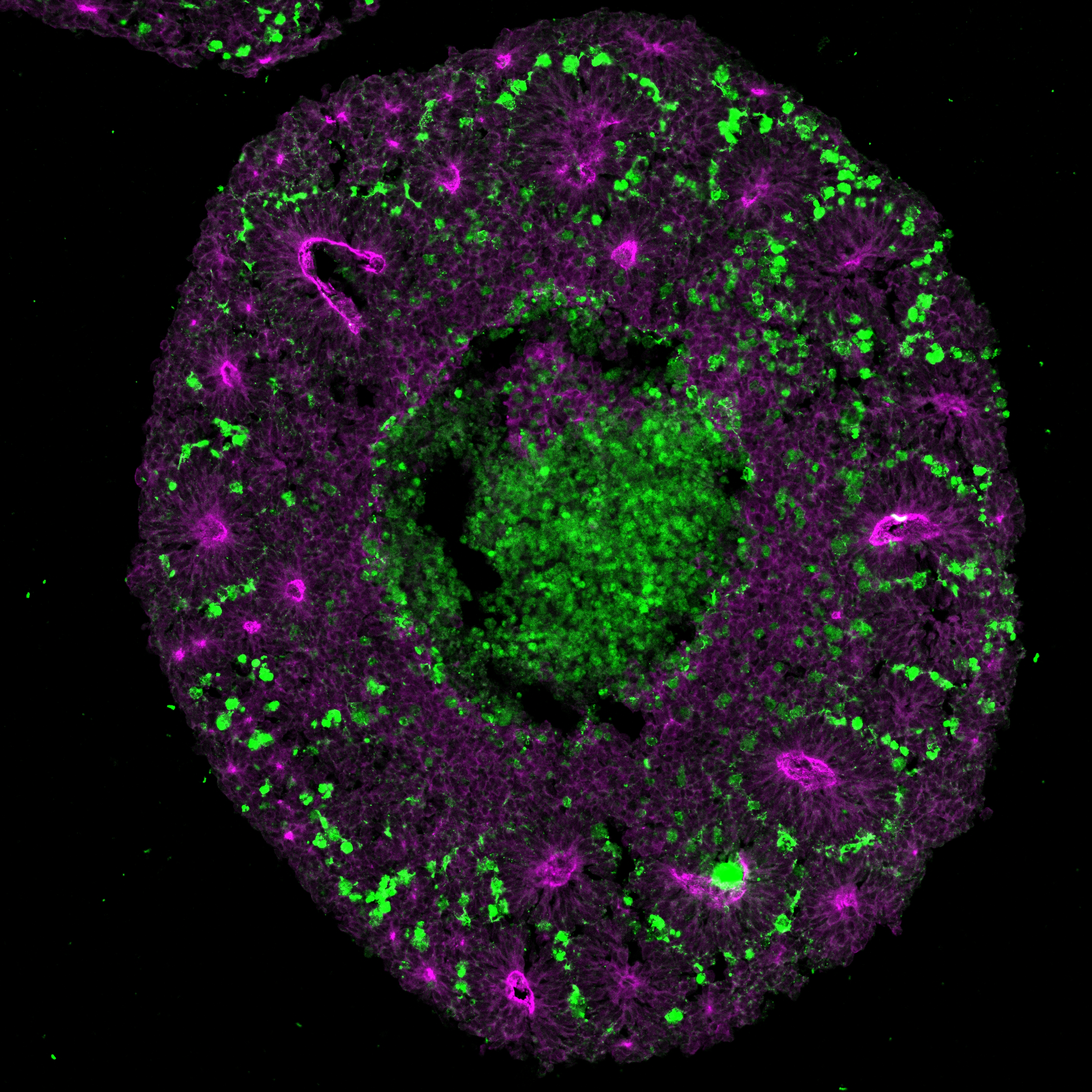

Supplement: Supplementary file 3 — Source Data for Figure 2 [file EMBJ-42-e113213-s006.zip › Figure2/Fig2G/Fig2G_H9_MGnull_D20_greenFN-magentaPKC.jpg]

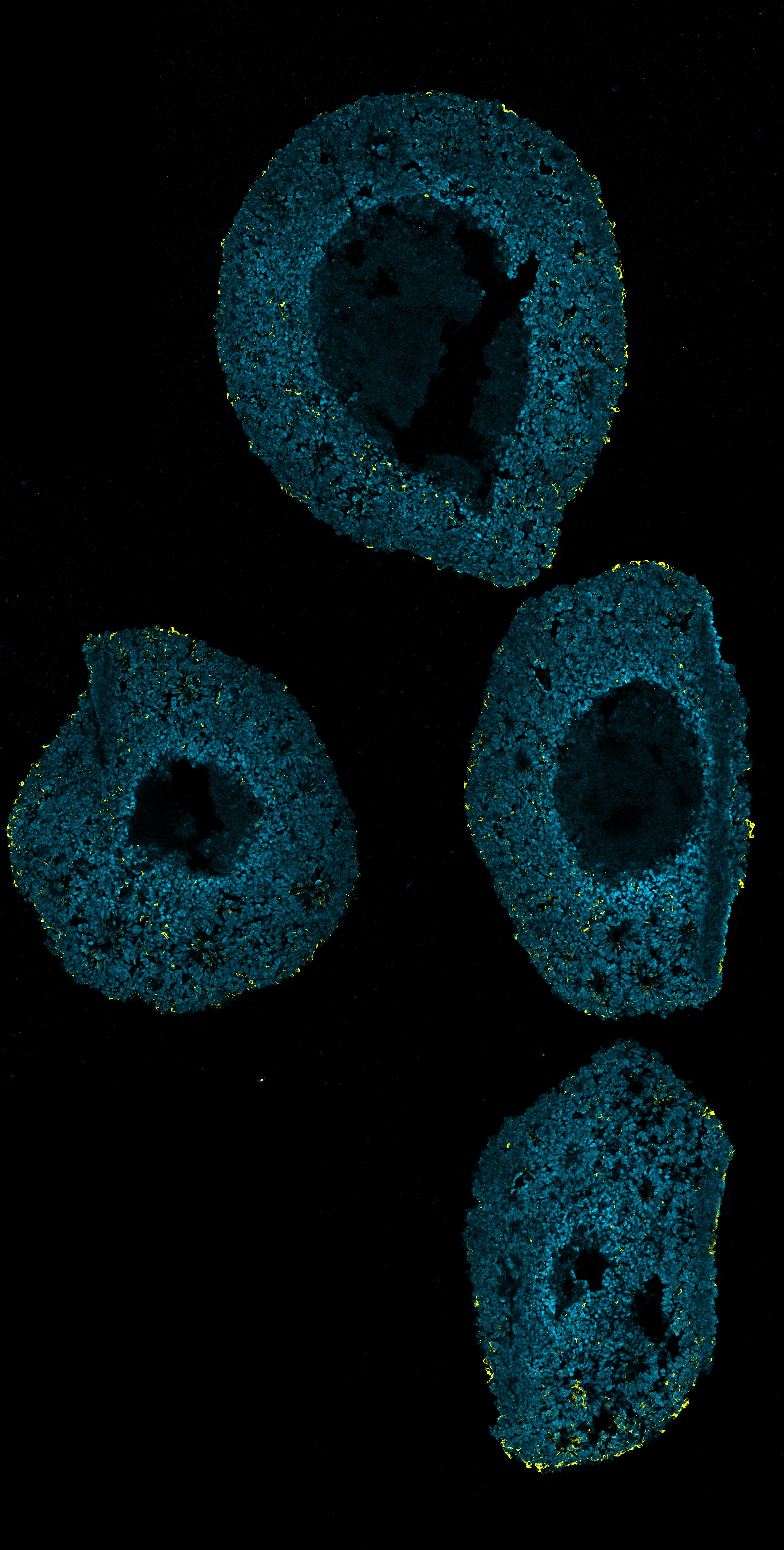

Supplement: Supplementary file 4 — Source Data for Figure 3 [file EMBJ-42-e113213-s002.zip › Figure3/Fig3B/Fig3B_H9_MGnull_D20_yellowMAP2-cyanSOX2.jpg]

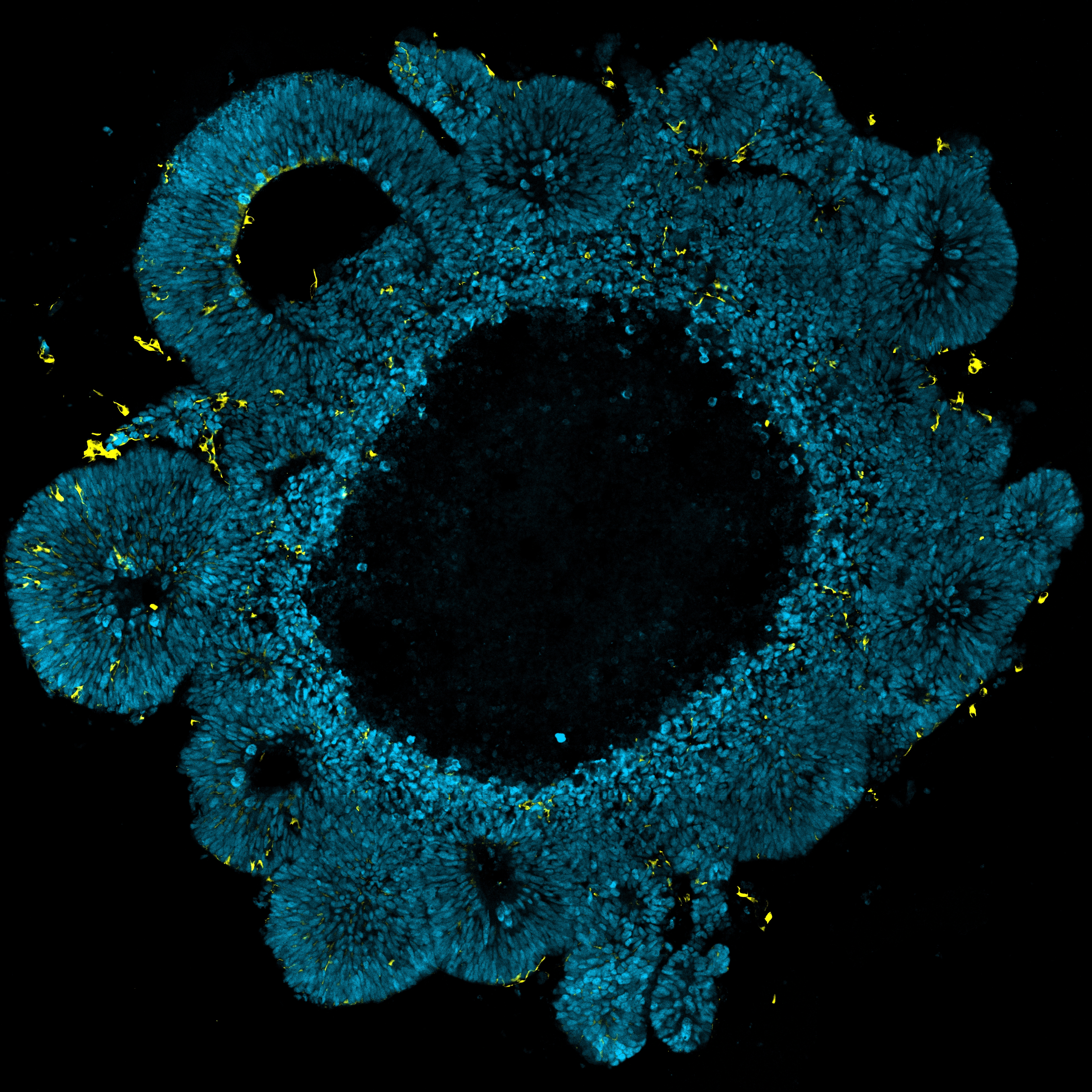

Supplement: Supplementary file 4 — Source Data for Figure 3 [file EMBJ-42-e113213-s002.zip › Figure3/Fig3B/Fig3B_H9_MGdrop_D20_yellowMAP2-cyanSOX2.jpg]

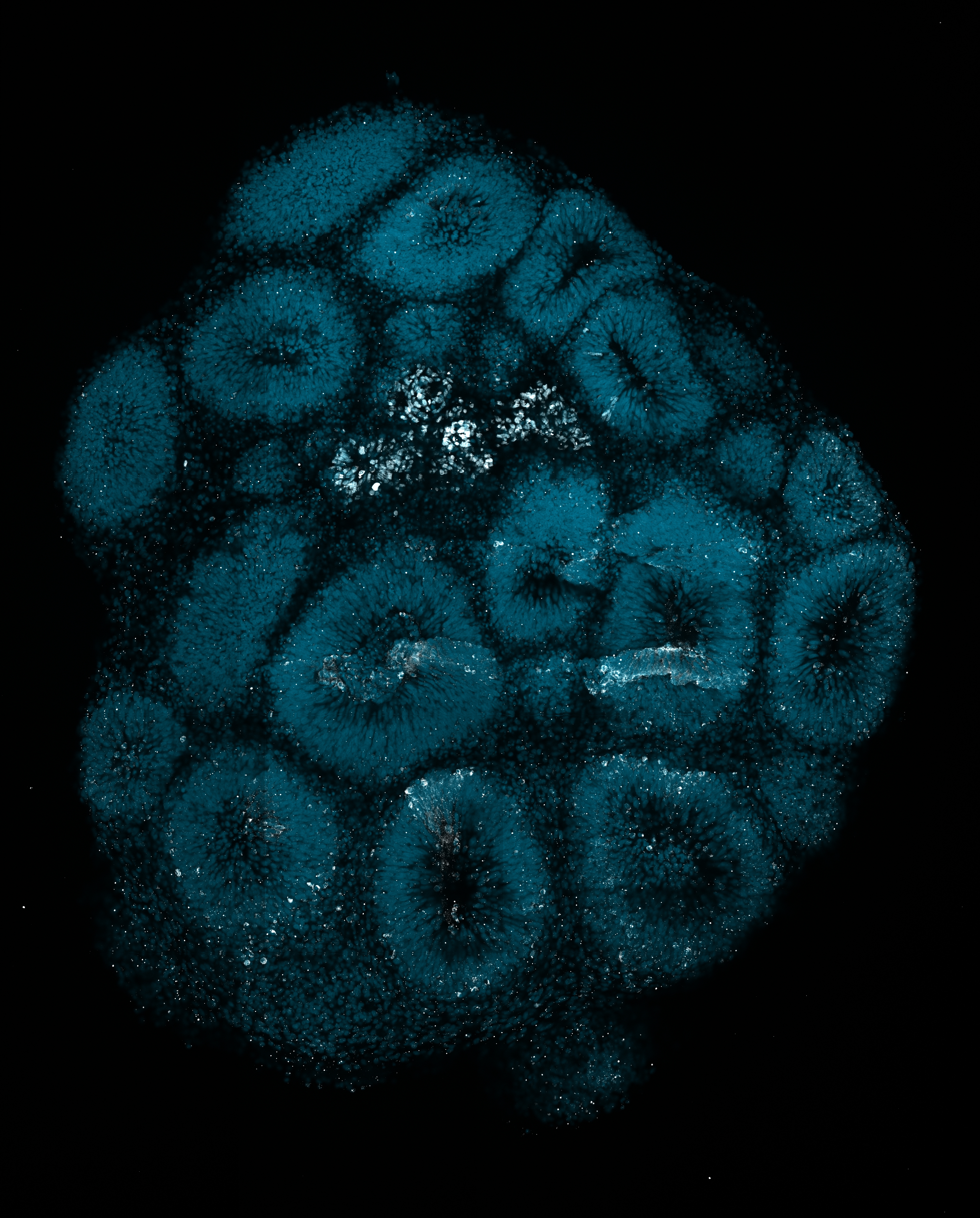

Supplement: Supplementary file 5 — Source Data for Figure 4 [file EMBJ-42-e113213-s001.zip › Figure4/Fig4D/Fig4D_H9_MGnull_D40_whiteOTX2-cyanDAPI.jpg]

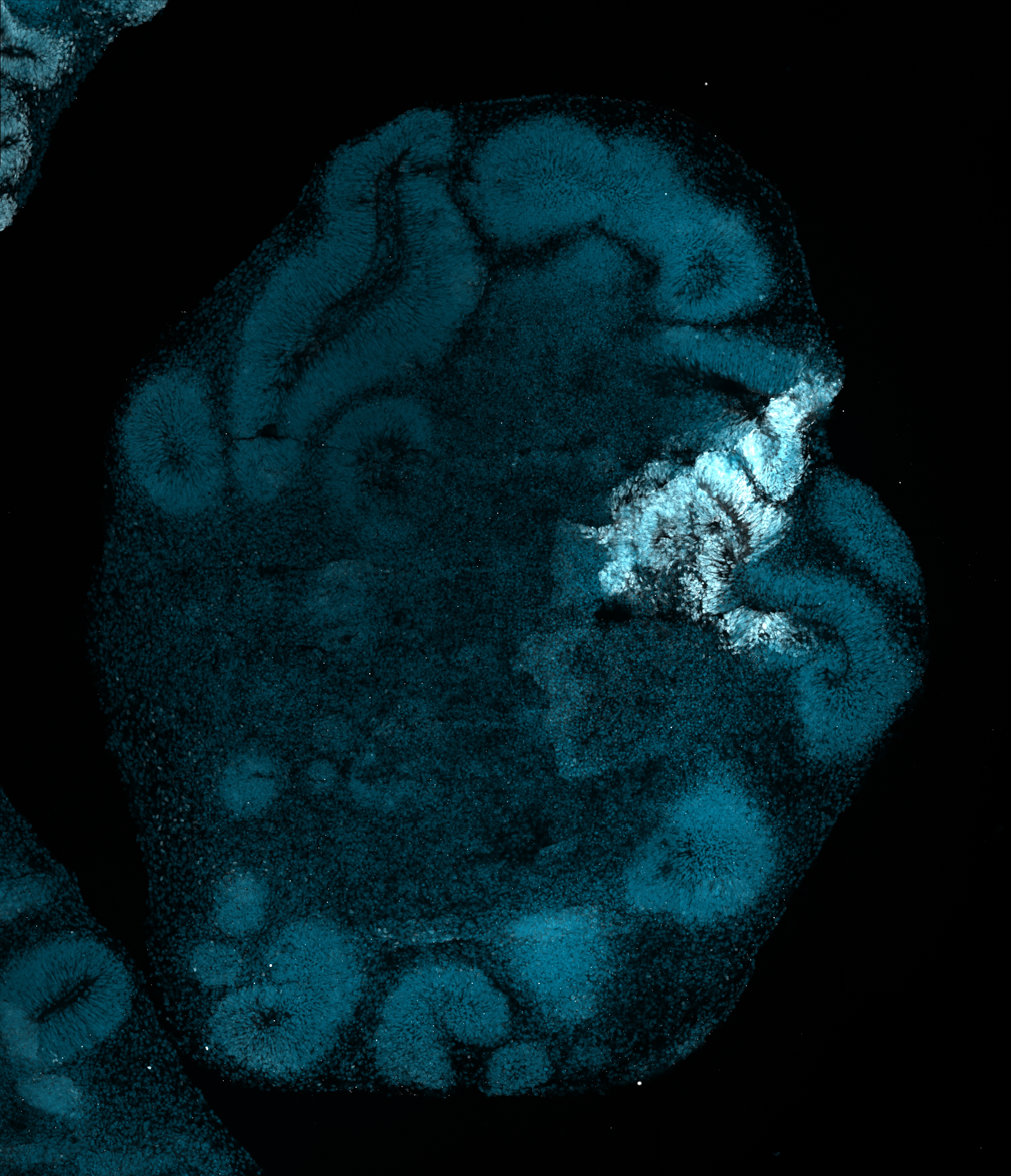

Supplement: Supplementary file 5 — Source Data for Figure 4 [file EMBJ-42-e113213-s001.zip › Figure4/Fig4D/Fig4D_H9_MGliq_D40_whiteOTX2-cyanDAPI.jpg]

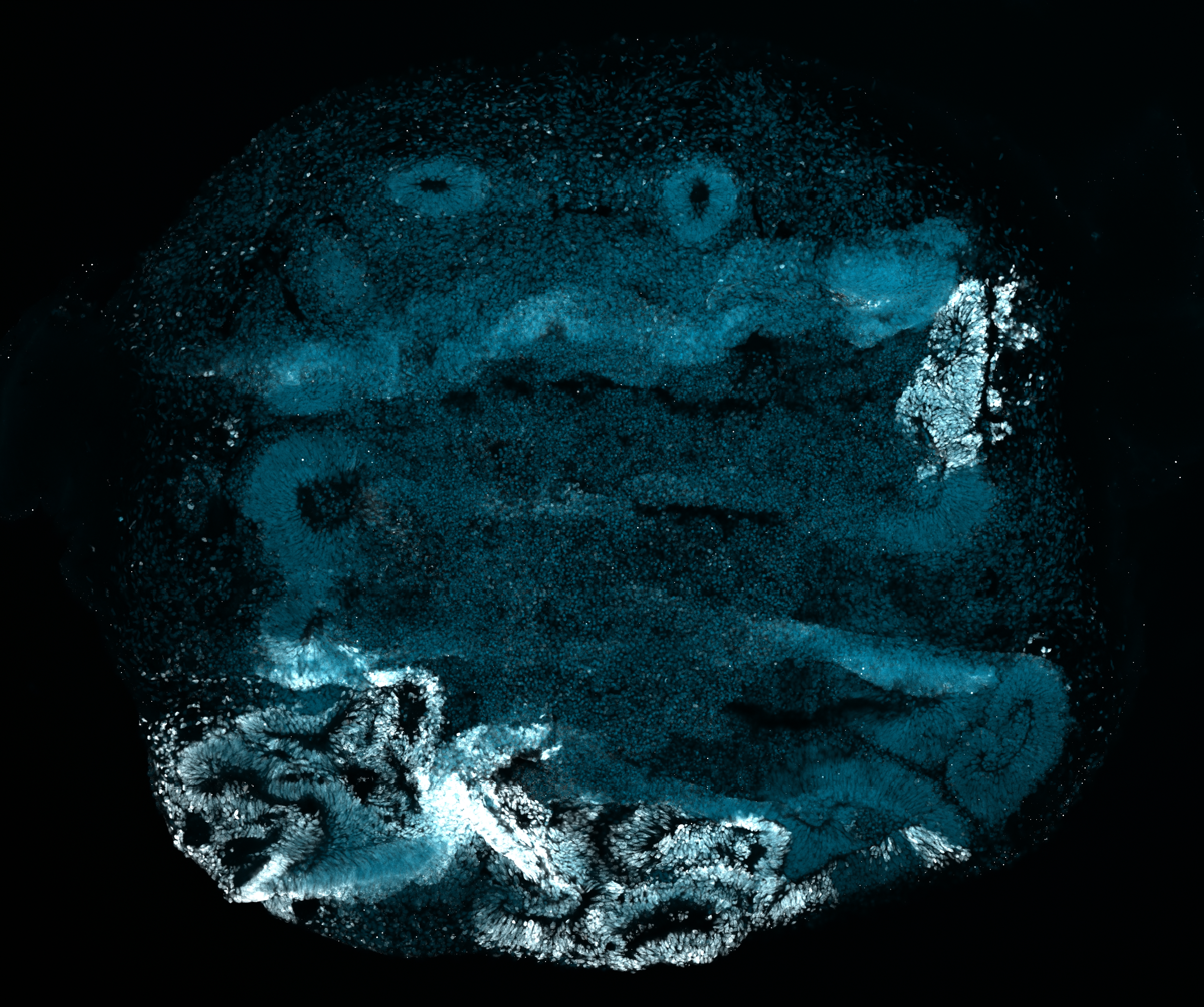

Supplement: Supplementary file 5 — Source Data for Figure 4 [file EMBJ-42-e113213-s001.zip › Figure4/Fig4D/Fig4D_H9_MGdrop_D40_whiteOTX2-cyanDAPI.jpg]

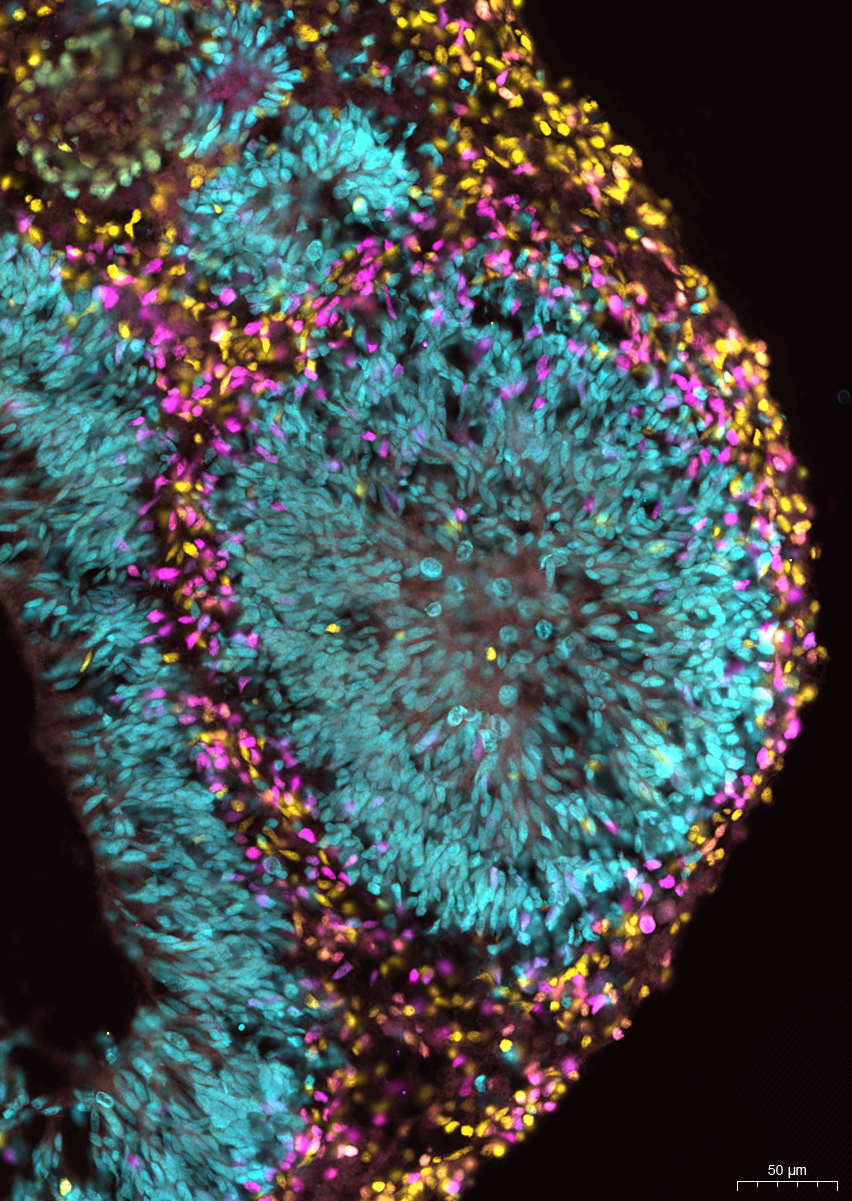

Supplement: Supplementary file 5 — Source Data for Figure 4 [file EMBJ-42-e113213-s001.zip › Figure4/Fig4C/Fig4C_H9_MGdrop_D40_cyanSOX2-magentaTBR2-yellowCTIP2.jpg]

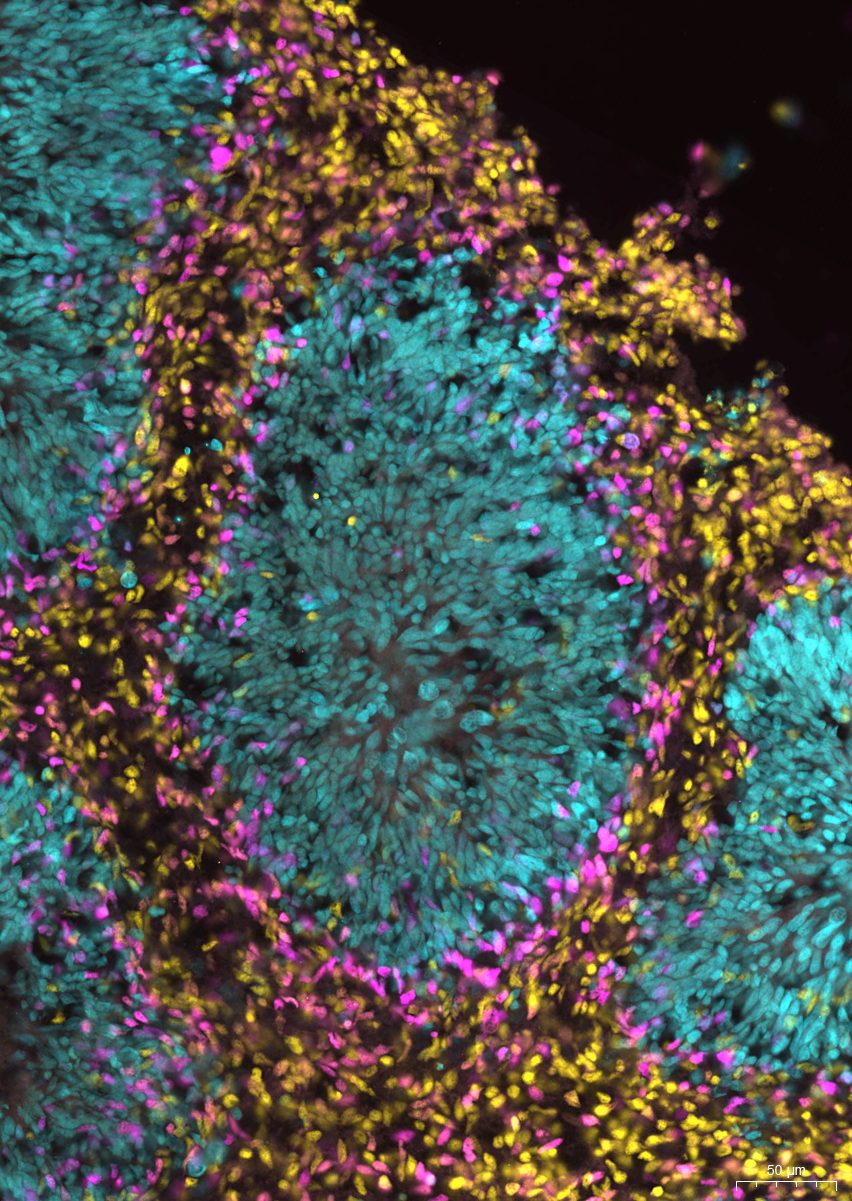

Supplement: Supplementary file 5 — Source Data for Figure 4 [file EMBJ-42-e113213-s001.zip › Figure4/Fig4C/Fig4C_H9_MGnull_D40_cyanSOX2-magentaTBR2-yellowCTIP2.jpg]

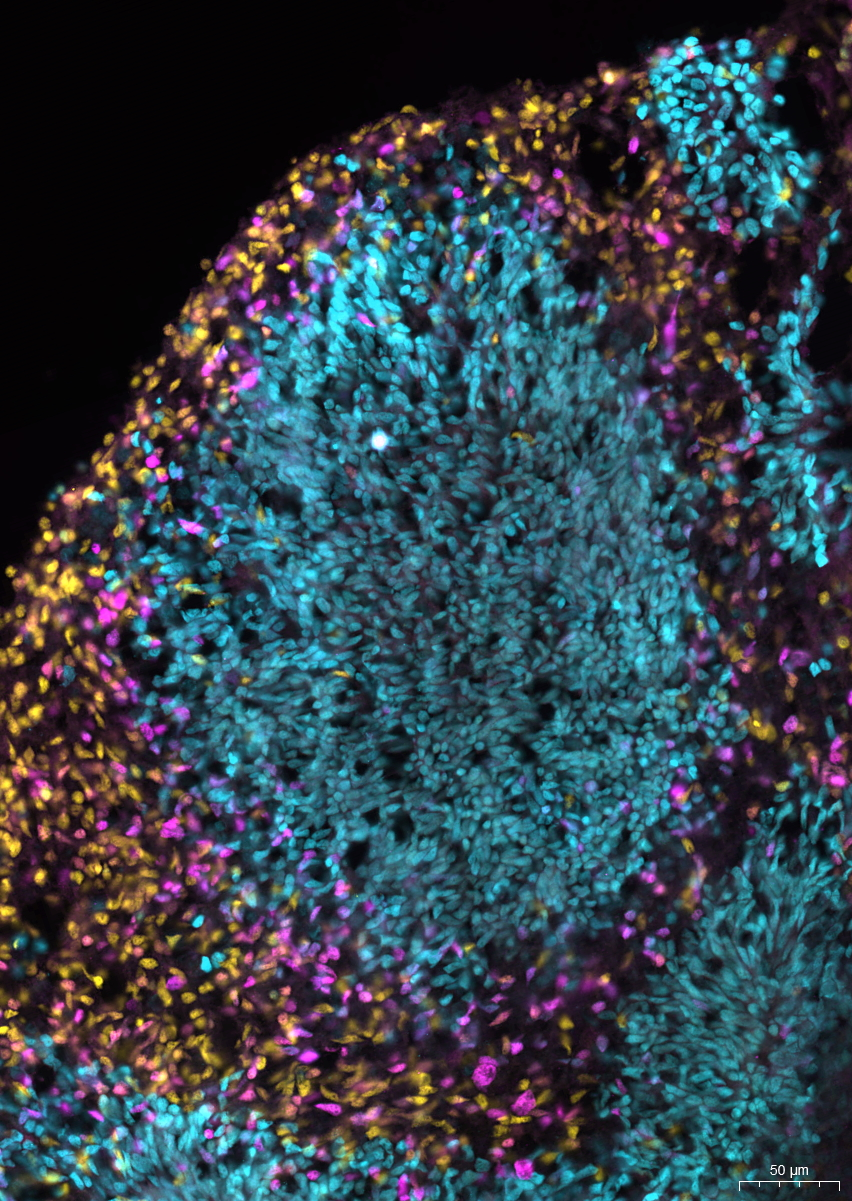

Supplement: Supplementary file 5 — Source Data for Figure 4 [file EMBJ-42-e113213-s001.zip › Figure4/Fig4C/Fig4C_H9_MGliq_D40_cyanSOX2-magentaTBR2-yellowCTIP2.jpg]

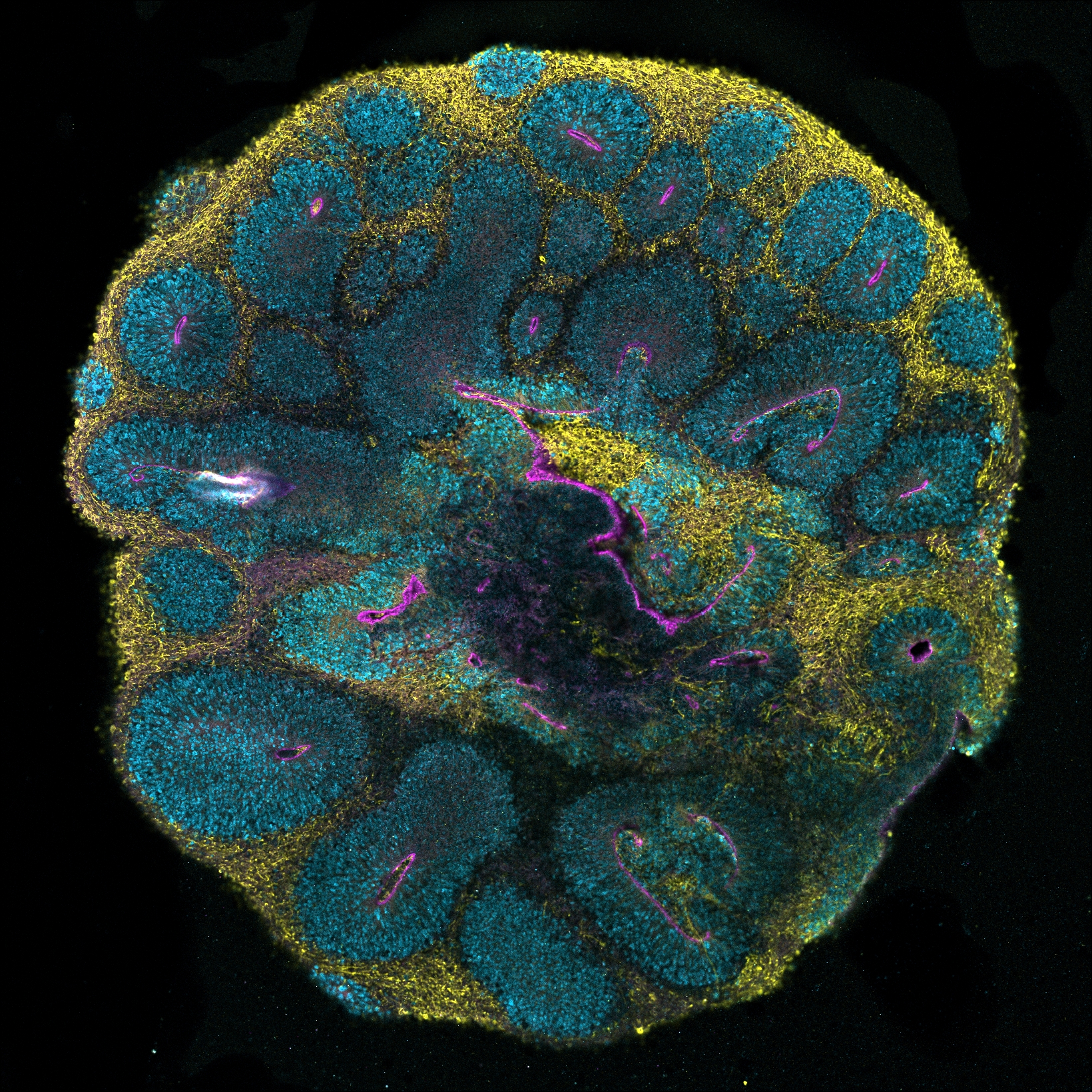

Supplement: Supplementary file 5 — Source Data for Figure 4 [file EMBJ-42-e113213-s001.zip › Figure4/Fig4B/Fig4B_H9_MGliq_D40_magentaPKC-cyanSOX2-yellowMAP2.jpg]

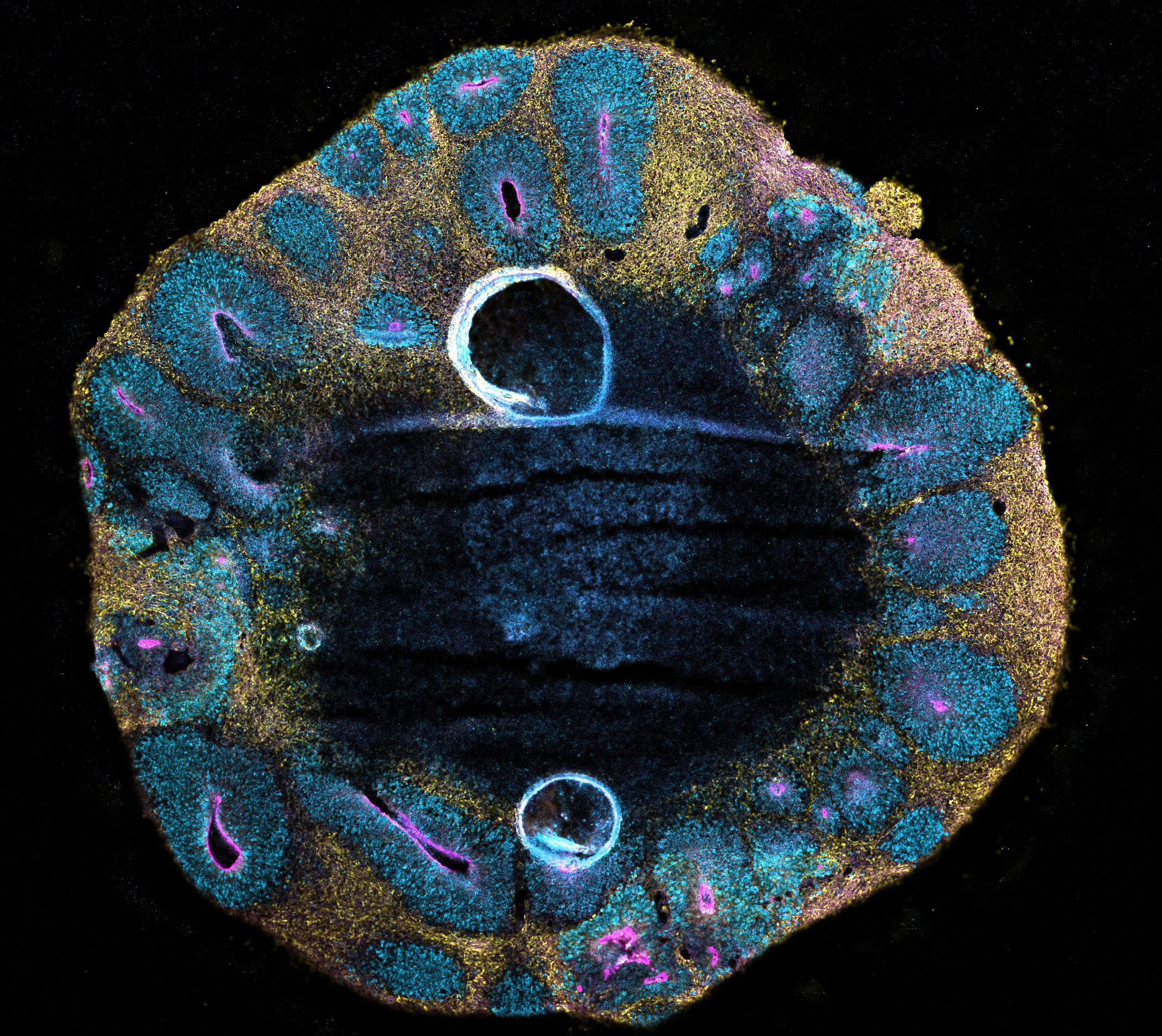

Supplement: Supplementary file 5 — Source Data for Figure 4 [file EMBJ-42-e113213-s001.zip › Figure4/Fig4B/Fig4B_H9_MGdrop_D40_magentaPKC-cyanSOX2-yellowMAP2.jpg]

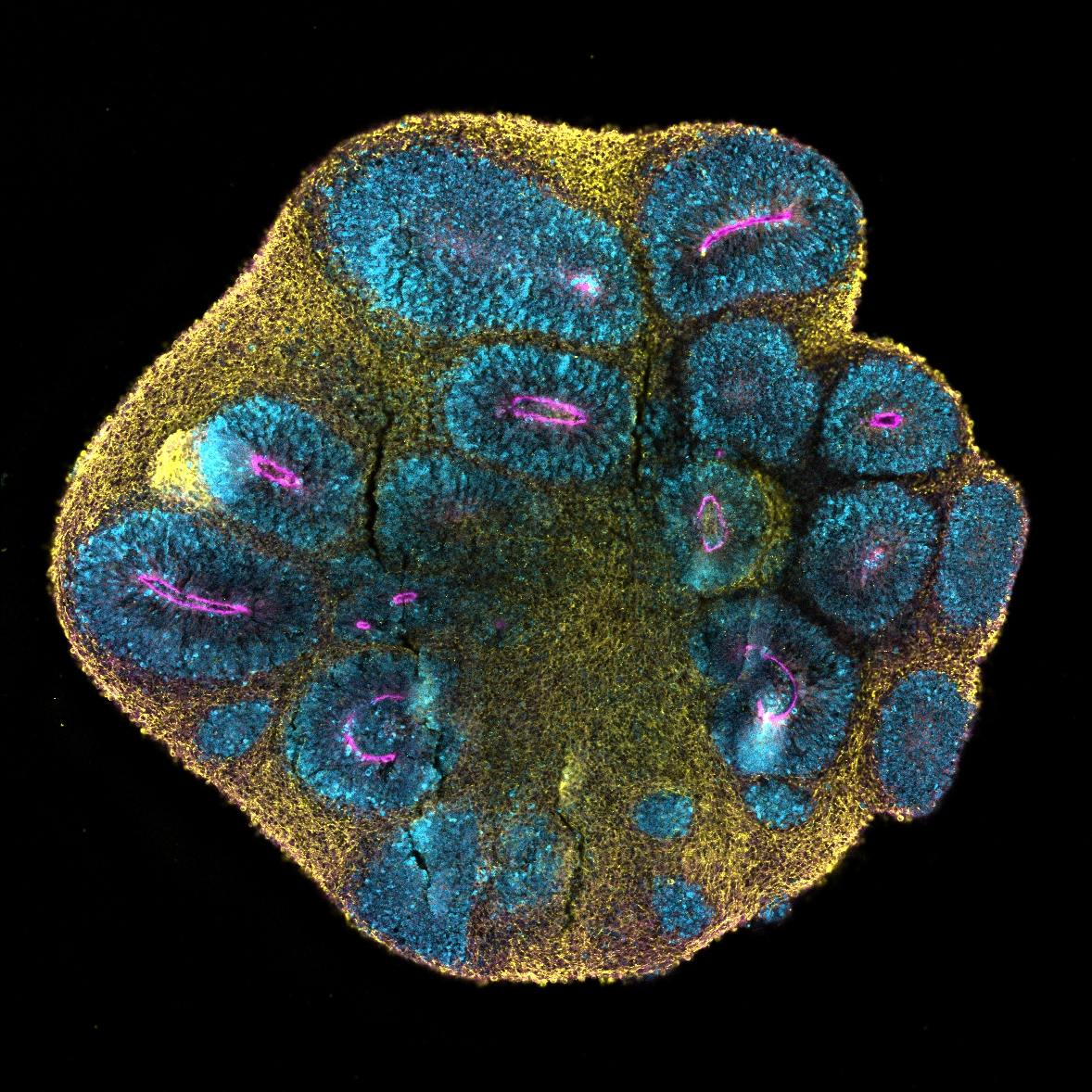

Supplement: Supplementary file 5 — Source Data for Figure 4 [file EMBJ-42-e113213-s001.zip › Figure4/Fig4B/Fig4B_H9_MGnull_D40_magentaPKC-cyanSOX2-yellowMAP2.jpg]

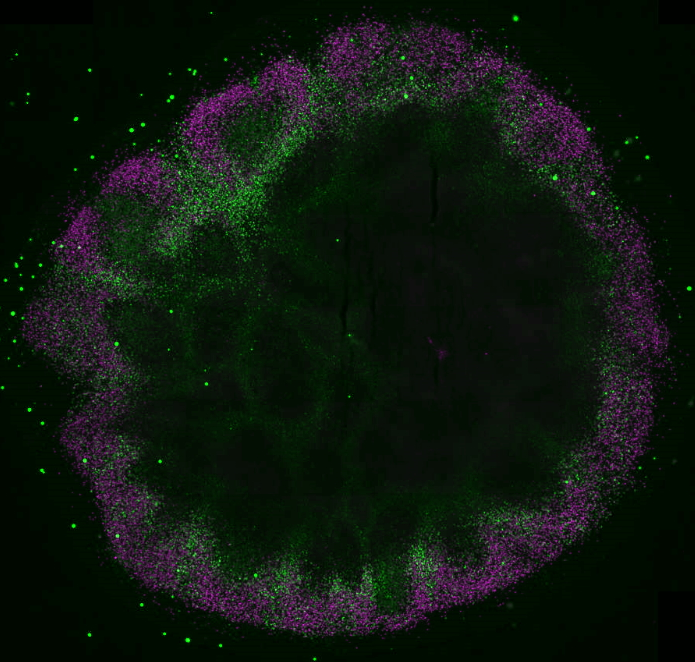

Supplement: Supplementary file 6 — Source Data for Figure 5 [file EMBJ-42-e113213-s007.zip › Figure5/Fig5I/SATB2-CTIP2/Fig5I_H9_D120_MGliq_greenCTIP2-magentaSATB2.jpg]

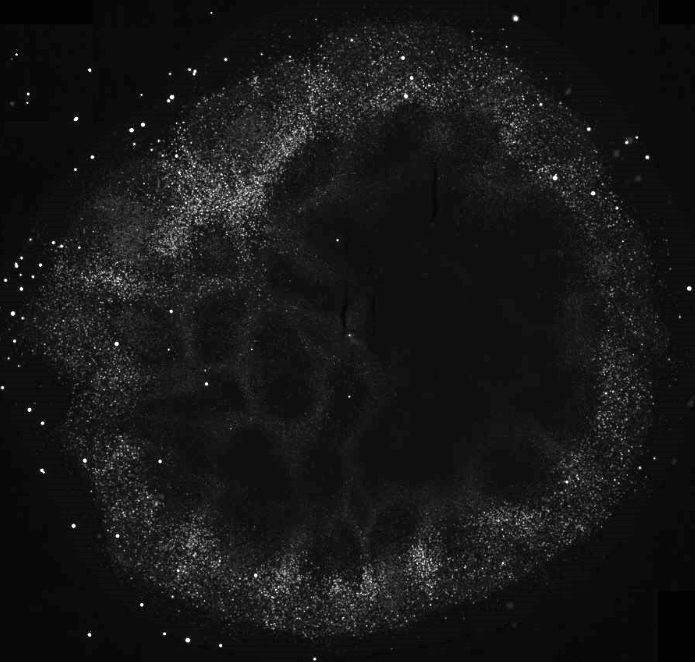

Supplement: Supplementary file 6 — Source Data for Figure 5 [file EMBJ-42-e113213-s007.zip › Figure5/Fig5I/SATB2-CTIP2/Fig5I_H9_D120_MGliq_CTIP2.jpg]

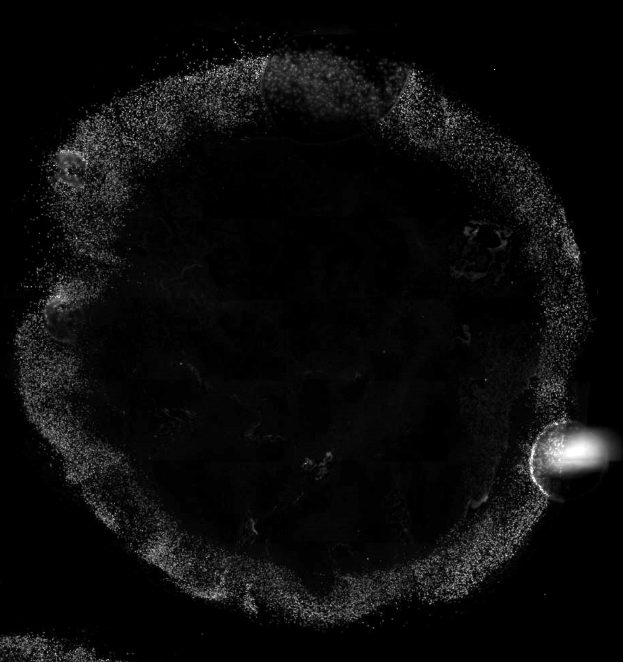

Supplement: Supplementary file 6 — Source Data for Figure 5 [file EMBJ-42-e113213-s007.zip › Figure5/Fig5I/SATB2-CTIP2/Fig5I_H9_D120_MGdrop_SATB2.jpg]

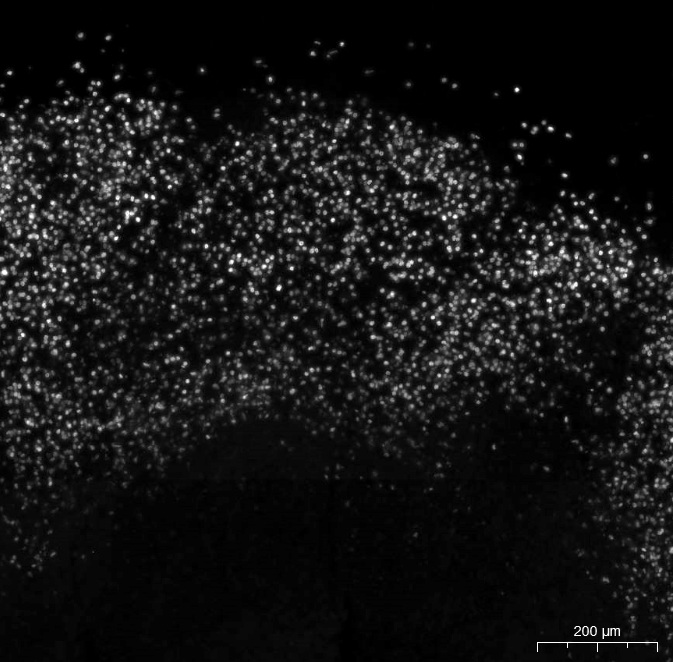

Supplement: Supplementary file 6 — Source Data for Figure 5 [file EMBJ-42-e113213-s007.zip › Figure5/Fig5I/SATB2-CTIP2/Fig5I_H9_D120_MGliq_SATB2_zoom.jpg]

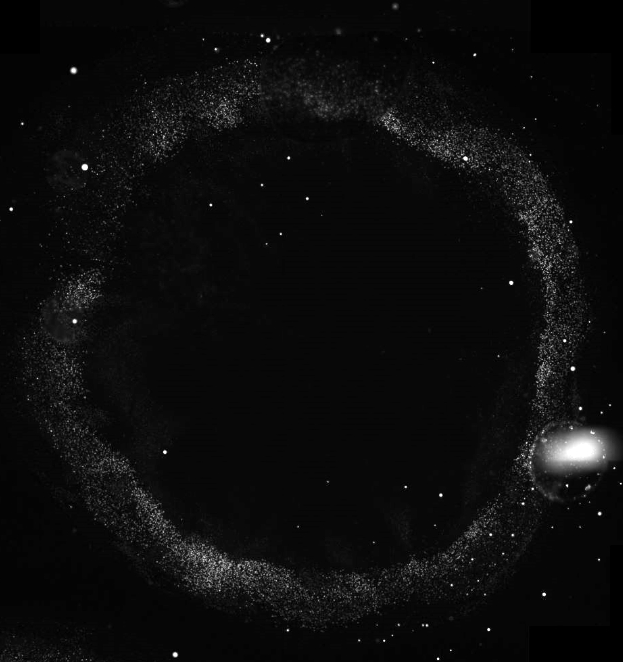

Supplement: Supplementary file 6 — Source Data for Figure 5 [file EMBJ-42-e113213-s007.zip › Figure5/Fig5I/SATB2-CTIP2/Fig5I_H9_D120_MGdrop_CTIP2.jpg]

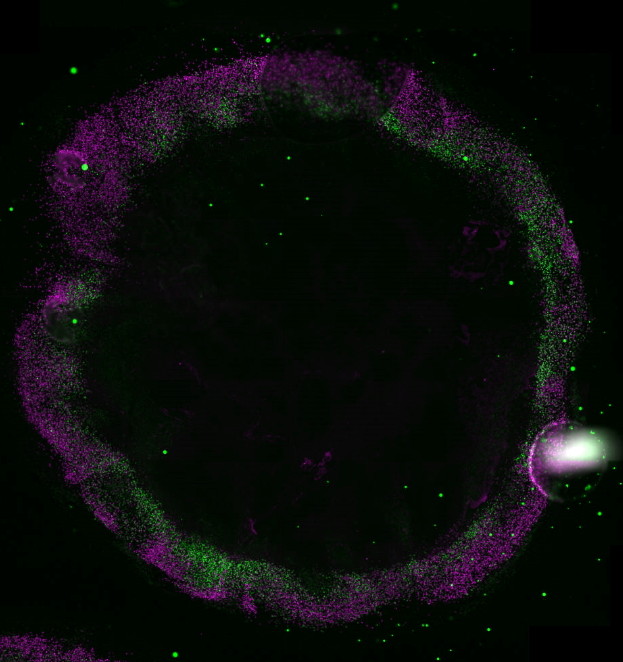

Supplement: Supplementary file 6 — Source Data for Figure 5 [file EMBJ-42-e113213-s007.zip › Figure5/Fig5I/SATB2-CTIP2/Fig5I_H9_D120_MGdrop_greenCTIP2-magentaSATB2.jpg]

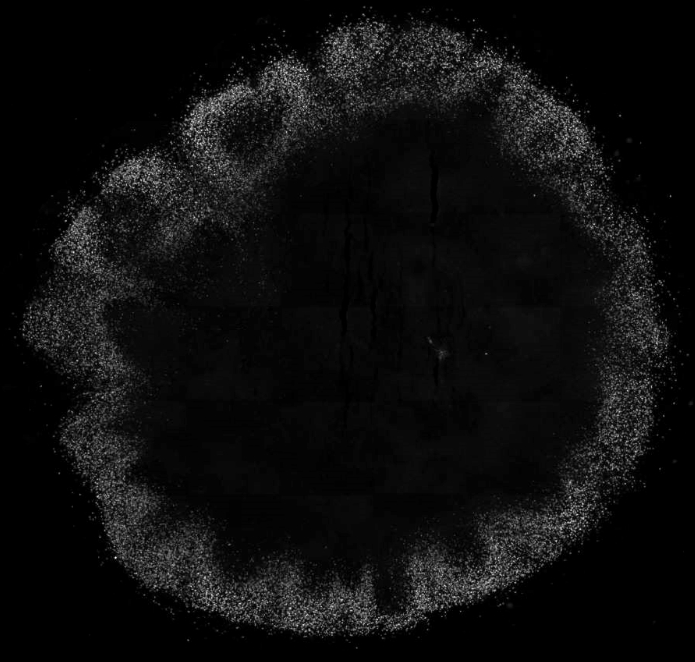

Supplement: Supplementary file 6 — Source Data for Figure 5 [file EMBJ-42-e113213-s007.zip › Figure5/Fig5I/SATB2-CTIP2/Fig5I_H9_D120_MGliq_SATB2.jpg]

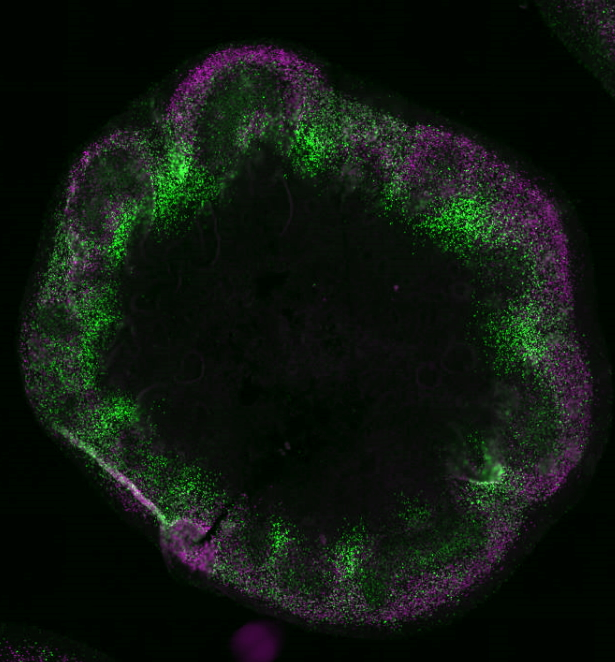

Supplement: Supplementary file 6 — Source Data for Figure 5 [file EMBJ-42-e113213-s007.zip › Figure5/Fig5I/SATB2-CTIP2/Fig5I_H9_D120_MGnull_greenCTIP2-magentaSATB2.jpg]

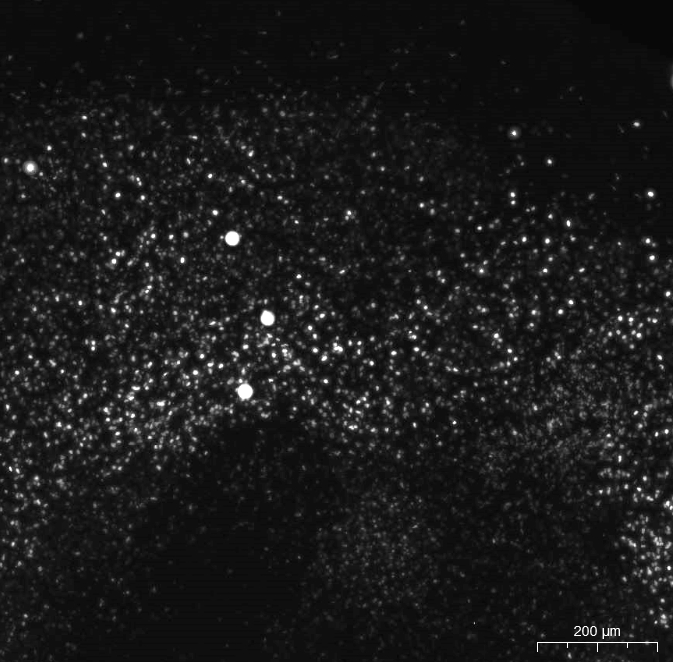

Supplement: Supplementary file 6 — Source Data for Figure 5 [file EMBJ-42-e113213-s007.zip › Figure5/Fig5I/SATB2-CTIP2/Fig5I_H9_D120_MGliq_CTIP2_zoom.jpg]

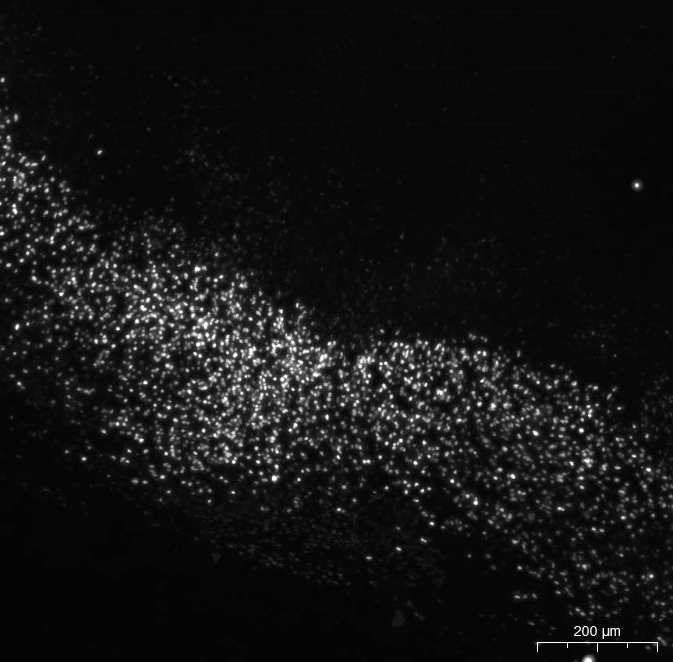

Supplement: Supplementary file 6 — Source Data for Figure 5 [file EMBJ-42-e113213-s007.zip › Figure5/Fig5I/SATB2-CTIP2/Fig5I_H9_D120_MGdrop_CTIP2_zoom.jpg]

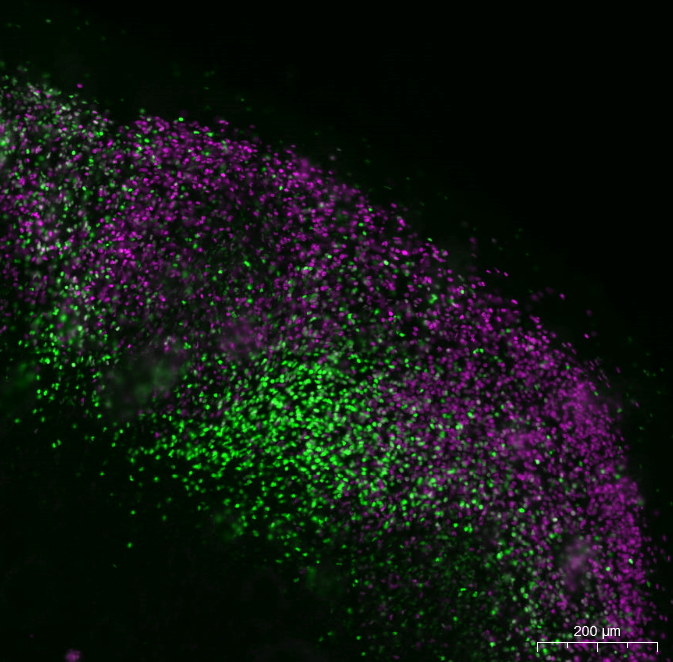

Supplement: Supplementary file 6 — Source Data for Figure 5 [file EMBJ-42-e113213-s007.zip › Figure5/Fig5I/SATB2-CTIP2/Fig5I_H9_D120_MGnull_greenCTIP2-magentaSATB2_zoom.jpg]

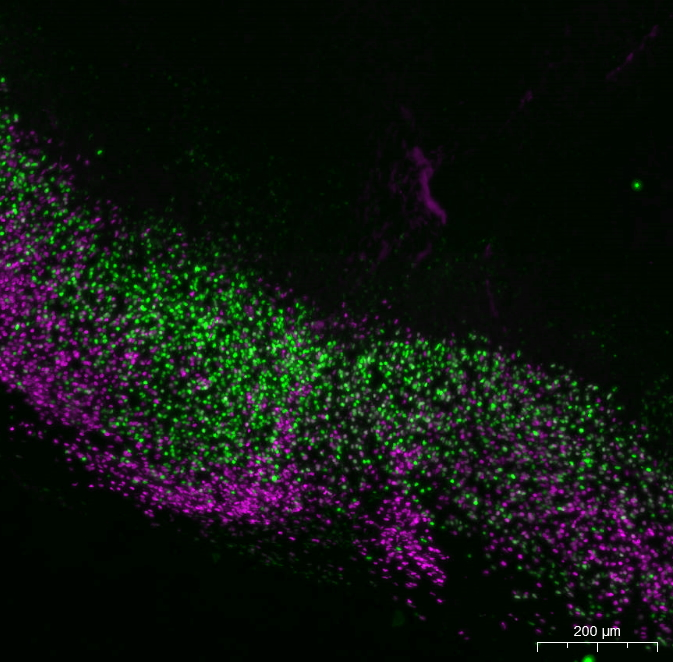

Supplement: Supplementary file 6 — Source Data for Figure 5 [file EMBJ-42-e113213-s007.zip › Figure5/Fig5I/SATB2-CTIP2/Fig5I_H9_D120_MGdrop_greenCTIP2-magentaSATB2_zoom.jpg]

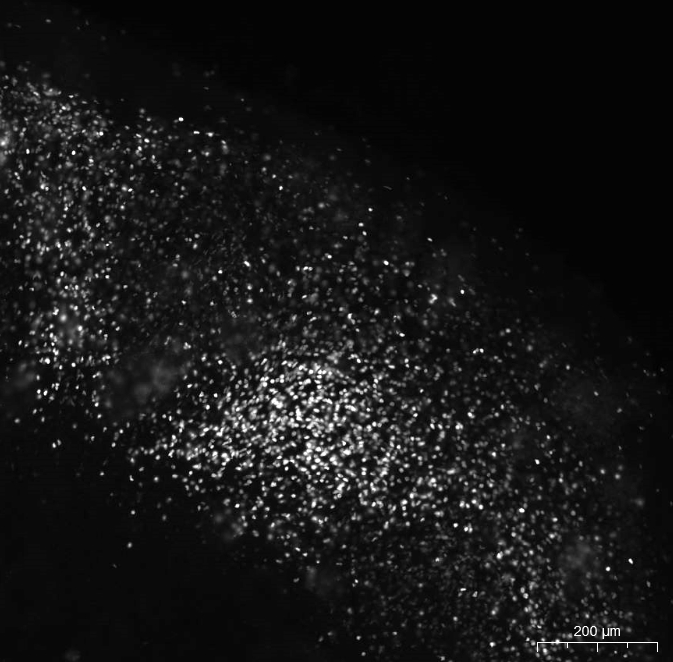

Supplement: Supplementary file 6 — Source Data for Figure 5 [file EMBJ-42-e113213-s007.zip › Figure5/Fig5I/SATB2-CTIP2/Fig5I_H9_D120_MGnull_CTIP2_zoom.jpg]

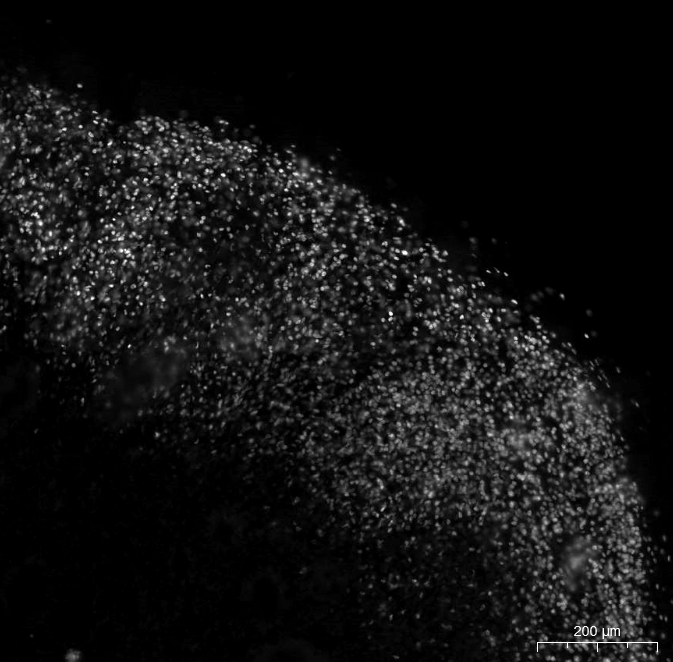

Supplement: Supplementary file 6 — Source Data for Figure 5 [file EMBJ-42-e113213-s007.zip › Figure5/Fig5I/SATB2-CTIP2/Fig5I_H9_D120_MGnull_SATB2_zoom.jpg]

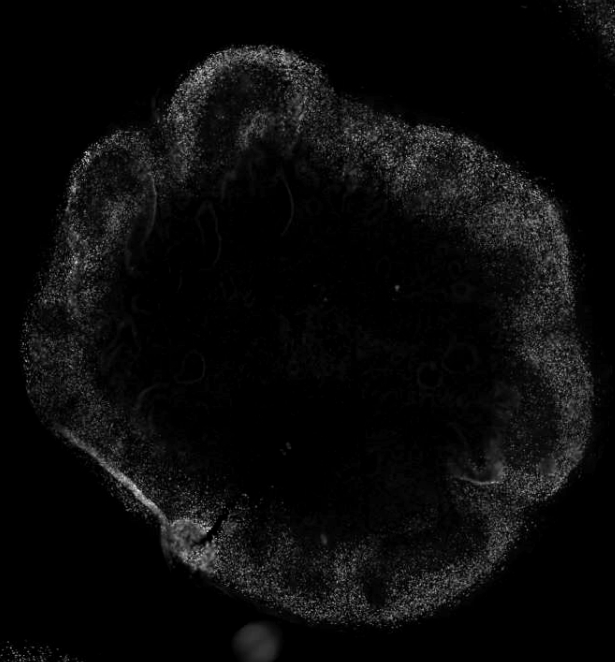

Supplement: Supplementary file 6 — Source Data for Figure 5 [file EMBJ-42-e113213-s007.zip › Figure5/Fig5I/SATB2-CTIP2/Fig5I_H9_D120_MGnull_SATB2.jpg]

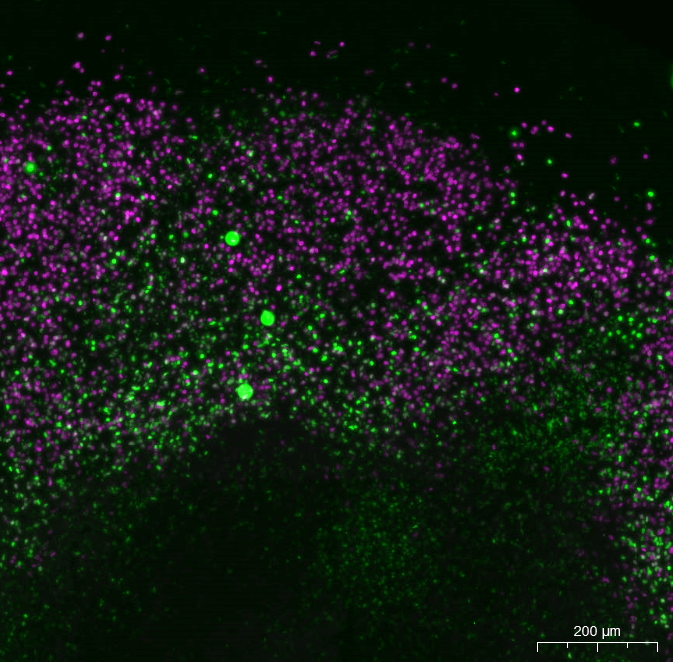

Supplement: Supplementary file 6 — Source Data for Figure 5 [file EMBJ-42-e113213-s007.zip › Figure5/Fig5I/SATB2-CTIP2/Fig5I_H9_D120_MGliq_greenCTIP2-magentaSATB2_zoom.jpg]

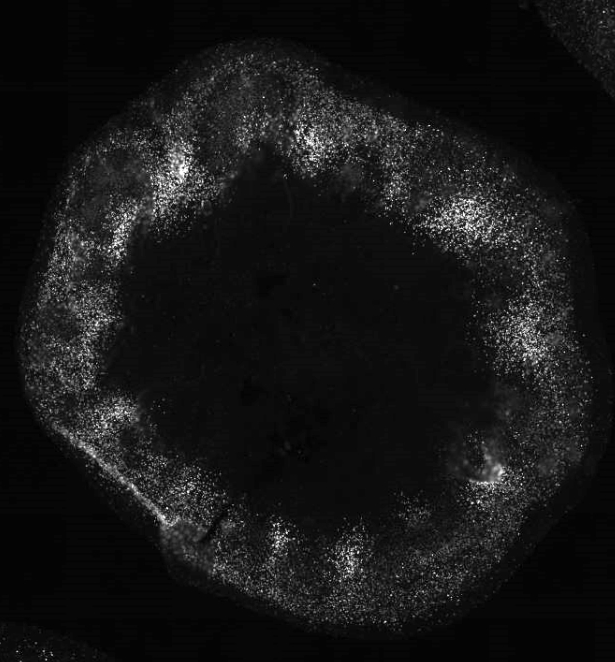

Supplement: Supplementary file 6 — Source Data for Figure 5 [file EMBJ-42-e113213-s007.zip › Figure5/Fig5I/SATB2-CTIP2/Fig5I_H9_D120_MGnull_CTIP2.jpg]

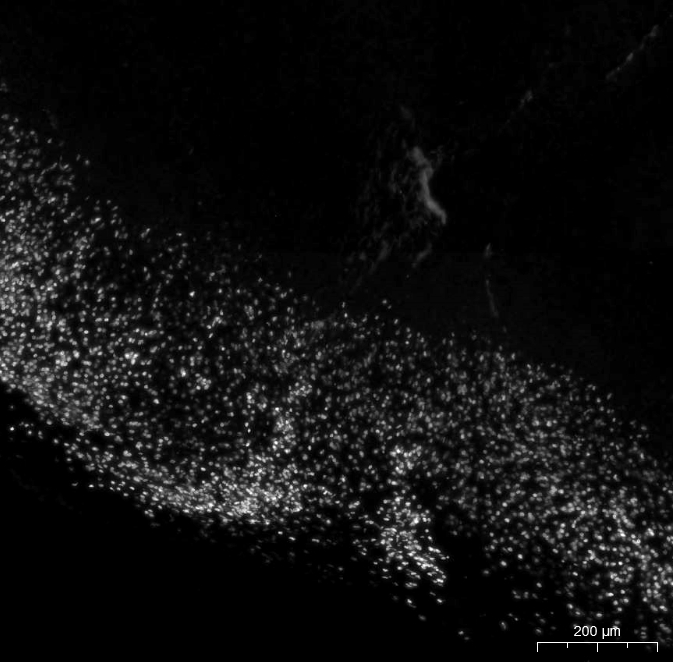

Supplement: Supplementary file 6 — Source Data for Figure 5 [file EMBJ-42-e113213-s007.zip › Figure5/Fig5I/SATB2-CTIP2/Fig5I_H9_D120_MGdrop_SATB2_zoom.jpg]

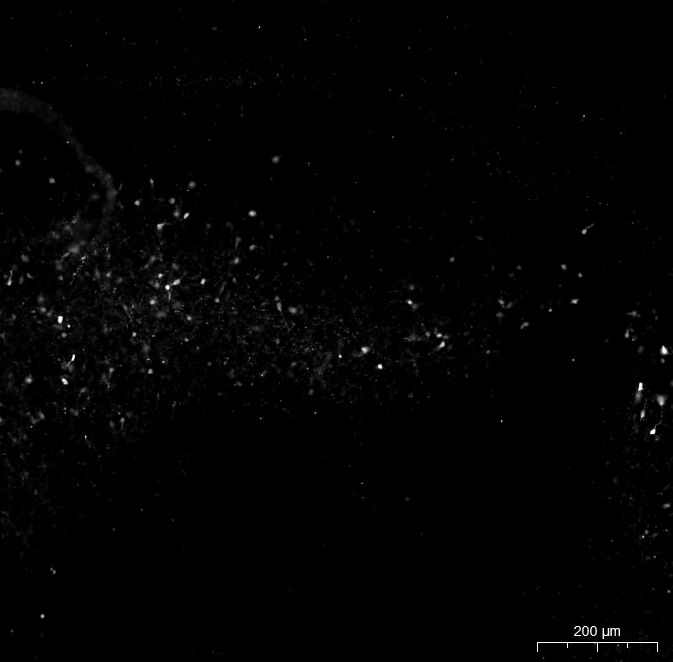

Supplement: Supplementary file 6 — Source Data for Figure 5 [file EMBJ-42-e113213-s007.zip › Figure5/Fig5I/SCGN-COUPTFii/Fig5I_H9_D120_MGliq_SCGN_zoom.jpg]

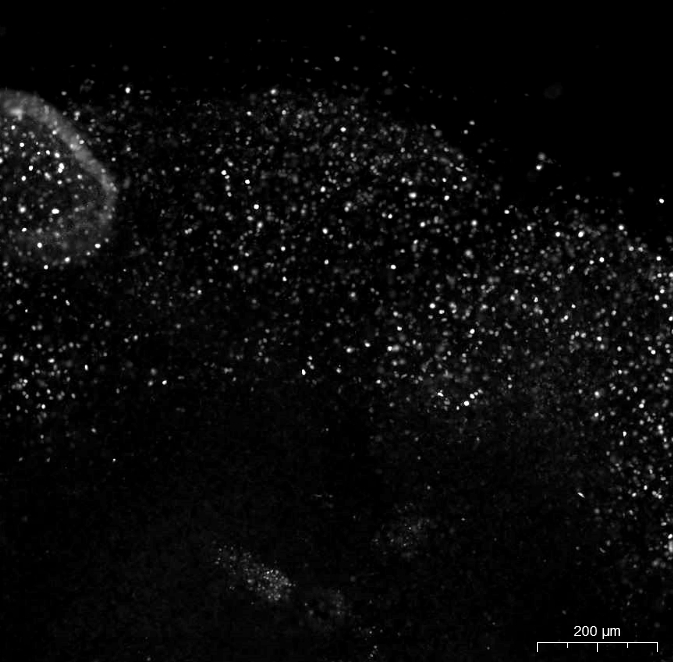

Supplement: Supplementary file 6 — Source Data for Figure 5 [file EMBJ-42-e113213-s007.zip › Figure5/Fig5I/SCGN-COUPTFii/Fig5I_H9_D120_MGliq_COUPTFii_zoom.jpg]

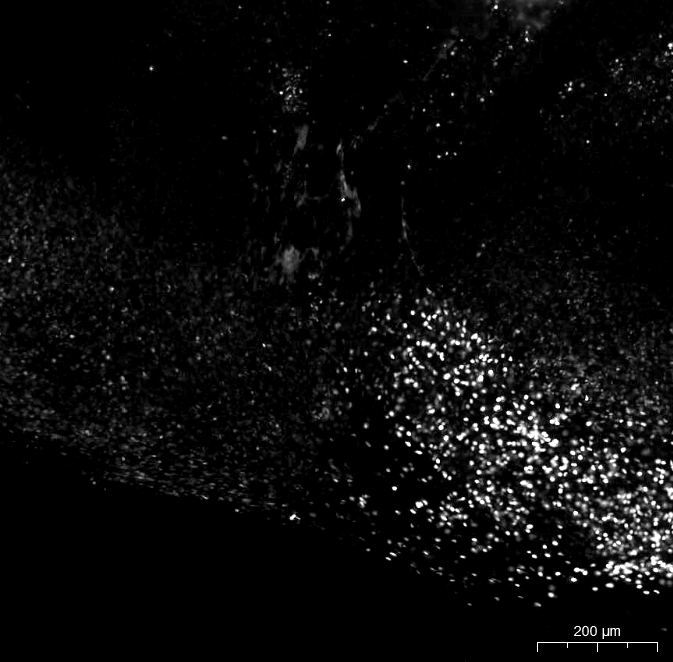

Supplement: Supplementary file 6 — Source Data for Figure 5 [file EMBJ-42-e113213-s007.zip › Figure5/Fig5I/SCGN-COUPTFii/Fig5I_H9_D120_MGdrop_COUPTFii_zoom.jpg]

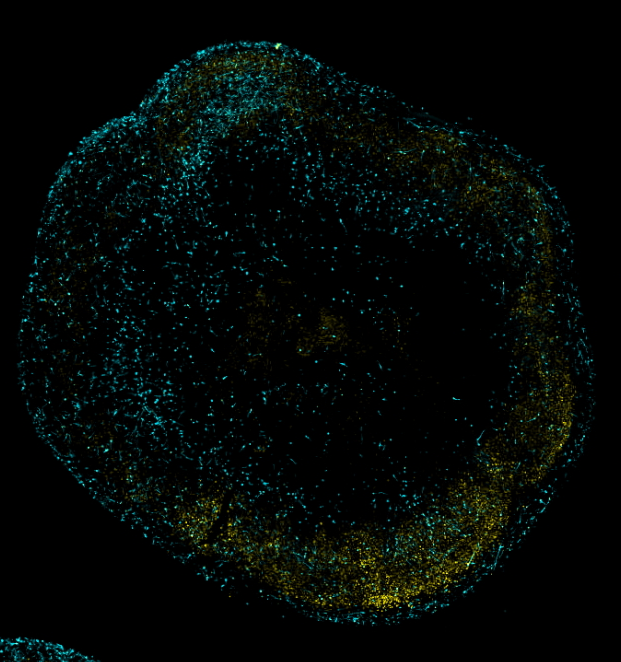

Supplement: Supplementary file 6 — Source Data for Figure 5 [file EMBJ-42-e113213-s007.zip › Figure5/Fig5I/SCGN-COUPTFii/Fig5I_H9_D120_MGnull_cyanSCGN-yellowCOUPTFii.jpg]

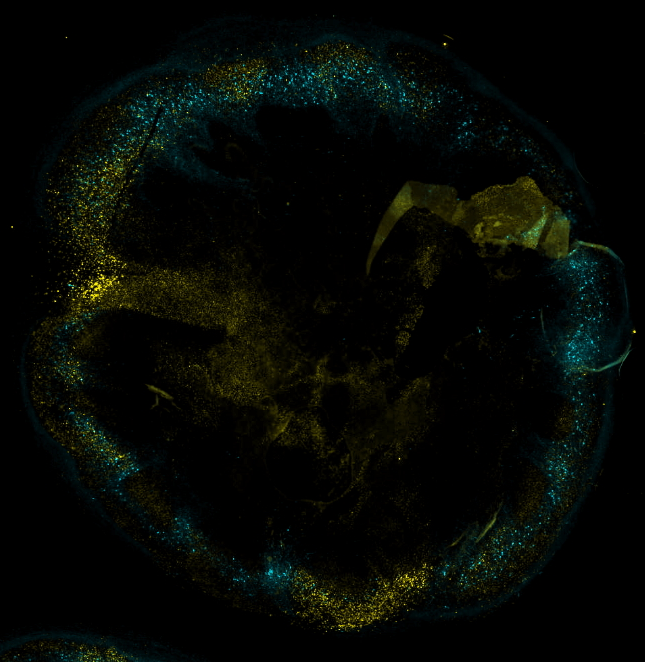

Supplement: Supplementary file 6 — Source Data for Figure 5 [file EMBJ-42-e113213-s007.zip › Figure5/Fig5I/SCGN-COUPTFii/Fig5I_H9_D120_MGdrop_cyanSCGN-yellowCOUPTFii.jpg]

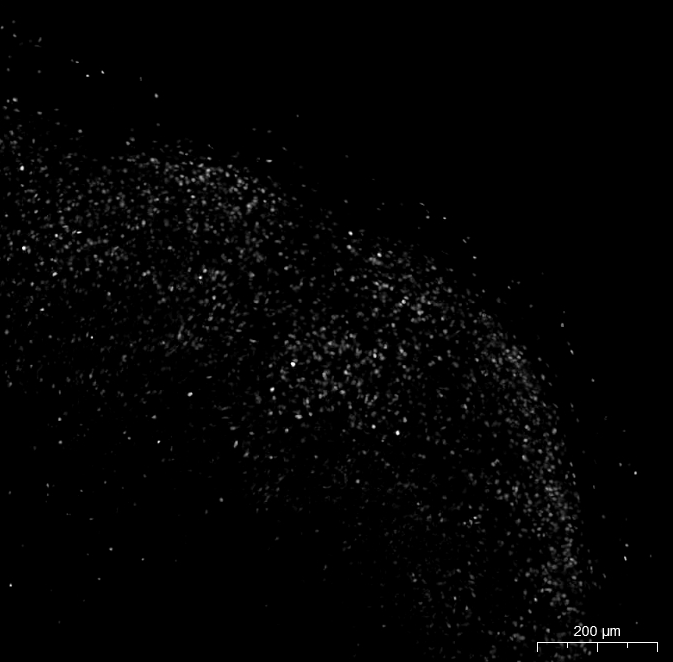

Supplement: Supplementary file 6 — Source Data for Figure 5 [file EMBJ-42-e113213-s007.zip › Figure5/Fig5I/SCGN-COUPTFii/Fig5I_H9_D120_MGnull_COUPTFii_zoom.jpg]

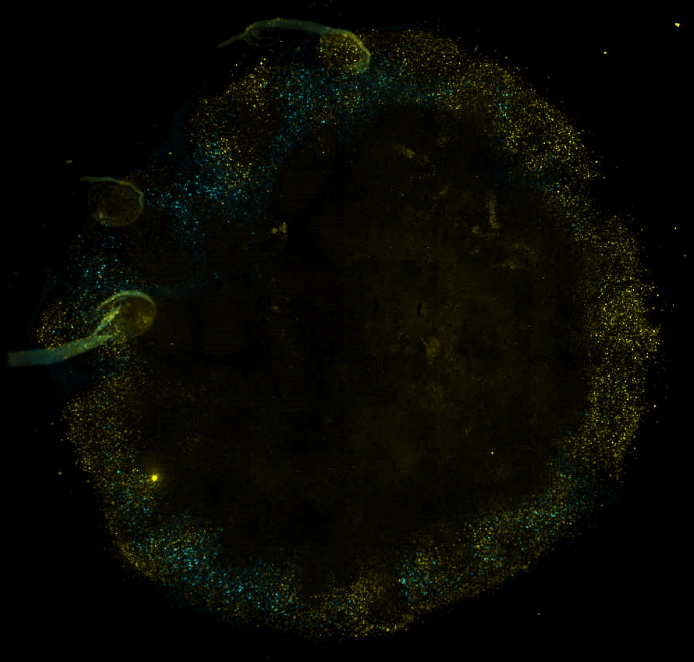

Supplement: Supplementary file 6 — Source Data for Figure 5 [file EMBJ-42-e113213-s007.zip › Figure5/Fig5I/SCGN-COUPTFii/Fig5I_H9_D120_MGliq_cyanSCGN-yellowCOUPTFii.jpg]

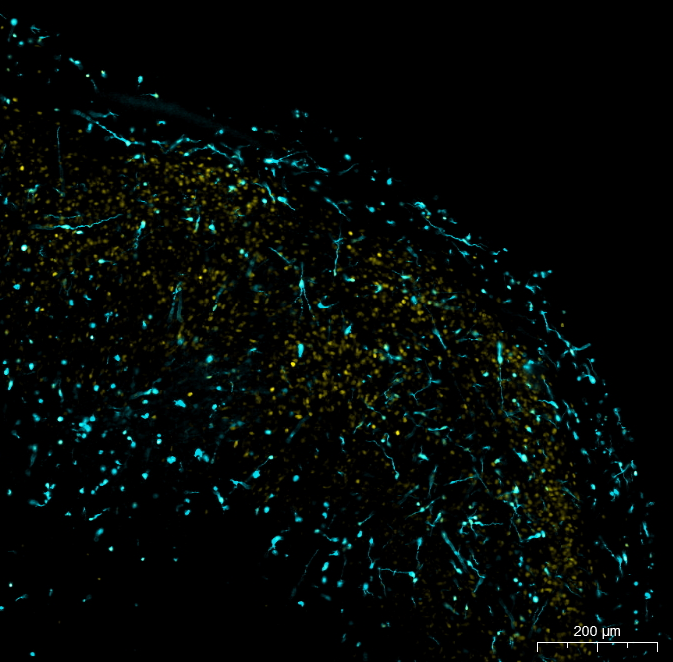

Supplement: Supplementary file 6 — Source Data for Figure 5 [file EMBJ-42-e113213-s007.zip › Figure5/Fig5I/SCGN-COUPTFii/Fig5I_H9_D120_MGnull_cyanSCGN-yellowCOUPTFii_zoom.jpg]

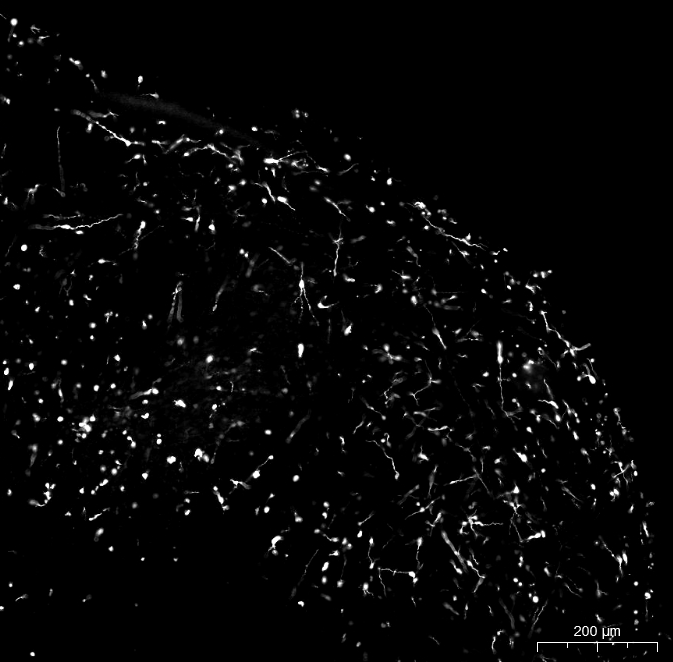

Supplement: Supplementary file 6 — Source Data for Figure 5 [file EMBJ-42-e113213-s007.zip › Figure5/Fig5I/SCGN-COUPTFii/Fig5I_H9_D120_MGnull_SCGN_zoom.jpg]

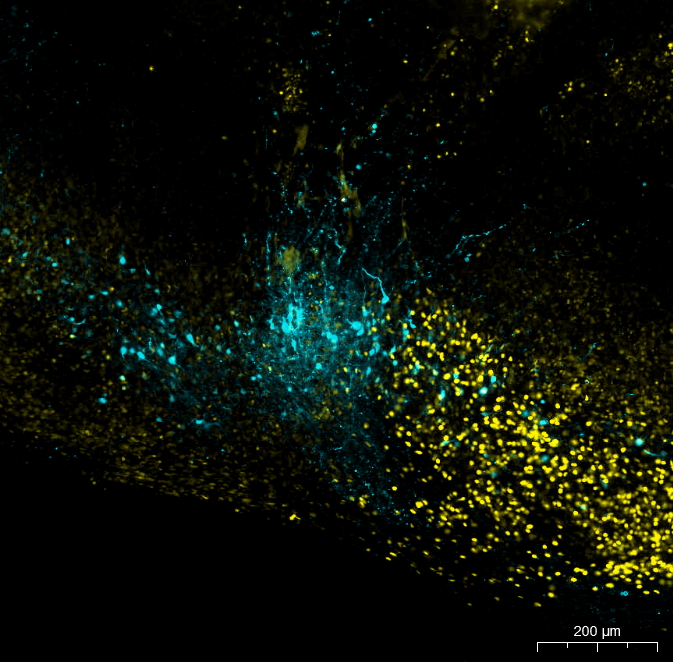

Supplement: Supplementary file 6 — Source Data for Figure 5 [file EMBJ-42-e113213-s007.zip › Figure5/Fig5I/SCGN-COUPTFii/Fig5I_H9_D120_MGdrop_cyanSCGN-yellowCOUPTFii_zoom.jpg]

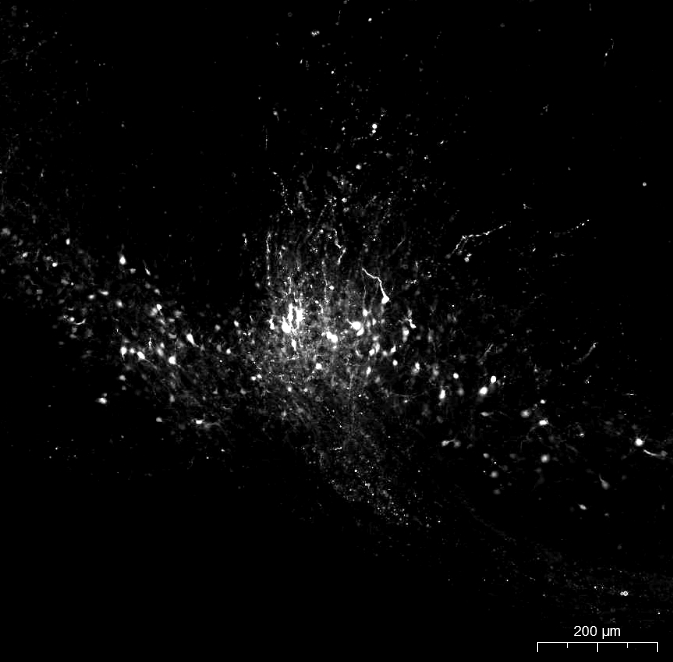

Supplement: Supplementary file 6 — Source Data for Figure 5 [file EMBJ-42-e113213-s007.zip › Figure5/Fig5I/SCGN-COUPTFii/Fig5I_H9_D120_MGdrop_SCGN_zoom.jpg]
