# Supplementary figures and images for: Morphogenesis and development of human telencephalic organoids in the absence and presence of exogenous extracellular matrix (part 2 of 2)
Source: EMBO J. 2023 Oct 16;42(22):e113213. doi: 10.15252/embj.2022113213 (PMC10646563; doi:10.15252/embj.2022113213)

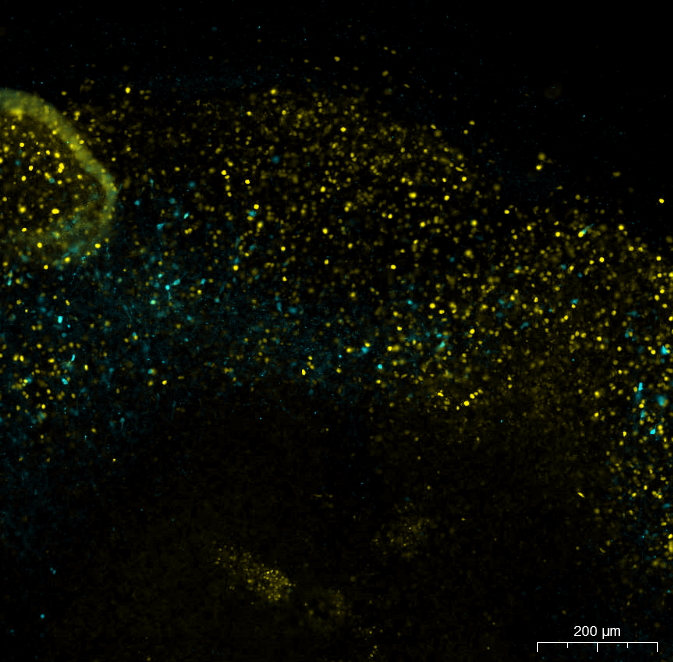

Supplement: Supplementary file 6 — Source Data for Figure 5 [file EMBJ-42-e113213-s007.zip › Figure5/Fig5I/SCGN-COUPTFii/Fig5I_H9_D120_MGliq_cyanSCGN-yellowCOUPTFii_zoom.jpg]
