# Supplementary material for: Morphogenesis and development of human telencephalic organoids in the absence and presence of exogenous extracellular matrix
Source: EMBO J. 2023 Oct 16;42(22):e113213. doi: 10.15252/embj.2022113213 (PMC10646563; doi:10.15252/embj.2022113213)
Supplement: Supplementary file 7 [file EMBJ-42-e113213-s005.pdf]

THE EMBO JOURNAL

Morphogenesis and development of human telencephalic organoids in the absence and presence of exogenous extracellular matrix

Catarina Martins-Costa, Vincent Pham, Jaydeep Sidhaye, Maria Novatchkova, Andrea Wiegers, Angela Peer, Paul Möseneder, Nina Corsini, and Juergen Knoblich
DOI: 10.15252/embj.2022113213

Corresponding authors: Juergen Knoblich (juergen.knoblich@imba.oeaw.ac.at) , Nina Corsini (nina.corsini@imba.oeaw.ac.at)

Review Timeline:

Submission Date:	6th Dec 22
Editorial Decision:	18th Jan 23
Appeal:	24th Jan 23
Editorial Decision:	27th Jan 23
Revision Received:	9th Jun 23
Editorial Decision:	9th Aug 23
Revision Received:	8th Sep 23
Accepted:	14th Sep 23

Editor: Ieva Gailite

Transaction Report:

(Note: With the exception of the correction of typographical or spelling errors that could be a source of ambiguity, letters and reports are not edited. Depending on transfer agreements, referee reports obtained elsewhere may or may not be included in this compilation. Referee reports are anonymous unless the Referee chooses to sign their reports.)

Dear Jürgen,

Thank you for submitting your manuscript for consideration by The EMBO Journal. I sincerely apologise for the protracted review process due to delayed submission of referee reports. We have now received comments from two reviewers, which are included below for your information. Based on these comments, we unfortunately had to conclude that the study is not a sufficiently strong candidate for publication in The EMBO Journal.

As you can see, while the reviewers find the topic of interest, they also raise novelty concerns by pointing out that previous work has already reported on the effect of Matrigel on brain organoid development and alternative protocols of Matrigel-free brain organoid culture have been published. Furthermore, they indicate that the analysis would have to be substantially deepened regarding the contribution of particular Matrigel and endogenous ECM components on organoid and particular cell type growth and differentiation, and the single cell transcriptomics analysis of organoid development. Finally, reviewer #2 finds that the data quantification should be improved. Given these critical opinions from good experts in the research field, and since major experimental revision beyond our usual 3-month timeframe and with uncertain outcome would be needed to address the main referee concerns, I am afraid that we cannot offer to invite a revised manuscript.

Nevertheless, if you find that you can address all or most of the experimental points to extend manuscript depth towards more detailed investigation of cell type differentiation and individual ECM component impact, I would be happy to reconsider the revised manuscript. In this case, it means that I would send it back to the same reviewers, if possible, but would allow them to make new comments on the data, which might then have to be further addressed if the reviewers are more positive in this round of assessment.

Thank you in any case for the opportunity to consider this manuscript. I am sorry that I could not communicate more positive news, but I nevertheless hope that you will find our reviewers' comments helpful for further improvement of the manuscript.

With kind regards,

Ieva

Ieva Gailite, PhD
Senior Scientific Editor
The EMBO Journal
Meyerhofstrasse 1
D-69117 Heidelberg
Tel: +4962218891309
i.gailite@embojournal.org

Referee #1:

In the current manuscript, Catarina Martins-Costa and colleagues examined the effects of exogenous ECM exposure on human telencephalic organoid generation. The authors demonstrated that the early exposure of ECM causes rapid tissue polarization and complete rearrangement of neuroepithelial architecture. In addition, the authors claimed that exogenous and intrinsic ECM offer alternative paths for reaching comparable architecture. This will be very informative to link the ECM and neuroepithelial structure in brain organoids. However, there are several concerns to address before publication.

Major comments:
1. The authors described the effects of exogenous ECM during organoid generation. However, only Matrigel was used in the experiments. So, it means the authors examined the effects of Matrigel during organoid generation in the manuscript. Matrigel has been commonly used in the organoid culture. But there are many disadvantages such as lack of tissue-specific ECM compositions, poorly defined, lot-to-lot variability, and so on (Heo et al., 2022; Kozlowski et al., 2021). Previous studies (Chang et al., 2022; Magni, M. et al., 2021) already described the effect of Matrigel on brain organoids through organoid morphology and RNA sequencing. Although the authors nicely displayed the neuroepithelial architectures upon Matrigel, the effects of Matrigel on brain organoids were already described. For the effects of exogenous ECM, the authors need to test the different types of ECM including brain-specific ECM (Cho et al., 2021). Or, minimally authors should select only laminin and examine its impact on organoid formation. Authors may identify one of the ECM in Matrigel that drive brain organoid into optic-cup lineage.

2. The authors claimed Matrigel affected on early morphogenesis but not the later developmental time. However, in figure 5 the authors performed scRNA seq only at later stage (D120). This only show a limited information regarding the impact of Matrigel

on early basal layer formation. To reveal the cellular states and molecular features at early stage, the authors should perform scRNA seq on early brain organoids, and describe what are the molecular regulation by Matrigel.

3. In figure 1C, the growth rate was significantly increased upon treating Matrigel. ECM might help for expanding of progenitor populations at early stage. This difference needs to be investigated though proliferation rate, the number of ventricular zones, and so on.

Referee #2:

In the manuscript "Morphogenesis and development of human telencephalic organoids in the absence and presence of exogenous ECM" by Martins-Costa et al., the authors assess the effect of exogenous extracellular matrix (ECM) provided in the form of Matrigel on the development of telencephalic organoids. They particularly assess the overall size and morphology, tissue polarity and architecture as well as tissue identity over a time course from 10 to 120 days of development using extensive immunostainings as well as scRNA-seq. They show that, while early in development (until day 20) exogenous ECM influences organoid morphology, polarity and growth, at later time points they report that they find similar tissue polarity and organization and comparable cell types in both conditions.
Matrigel is debated in the field due to its murine source, its undefined composition, and it has been suspected as a source of organoid variability. The paper therefore addresses an important question. The authors present an impressive amount of stainings and I appreciate that most data is shown for 4 different lines including one ESC and 3 iPSC lines. However, a major concern for publication in EMBO journal is that the manuscript lacks quantification to support its major claims and fails to reveal any mechanistic insight. The presented stainings look convincing, however the lack of quantification makes it unclear if only the chosen organoids showed the results or if the results are consistent across organoids and batches. In addition, the result that telencephalic organoids can be generated without matrigel is not new as protocols exist to grow telencephalic organoids without matrigel (e.g. protocols from Sergiu Pasca's lab). Further, I find it misleading to read "morphogenesis and development of human telencephalic organoids" in the title, since the paper does not really explore morphogenetic mechanisms. The study provides a qualitative demonstration that exogenously applied ECM in the form of Matrigel is dispensable for generating human telencephalic organoids since a similar overall tissue morphology and organization can be generated without it. This is an interesting but rather small insight that might not be of interest to the wide audience targeted by EMBO Journal. A more specialized journal might be better suited.

Major concerns:
1) Lack of quantification: Throughout the manuscript, information about the number of batches, organoids, organoid slices analyzed is missing. This is important as there can be variability between organoids and organoid batches. Also, very often a lot of stainings are shown providing a qualitative impression, but a quantification is missing. For example Figures 2 and 3 and S2-S5 show stainings but no quantification. It is unclear whether the effects are consistent across different regions within an organoid, across organoids and across organoid batches.
2) Some of the key results in the manuscript are in my opinion overinterpretations to derive causality. A major result of the manuscript stating that organoids grown in the absence of Matrigel secrete endogenous ECM is an important insight indicating a possible mechanism leading to self-polarisation. This result however is not supported by sufficient experiments. The authors use just one antibody staining against Fibronectin to claim that organoids in the absence of Matrigel secrete ECM, but no other ECM genes secreted in brain development are assessed. The authors show in Figure 3 stainings for mouse LAM1 to visualize laminin provided by matrigel, but it would be interesting to assess whether human LAM1is being secreted by the organoid cells. Overall, the authors use the term "endogenous ECM", but only assess fibronectin. They should therefore be more precise with their wording unless they assess more ECM proteins. Further, in Line 22 the authors write "In unexposed cultures, endogenous ECM production by NPCs results in gradual polarity acquisition over an extended time." The authors do not provide evidence supporting this claim that FN secretion is directly causal to polarity establishment overtime.
3) The analysis of the scRNA-seq data is inconclusive. In Figure 5, the authors present scRNA-seq data for 3 120 day old organoids from each condition and they assess the cell type composition in each organoid. One Matrigel exposed organoid contained a larger fraction of OTX2 and RAX positive cells and organoids not exposed to matrigel overall contained more ventral telencephalic cells, but also beyond these two more obvious points, there seem to be variability in cell type proportions across organoids and protocols. However, this is not quantitatively assessed in terms of enrichment and depletion. The stacked barplot in panel H only provides a qualitative picture. The fact that one out of 3 organoids had a significant proportion of non-telencephalic cells suggests that telencephalic specification did not happen very efficiently. Did the authors assess with stainings against FOXG1, OTX2 and RAX the proportion of organoids with pure telencephalic identity in all three conditions? What does it mean that there are more ventral telencephalic cells in the organoids not exposed to matrigel? Is this an effect of the early differences observed? To me, this suggests that there are in fact differences in cell type composition of organoids generated by the three different protocols, which would be in disagreement with the general message brought across by the authors. More organoids and organoids from different batches might need to be explored in order to get a conclusive picture.

Minor Comments
- The authors use Dorsal telencephalic or telencephalic organoids interchangeably. However, Fig 5 indicates ventral

telencephalic cells are also present in the organoids and it seems that "telencephalic organoids" should therefore be used.
- The use of ECM throughout the manuscript to refer to Matrigel can be misleading. It gives the reader the impression that more than just one matrix is being explored. Consider revising to MATD, MATL, MAT-.
- Line 68, "different modes of ECM". Only Matrigel is analysed in this study and this wording should therefore be changed. No comparison for any other ECM source such as Geltrex or any synthetic matrix.
- Line 78, it's not clear from previous introduction why the authors want to "assess the intrinsic ability of neuroepithelium to secrete ECM"
- Line 92, no rationale is provided for the initial setup of conditions. Why was there a decision to test Matrigel in liquid, why was 2% chosen?
- Line 110, How were the organoids shown chosen? What is the number of organoids that reach the same size? Only 3 organoids are shown over time- do all organoids show the same behaviour? Are there batch differences? Cell line differences?
- Line 127, Does the SOX2 line grow at the same rate as the parental line? Is the mixing consistent to interpret SOX2+ cells between conditions?
- Line 153 Fig 3A,B: PKCζ appears to be expressed not just apically, but also in cells basally located for ECM+ organoids at day 13. This has not been mentioned in the text. How variable is this result? There is a need to better quantify the staining.
- Line 154-155 Is it the exogenous ECM remaining or is it secreted ecm supporting the initial exECM and getting stained?
- Line 156 Fig 3A, FN1 spots are also seen in exECM organoids. This needs quantification of FN1 across the conditions. How many lines/batches were tested?
- Fig S5A Which condition is shown here?
- Fig S2 the different cell lines all show FN1 speckles, please quantify and comment. n number of organoids that show this?
- Fig S5 and line 182-183 the LUM staining is very comparable across conditions. Doesn't this indicate the conditions are polarised and develop very similar even at early stages? By Day 16/20 all conditions catch up and reach same level?
- Line 210-11, Fig 4D, S10D, The OTX2 staining does not look very clear and it is observed in all conditions, whereas OTX2 positive cells are seen in the scRNA-seq data only in the +Matrigel condition. How does this fit together?
- Line 216-218 "Overall, organoids cultured in the absence of exogenous ECM were more homogenous, and continued signaling from exogenous ECM potentiated an increased differentiation or expansion of non-telencephalic". What is the proportion on non-telencephalic tissue? No clear quantification to support the statement from either statining or scRNAseq
- Fig 5 UMAP doesn't show conditions.
- Line 303-306, please comment on cell line dependency, batch variability, organoid numbers for this conclusion.
- 344-346 "Despite these commonalities, biases in tissue patterning that favor the expansion of optic cup tissue in exECM+D organoids are already significantly higher at D40 and persist throughout time." This was not quantified properly.

** As a service to authors, EMBO Press provides authors with the possibility to transfer a manuscript that one journal cannot offer to publish to another EMBO publication or the open access journal Life Science Alliance launched in partnership between EMBO Press, Rockefeller University Press and Cold Spring Harbor Laboratory Press. The full manuscript and if applicable, reviewers' reports, are automatically sent to the receiving journal to allow for fast handling and a prompt decision on your manuscript. For more details of this service, and to transfer your manuscript please click on Link Not Available. **

Please do not share this URL as it will give anyone who clicks it access to your account.

Dear Ieva,

Thank you for considering our manuscript for publication at EMBO Journal and for the editorial assessment after peer review.

While we are pleased that both reviewers find our work insightful, important, and of high quality, they also raise some valid concerns. Both reviewers suggest that **including other ECM preparations** or components besides Matrigel to evaluate effects of exogenous ECM exposure, as well as a more thorough characterization of endogenous ECM production, could greatly improve the insight and novelty of the current findings and make the study suitable for publication in EMBO Journal. We are confident that we will be able to address these concerns as well as the majority of the other concerns raised by the reviewers in a **reasonable timeline (< 3 months)**, as detailed below.

In the revised version of the manuscript, we will:

- **Expand the analysis of endogenously produced ECM components** (**point 2 reviewer#2**)**:**

We based most of our analysis on fibronectin staining, as a proxy for ECM. In the revised manuscript, we will add immunohistochemistry on samples that we have previously generated for other ECM components including laminin and different types of collagens, to improve this characterization.

- Assess the **effect of purified exogenous ECM components on organoid development:**

We will analyse the effect of exogenous addition of purified laminin on early organoid development (until D20), and analyse whether these effects are comparable to those of Matrigel (**point 1 reviewer#1**). By doing so, we will provide further insight into ECM components responsible (or not) for the observed effects of Matrigel in epithelial morphogenesis. Should purified laminin or collagen 4 have a similar effect, we could offer an alternative to Matrigel for experiments that need early establishment of epithelial polarity.

We will also address other concerns brought up by the reviewers as follows:

- **Improve quantifications.**
  - Quantify the endogenous production of ECM at early timepoints (D20), to assess consistency “across different regions within an organoid, across organoids and across organoid batches” (**point 1 reviewer#2**).
  - Quantify dividing progenitors in early (D20) and mid-development (D40) resorting to phospho-histone H3 staining to assess potential differences in cell division induced by Matrigel (**point 3 reviewer#1**).
- **Expand late-stage analysis** (**point 3 reviewer#2**)**.** Reviewer 2 mentions that there are important differences in terms of cell type composition present in the scRNAseq data besides the ones we identified and commented on. As detailed in the manuscript, we found that the scRNAseq data mostly shows a comparable tissue composition, with just slight trends in lineage proportions that cannot be generalized with the number of samples and cells analysed. To address the reviewers comment however, we will expand the scRNAseq analysis with more quantitative metrics that show that the cell types generated are

transcriptionally comparable across ECM conditions. Moreover, we will use D120 samples of fixed tissue from all cell lines and different batches to quantitatively address: telencephalic identity (FOXG1) vs non-telencephalic identity (OTX2); ventral telencephalic identity (DLX2). We believe that basing our findings them on quantitative methods would certainly strengthen the message.

While we believe that the above mentioned revisions could be performed in the suggested 3 months time frame, we consider that performing scRNA sequencing **at early stages** (**point 2 reviewer#1**) would be too expensive and time consuming (in terms of data analysis), while bringing limited benefit to the main message of the paper: that different morphogenic states can be achieved by endogenously or exogenously guided approaches, culminating in comparable tissue morphologies and cell types. This message is, we believe, well supported with current data, and the additional quantifications we mention beforehand.

Finally, **both reviewers raise novelty concerns. On this aspect, we respectfully disagree** – we believe that the current manuscript already offers extremely valuable insights to the brain organoid field. Besides studies from the laboratory of Sergiu Pasca, almost all brain organoid publications use Matrigel. As both reviewers point out, and we have also emphasized in the manuscript, Matrigel is an extremely disadvantageous culture component: expensive, variable, animal-derived. So, we raise the question: why are so many labs using it? For the first time, we make a thorough parallel characterization of the effects of this reagent in brain organoid culture. In our view, publications cited by reviewer 1 take none of the novelty away. Chang et al., 2022 compare two types of Matrigel in droplet embedding of organoids, but make no comparison to Matrigel dissolution in the medium or no-Matrigel conditions. In Magni, M. et al., 2021, the authors use three different organoid protocols previously described in the literature, two of which use Matrigel, and one of which doesn't; however, the protocols differ in many variables other than Matrigel, such as presence of dual SMAD inhibition and activation of different pathways for guided organoid differentiation. Thus, it is simply a comparison of different published protocols, not of the effects of Matrigel. We, on the other hand, use Matrigel as the single medium component variable and explore the effect of this particular component in organoid development. We believe that it is not fair to directly compare insights from those papers to the ones we produced, much less to devalue our contribution based on those publications. We remain confident that our study is of the utmost significance for a wide audience interested in tissue morphogenesis, development, and organoid research, thus benefitting the wide audience targeted by EMBO Journal.

We hope, those facts will make you reconsider your decision and you will allow us to resubmit a revised version of our manuscript within three months. If necessary, we would also be happy to discuss our ideas and concerns over the phone?
Thank you very much for your attention and we will be waiting to hear your thoughts on our proposed plan.
Kind regards,

Jürgen Knoblich
Catarina Costa
Nina Corsini

Appeal - Editorial Decision 27th Jan 2023

Dear Jürgen,

Thank you for contacting me with a preliminary revision plan for your manuscript. I have now gone through it, and I am glad to see that you are prepared to tackle the issues raised by the referees in a major revision. I find your outline reasonable and would like to invite you to submit a revised manuscript in response to the reviewers' comments along the lines indicated in your revision plan. Please note that we will ultimately require strong support from the reviewers for publication here.

Regarding point 2 by reviewer #1, while your point on the limited insight and low cost and time effectiveness of scRNAseq analysis is well taken, I wonder whether you would be able to extend the analysis with differentiation marker stainings at earlier time points. I think it would indeed be useful to discuss the revision in more detail via phone/videoconferencing - please let me know which option you prefer.

We generally allow three months as standard revision time. As a matter of policy, competing manuscripts published during this period will not negatively impact on our assessment of the conceptual advance presented by your study. However, please contact me as soon as possible upon publication of any related work to discuss the appropriate course of action. Should you foresee a problem in meeting this three-month deadline, please let us know in advance to arrange an extension.

When preparing your letter of response to the referees' comments, please bear in mind that this will form part of the Review Process File and will therefore be available online to the community. For more details on our Transparent Editorial Process, please visit our website: https://www.embopress.org/page/journal/14602075/authorguide#transparentprocess. Please also see the attached instructions for further guidelines on preparation of the revised manuscript.

Please feel free to contact me if you have any further questions regarding the revision. Thank you for the opportunity to consider your work for publication. I look forward to receiving the revised manuscript.

With best regards,

Ieva

Ieva Gailite, PhD
Senior Scientific Editor
The EMBO Journal
Meyerhofstrasse 1
D-69117 Heidelberg
Tel: +4962218891309
i.gailite@embojournal.org

Instructions for preparing your revised manuscript:

Please make sure you upload a letter of response to the referees' comments together with the revised manuscript.

Please also check that the title and abstract of the manuscript are brief, yet explicit, even to non-specialists.

When assembling figures, please refer to our figure preparation guideline in order to ensure proper formatting and readability in print as well as on screen:
https://bit.ly/EMBOPressFigurePreparationGuideline
See also guidelines for figure legends: https://www.embopress.org/page/journal/14602075/authorguide#figureformat

At EMBO Press we ask authors to provide source data for the main manuscript figures. Our source data coordinator will contact you to discuss which figure panels we would need source data for and will also provide you with helpful tips on how to upload and organize the files.

IMPORTANT: When you send the revision we will require
- a point-by-point response to the referees' comments, with a detailed description of the changes made (as a word file).
- a word file of the manuscript text.
- individual production quality figure files (one file per figure)
- a complete author checklist, which you can download from our author guidelines (https://www.embopress.org/page/journal/14602075/authorguide).
- Expanded View files (replacing Supplementary Information)

Please see out instructions to authors
https://www.embopress.org/page/journal/14602075/authorguide#expandedview

Please remember: Digital image enhancement is acceptable practice, as long as it accurately represents the original data and conforms to community standards. If a figure has been subjected to significant electronic manipulation, this must be noted in the figure legend or in the 'Materials and Methods' section. The editors reserve the right to request original versions of figures and the original images that were used to assemble the figure.

Further information is available in our Guide For Authors: https://www.embopress.org/page/journal/14602075/authorguide

We realize that it is difficult to revise to a specific deadline. In the interest of protecting the conceptual advance provided by the work, we recommend a revision within 3 months (27th Apr 2023). Please discuss the revision progress ahead of this time with the editor if you require more time to complete the revisions.

Manuscript EMBOJ-2022-113213

Response to the referees' comments

We thank the referees for their insightful comments, which we believe have greatly improved our manuscript. The main changes made were:

- Transcriptional analysis of organoids at early developmental stages.
- Testing the effect of purified ECM components (Laminin and Collagen IV) on organoid development.
- Clarification of the number of organoids and batches used for each analysis.
- Expansion of ECM production analysis to more ECM proteins.
- Quantification of endogenous versus exogenous ECM proteins present in the tissue.
- Expansion of the patterning analysis done at late stages to include immunostaining analysis of 100 organoids of all cell lines.

Please note that the previous scRNAseq data, as well as the new bulk RNAseq data, are accessible through the GSE series accession number **GSE220085**, and can be accessed with the reviewer access token **qtwbwaygtdsjhwn**.

Below, we present a point-by-point response to each of the referees' comments.

<u>Referee #1:</u>

In the current manuscript, Catarina Martins-Costa and colleagues examined the effects of exogenous ECM exposure on human telencephalic organoid generation. The authors demonstrated that the early exposure of ECM causes rapid tissue polarization and complete rearrangement of neuroepithelial architecture. In addition, the authors claimed that exogenous and intrinsic ECM offer alternative paths for reaching comparable architecture. **This will be very informative to link the ECM and neuroepithelial structure in brain organoids.** However, there are several concerns to address before publication.

Major comments:

1. The authors described the effects of exogenous ECM during organoid generation. However, only Matrigel was used in the experiments. So, it means the authors examined the effects of Matrigel during organoid generation in the manuscript. Matrigel has been commonly used in the organoid culture. But there are many disadvantages such as lack of tissue-specific ECM compositions, poorly defined, lot-to-lot variability, and so on (Heo et al., 2022; Kozlowski et al., 2021). Previous studies (Chang et al., 2022; Magni, M. et al., 2021) already described the effect of Matrigel on brain organoids through organoid morphology and RNA sequencing. Although the authors nicely displayed the neuroepithelial architectures upon Matrigel, the effects of Matrigel on brain organoids were already described.

We thank the referee for this insightful comment.

Regarding the **previous publications mentioned**, we would like to point out the following**:**
Chang et al., 2022 studied the effect of different types of Matrigel on organoid development. By finding the upregulation of different signaling pathways depending on the Matrigel type, the authors confirmed that Matrigel introduces unknown patterning cues – one of the main disadvantages of using this reagent in organoid cultures. However, there was **no comparison of different modes of Matrigel application or any condition without Matrigel.** Therefore, we believe that our study is very different from this one.
Magni et al., 2021 compared guided and unguided organoid protocols, which had been previously published. The main goal was to compare tissue patterning induced by these different small molecule cocktails used to pattern the neuroepithelium. Because the protocols were used exactly as published before, two contained Matrigel and one did not. However, **the variable being studied was not Matrigel itself.** Therefore, we believe this study is, too, very different from our own.

Therefore, to our knowledge, **there have not been any publications that compare the effect of Matrigel, as a sole culture variable, on organoid development.** We are confident that this analysis is one of the main points of novelty in our manuscript, and that this new insight can be very useful for the organoid community, aiding scientists in the decision of using, or not, this potentially detrimental culture component.

For the effects of exogenous ECM, the authors need to test the different types of ECM including brain-specific ECM (Cho et al., 2021). Or, minimally authors should select only laminin and examine its impact on organoid formation. Authors may identify one of the ECM in Matrigel that drive brain organoid into optic-cup lineage.

We thank the referee for this comment, we believe this is a very important point. According to the referee's comments, we have now included additional experiments on additional ECM components, which have significantly improved our manuscript.

We exposed organoids to purified ECM components – Laminin and Collagen IV. The choice of these components was deliberate. *In vivo*, Laminin directs the organization of the interstitial ECM, and Collagens are the main structural element of the ECM (Rauti et al., 2019). Both laminins and collagens are necessary during brain development (Amin and Borrell 2020). Furthermore, these are the two main components of Matrigel (Laminin 60% and collagen IV 30%). Therefore, we could not only assess the sole action of ECM proteins with important roles in vivo, but also evaluate whether single main components of Matrigel could replicate its effect and, thereby, serve as replacements.

In the revised manuscript, we have added several experiments that address these questions. We tested organoid liquid embedding with Laminin or Collagen IV, side-by-side with the conditions previously described (Matrigel droplet embedding, Matrigel liquid embedding, and no embedding). We performed brightfield imaging, immunostaining and bulk RNAseq analyses. We believe that this analysis has significantly strengthened the messages of the paper and its novelty, and we thank the referee for the insightful suggestion. The results are as follows:

New figure panels: Figure 2J-K; Figure S6; Figure 3; Figure S7.

New results:

Purified ECM components do not impact neuroepithelial morphogenesis

*Given the undefined composition and batch-to-batch variability of Matrigel, several attempts have been made to replace it with synthetic alternatives or chemically defined ECM proteins, which has been feasible in other organoid systems (**Kozlowski et al.**, 2021). In telencephalic organoids, however, it is currently unclear whether minimal components can replicate the effect of Matrigel. To address this question, we exposed organoids to ECM proteins known to play important roles in brain development (**Amin & Borrell**, 2020) and to be the most abundant ECMs found in Matrigel (**Corning Incorporated Life Sciences**, 2016) – Laminin or Collagen IV, each purified from mouse EHS sarcomas. We used the same protocol as for MG^{+L} conditions, dissolving these proteins in the culture medium (2%V/V), from D13 to D16 (**Fig. 2J**, **Fig. S6A**, $Laminin^{+L}$ and $Coll.IV^{+L}$). To assess organoid morphology, we resorted to brightfield imaging. Interestingly, $Laminin^{+L}$ and $Coll.IV^{+L}$ organoids were comparable to $exECM^{-}$ organoids, presenting a smooth surface with outer brightening, and lacking the budding seen in MG^{+} organoids (**Fig. 2K**, **Fig. S6B**). To assess ECM distribution, we stained these*

*tissues with Ms-LAMA1 and Ms/h-LAMA1 and Ms/h-Perlecan antibodies. A thin Ms-LAMA1 coating was visible in Laminin^{+L} organoids (**Fig. 2K**, **Fig. S6C**), indicating accumulation at the organoid surface. However, ECM proteins of human origin presented a speckled pattern around neural rosettes in Laminin^{+L} and Coll.IV^{+L} organoids, analogous to exECM^{-} conditions (**Fig. 2K**, **Fig. S6C**). Thus, single ECM components were not able to replicate the effects of Matrigel on neuroepithelial budding, following instead a development that closely resembled exECM^{-} cultures.*

Matrigel exposure upregulates transcriptional pathways of eye development

To assess how exogenous ECM signaling affects early organoid patterning and gene expression profiles, we performed bulk RNA sequencing at D20, from single H9 ESCs-derived organoids of all experimental conditions (Fig. 3A and Fig. S7A). (…) The pattern of DE genes confirmed the transcriptional similarity between exECM^{-}, Laminin^{+L}, and Coll.IV^{+L} organoids; and between MG^{+D} and MG^{+L} organoids. (…) Importantly, pure ECM components did not have the same effect, indicating that signaling cues introduced by Matrigel are absent from these purified preparations, which seem to act as inert matrices.

New discussion points:

*Remarkably, when exposed to purified ECM components, Laminin or Collagen IV, organoids presented morphology and transcriptional programs analogous to exECM^{-} conditions. Several aspects may contribute to this result. The mix of ECMs present in Matrigel more closely resembles an in vivo environment, where ECMs are present in combination and not as single components. Also, due to its composition, Matrigel undergoes gelation at temperatures of 22-37 ºC, such that entactin crosslinks Laminin and Collagen IV, creating a hydrogel (**Aisenbrey & Murphy**, 2020). These physical properties, which promote the stickiness and jellification of Matrigel on the organoid tissue, produce an efficient stimulation of basal signaling, whereby several ECM proteins localize simultaneously and at a high concentration at the organoid surface. Additionally, in contrast to Laminin or Collagen IV alone, Matrigel led to the upregulation of Wnt and FGF receptors. This indicates that there may be growth factors in Matrigel that further contribute to its morphogenic action. Overall, our results show that the complex composition and biophysical and/or biochemical properties of Matrigel could not be replaced by pure ECM proteins in the model system here characterized.*

2. The authors claimed Matrigel affected on early morphogenesis but not the later developmental time. However, in figure 5 the authors performed scRNA seq only at later stage (D120). This only show a limited information regarding the impact of Matrigel on early basal layer formation. To reveal the cellular states and molecular features at early stage, the authors should perform scRNA seq on early brain organoids, and describe what are the molecular regulation by Matrigel.

We are grateful that this fair point was raised by the referee, and we have included new experiments to address this concern. Because the organoid tissue is homogenous and has low complexity of cell types at this stage (a large majority is neural progenitors, as shown in Figure 3B), **we performed bulk RNA sequencing at early stages of development (D20).** We were able

to sequence 25 individual organoids from the five conditions of ECM exposure, gaining high resolution in terms of organoid-to-organoid variability at the transcriptional level, as well as sequencing depth. We believe that this experimental setup provided **valuable insights into the cellular states and molecular features on early-stage organoids**.

With this analysis, we believe that our previous conclusions are strengthened and that the level of insight has been increased. By combining a thorough morphological analysis with transcriptional profiling at early stages, we prove that **Matrigel highly impacts organoid architecture and slightly impacts organoid patterning**, promoting eye development pathways (corroborating results at D40 and D120). However, the low number of differentially expressed genes and the homogenous expression of known cell marker genes prove that **organoid identity is largely comparable between conditions**. We further gained insight into the action of Laminin and Collagen IV, which showed a high similarity to exECM^{-} conditions. The exact results are as follows:

New figure panels: Figure 3; Figure S7.

New results:

Matrigel exposure upregulates transcriptional pathways of eye development

*To assess how exogenous ECM signaling affects early organoid patterning and gene expression profiles, we performed bulk RNA sequencing at D20, from single H9 ESCs-derived organoids of all experimental conditions (**Fig. 3A** and **Fig. S7A**). At this stage, organoids were almost exclusively composed of neural progenitor cells (SOX2^{+}) and the first neurons (MAP2^{+}) started to be differentiated (**Fig. 3B**, **Fig. S8**). To assess tissue identity, we verified the expression of common cell-fate marker genes (**Fig. 3C**). Genes marking the telencephalon (FOXG1), neural progenitors (SOX2, NES), cycling NPCs (MKI67, PCNA), dorsal telencephalic progenitors (PAX6) and intermediate progenitors (TBR2), and early-born excitatory neurons (TBR1, CTIP2) were equally expressed across exECM conditions. Markers of the ventral telencephalon (DLX5) were low or absent. Several ECM components known to be expressed during human brain development (LAMA1, FN1, NCN, COL2A1) (**Amin & Borrell**, 2020) showed comparable levels across conditions. To identify putative differences, we performed differential expression (DE) analysis (**Fig. 3D**, **Fig. S7B**). Only 28 genes were found DE between MG^{+D} and exECM^{-} conditions (**Fig. 3D**), and 38 genes considering all pairwise comparisons (**Fig. S7B**). The two clusters of DE genes confirmed the transcriptional similarity between exECM^{-}, Laminin^{+L}, and Coll.IV^{+L} organoids; and between MG^{+D} and MG^{+L} organoids. To assess the cell processes associated with DE genes, we performed gene ontology enrichment analysis. Genes downregulated in MG^{+} conditions did not yield any GO term enrichment. Genes upregulated in MG^{+} conditions were associated with eye development and morphogenesis (**Fig. 3E**) and this signature was stronger in MG^{+D} than in MG^{+L} organoids (**Fig. 3D**, **Fig. S7B**). Notably, Frizzled-5 (FZD5) and fibroblast growth factor binding protein 3 (FGFBP3) were upregulated in MG^{+} organoids (**Fig. 3D**, **Fig. S7B**), suggesting that Wnt and FGF ligands present in Matrigel may influence tissue patterning. Laminin of Collagen IV did not have the same effect, indicating that signaling cues introduced by*

Matrigel are absent from these purified preparations, which likely act as inert matrices. Overall, D20 organoids showed comparable transcription of markers indicative of telencephalic identity, cell-type composition, NPC proliferation, and ECM production, independent of exogenous ECM exposure. Based on the few DE genes, Matrigel activated eye development pathways, particularly when provided as a pure droplet.

3. In figure 1C, the growth rate was significantly increased upon treating Matrigel. ECM might help for expanding of progenitor populations at early stage. This difference needs to be investigated though proliferation rate, the number of ventricular zones, and so on.

We appreciate this comment by the reviewer and we have added new analyses to fully address these points. Specifically, we added an **extensive analysis of the number of ventricular zones during organoid development**, based on immunostaining data of more than 400 organoids of all cell lines at D13-D20, and 190 organoids at D40. Furthermore, we assessed **markers of cellular proliferation in the new bulk RNAseq experiment**.

We concluded that there is **no evidence for higher proliferation rate in Matrigel-exposed organoids**. In fact, transcriptional analysis did not reveal any cell proliferation markers as differentially expressed between conditions, and showed instead comparable expression of MKI67, Nestin, Vimentin, and ID2. However, the data show differences in number and size of ventricular zones, whereby Matrigel induces the formation of many ventricular zones from early on (D20), which are larger but in lower numbers (D40 data). We show that tissue architecture starts with several cavitation spots at D10. By adding a strong signal for polarization, Matrigel likely leads to the merging of these cavitation spots, thereby expanding tissue size by forming rosettes with large fluid-filled lumina. We believe that this analysis answers this important question raised by the referee. The exact results are as follows:

New figure panels: Figure 2B and 2F; Figure S2; Figure 3C-E; Figure S7B

New results:

*Genes marking the telencephalon (FOXG1), neural progenitors (SOX2, NES), **cycling NPCs (MKI67, PCNA),** dorsal telencephalic progenitors (PAX6) and intermediate progenitors (TBR2), and early-born excitatory neurons (TBR1, CTIP2) were equally expressed across exECM conditions.*

*To quantify these observations, we counted the number of PKCζ^{+} neural rosette lumina per cross section, from D13 to D20, in over 400 organoids of all cell lines (**Fig. 2F**, **Fig. S2B-D**). The induction of rosette formation was equally efficient in liquid and droplet embedding modes, as seen by comparable number of neural rosettes in MG^{+D} and MG^{+L} organoids from D13 (**Fig. 2F**, **Fig. S2B-D**). In exECM^{-} organoids, the number of neural rosettes was initially lower (**Fig. S2B-C**) but, by D20, was comparable among all conditions in most cell lines (**Fig. S2D**). Thus, Matrigel exposure caused fast*

changes in tissue polarity and NPC organization, concomitant with the formation of neural rosettes. Interestingly, analogous reorganization happened in the absence of exogenous ECM with a delay of 5-7 days, suggesting that intrinsic self-organization processes must be in place in exECM^{-} organoids.

*To further quantify morphological features, we measured the size of over 250 organoids (**Fig. S13A**) and quantified the area and number of around 8000 rosettes in a total of 190 organoids (**Fig. S13B**). This analysis revealed that MG^{+D} organoids were significantly larger (**Fig. 4E**, **Fig. S13A**) and presented a larger rosette area than exECM^{-} (**Fig. 4F**, **Fig. S13B**), whereas differences between MG^{+L} and exECM^{-} were less or not significant (**Fig. S13A-B**). On the other hand, exECM^{-} organoids presented a higher number of rosettes than MG^{+} organoids (**Fig. 4G**, **Fig. S13C**). Thus, during production of deep-layer excitatory neurons, general features of rosette identity and spatial organization were largely independent of early Matrigel exposure or hPSC genetic background. Matrigel embedding caused an expansion of tissue and rosette size, balanced by a lower number of rosettes in comparison to exECM^{-} organoids.*

Referee #2:

In the manuscript "Morphogenesis and development of human telencephalic organoids in the absence and presence of exogenous ECM" by Martins-Costa et al., the authors assess the effect of exogenous extracellular matrix (ECM) provided in the form of Matrigel on the development of telencephalic organoids. They particularly assess the overall size and morphology, tissue polarity and architecture as well as tissue identity over a time course from 10 to 120 days of development using extensive immunostainings as well as scRNA-seq. They show that, while early in development (until day 20) exogenous ECM influences organoid morphology, polarity and growth, at later time points they report that they find similar tissue polarity and organization and comparable cell types in both conditions. Matrigel is debated in the field due to its murine source, its undefined composition, and it has been suspected as a source of organoid variability. The paper therefore addresses an important question. The authors present an impressive amount of stainings and I appreciate that most data is shown for 4 different lines including one ESC and 3 iPSC lines.

However, a major concern for publication in EMBO journal is that the manuscript lacks quantification to support its major claims and fails to reveal any mechanistic insight. The presented stainings look convincing, however the lack of quantification makes it unclear if only the chosen organoids showed the results or if the results are consistent across organoids and batches. In addition, the result that telencephalic organoids can be generated without matrigel is not new as protocols exist to grow telencephalic organoids without matrigel (e.g. protocols from Sergiu Pasca's lab). Further, I find it misleading to read "morphogenesis and development of human telencephalic organoids" in the title, since the paper does not really explore morphogenetic mechanisms. The study provides a qualitative demonstration that exogenously applied ECM in the form of Matrigel is dispensable for generating human telencephalic organoids since a similar overall tissue morphology and organization can be generated without it. This is an interesting but rather small insight that might not be of interest to the wide audience targeted by EMBO Journal. A more specialized journal might be better suited.

We thank the referee for their global assessment of our manuscript.
While other labs have used organoid protocols that lack Matrigel addition (as mentioned and cited in the Introduction of our manuscript), we strongly believe that a **systematic comparison of the effects of Matrigel on organoid development is lacking in the literature.** As an emerging tool to model neurodevelopment, and given the strong problems associated with Matrigel usage, we do believe that this characterization is of the utmost importance to the field. We have, for the first time, thoroughly characterized the effects of Matrigel longitudinally over 120 days of organoid development. These data are strongly reproduced in 4 cell lines and provide novel knowledge about the **need and impact of this culture component** that were not previously known. We believe that this constitutes an important contribution to neural organoid research.

We are also of the opinion that **a large fraction of our work assesses organoid morphogenesis:** We address cavitation, rosette formation, polarity, cellular organization and spatial distribution, as well as global features of tissue architecture. In the current version of the manuscript,

morphological assessments/measurements are made in the following figure panels: Fig. 1B-C, Fig. S2A, Fig. 2, Fig. S3, Fig. S5, Fig. S6, Fig. 4., Fig. S10, Fig. S11, Fig. S13, Fig. 5, Fig. S15, Fig. S16. In fact, to our knowledge, this is one of the most extensive works in **organoid staining and measurement of morphological characteristics, for which 4 cell lines and thousands of organoids across development have been used.** Some of these analyses have been expanded or clarified as per the referee's suggestions (please see below) and helped strengthen this part of the message. Therefore, we argue that the title of the manuscript is justified.

Major concerns:

1) Lack of quantification: Throughout the manuscript, information about the number of batches, organoids, organoid slices analyzed is missing. This is important as there can be variability between organoids and organoid batches. Also, very often a lot of stainings are shown providing a qualitative impression, but a quantification is missing. For example Figures 2 and 3 and S2-S5 show stainings but no quantification. It is unclear whether the effects are consistent across different regions within an organoid, across organoids and across organoid batches.

We thank the referee for this fair and important comment. In fact, we had failed to provide this valuable information in the first version of the manuscript. In the revised version, **the information on organoid numbers and batches is provided, as well as relevant statistical analyses,** as follows (please see results and figures for full information):

Data	Figure	Cell lines	Total N numbers
Organoid size D10-20	S1	4	>1000 organoids
Rosette number D10-20	S2	4	>400 organoids
Endogenous vs exogenous ECM	S4	4	88 organoids, 276 rosettes
Organoid size D40	4E + S13A	4	254 organoids
Rosette area number	4F-G, S13B-C	4	190 organoids, >7900 rosettes
$OTX2^{+}$ area D40	4H + S13D	4	190 organoids
Telencephalic patterning D120	5J, S16F	4	98 organoids
Dorsal/ventral patterning D120	5K, S16F	4	98 organoids

We believe that all experiments have been sufficiently reproduced across cell lines and batches and that this information clarifies the reproducibility and strengthens the conclusions of the work.

2) Some of the key results in the manuscript are in my opinion overinterpretations to derive causality. A major result of the manuscript stating that organoids grown in the absence of Matrigel secrete endogenous ECM is an important insight indicating a possible mechanism leading to self-polarisation. This result however is not supported by sufficient experiments. The authors use just one antibody staining against Fibronectin to claim that organoids in the absence of Matrigel secrete ECM, but no other ECM genes secreted in brain development are assessed. The authors show in Figure 3 stainings for mouse LAM1 to visualize laminin provided by matrigel, but it would be interesting to assess whether human LAM1 is being secreted by the organoid cells. Overall, the authors use the term "endogenous ECM", but only assess fibronectin. They should therefore be more precise with their wording unless they assess more ECM proteins. Further, in Line 22 the authors write "In unexposed cultures,

endogenous ECM production by NPCs results in gradual polarity acquisition over an extended time." The authors do not provide evidence supporting this claim that FN secretion is directly causal to polarity establishment overtime.

We thank the referee for their suggestion, based on which we have made important changes to the manuscript. We believe that these insightful comments have greatly improved the study.

To tackle the question of production of other ECM components, we have **expanded our immunostaining analysis** to show production of

- **Fibronectin**
- **Laminin**, as per referee's request. Laminin is essential for the expansion, maintenance and differentiation of mouse and human NPCs (Amin and Borrell 2020).
- **Perlecan**, an ECM component of the basement membrane which has important functions in support and proliferation of neuroepithelial cells in vivo. Mutations in Perlecan cause severe neurodevelopmental effects in the mouse (Amin and Borrell 2020)
  Importantly, the patterns of expression of FN, Laminin, and Perlecan were overlapping in organoids, occupying outer rosette regions (basal ECM, Figure 2H).
- **Lumican** (already in the first manuscript, apical accumulation seen in all organoids)

When stained together with the antibody for mouse-derived Laminin (useful to identify Matrigel), these antibodies were useful to **distinguish and confirm the endogenous production of several ECMs.** We expanded this analysis at D20, also quantifying endogenous and exogenous ECM components (detailed in another point below) and shown sustained ECM production at D40.

We have also confirmed the **comparable expression of ECM proteins** across conditions with **bulk RNAseq at D20**, showing exemplary markers: LAMA1 and FN1, to corroborate the immunostaining analysis; NCAN and COL2A1, with known inportance during human brain development (Amin and Borrell, 2020).

We believe that these analyses sufficiently expands on our previous data and **supports our global claims of endogenous ECM production in organoids.**

The exact results are as follows:
New figure panels: Fig. 2G-I, Fig. S4, Fig. S9A-B, Fig. 3C

New results:

*To discriminate between ECM produced endogenously and ECM contributed by Matrigel, we used an antibody that recognizes mouse, but not human, laminin-α1 (Ms-LAMA1); together, we used antibodies that recognize both mouse and human FN, LAMA1 and Perlecan (**Fig. 2H**). In exECM-*

*organoids, Ms-LAMA1 was absent, as expected; Ms/h-FN, LAMA1, and Perlecan showed overlapping expression, with a speckled pattern around neural rosettes that did not reach the outer-most surface of the organoids – indicative of endogenously-produced ECM. In MG⁺ organoids, the smooth FN⁺LAMA1⁺Perlecan⁺ surface was co-positive for Ms-LAMA1, identifying Matrigel-derived ECM; in addition, Ms/h-ECM-positive but Ms-LAMA1-negative speckles were seen within the tissue. (…) To further address the production of ECM proteins absent from Matrigel, we assessed the presence and tissue distribution of Lumican (LUM), which is produced by human NPCs and plays an important role in cortical development in vivo (**Long et al.**, 2018). LUM was abundant in organoids from early stages of development, and its tissue distribution followed a pattern similar to that of PKCζ. We observed scattered and disordered distribution of LUM at D10 (**Fig. S5A**) and accumulation of LUM in rosette lumina from D13 in MG⁺ organoids (**Fig. S5B**), and at D20 in all conditions (**Fig. S5C**). Thus, Matrigel addition led to the formation of a sheet of ECM at the outer-most organoid surface, distinguishable from, but not replacing, endogenously-produced ECM within the tissue; in its absence, several ECM components were produced endogenously and underwent self-organization in all organoids analysed, corroborating the robustness of this process.*

*To assess if the initial differences in neuroepithelial morphogenesis affected early neurogenic stages, we evaluated tissue architecture at D40. At this stage, prominent neural rosettes were visible in all conditions with brightfield imaging (**Fig. 1B**, **Fig. S1**, tissue architecture schematized in **Fig. 4A**). To assess the presence and distribution of endogenous and exogenous ECM, we resorted to immunostaining with Ms-LAMA1 and Ms/h-LAMA1. Ms-LAMA1 staining showed that most MG^{+D} organoids remained encapsulated in a Matrigel droplet, while remnants of Matrigel were still visible within MG^{+L} organoids (**Fig. S9A**). The production of endogenous ECM was also sustained, as seen by abundant Ms/h-LAMA1 expression (**Fig. S9B**). Whereas all rosettes in $exECM^{-}$ organoids were surrounded by endogenously produced LAMA1, some rosettes in MG⁺ organoids were still encapsulated by Matrigel-derived ECM (**Fig. S9B**). Thus, there was continued presence of Matrigel in the tissue, even 30 days after exposure.*

*Several ECM components known to be expressed during human brain development (LAMA1, FN1, NCAN, COL2A1) (**Amin & Borrell**, 2020) showed comparable levels across conditions.*

New discussion points:

*Furthermore, $exECM^{-}$ organoids produce ECM proteins – such as Fibronectin, Laminin, Perlecan, Lumican, Neurocan, and Collagens – that self-assemble along an apical-basal polarity axis at pre-neurogenic stages. This shows that organoid NPCs produce ECM proteins with relevance during in vivo neurodevelopment (**Amin & Borrell**, 2020; **Camp et al.**, 2015). In the human brain, NPCs have been postulated to contribute to basal deposition of ECM constituents via vesicular transport in their basal processes (**Fietz et al.**, 2012), thereby contributing to tissue polarization. We propose that an analogous self-sustained process may be taking place during in vitro development, contributing to*

the establishment and maintenance of apical-basal polarity in the absence of exogenous instructive signals.

Regarding the **claims on causality**, we have rephrased our conclusions to convey the correlation/simultaneity of processes (ECM production – ECM organization – polarity establishment) rather than causality, in the following manner:

> *Thus, Matrigel exposure caused fast changes in tissue polarity and NPC organization,* ***concomitant with*** *the formation of neural rosettes.*
>
> *In summary, exogenous ECM addition established a clear basal-out/apical-in polarity axis in MG^{+} organoids from D13; and exECM^{-} organoids* ***endogenously produced fibronectin that self-organized*** *around neural rosettes (schematized in Fig. S3C).*
>
> *Thus, Matrigel addition led to the formation of a sheet of ECM at the outer-most organoid surface, distinguishable from, but not replacing, endogenously-produced ECM within the tissue; in its absence, several ECM components were produced endogenously and* ***underwent self-organization*** *in all organoids analysed, corroborating the robustness of this process.*

3) **The analysis of the scRNA-seq data is inconclusive.** In Figure 5, the authors present scRNA-seq data for 3 120 day old organoids from each condition and they assess the cell type composition in each organoid. One Matrigel exposed organoid contained a larger fraction of OTX2 and RAX positive cells and organoids not exposed to matrigel overall contained more ventral telencephalic cells, but also beyond these two more obvious points, there seem to be variability in cell type proportions across organoids and protocols. However, this is not quantitatively assessed in terms of enrichment and depletion. The stacked barplot in panel H only provides a qualitative picture. The fact that one out of 3 organoids had a significant proportion of non-telencephalic cells suggests that telencephalic specification did not happen very efficiently. Did the authors assess with stainings against FOXG1, OTX2 and RAX the proportion of organoids with pure telencephalic identity in all three conditions? What does it mean that there are more ventral telencephalic cells in the organoids not exposed to matrigel? Is this an effect of the early differences observed? To me, this suggests that there are in fact differences in cell type composition of organoids generated by the three different protocols, which would be in disagreement with the general message brought across by the authors. More organoids and organoids from different batches might need to be explored in order to get a conclusive picture.

We are grateful for the referee's comment and suggestions on how to strengthen our analysis at late developmental stages. We have added important new data to tackle these questions.

We believe that the single-cell RNA sequencing analysis presented offers important insights into long-term organoid development. In particular, it shows that the global cell-type composition and cell-fate acquisitions at late stages of organoid development are not significantly affected by early Matrigel exposure. Being currently one of the golden standard analyses in the field, we are confident that this scRNAseq data can be very valuable, setting a benchmark for the organoid community interested in performing long-term experiments with the liquid-embedding or Matrigel-free protocols (not previously evaluated with this technique). However, scRNAseq allows only limited numbers of single-organoid multiplexing (here, we have only 3 organoids per condition), and different cell types may be differently susceptible to dissociation and FACS sorting, and therefore recovered in proportions that are not completely representative of the initial tissue composition. These are some of the reasons why we refrained from overinterpreting our results, potentially contributing to the referee's assessment that the data were inconclusive.

As suggested by the referee **we analyzed "More organoids and organoids from different batches ...in order to get a conclusive picture".** We performed an extensive **immunostaining analysis at D120, a total of 100 organoids** from all cell lines. As suggested, we assessed **telencephalic vs non-telencephalic patterning** using FOXG1 and OTX2 co-staining; as well as **dorsal/ventral patterning** using SATB2 and DLX2 co-staining.

We confirm that **non-telencephalic patterning occurs more extensively Matrigel droplet embedded organoids**, corroborating several other experiments at different timepoints (bulk RNAseq at D20, OTX2+ area at D40, and scRNAseq at D120). Furthermore, we show that **dorsal/ventral patterning is not a function of Matrigel exposure**, mainly depending on the cell line – certain cell lines are more prone to generate more ventral tissue than others, likely due to intrinsic pre-patterning. Overall, we believe that this analysis significantly improves our knowledge of the long-term impact of Matrigel on organoid development and patterning, constituting a major improvement to the manuscript. The exact results are as follows:

New figure panels: Fig.5 J-K, Fig. S16

New results:
*To validate the cell-type composition in all cell lines and conditions, we resorted to immunostaining at D120 (**Fig. 5I**, **Fig. S15**). Organoids were composed mostly of deep- and upper-layer ExNs (CTIP2$^+$ and SATB2$^+$, respectively) with rudimentary layer organization. Interneurons (SCGN$^+$ and COUPTFII$^+$, indicating caudal ganglionic eminence origin) were found intermingled with ExNs in all conditions and cell lines (**Fig. 5I**, **Fig. S15B**). To quantify potential patterning differences, we measured the ratio between telencephalic/non-telencephalic and dorsal/ventral tissue in around 100 organoids of all cell lines (**Fig. 5J-K**, **Fig. S16**). The proportion of area positive for FOXG1 (telencephalon) and OTX2 (non-telencephalon) (**Fig. S16A-B**) showed that most of the tissue was FOXG1$^+$ for all cell lines and batches (**Fig. 5J**, **Fig. S16E**). However, MG^{+D} conditions contributed to an expansion of OTX2$^+$ non-telencephalic regions, most prominently in H9- and iPSCs#2-derived organoids (**Fig. 5J**, **Fig. S16E**).*

*The increased mis-patterning was driven by exposure to Matrigel and not cell line dependent (**Fig. 5J**). Because interneurons are often seen intermingled with excitatory neurons, it is not always possible to delineate dorsal and ventral areas within an organoid. Therefore, the nuclear markers SATB2 and DLX2 were used as proxies for dorsal and ventral cells (**Fig. S16C**), which could be segmented and counted (**Fig. S16D**). Interestingly, this analysis revealed that the dorsal/ventral patterning was mostly independent from Matrigel exposure (**Fig. 5K**, **Fig. S16E**). Instead, there was a cell line dependency, whereby some cell lines showed an intrinsic tendency to produce more interneurons, such as seen for iPSCs#1 (**Fig. 5K**, **Fig. S16E**). These findings corroborated the patterning analyses done at D20 and D40 (**Fig. 4B**, **Fig. S10**) and the indications from the scRNAseq data at D120. Thus, aside the expansion of optic cup tissue promoted by Matrigel, global telencephalic patterning and cell-type composition was highly similar across experimental conditions at D120. Overall, exposure to Matrigel at the neuroepithelial stages has few long-lasting effects in organoid development.*

Minor Comments

We thank the referee for all the "minor" suggestions made, which we believe have significantly improved the clarity of our results, as well as added important new analyses and quantifications.

- The authors use Dorsal telencephalic or telencephalic organoids interchangeably. However, Fig 5 indicates ventral telencephalic cells are also present in the organoids and it seems that "telencephalic organoids" should therefore be used.

  We thank the referee for pointing out this issue. We have rephrased as "telencephalic" or, in some cases, "dorsal-tissue enriched".

- The use of ECM throughout the manuscript to refer to Matrigel can be misleading. It gives the reader the impression that more than just one matrix is being explored. Consider revising to MATD, MATL, MAT-.

  We thank the referee for raising this question. To tackle this comment and also accommodate the new experiments with Laminn and Collagen IV (asked by Referee#1) we have rephrased the conditions as: MG^{+D}, MG^{+L}, $Laminin^{+L}$, $Coll.IV^{+L}$, ad $exECM^{-}$

- Line 68, "different modes of ECM". Only Matrigel is analysed in this study and this wording should therefore be changed. No comparison for any other ECM source such as Geltrex or any synthetic matrix.

  We thank the referee for flagging this point that could be misunderstood. By "different modes" we meant droplet vs liquid embedding. Because we realize this may not have been clear, we have either eliminated these instances or rephrased as "liquid and droplet embedding modes". In addition to Matrigel, we have now also analyzed the effects of Laminin and Collagen IV in liquid embedding mode.

- Line 78, it's not clear from previous introduction why the authors want to "assess the intrinsic ability of neuroepithelium to secrete ECM"

  Because the introduction has been slightly rephrased, this part has been removed.

  Line 92, no rationale is provided for the initial setup of conditions. Why was there a decision to test Matrigel in liquid, why was 2% chosen?

  We thank the referee for their comment. Inspired by protocols used for other organoid systems (Eiraku et al., 2011; Hocevar et al., 2021; Sanaki-Matsumiya et al., 2022; Veenvliet et al., 2020), we have previously tested different concentrations of Matrigel dissolution in the culture medium in the lab. We finally chose 2% due to it being a low concentration (beneficial for saving resources) that produced standard organoid morphology. Because this is a technically easier way of Matrigel exposure, we believe that it may provide significant technical benefits to droplet embedding. Therefore, we decided to also test this condition in our work.

- Line 110, How were the organoids shown chosen? What is the number of organoids that reach the same size? Only 3 organoids are shown over time- do all organoids show the same behaviour? Are there batch differences? Cell line differences?

  We thank the referee for raising these questions.

  Regarding organoid choice, **the only choice made happened at D10**, as we have written (already in the previous version) in the Materials and Methods: *On Day 10, batches in which over 80% of EBs formed successfully were kept for further experiments. Quality criteria included EB size above 500 μm, round morphology, and the appearance of peripheral tissue clearing, indicative of the start of neuroepithelium formation. Batches compliant with these criteria were randomly divided at D10 into three groups of different conditions of exogenous ECM (exECM) supplementation.*

  From the batches chosen to proceed, organoids were imaged randomly and representative images with stereotypical morphological features are shown in the figures. The reproducibility of the results and organoid growth dynamics can be assessed in Figure 1C (we added the N numbers in Figure S1B), which includes results from over 1000 organoids of all cell lines and 2 or more batches per cell line. We also added a sentence in the results: *These growth dynamics were reproducible across different batches of the same cell line within each experimental condition, showing slight variation across all four cell lines.*

- Line 127, Does the SOX2 line grow at the same rate as the parental line? Is the mixing consistent to interpret SOX2+ cells between conditions?

  We thank the referee for asking this question. In preparation for this experiment **we tried 1% and 20%.** We concluded that 1**% GFP+ cells was too little** to get an idea of the overall distribution of cells. On the other hand, **20% allowed sparse visualization** of cells. As mentioned in the results, the one goal of the sparse labelling was to see the position of individual progenitors: *We analyzed organoids containing 80% H9 wild-type (WT) ESCs and 20% H9 SOX2::EGFP ESCs, as this mixing ratio was sparse enough to allow the recognition of individual SOX2::EGFP NPCs while also revealing their overall tissue distribution (**Fig. 2A** and **C-D**).* Also, **the mixing is consistent between conditions**

because the mixing was **done at the stem cell stage,** when EBs were set up. So, each batch of EBs was generated from **exactly the same pool of cells**, and only later split into the three conditions. This experimental setup is described in the Materials and Methods. We did not assess growth rates but, as mentioned before, the only goal was to achieve sparse labelling, so this factor does not change the interpretation of the results, which are merely qualitatively showing progenitor arrangement.

- Line 153 Fig 3A,B: PKCζ appears to be expressed not just apically, but also in cells basally located for ECM+ organoids at day 13. This has not been mentioned in the text. How variable is this result? There is a need to better quantify the staining.

We thank the referee for bringing up this point. The main message from this panel is that Matrigel accumulates at the organoid surface from D13 (FN^+ staining) and opposes a PKC^+ lumen. We agree that complete PKC polarization becomes more clear at D16. To clarify this point, we have added a few sentences to the results:

*To better understand the timeline of NPC polarization, we assessed the location of PKCζ and Fibronectin (FN), markers of apical and basal domains, respectively (**Fig. 2G**, **Fig. S3**). (…) Embedding in a droplet of Matrigel led to the formation of a permanent basal domain on the outer organoid surface, as seen by the surrounding mesh of FN from D13 to D20 (**Fig. 2G**, **Fig. S3B**; MG^{+D}). Matrigel dissolution in the culture medium led to the formation of a thin ECM coating at the organoid surface that remained visible even one week after exposure (**Fig. 2G**, **Fig. S3B**, MG^{+L}). <u>A complete polarization of $PKC\zeta^+$/FN^+ surfaces was achieved between D13 and D16</u> (**Fig. S3B**).*

- Line 154-155 Is it the exogenous ECM remaining or is it secreted ecm supporting the initial exECM and getting stained?

We thank the referee for their question. By using the **mouse-LAMA1 antibody, we show that exogenous ECM is being stained** (please see Fig. 2H-I and Fig. S4). Furthermore, if there would be a replacement of exogenous ECM by endogenous, we would observe a reduction of the Ms-LAMA1 signal over time, which is not the case. In fact, we observe **permanence of Matrigel** encapsulating or within the organoid tissue over extended periods of time, even at D40 and 120 (please see Fig. S9). However, **we do not exclude that endogenous ECM is recruited to the organoid surface as well**. This point is discussed as follows:

In telencephalic organoids, the action of Matrigel is likely two-fold: 1) introduction of a strong basement membrane signal at the organoid surface, seen to persist for many days after initial exposure, in both MG^{+D} and MG^{+L} conditions; and 2) <u>signal amplification by recruitment and polymerization of endogenously produced ECM.</u> In fact, endogenous ECM within the organoid tissue is widespread in $exECM^-$ organoids and sparser in MG^+ organoids; <u>these differences may be due to recruitment and assembly of endogenously produced FN at the organoid surface in the presence of</u>

*Matrigel. Laminin may play a role in these processes, as it constitutes around 60% of Matrigel (**Corning Incorporated Life Sciences**, 2016), and has been shown to form the initial cell-anchored polymer needed for subsequent ECM assembly, and to nucleate the polymerization of other ECM proteins (**Cheng et al.**, 1997; **S. Li et al.**, 2002, 2003). Thus, slow assembly of endogenous ECM is overtaken by a mass-action of exogenous ECM upon Matrigel exposure, leading to a quick polarization process that likely occurs through a different molecular mechanism than that seen in exECM$^-$ organoids.*

- Line 156 Fig 3A, FN1 spots are also seen in exECM organoids. This needs quantification of FN1 across the conditions. How many lines/batches were tested?
- Fig S2 the different cell lines all show FN1 speckles, please quantify and comment. n number of organoids that show this?

We thank the referee for these insightful comments, which have prompted us to **expand our analysis of endogenous and exogenous ECM components present in the organoid tissue.** We have leveraged the co-staining of Ms-LAMA1 and Ms/h-FN to distinguish and quantify the percentage of area surrounding rosettes that is covered by endogenous or exogenous ECM. We have performed this analysis in 88 organoids from all cell lines, in a total of 276 rosettes. We believe these new results corroborate our previous claims, strengthen the message of the manuscript, and answer the question of how robust the process of ECM production is. The exact results are as follows:

New figure panels: Fig. 2I; Fig S4s

New results:

*The patterns of FN$^+$ regions at D20 were very different in exECM$^-$ and MG$^+$ conditions (**Fig. 2G**). To discriminate between ECM produced endogenously and ECM contributed by Matrigel, we used an antibody that recognizes mouse, but not human, laminin-α1 (Ms-LAMA1); together, we used antibodies that recognize both mouse and human (Ms/h) FN, LAMA1, and Perlecan (**Fig. 2H**), known components of the brain ECM in vivo (**Amin & Borrell**, 2020). In exECM$^-$ organoids, Ms-LAMA1 was absent, as expected; Ms/h-FN, LAMA1, and Perlecan showed overlapping expression, with a speckled pattern around neural rosettes that did not reach the outer-most surface of the organoids – indicative of endogenously produced ECM. In MG$^+$ organoids, the smooth FN$^+$LAMA1$^+$Perlecan$^+$ surface was co-positive for Ms-LAMA1, identifying Matrigel-derived ECM; in addition, Ms/h-ECM-positive but Ms-LAMA1-negative speckles were seen within the tissue. To quantify these observations, we analysed 276 rosettes of 88 D20 organoids from all cell lines. We segmented and measured the percentage of the area surrounding rosettes that was covered by endogenous or exogenous ECM (**Fig. 2I**, **Fig. S4**). We show that there was a large proportion of MG-derived ECM in both MG$^+$ conditions, especially in MG^{+D} organoids, which were covered by a thicker Matrigel layer. In exECM$^-$ conditions, the percentage of area covered by ECM was comparable to MG^{+L} organoids, but its origin entirely endogenous. To further address the production of ECM proteins absent from Matrigel, we assessed the presence and tissue distribution of Lumican (LUM), which is produced by human NPCs and plays an important role*

*in cortical development in vivo (**Long et al.**, 2018). LUM was abundant in organoids from early stages of development, and its tissue distribution followed a pattern like that of PKCζ: scattered and disordered at D10 (**Fig. S5A**) and accumulated in rosette lumina from D13 in MG⁺ organoids (**Fig. S5B**) and at D20 in all conditions (**Fig. S5C**). Thus, Matrigel addition led to the formation of a sheet of ECM at the outer-most organoid surface, distinguishable from, but not replacing, endogenously produced ECM within the tissue; in its absence, several ECM components were produced endogenously and underwent self-organization in all organoids analysed, corroborating the robustness of this process.*

- Fig S5A Which condition is shown here?

  We thank the referee for their question. **At day 10, organoids have not yet been distributed in the different conditions**, as it is the common pool of EBs. The details of this procedure can be found in the Materials and Methods.

- Fig S5 and line 182-183 the LUM staining is very comparable across conditions. Doesn't this indicate the conditions are polarised and develop very similar even at early stages? By Day 16/20 all conditions catch up and reach same level?

  We agree with this observation and make that point in the text: *Thus, Matrigel addition led to the formation of a sheet of ECM at the outer-most organoid surface, distinguishable from, but not replacing, endogenously-produced ECM within the tissue; in its absence, several ECM components were produced endogenously and underwent self-organization in all organoids analysed, corroborating the robustness of this process.*

- Line 210-11, Fig 4D, S10D, The OTX2 staining does not look very clear and it is observed in all conditions, whereas OTX2 positive cells are seen in the scRNA-seq data only in the +Matrigel condition. How does this fit together?

  We thank the referee for this observation. We agree that in the first version of the manuscript the staining was not perfectly clear, due to the imaging method (widefield imaging with slide scanner). To improve the clarity of this results **we have now replaced those figure panels with confocal images**, which, we believe, are much sharper in quality (Fig.4D, Fig.S12). We also believe that **the extent of non-telencephalic cells** is now much more well supported by different experiments:

  - **Bulk RNAseq at D20**, showing already at this stage an eye development signature that is strongest in Matrigel droplet embedding and less prominent in liquid embedding conditions
  - **Quantifications at D40**, showing that the extent of $OTX2^+$ area is much higher in droplet embedding than in the other conditions, with mild levels in liquid embedded organoids.
  - **Quantification of organoid patterning at D120**, corroborating the observations at D40.

    Overall, all these analysis converge to the same conclusion that Matrigel promotes the differentiation of optic cup populations from very early stages of development and that this effect is strongest in Matrigel droplet embedding, less prominent in liquid embedding conditions, and much lower in non-embedded conditions. This is not an effect seen in every single droplet-embedded organoid, but it is more likely to happen in this condition.

This point is discussed as follows:

*To assess tissue patterning and cell type composition, we extensively characterized organoids from D20 to D120 of development. We observed that biases in tissue patterning that increase the likelihood of optic cup tissue expansion in MG^{+D} organoids are already significantly higher at D20 and persist throughout time. These findings are in agreement with pioneering studies on in vitro differentiation of the optic cup, where the retinal epithelium was shown to expand in the presence of Matrigel, but not in its absence (**Eiraku et al.**, 2011). Importantly, although MG^{+L} conditions efficiently promoted rosette formation, unwanted expansion of non-telencephalic tissue was lower than in MG^{+D} conditions. Thus, we propose that when fast tissue polarization is experimentally required, liquid embedding of EBs may be advantageous in comparison to droplet embedding.*

- Line 216-218 "Overall, organoids cultured in the absence of exogenous ECM were more homogenous, and continued signaling from exogenous ECM potentiated an increased differentiation or expansion of non-telencephalic". What is the proportion on non-telencephalic tissue? No clear quantification to support the statement from either statining or scRNAseq

  We thank the referee for this comment. We had previously **quantified these data at D40 in 190 organoids** (Figure 4H and Figure S13D) and now added a quantification also at **D120 in 98 organoids** (Figure 5J and Figure S16). We believe that, together, previous and new data are sufficient to support this claim.

- Fig 5 UMAP doesn't show conditions.

  We thank the referee for this question. For clarification: in Figure 5B-C and 5E-F, the UMAP shows all conditions; in Figure 5G, the UMAP shows the three Matrigel conditions, as labeled.

- Line 303-306, please comment on cell line dependency, batch variability, organoid numbers for this conclusion.

  The excerpt mentioned by the referee is: *Another important conclusion is that Matrigel exposure in the form of a jellified matrix ($exECM^{+D}$) or transient dissolution in the medium ($exECM^{+L}$) does not critically affect early tissue morphogenesis. In fact, both formulations result in the accumulation of ECM proteins at the surface of the organoids, leading to polarity establishment and rosette formation within 3 days.*

  To tackle this point, we have greatly expanded our analysis on **rosette number from D13 to D20** (in over 400 organoids) as well as presence of **exogenous and endogenous ECM in the organoid tissue at D20**. We observe, in fact, that from D13, the **number of rosettes** formed in the presence of liquid embedding is **comparable** or superior to droplet embedding, in all cell lines. Furthermore, we confirm at D20 that the **accumulation of Matrigel** at the organoid surface is **persistent** in liquid embedding conditions. Therefore, we believe we provide sufficient new information to address these concerns and strengthen our claims.

New figure panels: Figure 2F and 2I; Fig. S2; Fig. S4

New results:

*To quantify these observations, we counted the number of PKCζ$^+$ neural rosette lumina per cross section, from D13 to D20, in over 400 organoids of all cell lines (**Fig. 2F**, **Fig. S2B-D**). The induction of rosette formation was very efficient in liquid embedding mode, as seen by comparable or higher number of neural rosettes in MG^{+L} than in MG^{+D} organoids from D13 to D20 (**Fig. 2F**, **Fig. S2B-D**). In exECM$^-$ organoids, the number of neural rosettes was initially lower (**Fig. S2B-C**) but, by D20, was comparable among all conditions in most cell lines (**Fig. S2D**).*

We show that there was a large proportion of MG-derived ECM in both MG$^+$ conditions, especially in MG^{+D} organoids, which were covered by a thicker Matrigel layer. (...) Thus, Matrigel addition led to the formation of a sheet of ECM at the outer-most organoid surface, distinguishable from, but not replacing, endogenously-produced ECM within the tissue (...)

- 344-346 "Despite these commonalities, biases in tissue patterning that favor the expansion of optic cup tissue in exECM+D organoids are already significantly higher at D40 and persist throughout time." This was not quantified properly.

We thank the referee for this comment. While the expansion of **OTX2$^+$ tissue at D40** was already previously quantified, we now provide the exact number of organoids analysed (**190 organoids** from all cell lines). We have also added an analogous analysis at **D120**, from **98 organoids** of all cell lines. Finally, these findings are also now corroborated by **transcriptional analysis at D20**. The data can be found in Figure 3D, Figure 4H, Figure S13D, Figure 5J, and Figure S16.

Once again, we are very grateful for the constructive comments of both referees, which truly helped us improve our manuscript.

1st Revision - Editorial Decision 9th Aug 2023

Dear Jürgen,

Thank you for submitting a revised version of your manuscript. Based on the input from one of the original reviewers and your responses during the pre-decision consultation, I invite you to submit the final version of your manuscript, in which you include a textual response to the issues raised by the reviewer and highlight the provided data on soluble laminin interaction with the organoids, as well as discuss the experimental challenges for detection of the exogenously provided collagen and the caveats of soluble ECM component functionality and effect on organoid development in comparison with polymerised matrices.

There are also a few editorial points that have to be addressed before I can extend the acceptance of the manuscript:

1. Our publisher has done their pre-publication check on your manuscript. I have attached the file here. Please take a look at the word file and the comments regarding the figure legends and respond to the issues.
2. Please reduce the number of keywords to five.
3. Please rename "Data and code availability" section into "Data availability".
4. Please rename "Conflict of interest" section into "Disclosure and competing interests statement" (further info: https://www.embopress.org/page/journal/14602075/authorguide#conflictsofinterest).
5. CRediT has replaced the traditional author contributions section because it offers a systematic, machine-readable author contributions format that allows for more effective research assessment. Please remove the Author Contributions from the manuscript and use the free text boxes beneath each contributing author's name in our online submission system to add specific details on the author's contribution. Further information is available in our guide to authors.
6. Please update the references according to The EMBO Journal style (up to 10 authors followed by et al.) Further information can be found here: https://www.embopress.org/page/journal/14602075/authorguide#referencesformat
7. If possible, the figure callouts should follow the alphabetic order in the manuscript text. If feasible, callout for Fig. 2B should be after 2A, 4D after 4C, S3B after S3A, S12B after S12A, S16C-D after S16B.
8. Appendix Figures S4A-D, S15A and S16F are not mentioned in the manuscript text. We are also missing a callout for Appendix Table S1.
9. Please rename the supplementary figures and tables into Appendix Figure S1/Appendix Table S1, etc. and update the nomenclature in the manuscript accordingly. Please add to the Appendix file a short table of contents that includes the page numbers.
10. Our standard prepublication image analysis shows that several panels from Figure 2 are reused in Appendix Figures S3 and S4. Please clearly state this in the figure legends.
11. Please submit source data as requested by our data editor after invitation to revise the manuscript. I have attached the checklist below - please note that the figure panel numbers refer to the previous version of the manuscript.
12. Papers published in The EMBO Journal are accompanied online by a 'Synopsis' to enhance discoverability of the manuscript. It consists of A) a short (1-2 sentences) summary of the findings and their significance, B) 3-4 bullet points highlighting key results and C) a synopsis image that is 550x300-600 pixels large (width x height, jpeg or png format). You can either show a model or key data in the synopsis image. Please note that the image size is rather small and that text needs to be readable at the final size. Please send us this information together with the revised manuscript.

Please let me know if you have any questions regarding any of these points. You can use the link below to upload the revised files.

Thank you again for giving us the chance to consider your manuscript for The EMBO Journal. I look forward to receiving the final version.

With best wishes,

Ieva

Ieva Gailite, PhD
Senior Scientific Editor
The EMBO Journal
Meyerhofstrasse 1
D-69117 Heidelberg
Tel: +4962218891309
i.gailite@embojournal.org

Further information is available in our Guide For Authors: https://www.embopress.org/page/journal/14602075/authorguide

We realize that it is difficult to revise to a specific deadline. In the interest of protecting the conceptual advance provided by the

work, we recommend a revision within 3 months (7th Nov 2023). Please discuss the revision progress ahead of this time with the editor if you require more time to complete the revisions.

Referee #2:

I have evaluated the revised version of the manuscript "Morphogenesis and development of human telencephalic organoids in the absence and presence of exogenous ECM" and while I appreciate some of the additional data and analyses presented in the revised manuscript, I still have some concerns.

My major criticism concerns the experiments where the authors are adding laminin or collagen in solution to the organoids and claim that these two components have no effect on organoid development, hence the organoids develop as in the exECM- condition. To my knowledge, Laminin or Collagen do not polymerize if they are added to the media and hence will not be available to the tissue. I could not find papers that use dissolved laminin. It is normally used for coating plates for 2D cell culture but this is a different scenario. I found this paper https://www.sciencedirect.com/science/article/pii/S0021925818311840 which states that laminin needs an acidic environment to aggregate. There is a lot of active research ongoing where bioengineers (e.g. Lutolf lab) attempt to make defined gels and fibres that include laminin or collagen, which is then available to the tissue and has potential to replace matrigel. If the authors want to make the statement that laminin or collagen alone have no effect on organoid development, they would need to show that in their experimental conditions these compounds are able to interact with the cells on the organoid surface.

The authors write:
"Interestingly, Laminin+L and Coll.IV+L organoids were comparable to exECM- organoids, presenting a smooth surface with outer brightening, and lacking the budding seen in MG+ organoids (Fig. 2K, Fig. S6B)."

This to me indicates that the experiment might not have worked. If matrigel is approximately 60% laminin and 30% Collagen, should the addition of these compounds in their biologically relevant form to the organoid not show an effect that is similar to addition of matrigel and show at least some transcriptional response by the organoid cells? I was surprised that the organoids are transcriptionally pretty much the same as the exECM- organoid cells.

I think what the authors show is that dissolving laminin and collagen in the media and adding it in this form to organoids does not influence organoid development and does not recapitulate the effect of matrigel. But the reason for this might be that the compounds are not provided in a way that the organoid cells can interact with it.

Overall, I do not think the manuscript should be published as is, as there would be confusing messages delivered to the organoid community.

The authors addressed the remaining editorial issues.

3rd Revision - Editorial Decision 14th Sep 2023

Dear Jürgen,

Thank you for addressing most of the final editorial points. I am now pleased to inform you that your manuscript has been accepted for publication.

I will look into the synopsis text in the next couple of days and let you know if any edits to the journal style are needed.

Please note that it is EMBO Journal policy for the transcript of the editorial process (containing referee reports and your response letter) to be published as an online supplement to each paper. If you do NOT want this, you will need to inform the Editorial Office via email immediately. More information is available here:
https://www.embopress.org/page/journal/14602075/authorguide#transparentprocess

Your manuscript will be processed for publication in the journal by EMBO Press. Manuscripts in the PDF and electronic editions of The EMBO Journal will be copy edited, and you will be provided with page proofs prior to publication. Please note that supplementary information is not included in the proofs.

You will be contacted by Wiley Author Services to complete licensing and payment information. The required 'Page Charges Authorization Form' is available here: https://www.embopress.org/pb-assets/embo-site/tej_apc.pdf - please download and complete the form and return to embopressproduction@wiley.com

EMBO Press participates in many Publish and Read agreements that allow authors to publish Open Access with reduced/no publication charges. Check your eligibility: https://authorservices.wiley.com/author-resources/Journal-Authors/open-access/affiliation-policies-payments/index.html

Should you be planning a Press Release on your article, please get in contact with embojournal@wiley.com as early as possible, in order to coordinate publication and release dates.

If you have any questions, please do not hesitate to call or email the Editorial Office. Thank you for this contribution to The EMBO Journal and congratulations on a successful publication!

Best regards,

Ieva

Ieva Gailite, PhD
Senior Scientific Editor
The EMBO Journal
Meyerhofstrasse 1
D-69117 Heidelberg
Tel: +4962218891309
i.gailite@embojournal.org

EMBO Press Author Checklist

Corresponding Author Name: Nina S. Corsini, Jürgen A. Knoblich	
Journal Submitted to: The EMBO Journal	
Manuscript Number: EMBOJ-2022-113213R1	

USEFUL LINKS FOR COMPLETING THIS FORM
The EMBO Journal - Author Guidelines
EMBO Reports - Author Guidelines
Molecular Systems Biology - Author Guidelines
EMBO Molecular Medicine - Author Guidelines

Reporting Checklist for Life Science Articles (updated January
This checklist is adapted from Materials Design Analysis Reporting (MDAR) Checklist for Authors. MDAR establishes a minimum set of requirements in transparent reporting in the life sciences (see Statement of Task: 10.31222/osf.io/9sm4x). Please follow the journal's guidelines in preparing your
Please note that a copy of this checklist will be published alongside your article.

Abridged guidelines for figures
1. Data
The data shown in figures should satisfy the following conditions:
- the data were obtained and processed according to the field's best practice and are presented to reflect the results of the experiments in an accurate and unbiased manner.
- ideally, figure panels should include only measurements that are directly comparable to each other and obtained with the same assay.
- plots include clearly labeled error bars for independent experiments and sample sizes. Unless justified, error bars should not be shown for technical
- if n<5, the individual data points from each experiment should be plotted. Any statistical test employed should be justified.
- Source Data should be included to report the data underlying figures according to the guidelines set out in the authorship guidelines on Data

2. Captions
Each figure caption should contain the following information, for each panel where they are relevant:
- a specification of the experimental system investigated (eg cell line, species name).
- the assay(s) and method(s) used to carry out the reported observations and measurements.
- an explicit mention of the biological and chemical entity(ies) that are being measured.
- an explicit mention of the biological and chemical entity(ies) that are altered/varied/perturbed in a controlled manner.
- the exact sample size (n) for each experimental group/condition, given as a number, not a range;
- a description of the sample collection allowing the reader to understand whether the samples represent technical or biological replicates (including how many animals, litters, cultures, etc.).
- a statement of how many times the experiment shown was independently replicated in the laboratory.
- definitions of statistical methods and measures:
  - common tests, such as t-test (please specify whether paired vs. unpaired), simple χ2 tests, Wilcoxon and Mann-Whitney tests, can be unambiguously identified by name only, but more complex techniques should be described in the methods section;
  - are tests one-sided or two-sided?
  - are there adjustments for multiple comparisons?
  - exact statistical test results, e.g., P values = x but not P values < x;
  - definition of 'center values' as median or average;
  - definition of error bars as s.d. or s.e.m.

Please complete ALL of the questions below.
Select "Not Applicable" only when the requested information is not relevant for your study.

Materials

Newly Created Materials	Information included in the manuscript?	In which section is the information available? (Reagents and Tools Table, Materials and Methods, Figures, Data Availability Section)
New materials and reagents need to be available; do any restrictions apply?	Yes	Materials and Methods.

Antibodies	Information included in the manuscript?	In which section is the information available? (Reagents and Tools Table, Materials and Methods, Figures, Data Availability Section)
For **antibodies** provide the following information:<br>- Commercial antibodies: RRID (if possible) or supplier name, catalogue number and or/clone number<br>- Non-commercial: RRID or citation	Yes	Materials and Methods and Supplementary Tables.

DNA and RNA sequences	Information included in the manuscript?	In which section is the information available? (Reagents and Tools Table, Materials and Methods, Figures, Data Availability Section)
**Short novel DNA or RNA including primers, probes**: provide the sequences.	Not Applicable	

Cell materials	Information included in the manuscript?	In which section is the information available? (Reagents and Tools Table, Materials and Methods, Figures, Data Availability Section)
**Cell lines:** Provide species information, strain. Provide accession number in repository **OR** supplier name, catalog number, clone number, and/**OR** RRID.	Yes	Materials and Methods.
**Primary cultures:** Provide species, strain, sex of origin, genetic modification status.	Not Applicable	
Report if the cell lines were recently **authenticated** (e.g., by STR profiling) and tested for mycoplasma contamination.	Yes	Materials and Methods.

Experimental animals	Information included in the manuscript?	In which section is the information available? (Reagents and Tools Table, Materials and Methods, Figures, Data Availability Section)
**Laboratory animals or Model organisms:** Provide species, strain, sex, age, genetic modification status. Provide accession number in repository **OR** supplier name, catalog number, clone number, **OR** RRID.	Not Applicable	
**Animal observed in or captured from the field:** Provide species, sex, and age where possible.	Not Applicable	
Please detail **housing and husbandry conditions**.	Not Applicable	

Plants and microbes	Information included in the manuscript?	In which section is the information available? (Reagents and Tools Table, Materials and Methods, Figures, Data Availability Section)
**Plants:** provide species and strain, ecotype and cultivar where relevant, unique accession number if available, and source (including location for collected wild specimens).	Not Applicable	
**Microbes:** provide species and strain, unique accession number if available, and source.	Not Applicable	

Human research participants	Information included in the manuscript?	In which section is the information available? (Reagents and Tools Table, Materials and Methods, Figures, Data Availability Section)
If collected and within the bounds of privacy constraints report on age, sex and gender or ethnicity for all study participants.	Not Applicable	

Core facilities	Information included in the manuscript?	In which section is the information available? (Reagents and Tools Table, Materials and Methods, Figures, Data Availability Section)
If your work benefited from core facilities, was their service mentioned in the acknowledgments section?	Yes	Acknowledgements.

Design

Study protocol	Information included in the manuscript?	In which section is the information available? (Reagents and Tools Table, Materials and Methods, Figures, Data Availability Section)
If study protocol has been **pre-registered, provide DOI in the manuscript**. For clinical trials, provide the trial registration number **OR** cite DOI.	Not Applicable	
Report the **clinical trial registration number** (at ClinicalTrials.gov or equivalent), where applicable.	Not Applicable	

Laboratory protocol	Information included in the manuscript?	In which section is the information available? (Reagents and Tools Table, Materials and Methods, Figures, Data Availability Section)
Provide DOI OR other citation details if **external detailed step-by-step protocols** are available.	Not Applicable	

Experimental study design and statistics	Information included in the manuscript?	In which section is the information available? (Reagents and Tools Table, Materials and Methods, Figures, Data Availability Section)
Include a statement about **sample size** estimate even if no statistical methods were used.	Yes	Results and materials and methods.
Were any steps taken to minimize the effects of subjective bias when allocating animals/samples to treatment (e.g. **randomization procedure**)? If yes, have they been described?	Not Applicable	
Include a statement about **blinding** even if no blinding was done.	Not Applicable	
Describe **inclusion/exclusion criteria** if samples or animals were excluded from the analysis. Were the criteria pre-established?<br><br>If sample or data points were omitted from analysis, report if this was due to attrition or intentional exclusion and provide justification.	Yes	Materials and methods.
For every figure, are **statistical tests** justified as appropriate? Do the data meet the assumptions of the tests (e.g., normal distribution)? Describe any methods used to assess it. Is there an estimate of variation within each group of data? Is the variance similar between the groups that are being statistically compared?	Yes	Materials and methods and supplementary tables.

Sample definition and in-laboratory replication	Information included in the manuscript?	In which section is the information available? (Reagents and Tools Table, Materials and Methods, Figures, Data Availability Section)
In the figure legends: state number of times the experiment was **replicated** in laboratory.	Yes	Figure legends.
In the figure legends: define whether data describe **technical or biological replicates**.	Yes	Figure legends.

Ethics

Ethics	Information included in the manuscript?	In which section is the information available? (Reagents and Tools Table, Materials and Methods, Figures, Data Availability Section)
Studies involving **human participants**: State details of **authority granting ethics approval** (IRB or equivalent committee(s), provide reference number for approval.	Not Applicable	
Studies involving **human participants**: Include a statement confirming that **informed consent** was obtained from all subjects and that the experiments conformed to the principles set out in the WMA Declaration of Helsinki and the Department of Health and Human Services Belmont Report.	Not Applicable	
Studies involving **human participants: For publication of patient photos**, include a statement confirming that consent to publish was obtained.	Not Applicable	
Studies involving experimental **animals**: State details of **authority granting ethics approval** (IRB or equivalent committee(s), provide reference number for approval. Include a statement of compliance with ethical regulations.	Not Applicable	
Studies involving **specimen and field samples:** State if relevant **permits** obtained, provide details of authority approving study; if none were required, explain why.	Not Applicable	

Dual Use Research of Concern (DURC)	Information included in the manuscript?	In which section is the information available? (Reagents and Tools Table, Materials and Methods, Figures, Data Availability Section)
Could your study fall under dual use research restrictions? Please check biosecurity documents and list of **select agents and toxins** (CDC): https://www.selectagents.gov/sat/list.htm	Not Applicable	
If you used a select agent, is the security level of the lab appropriate and reported in the manuscript?	Not Applicable	
If a study is subject to dual use research of concern regulations, is the name of the **authority granting approval and reference number** for the regulatory approval provided in the manuscript?	Not Applicable	

Reporting

The MDAR framework recommends adoption of discipline-specific guidelines, established and endorsed through community initiatives. Journals have their own policy about requiring specific guidelines and recommendations to complement MDAR.

Adherence to community standards	Information included in the manuscript?	In which section is the information available? (Reagents and Tools Table, Materials and Methods, Figures, Data Availability Section)
State if relevant guidelines or checklists (e.g., **ICMJE, MIBBI, ARRIVE, PRISMA**) have been followed or provided.	Not Applicable	
For **tumor marker prognostic studies,** we recommend that you follow the **REMARK** reporting guidelines (see link list at top right). See author guidelines, under 'Reporting Guidelines'. Please confirm you have followed these guidelines.	Not Applicable	
For **phase II and III randomized controlled trials**, please refer to the **CONSORT** flow diagram (see link list at top right) and submit the CONSORT checklist (see link list at top right) with your submission. See author guidelines, under 'Reporting Guidelines'. Please confirm you have submitted this list.	Not Applicable	

Data Availability

Data availability	Information included in the manuscript?	In which section is the information available? (Reagents and Tools Table, Materials and Methods, Figures, Data Availability Section)
Have **primary datasets** been deposited according to the journal's guidelines (see 'Data Deposition' section) and the respective accession numbers provided in the Data Availability Section?	Yes	Data and code availability.
Were **human clinical and genomic datasets** deposited in a public access-controlled repository in accordance to ethical obligations to the patients and to the applicable consent agreement?	Not Applicable	
Are **computational models** that are central and integral to a study available without restrictions in a machine-readable form? Were the relevant accession numbers or links provided?	Not Applicable	
If publicly available data were reused, provide the respective **data citations in the reference list**.	Not Applicable	
